# Mitochondrial calcium regulates lipid metabolism by modulating tethering of mitochondria to lipid droplets

**DOI:** 10.1038/s44318-026-00827-8

**Published:** 2026-07-03

**Authors:** Rebeca Acin-Perez, Essam A Assali, Michaela Veliova, Jennifer Ngo, Alexandra J Brownstein, Francisco Villalobos, Anton Petcherski, Pablo Hernansanz-Agustin, Doyeon Kim-Vasquez, Shili Xu, Mikayla Tamboline, Raquel Maria Silva, Alexander Upcher, Cynthia Shu, Daniel E Ferriss, Marc Liesa, Jose Antonio Enriquez, Israel Sekler, Orian S Shirihai

**Affiliations:** 1 Department of Medicine, Endocrinology, David Geffen School of Medicine, University of California, Los Angeles, CA USA; 2 Metabolism Theme, David Geffen School of Medicine, University of California, Los Angeles, CA USA; 3 Centro Nacional de Investigaciones Cardiovasculares Carlos III (CNIC), Madrid, Spain; 4 Centro de Investigación Biomédica en Red de Fragilidad y Envejecimiento Saludable, Madrid, Spain; 5 Department of Physiology and Cell Biology, Ben Gurion University of The Negev, Beer-Sheva, Israel; 6 Department of Molecular and Medical Pharmacology, University of California, Los Angeles, CA USA; 7 Molecular Cellular Integrative Physiology, University of California, Los Angeles, CA USA; 8 Centro de Neurociencias Cajal, (CNC-CSIC), Alcalá de Henares, Spain; 9 Crump Institute for Molecular Imaging, David Geffen School of Medicine, University of California Los Angeles, Los Angeles, CA USA; 10 Jonsson Comprehensive Cancer Center, David Geffen School of Medicine, University of California Los Angeles, Los Angeles, CA USA; 11 Electron Microscopy Unit. Ilse Katz Institute for Nanoscale Science and Technology, Ben Gurion University of the Negev, Beer-Sheva, Israel; 12 Department of Chemistry and Biochemistry, University of California, Los Angeles, USA; 13 Institut de Biologia Molecular de Barcelona, IBMB-CSIC, Barcelona, Spain; 14 CIBERDEM, Instituto de Salud Carlos III, Madrid, Spain

**Keywords:** Membranes & Trafficking, Metabolism, Signal Transduction

## Abstract

Adrenergic stimulation of brown adipocytes induces a robust detachment of mitochondria from lipid droplets (LD), which is followed by lipolysis and lipid catabolism. However, the signals inducing mitochondria attachment or detachment, and their role in lipid metabolism, remain unknown. Here, we reconstituted mitochondria-LD interaction in brown adipocyte tissue (BAT) ex vivo. We find that removal of mitochondria from lipid droplets permits higher lipolytic activity of recombinant lipases. Testing the effect of thermogenic secondary messengers and metabolites on attachment and detachment identified elevated mitochondrial matrix calcium as a potent inducer of detachment. Further, deletion of the mitochondrial sodium/calcium exchanger, NCLX, resulted in reduced attachment and increased detachment, while activation of NCLX increased attachment. We find that elevated matrix calcium causes detachment by inducing architectural transformation of peridroplet mitochondria (PDM) from their typical LD-surface-bound crescent shape into a round shape. PDE2A inhibition activates NCLX and increases PDM content in BAT in vitro and in vivo. We conclude that a surge in mitochondrial matrix calcium ions serves as a potent signal to induce mitochondrial detachment from lipid droplets, thereby facilitating lipolysis.

## Synopsis

Peridroplet mitochondria (PDM) are anchored to lipid droplets (LDs), preventing their catabolism, but what stimulates PDM departure from LDs during thermogenesis remains elusive. This study uncovers antagonistic roles for mitochondrial matrix calcium ions and sodium/calcium exchanger NCLX in determining mitochondria-LD interaction and facilitating lipolysis.

- Cell-free reconstitution of brown adipose tissue (BAT) LD-mitochondrial interaction allows the discovery of attachment and detachment signals.
- A surge in mitochondrial matrix Ca^2+^ induces detachment of PDMs from LDs, clearing the lipid droplet surface for lipolytic activity
- Sodium/calcium exchanger NCLX regulates PDM detachment from LDs.
- PDE2A inhibition in a obesity mouse model activates NCLX and increases PDM content in BAT in vivo.

## Abstract (web summary)

Mitochondrial matrix calcium and sodium/calcium exchanger NCLX antagonistically determine mitochondria-lipid droplet attachment, facilitating lipolysis in vivo.

## Graphical abstract

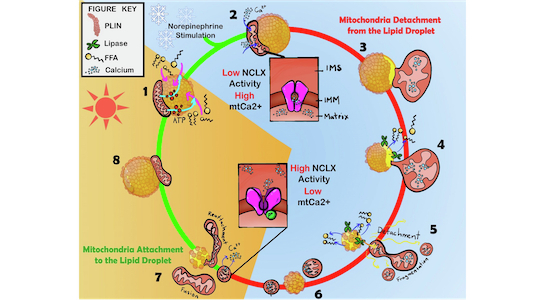

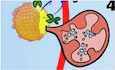

## Introduction

We recently described two distinctive mitochondrial populations in brown adipose tissue (BAT) (Benador et al, 2018). Peridroplet mitochondria (PDM) are physically attached to lipid droplets (LD), while cytosolic mitochondria (CM) are spatially separated from LD. These two mitochondrial populations display distinct metabolic features as well as fuel preferences, mitochondrial dynamics, and protein expression (Benador et al, 2018). While CM have a higher capacity to oxidize free fatty acids (FFA), PDM oxidize pyruvate to support lipid droplet expansion. This specialization and high oxidative capacity of PDM are conserved in humans and has been demonstrated in PDM from browned adipose tissue of human patients with pheochromocytoma (Acin-Perez et al, 2021). Cold-induced adrenergic stimulation results in a robust detachment of PDM from LD. Following detachment, mitochondria assume the CM phenotype characterized by fragmentation, balling, uncoupling, and lipid utilization (Acin-Perez et al, 2021; Benador et al, 2018). However, the molecular mechanisms regulating mitochondrial detachment from LDs upon adrenergic stimulation and re-attachment to LDs once the stimulus is withdrawn remains largely unknown.

BAT adrenergic stimulation triggers the activation of protein kinase A (PKA) signaling (Collins, 2011; Virtue and Vidal-Puig, 2013) as well as calcium (Ca^2+^) mobilization (Lee et al, 1993; Mohell, 1984; Pappone and Lee, 1995). Among PKA phosphorylation targets are enzymes involved in lipolysis such as Hormone Sensitive Lipase (HSL) (Holm, 2003; Lorente-Cebrian et al, 2011; Miyoshi et al, 2006) and Adipose Triglyceride Lipase (ATGL) (Hoy et al, 2011; Miyoshi et al, 2008; Pagnon et al, 2012; Schoiswohl et al, 2015), as well as the LD-coating proteins Perilipins (PLIN1-6) (Hansen et al, 2017; McDonough et al, 2013; Miyoshi et al, 2006; Sztalryd and Brasaemle, 2017). A member of the PLIN family, perilipin 5 (Plin5), mediates PDM interaction with LDs in BAT (Benador et al, 2019; Benador et al, 2018; Gallardo-Montejano et al, 2021; Kimmel and Sztalryd, 2014; Wang et al, 2011). Its role in lipolysis regulation has been studied in numerous tissues (Gallardo Montejano et al, 2026; Gao et al, 2024; Kang et al, 2026; Kawecka et al, 2025; Keenan et al, 2021; Kolleritsch et al, 2020; Liu and Zhao, 2025; Pollak et al, 2015; Zhang et al, 2025).

Parallel to the cyclic adenosine monophosphate (cAMP) signaling wave, Ca^2+^ also contributes to cold-adaptive thermogenesis, although its regulatory role and its contribution to lipid mobilization is poorly understood. The response of brown adipocytes (BA) to adrenergic stimulation is characterized by a transient Ca^2+^ surge in the mitochondrial matrix. The first phase of the surge is mediated by Ca^2+^ influx through the mitochondrial calcium uniporter (MCU). Within minutes, the rise in matrix Ca^2+^ is followed by a sharp decline in calcium due to the activation of calcium efflux by the Ca^2+^ extrusion transporter or mitochondrial sodium/calcium exchanger, NCLX, which exports Ca^2+^ in exchange for sodium (De Stefani et al, 2015; Marchi and Pinton, 2014). Mitochondrial Ca^2+^ (mt-Ca^2+^) can activate mitochondrial soluble adenylate cyclase (sAC, ADCY10) (Litvin et al, 2003; Steegborn, 2014), leading to an increase in the mitochondrial cAMP pool and can activate mitochondrial PKA (Acin-Perez et al, 2009). There are several known PKA targets in mitochondria (Acin-Perez et al, 2011a; Balaban, 2010; Boja et al, 2009; Hopper et al, 2006; Zhao et al, 2011), including NCLX (Boyman et al, 2013). Acting as the efflux arm of the Ca^2+^ surge, NCLX prevents the rise in Ca^2+^ induced by the adrenergic stimulation from turning into a toxic Ca^2+^ overload and cell death event. Ca^2+^ extrusion by NCLX is regulated by PDE2A, a unique phosphodiesterase (PDE) harbored in the mitochondria (Rozenfeld et al, 2022). Phosphorylation of NCLX by PKA activates its function, while dephosphorylation by PDE2A decreases NCLX activity.

Here, we describe an assay that successfully reconstitutes the interaction of mitochondria with LD ex vivo, enabling us to study the capacity of different agents to regulate mitochondrial-LD interactions. We demonstrate that PDM retain higher capacity to attach to LDs compared to CM. Furthermore, we demonstrate that mitochondrial binding to LD ex vivo is dependent on the substrate being oxidized by mitochondria, and that increased mitochondrial matrix Ca^2+^ is sufficient to detach mitochondria from LD ex vivo. Extrusion of Ca^2+^ by NCLX promotes attachment of mitochondria to LD, while NCLX inactivation by dephosphorylation promotes detachment.

## Results

### LD-mitochondrial interaction can be studied and modeled in a reconstituted in vitro assay

**Figure 1:**
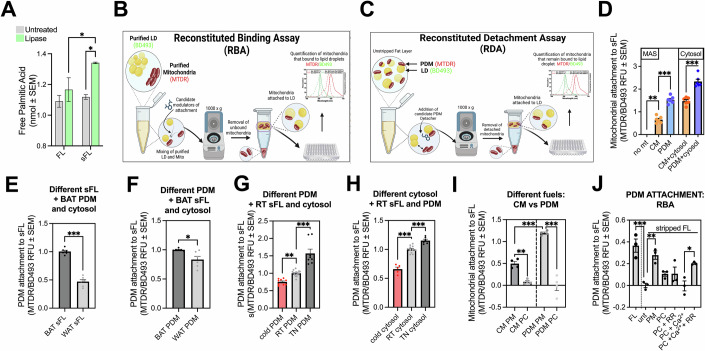
Purified peridroplet mitochondria (PDM) retain intrinsic lipid droplet (LD) binding capacity in a reconstituted binding assay that identifies attachment regulators (see also Fig. EV1 and Appendix Table S1). (**A**) Effect of PDM removal on lipolysis. In vitro assay measuring free palmitic acid release from fat layer (FL) and stripped fat layer (sFL) following exposure to recombinant lipase. BAT FL was treated with lipase for 20 min before and after mechanical removal of mitochondria by centrifugation (*n* = 2). Two-way ANOVA test. (**B**) Reconstituted Binding Assay (RBA) schematic. Following isolation of the FL together with bound PDM, mitochondria are mechanically stripped to generate purified PDM and sFL. In the RBA, purified mitochondria are mixed with sFL in a cell-free system. Candidate metabolites or ions are added to the reaction. Unbound mitochondria are removed by low-speed centrifugation, and mitochondria carried with LDs are quantified using fluorescent probes. (**C**) Reconstituted Detachment Assay (RDA) schematic. Detachment of mitochondria from LDs is assessed using purified FL containing mitochondria-bound LDs. Candidate regulators are added, and remaining LD-associated mitochondria are quantified. (**D**) Intrinsic attachment capacity of CM and PDM to BAT-derived sFL in MAS buffer or in the presence of purified cytosol (*n* ≥ 6). Purified PDM retain superior LD-binding capacity, which is enhanced by cytosol. Ordinary one-way ANOVA test. (**E**, **F**) Tissue-specific determinants of binding. (**E**) RBA using BAT-derived PDM and cytosol with sFL isolated from BAT or WAT (*n* = 6). Data are normalized to BAT sFL. (**F**) RBA using BAT-derived sFL and cytosol with PDM isolated from BAT or WAT (*n* = 6). Data are normalized to BAT PDM. Unpaired *t* test. (**G**, **H**) Temperature-dependent regulation of binding. (**G**) PDM isolated from BAT of mice housed at thermoneutrality (TN), room temperature (RT), or cold were tested for binding to RT-derived sFL in the presence of RT-derived cytosol (*n* ≥ 8). Data are normalized to RT PDM. (**H**) Cytosol isolated from BAT of mice housed at TN, RT, or cold was tested for its ability to support binding of RT-derived PDM to RT-derived sFL (*n* ≥ 5). Data are normalized to RT cytosol. Ordinary one-way ANOVA test. (**I**) Substrate dependence of attachment. RBA testing CM and PDM binding to sFL in the presence of pyruvate + malate (PM) or palmitoyl-carnitine (PC) in MAS buffer (*n* = 4). Ordinary one-way ANOVA test. (**J**) Calcium regulation of attachment. RBA assessing the effects of Ca²⁺ and the mitochondrial calcium uniporter (MCU) inhibitor Ruthenium Red (RR) on PDM attachment to BAT-derived sFL under the indicated substrate conditions (*n* = 3). Ordinary one-way ANOVA test. (**A**,** D**, **E**–**J**) Each point represents a biological replicate; technical replicates were averaged. Data are mean ± SEM. *P < 0.05; **P < 0.01; ***P < 0.001; ****P < 0.0001. Source data are available online for this figure.

Previous studies demonstrate that lipase action on LDs is facilitated by clearance of the LD surface (Kory et al, 2016; Kory et al, 2015; Olzmann and Carvalho, 2019). Thermogenic signals induce the detachment of mitochondria from LDs while enhancing lipolytic activity, leading to shrinkage in LD size and number. However, the role of mitochondrial detachment from LD in the activation of lipid utilization remains unclear. PDM detachment could either enhance lipolysis due to PDM no longer contributing to building the LD, or alternatively, because their removal enables lipases access to the LD surface. To explore this possibility, we conducted a proof-of-concept experiment comparing the capacity of lipases to act on LDs before and after the removal of PDM from the LD surface. We incubated both the fat layer (FL), consisting of LD surrounded by PDM, and stripped fat layer (sFL), consisting of naked LDs, with lipases and measured FFA release. Removal of mitochondria from LD resulted in increased lipolytic activity, reflected by higher palmitic acid release (Fig. 1A). These results suggest that PDM can serve as a lipolytic barrier and that detachment of PDM from LD may serve to accelerate LD utilization and fatty acid-fueled thermogenesis.

To directly study the mechanisms regulating PDM attachment and detachment, we first developed a cell-free, reconstituted PDM–LD interaction assay (Fig. 1B). Because PDM represent only ~20% of total mitochondria in BAT (Benador et al, 2019; Benador et al, 2018), we initially separated CM from PDM by exploiting the buoyancy of LDs, which selectively co-float with PDM. We then detached PDM from LDs by centrifugation, yielding two fractions in the final step: purified PDM and stripped LDs (stripped FL, sFL) (Ngo et al, 2021).

Using this approach, which we term the Reconstituted Binding Assay (RBA), isolated mitochondria were reintroduced to sFL, and PDM reattachment to LDs was quantitatively assessed. Reattachment was measured based on the buoyant behavior of LDs: mitochondria that reattached to LDs co-migrated to the top fraction, whereas non-attached mitochondria remained in the lower phase and were recovered by low-speed centrifugation. To enable quantification, mitochondria and LDs were labeled with the mitochondrial dye MitoTracker Deep Red (MTDR) that binds both depolarized and polarized mitochondria (Acin-Perez et al, 2020; Tovar-Ferrero et al, 2025) and the neutral lipid dye BODIPY 493/503 (BD493**)**, respectively. Reattachment efficiency was calculated as the ratio of mitochondrial to LD fluorescence intensity in each sample.

To quantify the rate of PDM detachment from LDs, we developed a Reconstituted Detachment Assay (RDA). In this assay, freshly isolated LDs together with their co-purified PDM were collected from tissue homogenates during fat layer separation, yielding an intact FL that had not been stripped of mitochondria. We then incubated the FL with candidate agents to test their ability to induce mitochondrial detachment from LDs. Following incubation, samples were subjected to slow-speed centrifugation, allowing mitochondria that had detached from LDs to partition into the infranatant, while LDs with remaining attached mitochondria remained in the buoyant fraction. The extent of detachment was quantified by measuring the ratio of mitochondrial (MTDR) to LD (BD493) fluorescence in the buoyant fraction (Fig. 1C) (Acin-Perez et al, 2021).

In parallel, we complemented these ex vivo measurements with confocal imaging of primary brown adipocytes (pBA). This combined approach allowed us to distinguish agents that act directly on LD–mitochondria interactions from those that require intact cellular signaling to promote PDM detachment.

### Validation of reconstituted cell free RBA and RDA assays

**Figure EV1:**
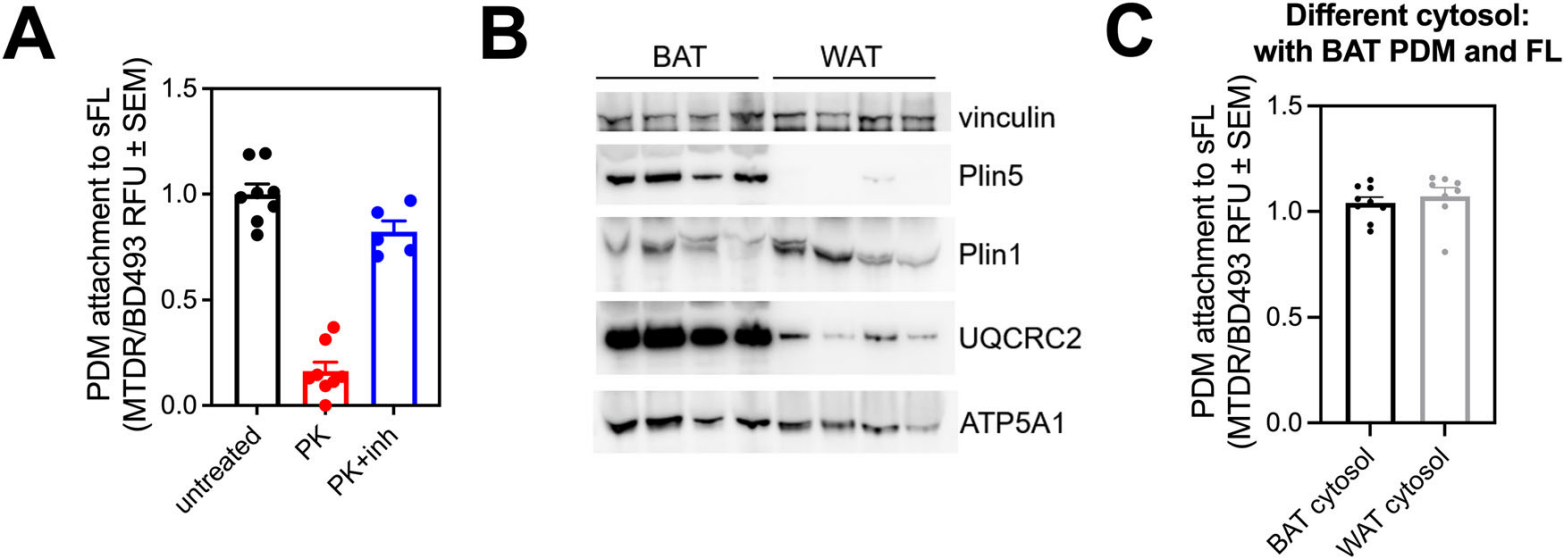
Proteins coating the LD are important in tethering mitochondrial interaction with LD. (**A**) Quantification of BAT PDM attached to LD in the presence of proteinase K and proteinase K inhibitors in MAS buffer (*n* ≥ 5). (**B**) Western blot in WAT and BAT lysates using the indicated antibodies. (**C**) Comparative analysis of attachment efficacy of mitochondria, LD and cytosol from BAT vs WAT. Reconstituted binding assay using FL and PDM from BAT and either WAT or BAT cytosol (*n* ≥ 9). (**A**, **C**) Each point represents a biological replica sample. For each biological replicate, technical replicates were averaged. Data represent average ± SEM. Source data are available online for this figure.

Next, we set out to validate the assays by testing the responses to interventions that are expected to alter mitochondria-LD interaction, thereby corroborating the specificity of mitochondria and LD binding. Contact sites between PDM and LDs depend on tethering proteins (Benador et al, 2019; Cui and Liu, 2020; Dejgaard and Presley, 2021; Sztalryd and Brasaemle, 2017). To validate the method, we performed an RBA in the presence of proteases and examined their effect on mitochondria-LD tethering in mitochondrial assay solution buffer (MAS) (Fig. EV1). Our results show that the highly active serine protease, Proteinase K completely prevented mitochondria from binding LDs. The preventative effect of Proteinase K on re-attachment was blunted by ~85% when the FL was pre-incubated with a cocktail mix of protease inhibitors (Fig. EV1A), confirming that proteins are crucial for mitochondria-LD attachment in the RBA assay.

To determine whether mitochondria retain subtype-specific binding properties outside the cellular context, we examined the binding specificity of CM and PDM in the RBA. Specifically, we asked whether isolated CM and PDM maintain differential affinity for LDs after removal from cells and stripping from LDs. RBA was performed by incubating sFL layers (used as the reference baseline) with either purified CM or PDM in MAS buffer (Fig. 1D; Appendix Table S1).

To assess the contribution of cytosolic metabolites, ions, and proteins to mitochondrial attachment, MAS buffer was replaced with purified, organelle-depleted cytosol from BAT tissue. Using this system, we observed that purified PDM exhibited significantly higher binding affinity to LDs than CM (1.5–2.5-fold), independent of the reaction buffer, indicating that PDM and CM possess distinct intrinsic binding properties that are preserved after dissociation from LDs (Fig. 1D). In addition, cytosol increased attachment of both mitochondrial populations to LDs by 1.5–2.5-fold compared to MAS buffer alone, suggesting that soluble cytosolic factors facilitate LD–mitochondria binding.

Previous studies have shown that white adipose tissue (WAT) and BAT from cold-exposed mice contain fewer PDM than BAT from mice housed at room temperature (RT) (Acin-Perez et al, 2021; Mirza et al, 2021; Yu et al, 2015). To further interrogate binding specificity and to assign regulatory contributions to LDs, mitochondria, or soluble factors, we compared the binding capacity of PDM, sFL, and cytosol isolated from BAT and gonadal WAT of mice housed at RT.

Using sFL derived from either BAT or WAT and incubating both with BAT-derived PDM and cytosol in the RBA, we found that sFL from WAT markedly reduced PDM attachment compared with sFL from BAT (Fig. 1E; Appendix Table S1). This reduction likely reflects differences in LD surface composition between tissues, consistent with distinct LD-associated protein profiles (Fig. EV1B). We next asked whether the tissue origin of PDM itself influences attachment. When sFL and cytosol from BAT were used as the binding substrate, PDM isolated from BAT exhibited a significantly higher attachment rate to LDs than PDM isolated from WAT (Fig. 1F; Appendix Table S1).

Finally, we evaluated the contribution of cytosolic factors by varying the tissue source of cytosol. Stripped FL and PDM from BAT attached at comparable levels in the presence of cytosol derived from either BAT or WAT (Fig. EV1C; Appendix Table S1), suggesting that, under RT conditions, the soluble factors that modulate mitochondrial–LD binding are largely conserved between these adipose depots.

### Identification of the signal controlling mitochondrial-LD binding

Previous studies in BAT have shown that cold exposure promotes detachment of PDM from LDs, whereas thermoneutrality (TN, 28 °C) enhances PDM association with LDs (Acin-Perez et al, 2021; Benador et al, 2018). This bidirectional response indicates that mitochondrial–LD interactions are physiologically regulated and can be acutely modulated by thermogenic signals. We reasoned that if the RBA preserves these temperature-dependent differences in PDM–LD association, it could be used to identify the molecular signals controlling attachment and detachment.

To test this, we performed RBAs using PDM isolated from BAT of mice housed at cold (4 °C), RT, or TN (Fig. 1G; Appendix Table S1). In this experiment, stripped LDs and cytosol were consistently derived from BAT of RT-housed mice, allowing the contribution of mitochondrial-intrinsic properties to be isolated. Under these conditions, PDM from TN-housed mice exhibited the highest binding affinity to LDs (∼1.5-fold higher than RT), whereas PDM from cold-exposed mice showed the lowest binding (∼25% lower than RT). Thus, temperature-dependent differences in PDM–LD association observed in vivo are retained in a cell-free system, indicating that a substantial component of binding regulation is encoded within the mitochondria themselves. These findings further validate the specificity of the RBA and its ability to recapitulate physiologically relevant PDM behavior ex vivo.

We next asked whether thermogenic response also alters soluble cytosolic modulators of mitochondrial–LD binding. To address this, we performed RBAs in which both PDM and LDs were isolated from RT-housed mice, while the cytosol was derived from BAT of mice housed at different temperatures. Using this design, we found that cytosol from cold-exposed BAT reduced PDM attachment to LDs by ∼40% compared with cytosol from TN-housed mice (Fig. 1H; Appendix Table S1).

Together, these results demonstrate that BAT cytosol contains temperature-regulated soluble factors capable of modulating mitochondrial–LD binding and that both mitochondrial-intrinsic properties and cytosolic components contribute to the dynamic regulation of PDM association during thermogenic remodeling.

### Mitochondrial calcium controls attachment and detachment of PDM to and from LD

To investigate the mechanism underlying cytosol-mediated regulation of mitochondrial–LD adherence, we considered cytosolic changes that accompany adrenergic activation of BAT. Thermogenic stimulation induces marked transition in metabolite availability, shifting from pyruvate, the preferred PDM fuel, to acyl-carnitines, the preferred CM fuel (Acin-Perez et al, 2021; Benador et al, 2018). We therefore tested whether the identity of the mitochondrial respiratory substrate influences PDM–LD interactions using the RBA by supplementing the assay buffer (MAS) with defined fuels.

When pyruvate plus malate (PM) was provided as substrate, PDM attachment to LDs was approximately twofold higher than when palmitoyl-carnitine (PC) was used (Fig. 1I; Appendix Table S1). In contrast, CM attachment to LDs was not significantly affected by substrate choice. These findings indicate that PDM–LD association is selectively sensitive to metabolic substrate availability, consistent with the metabolic specialization of PDM.

Because these effects occurred on a minute timescale, matching that of acute thermogenic activation, we next evaluated cytosolic signals that rise rapidly during thermogenesis, including increased lipolysis and β-oxidation, elevated cytosolic Ca²⁺, and transient increases in mitochondrial matrix Ca²⁺ (Abou-Rjeileh and Contreras, 2021; Dumonteil et al, 1994; Maus et al, 2017; Von Bank et al, 2021). To test the role of Ca²⁺ in mitochondrial–LD attachment under thermogenic-like conditions, we adapted the RBA to include PC as the dominant substrate. As a reference value for maximal attachment, we used the intact FL, consisting of LDs co-purified with their associated mitochondria.

Under these conditions, addition of Ca²⁺ markedly reduced PDM attachment to LDs, nearly abolishing PDM attachment to LD (Fig. 1J; Appendix Table S1). To determine whether this effect was mediated by mitochondrial matrix Ca²⁺ uptake, we inhibited Ca²⁺ entry into mitochondria using the MCU inhibitor Ruthenium Red (RR). RR fully prevented the Ca²⁺-dependent reduction in attachment, indicating that mitochondrial matrix Ca²⁺ is required for regulation of PDM–LD association (Fig. 1J).

**Figure 2:**
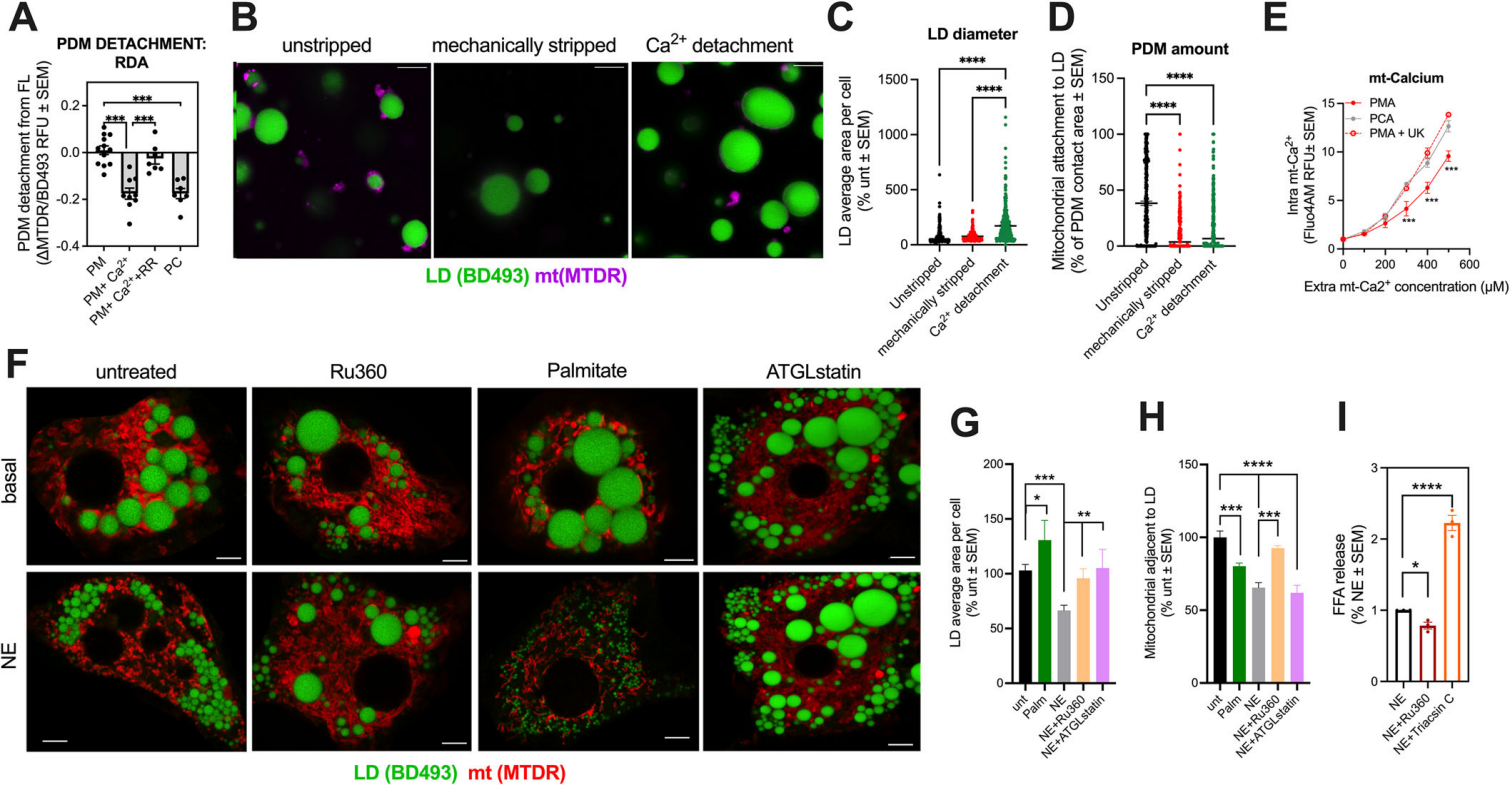
A surge in mitochondrial matrix Ca²⁺ induces detachment of peridroplet mitochondria (PDM) from lipid droplets (see also Fig. EV2 and Appendix Tables S1 and S2). (**A**–**D**) Cell-free Reconstituted Detachment Assay (RDA) in isolated BAT fat layer (FL). (**A**) Calcium- and substrate-dependent detachment. Lower values show detachment; zero value is the level of attachment in the isolated sample. Role of matrix Ca²⁺ is tested by the co-treatment with the MCU inhibitor Ruthenium Red (RR) (*n* ≥ 7). PM pyruvate-malate, PC palmitoyl carnitine. Ordinary one-way ANOVA test. (**B**) Super-resolution confocal images of FL, mechanically stripped FL, and FL subjected to RDA in the presence of Ca²⁺. Ca²⁺ induces mitochondrial departure from LDs without mechanical stripping. Mitochondria were stained with MitoTracker Deep Red (MTDR) and LDs with BODIPY 493/503 (BD493). (**C**) Quantification of LD diameter from super-resolution images in FL, mechanically stripped FL, and FL after Ca²⁺-induced detachment (*n* > 180 LDs). Ordinary one-way ANOVA test. (**D**) Quantification of PDM–LD contact area from super-resolution images under the indicated conditions (*n* > 180 LDs). Ordinary one-way ANOVA test. (**E**) Measurement of intramitochondrial Ca²⁺ using Fluo-4 AM under the indicated substrate conditions and in the presence of UK-5099 (UK), an inhibitor of mitochondrial pyruvate import. (*n* ≥ 2). Row statistics. (**F**–**I**) Calcium- and substrate-dependent detachment in intact brown adipocytes (BA). (**F**) Super-resolution confocal images of live cultured BA under the indicated conditions. Mitochondria were labeled with MTDR and LDs with BODIPY 493/503. (**G**) Effect of the MCU inhibitor Ru360 10 µM and ATGLstatin 50 µM on norepinephrine (NE)-induced LD shrinkage. Integrated LD area per cell is shown (*n* ≥ 18). Mixed-effects analysis and Welch’s test. (**H**) Effect of Ru360 and ATGLstatin on NE-induced PDM detachment. Mitochondria adjacent to LDs were quantified as mitochondrial pixels located within 0.3 µm of an LD. Notably, NE-induced detachment was not inhibited by ATGLstatin, indicating that detachment precedes lipolysis (*n* ≥ 18). Mixed-effects analysis and Welch’s test. (**I**) Effect of Ru360 on NE-induced free fatty acid (FFA) release from BA. Medium- and long-chain non-esterified fatty acids in culture media were quantified as described in “Methods” (*n* = 3). Ru360 reduced FFA release, supporting a role for mitochondrial matrix Ca²⁺ elevation in lipolysis. As a control for maximal lipolysis, cells were treated with NE+Triacsin-C to inhibit re-esterification. Ordinary one-way ANOVA test. Scale bars: 5 µm in (**B**); 10 µm in (**F**). Ca²⁺ concentrations represent effective free Ca²⁺ after correction for EGTA chelation in MAS buffer. Cells were treated with vehicle, Ru360 (10 µM), palmitate (200 µM; 4:1 palmitate:BSA), or ATGLstatin (50 µM) and imaged 2 h after stimulation with NE (1 µM). In panels (**A**, **C**–**E**, **G**–**I**): each point represents a biological replicate; technical replicates were averaged. Data are mean ± SEM. *P < 0.05; **P < 0.01; ***P < 0.001; ****P < 0.0001. Source data are available online for this figure.

We next asked whether the reduced attachment observed in the RBA reflected Ca²⁺-induced detachment rather than impaired binding per se. To address this, we employed the RDA (Fig. 1C), using intact FL labeled with MTDR and BD493. As in the RBA, RDA experiments were performed with either PM or PC, in the presence or absence of Ca²⁺ (10 µM). PC alone promoted PDM detachment from LDs, consistent with its reduced attachment in the RBA (Fig. 2A). Importantly, combining PC with Ca²⁺ further enhanced detachment, resulting in 35–40% of PDM separating from the fat layer. This Ca²⁺-induced detachment was entirely dependent on mitochondrial Ca²⁺ uptake, as it was fully blocked by RR (Fig. 2A; Appendix Table S1).

For these experiments, the intact FL served as a pre-detachment reference condition. In Fig. 2A, detachment is represented as a negative deviation from this baseline, allowing direct comparison of detachment capacity across metabolic conditions. Notably, the inability to reach maximal attachment in the presence of Ca²⁺ (Fig. 1J) mirrors the enhanced detachment observed in the RDA (Fig. 2A), supporting a unified mechanism.

To exclude the possibility that centrifugation artifacts contributed to the observed detachment, we independently validated the effect of Ca²⁺ using confocal imaging of FL before and after Ca²⁺ exposure. Imaging revealed that addition of Ca²⁺ resulted in LDs retaining a reduced number of attached PDM, while detached mitochondria were visible in the surrounding medium (Fig. 2B). Quantitative analysis showed that both mechanical stripping and Ca²⁺-induced detachment reduced PDM abundance without reducing LD size (Fig. 2C,D), confirming that detachment occurs independently of LD shrinkage, since LD surface area did not decrease. These findings corroborate the biochemical assays and are consistent with Fig. 1A, supporting the conclusion that lipolysis occurs downstream of mitochondrial detachment.

### Pyruvate and palmitoyl-carnitine may modulate mitochondrial binding to LD via their effect on matrix Ca^2+^

Mitochondrial matrix free Ca^2+^ levels control attachment and detachment of PDM to and from LD and that attachment is dependent on fuel utilization. The results described in Fig. 2A show that PC is sufficient to reproduce the same detachment effect as PM plus Ca^2+^, suggesting that PC could be triggering an intramitochondrial Ca^2+^ effect. These results suggested the possibility that the effect of the different fuels on attachment and detachment is mediated via their effects on matrix Ca^2+^. To test whether different fuels could be Ca^2+^ modifiers, we measured mt-Ca^2+^ concentrations in isolated mitochondria that were loaded with the mitochondrial matrix Ca^2+^ probe, Fluo4AM. As substrates, mitochondria were treated with either PM (PMA; pyruvate malate +ADP), or PC (PCA; palmitoyl carnitine + ADP). We found that treatment with PMA resulted in lower matrix Ca^2+^ compared to PCA (Fig. 2E). To confirm that pyruvate entry was required for the Ca^2+^ effect, we blocked its entry to the matrix using the mitochondrial pyruvate carrier (MPC) inhibitor UK5099. Indeed, inhibition of the MPC eliminated the differences between increased intramitochondrial Ca^2+^ in PMA to similar levels to those of PC (Fig. 2E).

The regulation of matrix Ca^2+^ by pyruvate and PC raises the possibility that the effect of these metabolites on mitochondrial attachment and detachment from LD is mediated via their effect on matrix Ca^2+^, further emphasizing a possible role for Ca^2+^ as a unifying regulator of mitochondria-LD attachment.

### Validation of Ca^2+^ and PC as PDM detachers in intact primary brown adipocytes

**Figure EV2:**
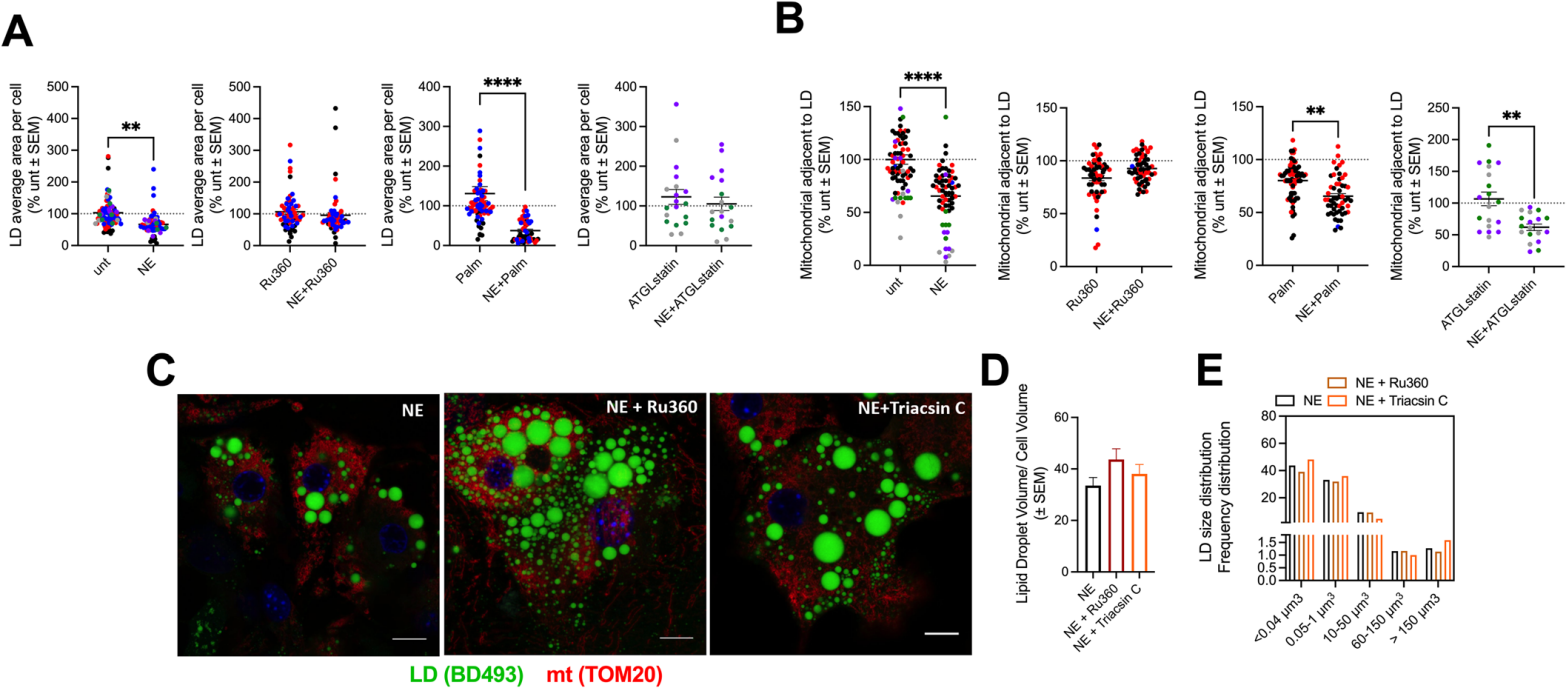
Effect of modulating re-esterification in lipid droplet size and distribution in pBA. (**A**, **B**) Integrated LD area per cell (**A**) and PDM (**B**) were quantified as mitochondrial pixels located within 0.3 µm of a lipid droplet in pBA under the indicated conditions (*n* ≥ 18). (**C**) Mitochondria (red, TOM20) and LD (green, BD493) 3D imaging in fixed BA under the indicated treatments. Scale bar = 10 µm. (**D**) Quantification of the volume of the cell occupied by LD versus the total volume of the cell in pBA subjected to the indicated treatments (*n* ≥ 25). (**E**) Analysis of LD size distribution in pBA subjected to the indicated treatments. Data represent average ± SEM. **P < 0.01; ****P < 0.0001. (**A**, **B**) Mixed-effects analysis and Welch’s test. Source data are available online for this figure.

To validate the findings obtained using the reconstituted binding and detachment assays (RBA and RDA), we performed quantitative image analysis in pBA under basal conditions or following norepinephrine (NE) stimulation for 2 h (Fig. 2F–I). NE treatment alone induced mitochondrial detachment from lipid droplets (LDs) and a concomitant reduction in LD diameter (Figs. 2F,G and EV2A; Appendix Table S2). Mitochondrial detachment and LD shrinkage were inhibited when NE was combined with the MCU inhibitor Ru360, indicating that mitochondrial Ca²⁺ entry is required for both detachment and lipolysis in intact cells (Figs. 2F–H and EV2A,B; Appendix Table S2). Together with the inhibitory effect of MCU blockade in the cell-free RDA (Fig. 2A), these results support a direct role for mitochondrial matrix Ca²⁺ in regulating mitochondrial detachment from LDs.

Cell-free RDA experiments further showed that PC alone is sufficient to promote PDM detachment (Fig. 2A). To assess whether this effect is recapitulated in cells, pBA were treated with palmitate (200 µM;4:1 palmitate:BSA) either alone or in combination with NE (Figs. 2F–H and EV2A,B; Appendix Table S2). Palmitate treatment under basal conditions resulted in enlarged LDs and reduced PDM association, consistent with enhanced lipid storage. As expected, NE stimulation induced LD shrinkage and increased mitochondrial detachment (Fig. EV2A,B).

Finally, to determine whether PDM detachment precedes lipolysis, we pharmacologically inhibited ATGL using ATGLstatin. Under these conditions, NE stimulation failed to reduce LD size but still promoted mitochondrial detachment from LDs (Figs. 2F–H and EV2A,B; Appendix Table S2), demonstrating that detachment occurs upstream of triglyceride hydrolysis. Free fatty acid release was inhibited when NE was combined with Ru360, indicating that mt-Ca²⁺ entry is required for both detachment and lipolysis in intact cells (Fig. 2I). Triacsin C, an inhibitor of long fatty acyl-coA synthetase, was used as control (Figs. 2I and  EV2C–E).

Collectively, these results validate the reconstituted assays in intact pBA and corroborate the findings shown in Fig. 1A, supporting the conclusion that PDM act as a lipolytic barrier. Moreover, they indicate that mitochondrial detachment from LDs is an early, Ca²⁺-dependent event that facilitates subsequent LD utilization and fatty acid–driven thermogenesis.

### Ca^2+^ signaling in CM versus PDM

**Figure EV3:**
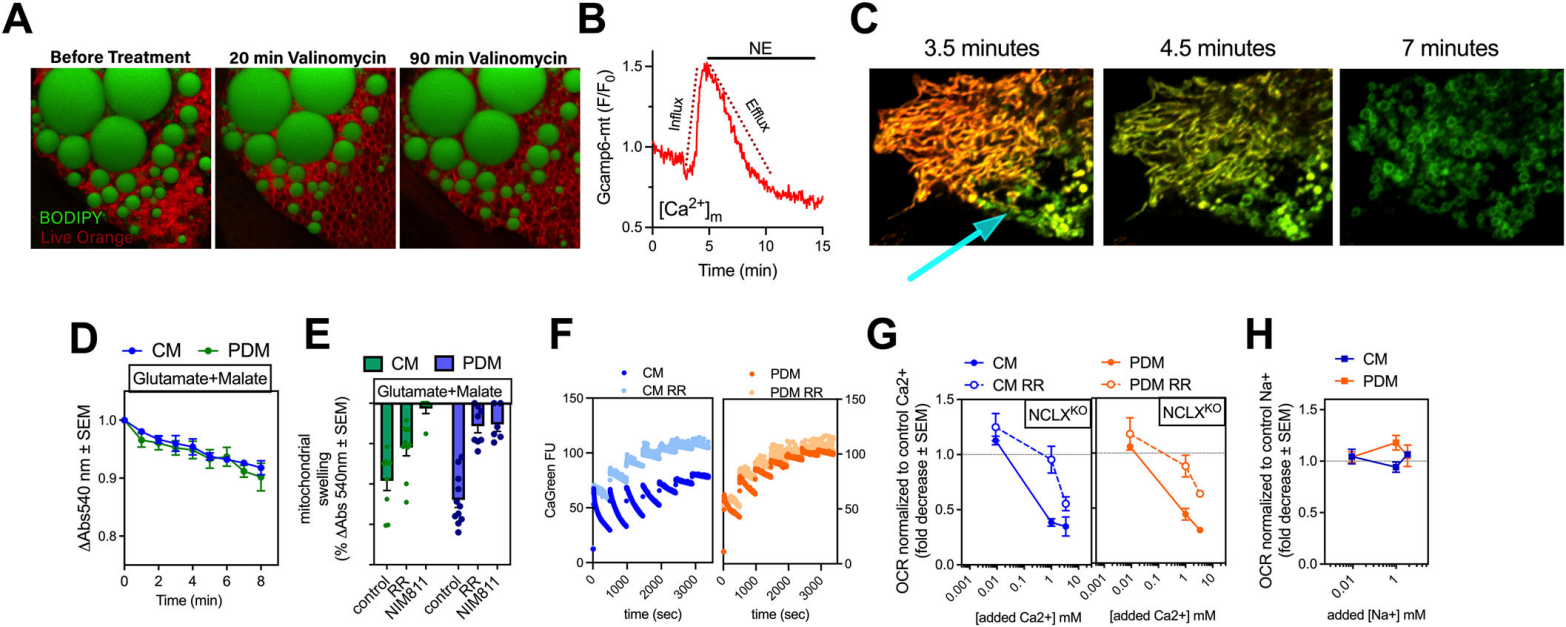
NE induces a transient surge of calcium in mitochondria that is associated with a change in mitochondrial architecture. (**A**) Super-resolution confocal images pBA in the presence of Valinomycin for the indicated times. Valinomycin induces swelling and PDM detachment. Mitochondria were stained with Live Orange and LDs with BODIPY 493/503 (BD493). (**B**) Representative trace of mt-Ca^2+^ influx and efflux in primary adipocytes using mt-GCaMP6. (**C**) Effect of NE on mitochondrial architecture and Δψ_m_ in primary brown adipocytes at different time points. Adipocytes were stained with TMRE (red) and Mitotracker Green (green). Depolarized mitochondria lose red TMRE staining and become green while mitochondria that maintain Δψ_m_ show yellow-orange mitochondrial staining. Note that NE-stimulation results in a mitochondrial shape change from elongated to fragmented and swollen mitochondria. From Wikstrom et al, 2014. (**D**, **E**) mt-Ca^2+^ induced swelling assay in CM and PDM isolated from WT mice using Glutamate+malate as substrates (*n* ≥ 6), and in the presence or absence of NIM811, a pharmacological inhibitor of the mitochondrial permeability pore (mt-PTP) modulator, Cyclophylin D (CypD). (**F**) Representative traces of buffer Ca^2+^ following the sequential addition of 50 µM bolus of CaCl_2_ in respiring isolated CM and PDM. RR is used as a negative control of Ca^2+^ entry in mitochondria. (**G**) PC-dependent respiration in BAT CM (blue) and PDM (orange) from NCLX^KO^ mice under the indicated Ca^2+^ concentration and in the absence or presence of RR (*n* = 3). Note that deletion of NCLX eliminates the differences between PDM and CM shown in Fig. 3I,J, resulting in CM taking the same Ca^2+^ sensitivity phenotype as PDM. (**H**) Testing whether altered sodium concentrations contribute to the observed Ca²⁺ sensitivity due to NCLX activity. State 3 PC-dependent respiration was measured in CM (blue) and PDM (orange) from WT mice under the varying sodium concentrations (*n* = 6). Note that changes in sodium concentrations did not impair respiration. Each point represents a biological replica sample. For each biological replicate, technical replicates were averaged. Data represent average ± SEM. Source data are available online for this figure.

Previous studies have shown that mitochondrial detachment from LDs in BAT is accompanied by a pronounced change in mitochondrial morphology: from elongated networks to rounded, swollen structures, following NE stimulation (Fig. EV3C) (Benador et al, 2018, Wikstrom et al, 2014). This observation raised the possibility that mitochondrial architectural changes contribute directly to the detachment process. Because elevations in mitochondrial matrix Ca²⁺ are known to induce mitochondrial swelling and “balling,” we asked whether swelling alone is sufficient to drive mitochondrial detachment from LDs.

**Figure 3:**
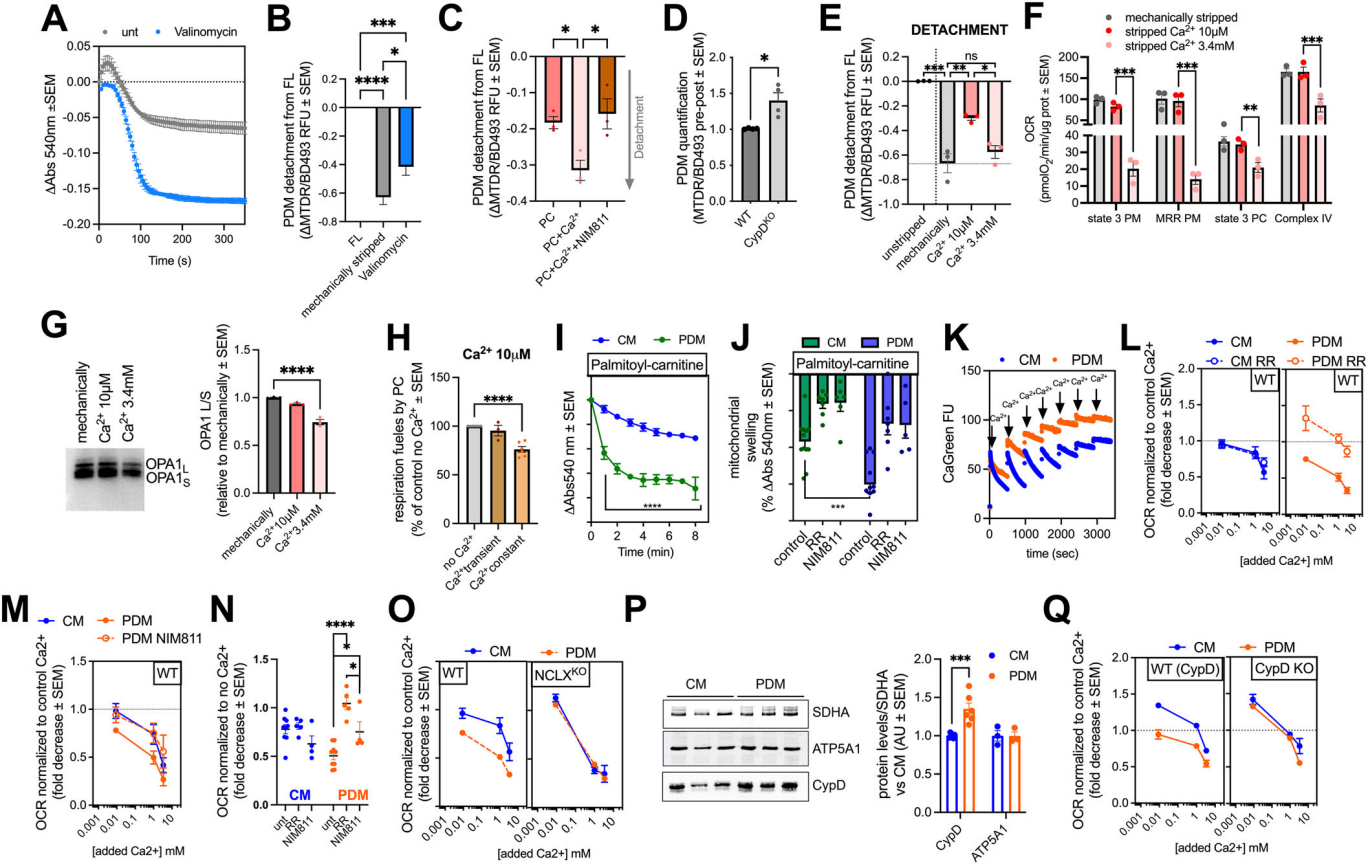
Calcium-induced changes in mitochondrial architecture drive PDM detachment from lipid droplets (see also Fig. EV3 and Appendix Table S3). (**A**, **B**) Mitochondrial swelling promotes detachment. (**A**) Mitochondrial swelling assay measured by 540 nm absorbance in isolated WT BAT mitochondria treated with valinomycin in the presence of succinate and rotenone (*n* ≥ 3). (**B**) Effect of valinomycin-induced swelling on PDM detachment from the fat layer (FL), assessed using the Reconstituted Detachment Assay (RDA) (*n* = 4). Ordinary one-way ANOVA test. (**C**, **D**) Role of transient permeability transition in mediating detachment. (**C**) RDA performed on BAT FL treated with the Cyclophilin D (CypD) inhibitor NIM811. Mechanically stripped FL (centrifugation) serves as a benchmark of maximal detachment; unstripped FL serves as a negative control (*n* = 3). Ordinary one-way ANOVA test. (**D**) In vivo quantification of PDM adherence to LDs in WT and CypD^KO^ BAT from mice housed at room temperature (*n* = 4). Unpaired *t* test. (**E**) Comparison of PDM detachment induced by mechanical stripping versus Ca²⁺-mediated stripping at physiological (10 µM) or toxic (3.4 mM) Ca²⁺ concentrations (*n* = 3). Ordinary one-way ANOVA test. (**F**–**H**) Physiological transient permeability versus irreversible permeability transition. (**F**) Oxygen consumption rates (OCR) of mitochondria stripped mechanically or biochemically. State 3 respiration (under indicated substrates), maximal respiration rate (MRR), and Complex IV-driven OCR were measured (*n* = 3). High Ca²⁺ concentrations impair respiratory function. Two-way ANOVA. (**G**) Representative immunoblot and quantification of OPA1 long-to-short (L/S) isoform ratio in mitochondria exposed to 10 µM or 3.4 mM Ca²⁺ (*n* = 3). Ordinary one-way ANOVA test. (**H**) Palmitoyl-carnitine (PC)-dependent state 3 respiration in PDM following transient (10 min) versus sustained (40 min) Ca²⁺ exposure, expressed as percentage of untreated control (*n* = 3). Ordinary one-way ANOVA test. (**I**–**K**) Differential Ca²⁺ sensitivity of PDM and CM. (**I**) Ca²⁺-induced swelling kinetics in CM and PDM isolated from WT mice in the presence of PC (*n* ≥ 6). Row statistics. (**J**) Quantification of swelling ± NIM811, demonstrating CypD dependence (*n* ≥ 5), two-way ANOVA. (**K**) Ca²⁺ retention capacity (CRC) in CM and PDM, shown as representative CaGreen traces following sequential 50 µM CaCl₂ additions. (**L**–**O**) PDM exhibit heightened sensitivity to Ca²⁺-induced respiratory impairment. (**L**) State 3 PC-driven respiration in CM (blue) and PDM (orange) under increasing Ca²⁺ concentrations ± Ruthenium Red (RR) (*n* ≥ 5). (**M**) State 3 PC-driven respiration ± NIM811 (*n* = 4). (**N**) OCR in CM and PDM at 1 mM Ca²⁺, normalized to respiration prior to Ca²⁺ addition (*n* ≥ 4). RR inhibits Ca²⁺ entry into the matrix. Two-way ANOVA. (**O**) PC-driven respiration in CM and PDM from WT and NCLX^KO^ mice under increasing Ca²⁺ concentrations (*n* = 3). Deletion of NCLX abolishes the differential Ca²⁺ sensitivity between CM and PDM observed in WT. (**P**, **Q**) CypD abundance contributes to differential Ca²⁺ sensitivity. (**P**) Western blot and quantification of CypD distribution in CM and PDM (*n* ≥ 3). Two-way ANOVA (**Q**) PC-driven respiration in CM and PDM from CypD^KO^ and WT littermates under increasing Ca²⁺ concentrations (*n* = 3). Deletion of CypD eliminates differential Ca²⁺ sensitivity. Data in panels L–M are normalized to untreated controls (no added Ca²⁺). Each point represents a biological replicate; technical replicates were averaged. Reported Ca²⁺ concentrations reflect effective free Ca²⁺ after correction for EGTA chelation in MAS buffer. (**B**–**J**, **N**–**P**) Each point represents a biological replicate; technical replicates were averaged. Data are mean ± SEM. *P < 0.05; **P < 0.01; ***P < 0.001; ****P < 0.0001. Source data are available online for this figure.

To isolate the mechanical contribution of swelling while avoiding Ca²⁺ influx, we induced mitochondrial swelling using valinomycin, a K⁺ ionophore that promotes osmotic water entry into the mitochondrial matrix. Using the RDA, we found that treatment with valinomycin for 10 min was sufficient to induce mitochondrial detachment from LDs (Fig. 3A,B), indicating that mitochondrial swelling per se can promote detachment independently of Ca²⁺ entry. Valinomycin effect in PDM swelling and detachment was validated using imaging techniques in pBA (Fig. EV3A).

We next tested whether Ca²⁺-induced detachment occurs through a Ca²⁺-dependent mitochondrial shape transition. Calcium-induced mitochondrial swelling is well characterized and is mediated by cyclophilin D (CypD), a matrix protein that serves as a Ca²⁺ sensor for mitochondrial shape changes associated with transient permeability transition. This transient permeability transition is defined by reversible matrix swelling that is CypD-dependent and preserves mitochondrial membrane integrity and respiratory function, in contrast to the permanent permeability transition associated with apoptosis (Agarwal et al, 2017). To determine whether the Ca²⁺-induced detachment observed in Fig. 2A requires CypD, we performed RDA experiments in the presence of the CypD inhibitor NIM811. Inhibition of CypD completely prevented Ca²⁺-induced mitochondrial detachment from LDs (Fig. 3C; Appendix Table S3), linking Ca²⁺-dependent detachment to a CypD-mediated mitochondrial shape transition.

To assess the relevance of this mechanism in vivo, we quantified PDM abundance BAT from CypD knockout (CypD^KO^) mice. Consistent with impaired Ca²⁺-induced detachment, CypD^KO^ BAT exhibited increased PDM abundance compared with controls (Fig. 3D). Together, these results demonstrate that elevations in mitochondrial matrix Ca²⁺ promote PDM detachment through a CypD-dependent mitochondrial shape transition.

To confirm that Ca²⁺-induced detachment is not associated with permanent permeability transition or mitochondrial dysfunction, we next examined the kinetics of Ca²⁺ transients and their impact on mitochondrial respiratory function.

### Mitochondrial shape transition in response to matrix Ca^2+^ surge is transient and does not compromise respiration

Thermogenic activation is accompanied by a transient elevation in mitochondrial matrix Ca²⁺ that is physiological, reversible, and does not induce cell death. In contrast, sustained Ca²⁺ overload triggers permanent permeability transition, irreversible loss of respiratory capacity, and apoptosis. These distinctions raise the question of whether the transient Ca²⁺ surge that promotes PDM detachment preserves mitochondrial respiratory function. To address this, we directly assessed respiratory capacity following Ca²⁺ exposures that induce mitochondrial detachment. Previous work, together with our measurements of matrix Ca²⁺ dynamics and mitochondrial architecture in BAT, indicates that both the Ca²⁺ surge and associated mitochondrial swelling occur within a time window of less than 10 min (Strubbe-Rivera et al, 2021) (Fig. EV3B,C). To experimentally mimic this transient Ca²⁺ exposure, we induced PDM detachment from the FL using Ca²⁺ in the RDA, then rapidly reduced extracellular Ca²⁺ by removing mitochondria from the detachment medium and subsequently assessed respiratory function. As a control, mitochondria detached mechanically by centrifugation without Ca²⁺ exposure were analyzed in parallel. Under these conditions, transient Ca²⁺ exposure did not impair mitochondrial respiratory capacity, indicating that Ca²⁺-induced detachment per se is not detrimental to mitochondrial function.

As a positive control for Ca²⁺ overload and mitochondrial toxicity, we treated FL with a high Ca²⁺ concentration (3.4 mM), which is expected to trigger permanent permeability transition and apoptosis (Giorgi et al, 2012). Detachment of PDM under these conditions resulted in a marked loss of respiratory capacity (Fig. 3E,F; Appendix Table S3), confirming the functional distinction between transient and pathological Ca²⁺ exposure.

To further assess whether Ca²⁺-induced detachment in the RDA reflects a reversible physiological process rather than irreversible mitochondrial damage, we analyzed processing of optic atrophy protein 1 (OPA1), a key regulator of inner mitochondrial membrane structure and cristae integrity (Acin-Perez et al, 2018; Baker et al, 2014; Ehses et al, 2009; Head et al, 2009; Kushnareva et al, 2013). Stress conditions associated with respiratory dysfunction and cytochrome c release are characterized by cleavage of long OPA1 isoforms into shorter forms, resulting in a reduced long-to-short (L/S) OPA1 ratio (Cogliati et al, 2013; Wolf et al, 2019). We therefore quantified OPA1 isoforms in PDM detached either mechanically or via Ca²⁺ exposure in the RDA. Whereas high Ca²⁺ (3.4 mM) markedly reduced the OPA1 L/S ratio, exposure to 10 µM Ca²⁺, the concentration used to induce detachment in the RDA did not alter OPA1 processing (Fig. 3G), indicating preservation of inner membrane structure.

Finally, we directly compared the effects of transient versus prolonged exposure to 10 µM Ca²⁺. While transient Ca²⁺ exposure preserved mitochondrial respiratory capacity, sustained exposure to the same Ca²⁺ concentration resulted in respiratory impairment (Fig. 3H). Together, these results demonstrate that the Ca²⁺ surge associated with PDM detachment represents a physiological, reversible signal that does not compromise mitochondrial integrity or function, in contrast to prolonged Ca²⁺ overload.

### CM differ from PDM in their response to Ca^2+^ surge

Although CM are not physically associated with LDs, they are nonetheless exposed to the cytosolic Ca²⁺ surge that accompanies thermogenic activation. Notably, we found that CM are markedly more resilient to Ca²⁺ overload than PDM. When energized with PC, PDM exhibited significantly greater Ca²⁺-induced swelling than CM (∼1.5-fold), whereas no such difference was observed when glutamate–malate was used as substrate (Figs. 3I,J and EV3D,E). These data indicate that differential sensitivity to Ca²⁺ is both mitochondrial-subtype specific and substrate dependent.

This difference was further supported by measurements of Ca²⁺ retention capacity, which reflects the amount of Ca²⁺ mitochondria can accumulate before triggering permeability transition. CM displayed a higher Ca²⁺ retention capacity than PDM, indicating greater resistance to Ca²⁺-induced permeability transition (Figs. 3K and EV3F). Ca²⁺-induced swelling of PDM was blunted by pre-incubation with either RR or the CypD inhibitor NIM811, confirming that swelling depends on Ca²⁺-mediated activation of the mitochondrial permeability transition pore (Figs. 3J and EV3D,E). Inhibition of Ca²⁺ entry through MCU using RR eliminated this difference, demonstrating that the recorded effect in this assay was Ca²⁺ dependent.

To assess the functional consequences of this differential Ca²⁺ sensitivity, we compared the ability of CM and PDM to maintain respiratory function under increasing Ca²⁺ concentrations in the presence of PC. Increasing free Ca²⁺ progressively reduced OCR in both populations; however, PDM were significantly more sensitive to Ca²⁺-induced respiratory inhibition than CM (Fig. 3L). Inhibition of Ca²⁺ entry or blockade of CypD abolished the heightened sensitivity of PDM to Ca²⁺, indicating that increased matrix Ca²⁺ accumulation and activation of permeability transition account for the differential respiratory response (Fig. 3L–N).

We next tested whether impaired Ca²⁺ extrusion contributes to the increased Ca²⁺ sensitivity of PDM. Deletion of NCLX eliminated the difference in Ca²⁺ sensitivity between CM and PDM observed in wild-type mitochondria (Fig. 3O), indicating that differential NCLX activity or expression underlies the heightened vulnerability of PDM to Ca²⁺ overload. Consistent with this interpretation, inhibition of Ca²⁺ entry with RR prevented Ca²⁺-induced respiratory impairment in NCLX-deficient PDM (Fig. EV3G), confirming that the effect of NCLX loss is mediated by excessive matrix Ca²⁺ accumulation. To exclude the possibility that Na⁺ handling rather than Ca²⁺ transport accounted for these differences, we treated isolated CM and PDM with increasing Na⁺ concentrations. Na⁺ exposure did not reproduce the differential respiratory effects observed with Ca²⁺ (Fig. EV3H), supporting a specific role for Ca²⁺ transport via NCLX.

Finally, the enhanced resilience of CM to Ca²⁺-induced permeability transition prompted us to examine expression of CypD, the Ca²⁺ sensor of the mt-PTP. Immunoblot analysis revealed lower CypD levels in CM compared with PDM (Fig. 3P). To directly test the contribution of CypD, we compared Ca²⁺ sensitivity in CM and PDM isolated from CypD knockout (CypD^KO^) and wild-type mice. Deletion of CypD abolished the differential sensitivity of CM and PDM to Ca²⁺ overload (Fig. 3Q), confirming a central role for CypD in mediating subtype-specific Ca²⁺ responses.

Together, these results demonstrate that PDM and CM differ fundamentally in their susceptibility to Ca²⁺-induced permeability transition, reflecting coordinated differences in Ca²⁺ extrusion and mt-PTP regulation. They further indicate that the transient nature of the NE-induced Ca²⁺ surge is essential for enabling PDM detachment without triggering irreversible permeability transition or loss of respiratory function, underscoring the importance of efficient Ca²⁺ extrusion mechanisms in safeguarding mitochondrial integrity during thermogenic activation.

### PDM and CM in the same cell have disparate Ca2+ response to adrenergic stimulation

Our results suggest that the kinetics of the Ca²⁺ surge are a critical determinant of the mitochondrial response to adrenergic stimulation, implicating both Ca²⁺ influx and efflux as key regulatory parameters. To directly test this, we quantified mitochondrial matrix Ca²⁺ dynamics in cultured pBA following NE stimulation.

**Figure 4:**
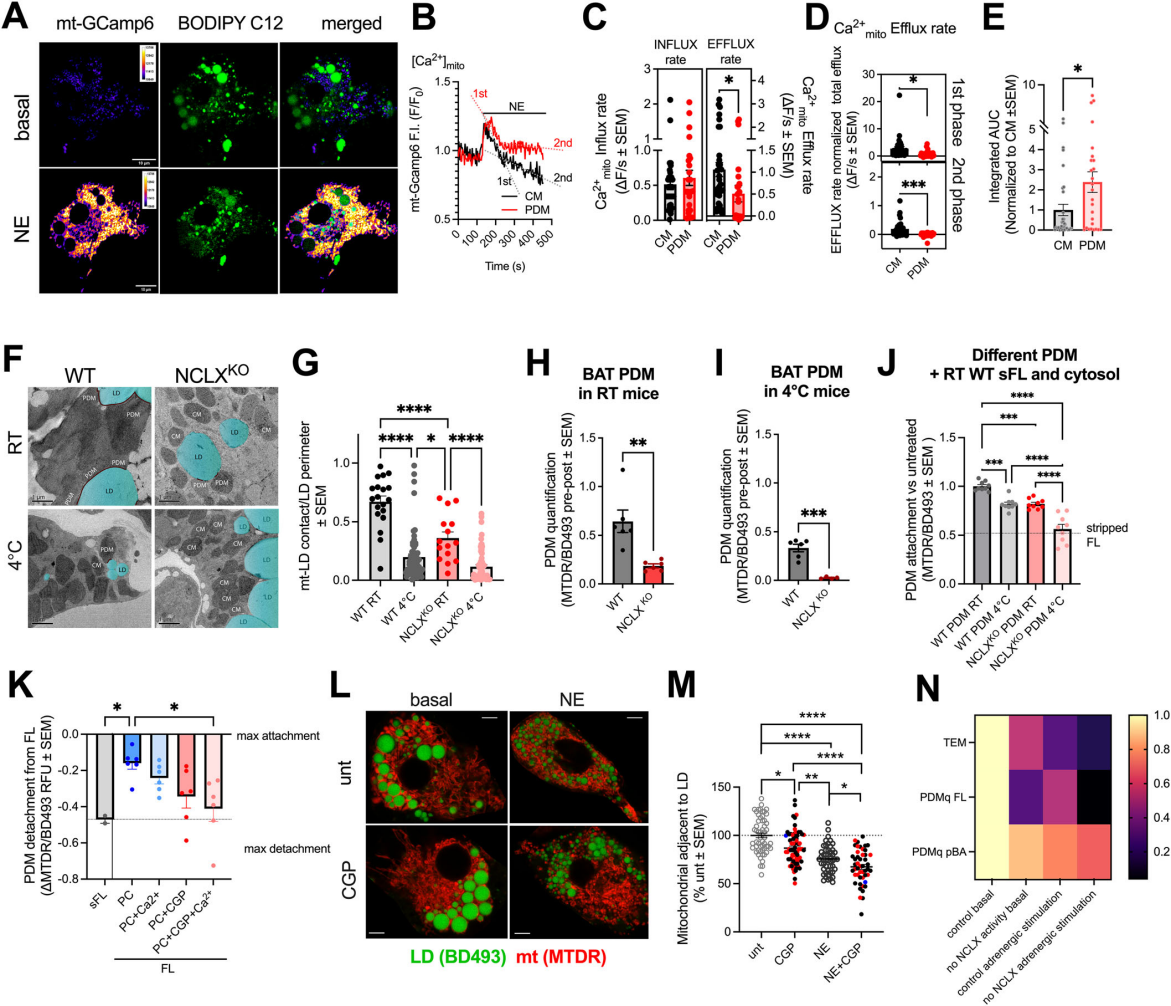
Ca²⁺ efflux through NCLX regulates PDM detachment from lipid droplets (see also Appendix Tables S2 and S4). (**A**–**E**) Differential mitochondrial Ca²⁺ handling in PDM and CM. (**A**) Representative images of mitochondrial Ca²⁺ (mt-Ca²⁺) dynamics in pBA expressing mt-GCaMP6. Images are shown using a hot-metal color scale. LDs were labeled with BODIPY C12 (green). Cells were imaged under basal conditions and 2 h after stimulation with NE (1 µM). Scale bar = 10 µm. (**B**) Representative mt-Ca²⁺ traces in PDM and CM following NE application. Shaded areas (red, PDM; green, CM) represent the integrated area under the curve (AUC; quantified in (**E**)). (**C**) Quantification of mitochondrial Ca²⁺ influx (left) and efflux (right) rates in CM and PDM (*n* ≥ 18). Unpaired *t* test. (**D**) Quantification of first and second phases of mt-Ca²⁺ efflux in CM and PDM (*n* ≥ 18). (**E**) Integrated AUC of mt-Ca²⁺ responses normalized to CM (*n* ≥ 18). Unpaired *t* test. (**F**–**I**) NCLX regulates mitochondrial–LD association in vivo. (**F**) Transmission electron microscopy (TEM) images of BAT from WT and NCLX^KO^ mice housed at RT or after cold exposure (4 °C). Scale bar = 1 µm. (**G**) Quantification of mitochondrial–LD contact normalized to LD perimeter (*n* ≥ 15). Ordinary one-way ANOVA. (**H**) Quantification of PDM attachment in WT and NCLX^KO^ BAT at RT (*n* = 6). Unpaired *t* test. (**I**) Quantification of PDM attachment in WT and NCLX^KO^ BAT following 5 h cold exposure (*n* ≥ 4). Unpaired *t* test. (**J**, **K**) Cell-free validation of NCLX-dependent regulation. (**J**) RBA using cytosol and sFL from RT-housed WT mice, combined with PDM isolated from WT or NCLX^KO^ mice housed at the indicated temperatures (*n* = 9). Ordinary one-way ANOVA. (**K**) RDA using FL under the indicated conditions. sFL serves as a maximal detachment control (*n* = 6). Ordinary one-way ANOVA. (**L**, **M**) Pharmacological inhibition of NCLX in intact cells. (**L**) Super-resolution confocal microscopy of live pBA labeled with MitoTracker Deep Red (MTDR) and BODIPY 493/503 (BD493). (**M**) Quantification of PDM adjacency to LDs (mitochondrial pixels within 0.3 µm of LD surface). Cells were treated with vehicle or CGP-37157 (CGP, 10 µM) and imaged 2 h after NE stimulation (1 µM). (*n* ≥ 48). Scale bar = 10 µm. (**N**) Heatmap comparing three independent methodologies to determine PDM amount in basal or adrenergic stimulation conditions in controls or when NCLX activity is absent (either genetically or pharmacologically). (**A**–**K**) Each point represents a biological replicate; technical replicates were averaged. Data are mean ± SEM. *P < 0.05; **P < 0.01; ***P < 0.001; ****P < 0.0001. Source data are available online for this figure.

Mitochondrial matrix Ca²⁺ concentrations were monitored by expressing the genetically encoded Ca²⁺ reporter mt-GCaMP6. To distinguish PDM from CM, cells were co-stained with the neutral lipid dye BODIPY 558/568-C12 (BODIPY C12) 1 h prior to imaging (Fig. 4A). NE stimulation triggered an immediate increase in mitochondrial matrix Ca²⁺ in both PDM and CM, as indicated by a rapid rise in mt-GCaMP6 fluorescence (Fig. 4A). Analysis of the initial slope of fluorescence increase revealed comparable rates of Ca²⁺ uptake in CM and PDM (Fig. 4B,C), indicating similar Ca²⁺ influx kinetics.

In contrast, Ca²⁺ efflux kinetics differed markedly between the two mitochondrial populations. In CM, Ca²⁺ efflux rapidly returned matrix Ca²⁺ to near-basal levels within ~2 min of the NE-induced peak, followed by a slower secondary phase that reduced matrix Ca²⁺ below pre-stimulation baseline (Fig. 4B). This biphasic behavior closely resembles previously reported mitochondrial Ca²⁺ dynamics in brown adipocytes exposed to NE (Assali et al, 2020). By comparison, PDM exhibited significantly slower Ca²⁺ efflux during both the rapid and slow phases of Ca²⁺ clearance (Fig. 4C,D).

The cumulative consequence of these kinetic differences is captured by the area under the curve (AUC), which integrates matrix Ca²⁺ levels over time and was significantly greater in PDM than in CM (Fig. 4E). These prolonged Ca²⁺ elevations in PDM provide a mechanistic explanation for their increased susceptibility to Ca²⁺-induced swelling, permeability transition, and respiratory impairment observed in Fig. 3I,J,L–N.

### NCLX regulates PDM detachment

To determine the role of NCLX in regulating mitochondrial detachment from LDs, we quantified PDM in BAT from NCLX knockout (NCLX^KO^) mice. Transmission electron microscopy (TEM) was performed on BAT from WT and NCLX^KO^ mice housed at RT or following 5 h of cold exposure (Fig. 4F). Quantitative TEM analysis revealed that NCLX^KO^ BAT contained significantly fewer PDM than WT BAT under RT conditions (Fig. 4G). Cold exposure further reduced PDM abundance in both genotypes; notably, in NCLX^KO^ BAT, cold exposure resulted in near-complete loss of detectable PDM at the LD surface (Fig. 4G).

To confirm that the TEM-based measurements reflected bona fide PDM attachment rather than mitochondrial proximity to LDs, we independently quantified PDM biochemically by isolating the FL from BAT and measuring mitochondrial content. PDM abundance was assessed in BAT from WT and NCLX^KO^ mice housed at RT (Fig. 4H) or cold-exposed for 5 h (Fig. 4I). Consistent with the imaging data, NCLX^KO^ BAT exhibited significantly fewer PDM per LD than WT controls under basal conditions, and cold exposure further reduced PDM abundance in both genotypes. These results indicate that loss of NCLX, and consequent impairment of mitochondrial Ca²⁺ extrusion, recapitulates key features of adrenergic stimulation–induced PDM detachment.

We next asked whether the effect of NCLX on PDM attachment is intrinsic to mitochondria. Using the RBA, we compared the ability of PDM isolated from WT or NCLX^KO^ mice to bind LDs isolated from WT mice housed at RT, in the presence of WT cytosol. PDM were isolated from WT or NCLX^KO^ mice maintained at RT or cold-exposed for 5 h, while LDs and cytosol were held constant to isolate mitochondrial-intrinsic effects. Under these conditions, PDM from NCLX^KO^ mice exhibited a significantly lower attachment rate than WT PDM, and attachment was further reduced by cold exposure (Fig. 4J; Appendix Table S4), consistent with in vivo observations.

To determine whether acute inhibition of NCLX is sufficient to promote mitochondrial detachment, we performed the RDA using FLs isolated from WT mice while pharmacologically inhibiting NCLX with CGP-37157 (CGP) (De La Fuente et al, 2018; Ruiz et al, 2014). Acute NCLX inhibition significantly increased mitochondrial detachment from LDs (Fig. 4K; Appendix Table S4). Moreover, combining CGP with PC and Ca²⁺ further enhanced detachment, indicating additive effects among these detachment-promoting factors and recapitulating the pronounced PDM loss observed in cold-exposed NCLX^KO^ mice (Fig. 4K).

Together, these results demonstrate that PDM detachment is governed by mitochondrial matrix Ca²⁺ handling and is regulated, at least in part, by NCLX-mediated Ca²⁺ extrusion.

Consistent with this conclusion, imaging of pBA demonstrated that pharmacological inhibition of NCLX induced PDM detachment (Fig. 4L,M; Appendix Table S2). Remarkably, across three independent methodologies used to quantify mitochondrial–LD association—TEM, fluorescence imaging of freshly isolated FL, and live-cell imaging of pBA (Fig. 4N)—inhibition or deletion of NCLX consistently reduced PDM abundance relative to control conditions.

Adrenergic stimulation, induced either by cold exposure in vivo or by NE treatment in cultured pBA, reduced PDM levels to a degree comparable to that observed following NCLX inhibition. Notably, combining adrenergic stimulation with NCLX inhibition or deficiency resulted in the most pronounced reduction in PDM across all conditions tested (Fig. 4N), indicating that NCLX activity restrains detachment and that its loss potentiates the effects of thermogenic signaling.

### Phosphodiesterase 2A negatively regulates NCLX in PDM

The results above indicate that CM and PDM differ in their capacity to handle Ca²⁺, with this divergence arising from two separable mechanisms: regulation of matrix Ca²⁺ levels by NCLX, and differential sensitivity to matrix Ca²⁺ mediated by CypD. Although both CM and PDM express NCLX, these findings raise the possibility that NCLX activity is differentially regulated between mitochondrial subtypes.

**Figure 5:**
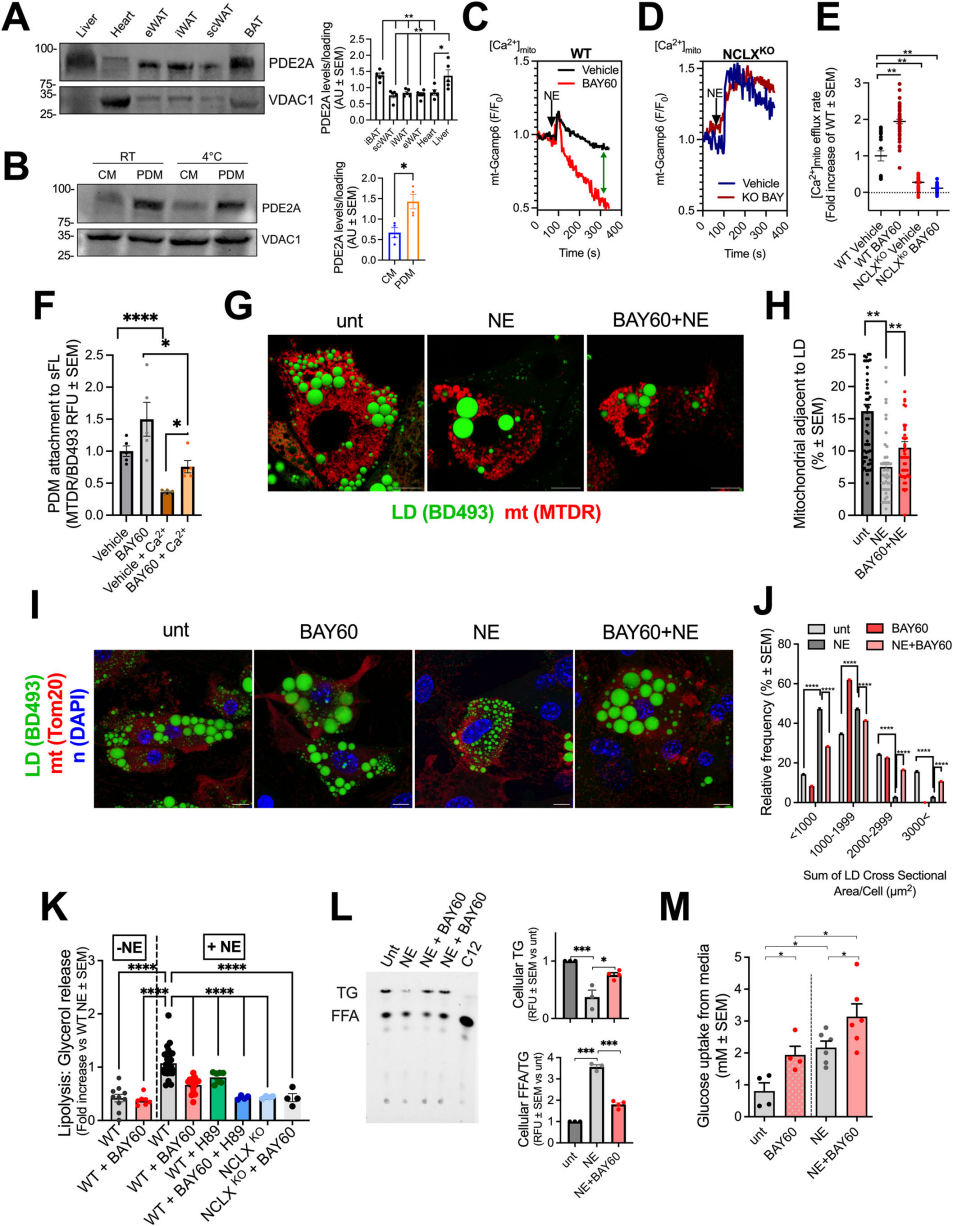
Phosphodiesterase 2A (PDE2A) negatively regulates NCLX activity in PDM, promoting mitochondrial detachment and lipolysis (see also Appendix Tables S2 and S4). (**A**) Western blot and quantification of PDE2A protein levels in lysates from multiple tissues (*n* = 5). Ordinary one-way ANOVA. (**B**) Western blot and quantification of PDE2A levels in CM and PDM isolated from BAT of WT mice housed at RT (*n* = 4). Unpaired *t* test. (**C**–**E**) PDE2A inhibition accelerates NCLX-dependent Ca²⁺ efflux. (**C**) Mitochondrial matrix Ca²⁺ dynamics in WT pBA pre-treated with BAY60 (10 µM, 1 h) and stimulated with NE, monitored using mt-GCaMP6. (**D**) Mitochondrial Ca²⁺ dynamics in NCLX^KO^ pBA treated with vehicle or BAY60. In the absence of NCLX, BAY60 does not alter Ca²⁺ kinetics. (**E**) Quantification of Ca²⁺ efflux rates in WT and NCLX^KO^ pBA under vehicle or BAY60 treatments (*n* ≥ 20). Ordinary one-way ANOVA. (**F**) Cell-free validation of PDE2A regulation. RBA testing the effect of BAY60 (10 µM) and Ca²⁺ (10 µM) on PDM attachment to sFL (*n* = 5). Ca²⁺ inhibits PDM–LD binding, which is partially restored by BAY60. Unpaired *t* test. (**G**, **H**) PDE2A inhibition prevents NE-induced PDM detachment in intact cells. (**G**) Super-resolution confocal microscopy of live pBA labeled with MitoTracker Deep Red (MTDR) and BODIPY 493/503. (**H**) Quantification of mitochondrial adjacency to LDs (mitochondrial pixels within 0.3 µm of LD surface) under basal and NE conditions ± BAY60 (*n* ≥ 5). Scale bar = 2 µm. Ordinary one-way ANOVA. (**I**, **J**) PDE2A inhibition prevents NE-induced LD breakdown. (**I**) Super-resolution confocal images of pBA under basal or NE stimulation ± BAY60 (10 µM). (**J**) Quantification of LD frequency and size distribution based on cross-sectional area per cell (*n* ≥ 31). BAY60 preserves large LDs and prevents NE-induced LD shrinkage. Scale bar = 10 µm. (**K**) PDE2A inhibition suppresses lipolysis in an NCLX-dependent manner. Glycerol release from WT and NCLX^KO^ pBA under basal and NE conditions ± BAY60 (6 h stimulation; *n* ≥ 5). BAY60 reduces lipolysis in WT but not in NCLXKO cells. Two-way ANOVA. (**L**) Thin-layer chromatography (TLC) and quantification of triglyceride (TG) and free fatty acid (FFA) content in pBA treated with BAY60 (10 µM, 6 h) (*n* = 4). Ordinary one-way ANOVA. (**M**) Glucose uptake assessed by measuring reduction in media glucose (initial concentration 5 mM) under the indicated conditions (*n* ≥ 4) after 6 h incubation. BAY60 enhances glucose utilization, consistent with reduced fatty acid mobilization. Ordinary one-way ANOVA. (**A**, **B**, **E**, **F**, **H**, **J**–**M**) Each point represents a biological replicate; technical replicates were averaged. Data are mean ± SEM. *P < 0.05; **P < 0.01; ****P < 0.0001. Source data are available online for this figure.

Recent work in neurons has demonstrated that NCLX activity is positively regulated by PKA and negatively regulated by PDEs (Kostic et al, 2018; Kostic et al, 2015). Notably, PDE2A is the only PDE known to localize to the mitochondrial matrix (Abusnina and Lugnier, 2017; Acin-Perez et al, 2011b; Lobo et al, 2020; Zhang et al, 2017). Based on these observations, we hypothesized that PDE2A mediates the delayed NCLX activation we observed in BAT PDM (Fig. 4A). To assess whether PDE2A is enriched in BAT, we first quantified PDE2A expression across mouse tissues. PDE2A levels were significantly higher in BAT mitochondria compared with mitochondria from heart, liver, subcutaneous WAT, and visceral WAT (Fig. 5A). Furthermore, within BAT, PDE2A expression was significantly higher in PDM than in CM (Fig. 5B).

To directly test whether PDE2A regulates mt-Ca²⁺ homeostasis in BAT, we pretreated pBA with the PDE2A-selective inhibitor Bay 60-7550 (BAY60) (Acin-Perez et al, 2011b; Shi et al, 2018; Soares et al, 2017; Wang et al, 2021) and monitored mitochondrial matrix Ca²⁺ dynamics using mt-GCaMP6. In WT pBA, BAY60 markedly accelerated mitochondrial Ca²⁺ *efflux* following NE stimulation, resulting in a pronounced overshoot of matrix Ca²⁺ levels below the pre-stimulation baseline compared with vehicle-treated controls (Fig. 5C,E). BAY60 treatment did not affect the initial Ca²⁺ *influx* induced by NE, indicating a selective effect on Ca²⁺ extrusion rather than uptake.

This synergistic effect of BAY60 and NE, producing a transient undershoot in mitochondrial Ca²⁺, is consistent with prior observations in hippocampal neurons (Rozenfeld et al, 2022). To confirm that the effect of PDE2A inhibition is mediated specifically through NCLX, we repeated these experiments in pBA derived from NCLX^KO^ mice. In the absence of NCLX, BAY60 treatment failed to alter the sustained elevation of mitochondrial Ca²⁺ following NE stimulation (Fig. 5D,E), demonstrating that PDE2A regulates mitochondrial Ca²⁺ dynamics exclusively through NCLX.

Together, these results establish PDE2A as a key regulator of mitochondrial Ca²⁺ extrusion in BAT and identify differential PDE2A–NCLX regulation as a mechanism underlying the distinct Ca²⁺ handling properties of PDM and CM.

### Activation of NCLX by the inhibition of PDE2 affects lipid mobilization by promoting PDM formation

The preceding results indicate that PDM detachment is driven by elevations in mitochondrial matrix Ca²⁺ and that this rise can be amplified through PDE2-mediated suppression of NCLX-dependent Ca²⁺ extrusion. These observations suggested that PDE2 activity may regulate mitochondrial attachment to and detachment from LDs. To test this directly, we performed the RBA in the presence of Ca²⁺ with or without the PDE2A inhibitor BAY60 (Fig. 5F). As expected, Ca²⁺ alone markedly reduced mitochondrial attachment to LDs. Co-treatment with BAY60 partially restored attachment, whereas BAY60 alone showed a non-significant trend toward increased binding (Fig. 5F; Appendix Table S4), consistent with enhanced NCLX-mediated Ca²⁺ extrusion.

To validate these findings in intact cells, we performed super-resolution microscopy in pBA stimulated with NE in the presence or absence of BAY60 (Fig. 5G,H; Appendix Table S2). PDM were quantified as mitochondrial pixels located within 0.3 µm of an LD surface. NE treatment reduced PDM abundance relative to untreated controls, as expected. BAY60 treatment prevented the NE-induced reduction in PDM, preserving mitochondrial–LD association. These results are consistent with PDE2A inhibition enhancing NCLX activity, limiting matrix Ca²⁺ accumulation, and thereby preventing PDM detachment.

Given that PDM promote fatty acid (FA) esterification and triglyceride (TG) synthesis and that their attachment limits lipase access to LDs (Fig. 1A), we next examined the metabolic consequences of PDE2 inhibition. Using high-resolution AiryScan imaging, we analyzed LD size distribution in pBA treated with NE in the presence or absence of BAY60 (Fig. 5I,J; Appendix Table S2). NE alone increased the frequency of small LDs and decreased the frequency of large LDs, consistent with lipolytic remodeling. BAY60 prevented these NE-induced changes in LD size distribution. The preservation of very large LDs in the presence of BAY60 was particularly pronounced (Fig. 5J), indicating suppression of LD breakdown.

To directly assess lipolysis, we measured glycerol release (Fig. 5K) and TG breakdown (Fig. 5L) in NE-stimulated BA treated with BAY60. The PKA inhibitor H89 served as a positive control for lipolysis inhibition. PDE2A inhibition significantly blunted NE-induced glycerol release and TG breakdown, indicating reduced lipolytic activity (Fig. 5K,L).

Adrenergic stimulation in BAT promotes uptake of multiple nutrients, including glucose and fatty acids. Previous studies have shown that increased mitochondrial attachment to LDs is associated with a shift toward pyruvate oxidation (Benador et al, 2018). We therefore hypothesized that preventing PDM detachment with BAY60 would shift substrate utilization from fatty acids toward glucose. To test this, we measured glucose levels in the culture medium of pBA pretreated with BAY60 and stimulated with NE for 6 h (Fig. 5M). BAY60 alone increased glucose uptake to levels comparable to NE treatment. Combined BAY60 and NE treatment further enhanced glucose uptake, indicating that PDE2A inhibition does not suppress NE-induced energy demand but instead promotes a metabolic shift toward glucose utilization.

Collectively, these data demonstrate that PDE2A inhibition prevents NE-induced mitochondrial detachment from LDs, attenuates lipolysis, preserves LD size, reduces glycerol release, and enhances glucose uptake. To determine whether these effects are mediated through NCLX, we repeated lipolysis measurements in BA derived from NCLX^KO^ mice. In the absence of NCLX, BAY60 failed to alter NE-induced glycerol release (Fig. 5K), indicating that the metabolic effects of PDE2A inhibition are dependent on NCLX-mediated Ca²⁺ extrusion.

### PDE2 inhibition in *ob/ob* mice increases PDM content in BAT in vivo

The ability of PDE2A inhibitors to increase PDM levels in cultured cells provided a tool to investigate the consequences of modulating mitochondrial binding to LDs in vivo and to assess the role of PDM in fuel preference and energy expenditure. We first tested whether PDE2A inhibition in vivo increases mitochondria–LD adherence.

We selected the *ob/ob* mouse as our animal model based on previous reports showing that the leptin pathway regulates PDE2A, and that PDE2A is expressed at uniquely high levels in BAT in rodent obesity models (Coudray et al, 1999). As such, *ob/ob* mice offered an opportunity to evaluate the role of PDE2A in mitochondrial attachment to LDs in vivo. Because PDE2A activity is expected to decrease NCLX activity and thereby promote PDM detachment from LDs, we anticipated that BAT from *ob/ob* mice would have lower PDM levels at either RT or TN. Acute 4 °C cold exposure is an extreme condition that leads to almost complete PDM removal from LDs in WT mice and is lethal to *ob/ob* mice; therefore, we avoided acute 4 °C exposure and instead used the milder cold stress of standard laboratory RT, reasoning that RT would provide a better dynamic range for the regulation of mitochondria–LD adherence (Milner and Trayhurn, 1989; Smith and Romsos, 1984).

**Figure 6:**
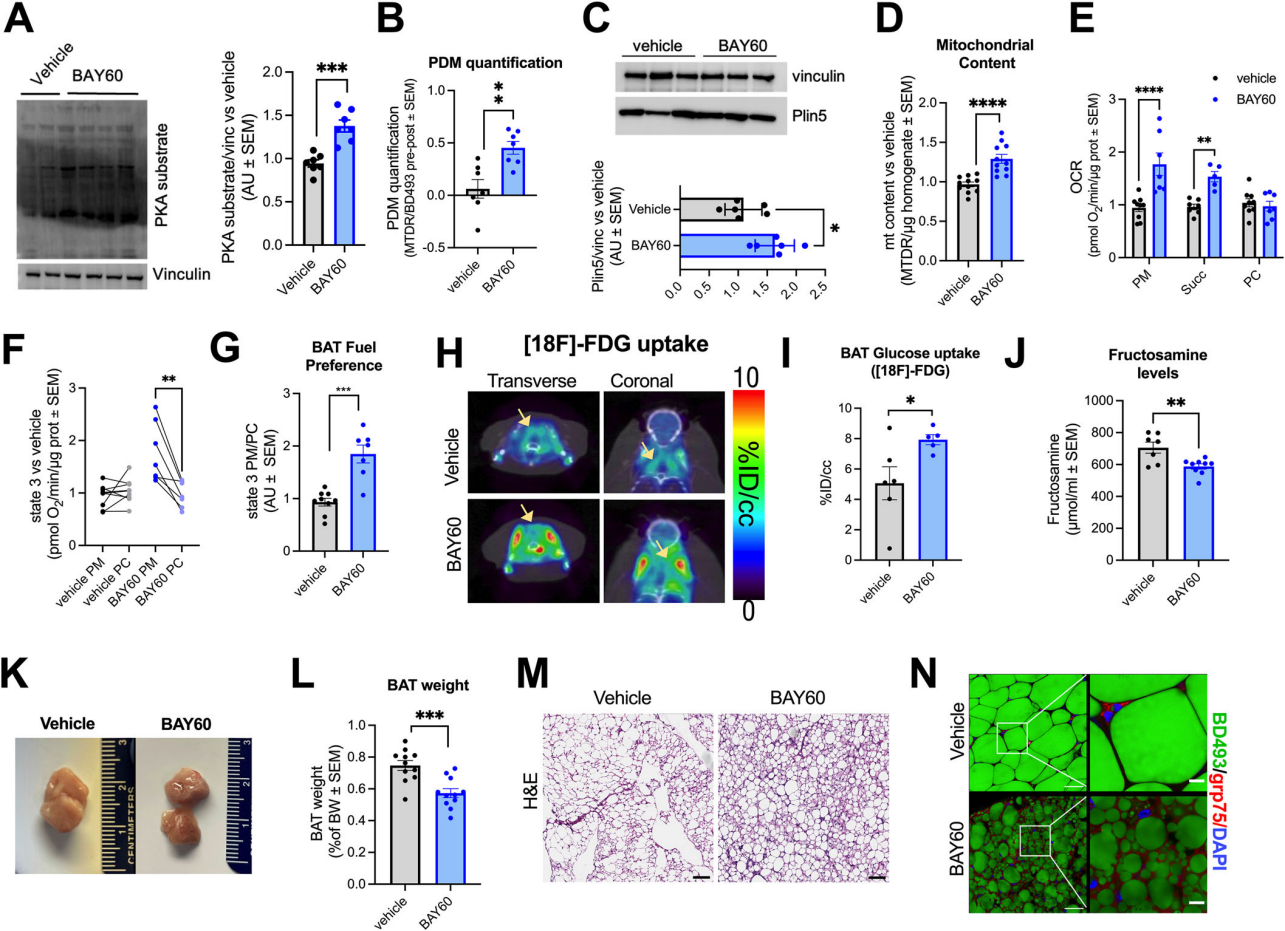
PDE2 inhibition increases PDM abundance and enhances metabolic remodeling in BAT of *ob/ob* mice (see also Fig. EV4). Male *ob/ob* mice were treated with the PDE2A inhibitor BAY 60-7550 (1 mg/kg) for 3 weeks. (**A**) Validation of PDE2A inhibition in vivo. Western blot and quantification of PKA substrate phosphorylation in BAT homogenates from vehicle- and BAY60-treated mice. Data are normalized to vehicle controls. Unpaired *t* test. (**B**) Effect of BAY60 on PDM abundance. PDM content was quantified in the isolated FL and normalized to mechanically stripped FL (sFL). PDM were nearly undetectable in vehicle treated *ob/ob* mice. Unpaired *t* test. (**C**) Perilipin 5 (Plin5) expression in BAT from vehicle- and BAY60-treated mice. Unpaired *t* test. (**D**) Total mitochondrial content in BAT homogenates, quantified by MitoTracker Deep Red (MTDR) staining and normalized to total protein. Unpaired *t* test. (**E**–**G**) Effect of BAY60 on mitochondrial respiration and substrate preference. (**E**) State 3 respiration in BAT homogenates under indicated substrates, normalized to vehicle controls. Two-way ANOVA. (**F**) Pairwise comparison of state 3 respiration with individual substrates. BAY60 increases pyruvate-supported respiration, a characteristic feature of PDM. Paired *t* test. (**G**) Fuel preference index calculated as the ratio of state 3 respiration with pyruvate-malate (PM) to palmitoyl-carnitine/malate (PC) (PM/PC). Unpaired *t* test. (**H**) Representative PET images (transverse and coronal views) showing [18 F]-FDG uptake in BAT of vehicle- and BAY60-treated mice housed at room temperature. Arrows indicate BAT. (**I**) Glucose uptake is enhanced in BAY60-treated mice at RT. Quantification of [18 F]-FDG uptake in BAT. Unpaired *t* test. (**J**) Serum fructosamine levels following 3 weeks of treatment. Unpaired *t* test. (**K**) Representative gross images of BAT from vehicle- and BAY60-treated mice. (**L**) BAT weight normalized to body weight (BW). Unpaired *t* test. (**M**) H&E staining of BAT sections. Scale bar = 100 µm. (**N**) Whole-mount imaging of BAT. BD493 (green) labels lipid droplets, grp75 (red) labels mitochondria, and DAPI (blue) labels nuclei. BAY60 treatment promotes multilocular LDs and increased mitochondrial association. Scale bars = 50 µm (overview), 10 µm (insets). (**A**–**N**) *n* ≥ 4 unless otherwise indicated. Each point represents a biological replicate; technical replicates were averaged. Data are mean ± SEM. *P < 0.05; **P < 0.01; ***P < 0.001; ****P < 0.0001. Source data are available online for this figure.

To test the capacity of PDE2A inhibition to increase mitochondrial adherence to LDs in vivo, *ob/ob* mice were treated with BAY60 or vehicle for 3 weeks. The efficacy of PDE2A inhibition in BAT was validated by measuring PKA activity products, which showed a ~40% increase in phosphorylated PKA substrates in BAT from BAY60-treated mice (Fig. 6A). PDM content was quantified in isolated BAT by determining mitochondrial content in the floating FL (Fig. 6B). Remarkably, PDM levels in the FL of vehicle-treated *ob/ob* mice were barely detectable. To increase sensitivity, we normalized PDM levels to a sFL in which PDM had been removed by centrifugation. Mice treated with BAY60 for 3 weeks showed a marked, >fourfold increase in PDM (Fig. 6B), accompanied by an increase in the LD protein perilipin 5 (Plin5) (Fig. 6C). This increase in PDM was not driven by a global rise in mitochondrial content, as total mitochondrial mass increased by less than 20% on average (Fig. 6D). PDM have previously been shown to exhibit higher complex V (CV) expression, greater pyruvate oxidation capacity, and reduced FA oxidation capacity (Benador et al, 2018). Given the BAY60-induced shift toward increased PDM, we next asked whether BAY60 alters the overall bioenergetic profile of BAT mitochondria. Respiratory analysis demonstrated an increase in state 3 respiration (Fig. 6E). Further analysis revealed a clear shift in fuel preference toward pyruvate utilization, with no change in PC-supported respiration, and this preference was observed in mitochondria from each BAY60-treated mouse (Fig. 6F,G).

To determine whether the mitochondrial fuel shift to pyruvate was accompanied by an increase in glucose uptake in vivo, we measured BAT glucose uptake at RT (Fig. 6H), reasoning that RT would allow a dynamic range of mitochondrial association with LDs and thus reveal fuel preference effects that might be masked under cold exposure. PET imaging of BAT glucose uptake using the Fludeoxyglucose F18 tracer ([18 F]-FDG) showed an >50% increase in glucose uptake (Fig. 6I). This change could reflect a shift toward glucose utilization, as suggested in Fig. 6I, but could also indicate a general increase in energy expenditure (EE).

**Figure EV4:**
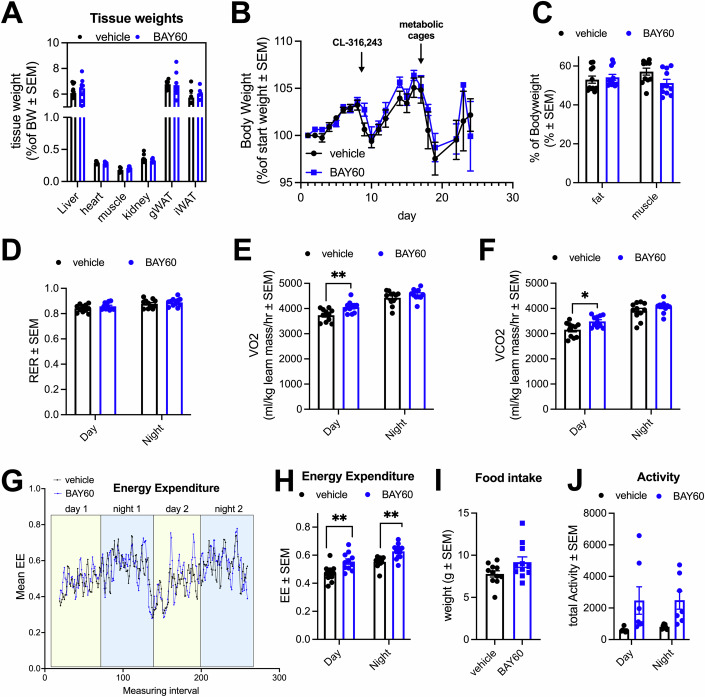
Mitochondrial function upon PDE2 inhibition in *ob/ob* mice. (**A**) Weight measurements of the indicated tissues from *ob/ob* mice treatment with vehicle or BAY60. (**B**) Body weight measurements of *ob/ob* mice treated with vehicle or BAY60. Body weights were normalized to weight at day 0 for each mouse. (**C**) Quantification of lean (muscle) and fat mass measured by NMR after 2 weeks of treatment with vehicle or BAY60. (**D**) Quantification of the RER measurements at both light and dark cycles. Values are calculated as the ratio of VCO_2_ to VO_2_ produced and consumed by the mice, respectively. (**E**) Quantification of VO_2_ normalized to lean mass in *ob/ob* mice treated with vehicle or BAY60. (**F**) Quantification of VCO_2_ normalized to lean mass in *ob/ob* mice treated with vehicle or BAY60. (**G**, **H**) Representative profile of Energy expenditure measurements during the day and night period in *ob/ob* mice treated with vehicle or BAY60 (**G**) and quantification (**H**). (**I**) Food intake measurements in *ob/ob* mice treated with vehicle or BAY60. (**J**) Total activity in *ob/ob* mice treated with vehicle or BAY60 measured in the metabolic cages. (**A**–**J**) (*n* ≥ 6). Measurements were after 2 weeks of treatment with vehicle or BAY60. Each point represents a biological replica sample. For each biological replicate, technical replicates were averaged. Data represent average  ± SEM. *P < 0.05; **P < 0.01. (**E**, **F**) Two-way ANOVA and (**H**) multiple unpaired *t* test. Source data are available online for this figure.

The shift toward glucose utilization raises the possibility that decreased lipid oxidation could constrain total EE. To evaluate EE, mice were weighed daily (Fig. EV4B) and then housed in CLAMS metabolic cages for an additional week. Body weight, food intake, lean and fat mass percentage, and respiratory exchange ratio (RER) did not differ significantly between groups (Fig. EV4C–F). In contrast, EE was significantly increased during both the dark and light phases in BAY60-treated mice (Fig. EV4G), without changes in food intake (Fig. EV4H). In addition to elevated EE, total activity was also increased in BAY60-treated mice (Fig. EV4I).

To determine whether these changes translated into a change in glycemic control, we measured circulating fructosamine in serum as an indicator of average blood glucose over 2–3 weeks (Malmstrom et al, 2014; Monnier, 2006; Riveros-Mckay et al, 2022). In humans with diabetes mellitus, elevated serum fructosamine reflects prolonged hyperglycemia over the preceding 2–3 weeks (Shohat et al, 2021). BAY60-treated mice exhibited lower fructosamine levels than vehicle-treated controls (Fig. 6J), indicating improved glycemic control in *ob/ob* mice treated with BAY60.

Macroscopic examination revealed that BAY60 treatment reduced BAT size and mass and produced a visibly darker tissue appearance (Fig. 6K,L). Histological analysis demonstrated reduced LD size and increased multilocularity (Fig. 6M), and, together with the observed increase in mitochondrial mass (Fig. 6N), these features are consistent with a browning phenotype. No additional macroscopic alterations were detected in other tissues examined (Fig. EV4A), suggesting a BAT-specific effect.

Previous studies have reported an association between PDM abundance and browning in both humans and mouse models (Acin-Perez et al, 2021; Benador et al, 2018; Winn et al, 2019). This raises the possibility that the increase in PDM observed following BAY60 treatment may be linked to the browning phenotype. However, whether PDM induction is causally upstream of browning or occurs as a consequence of tissue remodeling remains to be determined. Nevertheless, the in vitro RBA data (Fig. 5F–H) demonstrate that BAY60 directly enhances mitochondrial binding to LDs, suggesting that increased PDM abundance could potentially be an upstream event in the remodeling observed in vivo.

## Discussion

The isolation and biochemical characterization of LD-associated mitochondria demonstrated that individual cells can maintain distinct mitochondrial subpopulations that coexist side by side within the same cytoplasm, yet exhibit different proteomes and metabolic capacities (Acin-Perez et al, 2021; Benador et al, 2018). CM exhibit higher FA oxidation capacity, whereas PDM preferentially oxidize pyruvate, thereby supporting LD expansion by shifting the balance from lipolysis toward TG synthesis. In BAT, LD expansion is fueled specifically by ATP generated by PDM. Consistent with this concept, it has been recently demonstrated that hepatic PDM support de novo lipogenesis by metabolizing pyruvate to generate citrate (Najt et al, 2023; Talari et al, 2025).

While this metabolic specialization supports lipid storage under basal conditions, thermogenic activation rapidly reverses this balance. NE stimulation induces a pronounced architectural transformation of PDM: from elongated to swollen and fragmented, accompanied by their detachment from LDs (Figs. 2, 4 and 5) (Quiros et al, 2012; Wikstrom et al, 2014). This coordinated morphological and positional shift suggests that mitochondrial detachment is not merely a structural consequence of thermogenesis but represents a regulated step that reorients LD metabolism toward lipolysis.

LDs are enclosed by a phospholipid monolayer enriched with amphipathic proteins such as perilipins (PLINs), which regulate lipase access to the neutral lipid core (Sztalryd and Brasaemle, 2017). Lipid utilization is initiated when lipases gain access to surface “packing defects” (Kory et al, 2016; Kory et al, 2015), often generated by displacement or modification of LD-associated proteins. The observation that NE-induced lipolysis in BAT coincides with robust mitochondrial detachment from LDs raised the possibility that removal of PDM contributes to the formation of such permissive surface domains. In this framework, PDM detachment would not only reduce local esterification at the LD surface but also expose previously shielded membrane regions to lipolytic enzymes. Consistent with this model, mechanical removal of mitochondria from LDs by centrifugation increased the activity of recombinant lipases, supporting the idea that PDM function as both a metabolic and physical barrier to lipid mobilization. Whether the removal of mitochondria expose LDs to other lipolytic mechanisms such as lipophagy remains to be investigated.

These observations prompted us to move beyond correlation and directly interrogate the signals governing mitochondrial attachment to and detachment from LDs.

Given the rapid (minute-scale) kinetics of PDM detachment, we reasoned that the process is regulated by signaling events rather than de novo protein synthesis. To focus specifically on signals acting directly on mitochondria and LDs, we developed a cell-free reconstitution system that allowed systematic testing of candidate metabolites and ions characteristic of the transition from lipid storage to lipid utilization. This approach enabled us to determine whether specific factors are sufficient to drive attachment or detachment in isolation.

This reductionist strategy parallels classic cell-free systems such as those demonstrating microtubule-based motility or ribosomal protein synthesis. Moreover, certain mechanistic questions, such as whether PDM intrinsically exhibit higher LD binding affinity, cannot be resolved reliably in intact cells, where organelle trafficking and cytoskeletal architecture confound interpretation. The cell-free approach therefore provided mechanistic resolution unattainable in whole-cell systems.

A limitation of reconstitution systems is their potentially non-physiological context. We therefore performed extensive validation experiments to define the boundaries of the system. Isolated PDM retained their enhanced LD-binding capacity relative to CM. Moreover, PDM isolated from animals housed at different temperatures preserved temperature-dependent binding properties ex vivo. Particularly informative was the finding that cytosol from animals housed under different thermal conditions differentially regulated mitochondrial adherence, implicating soluble factors in the process. Systematic testing of thermogenesis-associated metabolites and ions identified Ca^2+^, pyruvate, and acyl-carnitines as key modulators of mitochondrial attachment and detachment. Acyl-carnitines are generated by CPT1 during the lipolytic surge induced by NE. Because CM preferentially oxidize acyl-CoA species, activation of CM lipid utilization may itself generate a detachment signal for PDM. Such a mechanism could create a feedforward amplification loop in which CM-driven FA oxidation promotes further lipolysis by inducing PDM detachment.

Mitochondrial pyruvate metabolism produces citrate, which can chelate Ca^2+^. This led us to hypothesize that Ca^2+^, pyruvate, and acyl-carnitines may converge on a common regulatory axis: mitochondrial matrix Ca^2+^. Indeed, our Ca^2+^measurements demonstrated that these fuels modulate free Ca^2+^levels, supporting a unifying mechanism centered on mt-Ca²⁺ handling.

The pronounced mitochondrial fragmentation and swelling observed during lipolytic activation suggested a structural mechanism linking Ca^2+^to detachment. Inducing mitochondrial swelling with the potassium ionophore valinomycin reproduced detachment in the absence of added Ca^2+^, demonstrating that architectural changes alone are sufficient to disrupt LD association.

Ca^2+^-induced swelling may reflect either transient permeability transition or irreversible apoptotic pore opening. The distinction lies in preservation of membrane integrity and respiratory function. Apoptotic permeability transition involves cytochrome c and NADH leakage and irreversible respiratory failure. Our data show that Ca^2+^-induced swelling associated with PDM detachment is reversible and does not compromise respiratory capacity. The rise in matrix Ca^2+^ implicates mitochondrial Ca^2+^ transporters in regulating PDM adherence. Kinetic analyses revealed slower Ca²⁺ extrusion in PDM relative to CM, providing a mechanism for prolonged Ca^2+^ elevation during NE stimulation. NCLX is the principal Ca^2+^ efflux pathway in BAT, and its deletion causes Ca^2+^ overload during acute cold exposure (Assali et al, 2020). This raises a critical question: how is Ca^2+^ elevation kept within a physiological range during thermogenic activation?

Our kinetic data suggest that Ca^2+^ extrusion eventually restores baseline levels within minutes, indicating tight regulation of NCLX activity. NCLX activity is positively regulated by PKA-dependent phosphorylation (Kostic et al, 2018; Kostic et al, 2015) and negatively regulated by PDE2A-mediated cAMP degradation (Rozenfeld et al, 2022). Inhibition of PDE2A accelerated Ca^2+^ extrusion and prevented PDM detachment in NE-stimulated cells. Although lipolysis was attenuated under these conditions, thermogenic EE was preserved, with substrate utilization shifting from fatty acids toward glucose.

In this study, we describe an additional characteristic that differentiates PDM from CM. Ca^2+^ sensitivity assays demonstrated that PDM show a uniquely higher sensitivity to a surge in extra mitochondrial Ca^2+^ concentrations, in part due to reduced NCLX activity mediated by increased PDE2A levels, and in part due to higher CyPD expression levels. This is the first description of a subpopulation of mitochondria within a cell that are uniquely programmed to be the first responders to a Ca^2+^ surge.

Together, these findings support a model in which mitochondrial matrix Ca²⁺ dynamics couple organelle positioning to metabolic flexibility. During thermogenic activation, a transient Ca²⁺ surge promotes CypD-dependent mitochondrial swelling and detachment of PDM from LDs, relieving a lipolytic barrier. Subsequent NCLX-mediated Ca²⁺ extrusion restores mitochondrial integrity and limits permeability transition, ensuring reversibility. By tuning this Ca²⁺ transient through the PDE2A–NCLX axis, cells regulate PDM abundance, substrate preference, and the balance between lipid mobilization and energy expenditure. Targeting this regulatory node may offer new opportunities to reprogram adipose metabolism in obesity and related metabolic disorders (Alcala et al, 2019; Cui and Chen, 2016; Kim and Plutzky, 2016; Srivastava and Veech, 2019; Vargas-Castillo et al, 2017).

*Ob/ob* mice BAT has been reported to show features of whitening, which include larger LDs and lower mitochondrial mass (Kotzbeck et al, 2018; Qian et al, 2013). We find that treatment of *ob/ob* mice with the PDE2A inhibitor resulted in features consistent with re-browning. Previous studies have demonstrated a correlation between PDM abundance and browning in both human BAT and mouse models of diet-induced re-browning (Acin-Perez et al, 2021; Benador et al, 2018; Winn et al, 2019). Our findings extend this association by providing mechanistic evidence that pharmacological inhibition of PDE2A directly promotes mitochondrial attachment to LDs in a cell-free system, independent of systemic remodeling cues. These data support a model in which PDM induction is not merely a downstream consequence of browning but may actively contribute to the establishment of the browning phenotype. By stabilizing mitochondrial–LD interactions, BAY60 may shift substrate utilization and local lipid handling in a manner that promotes mitochondrial expansion and multilocular morphology. While additional studies will be required to definitively establish directionality in vivo, the direct effect of BAY60 on mitochondrial binding argues that modulation of PDM abundance represents an upstream regulatory node in adipose tissue remodeling.

Several limitations of this study warrant consideration. First, although the cell-free reconstitution system provides mechanistic resolution, it cannot fully recapitulate the spatial constraints, cytoskeletal interactions, and organelle crowding present in intact cells. Second, although our data identify matrix Ca²⁺ and the PDE2A–NCLX axis as central regulators of PDM positioning, we cannot exclude contributions from additional post-translational modifications or structural tethering proteins that may cooperate with Ca²⁺ signaling. Third, while the *ob/ob* model enabled interrogation of this pathway in a metabolically challenged context, obesity-associated adaptations may influence mitochondrial behavior independently of PDE2A–NCLX signaling. Finally, loss of NCLX may trigger compensatory responses that limit mitochondrial Ca^2+^ accumulation. Possible mechanisms include increased activity of secondary alternative mitochondrial Ca^2+^ extrusion routes, modulation of ER-mitochondria Ca^2+^ transfer through altered contact site dynamics, and metabolic reprogramming that reduces Ca^2+^-sensitive fluxes. Such adaptations could partially preserve mitochondrial function in NCLX-deficient BAT and warrant targeted investigation.

## Methods

**Table:** Reagents and Tools Table

Reagent/resource	Reference or source	Identifier or catalog number
**Experimental models**		
C57BL/6NJ	Jackson lab	C57BL/6NJ
NCLX^KO^	Jackson lab	C57BL/6NJ-*Slc8b1*^*em1(IMPC)J*^/J
CypD^KO^	Jackson lab	B6;129-Ppif^tm1Jmol^/J
WT littermates CypD^KO^	Jackson lab	B6129SF2/J
**Recombinant DNA**		
mt-GCaMP6	Gift from Diego De Stefani	
**Antibodies**		
TOMM20	Sigma-Aldrich	WH0009804M1
Grp75	Abcam	ab53098
Alexa Fluor 568	Thermo Fisher Scientific	A11004
Alexa Fluor 488	Thermo Fisher Scientific	A21206
DyLight 680 (mouse)	Thermo Fisher Scientific	35518
DyLight 800 (rabbit)	Thermo Fisher Scientific	SA5-10036
HRP anti-rabbit	Cell Signaling Technology	7074S
SDHA	Thermo Fisher Scientific	459200
ATP5A1	Thermo Fisher Scientific	439800
UQCRC2	ProteinTech	14742-1-AP
Plin1	Abcam	ab61682
PKA substrate Ab	Cell Signaling Technology	9621S
CypD	Abcam	ab110324
Plin5	PROGEN Biotechnik	GP44
OPA1	BD Transduction Laboratories	612606
Tom20	Santa Cruz Biotechnology	sc-11415
PDE2A	Santa Cruz Biotechnology	sc-271394
VDAC1	Santa Cruz Biotechnology	sc-8828
vinculin	Sigma-Aldrich	V9131
**Oligonucleotides and other sequence-based reagents**		
Primers	This study	Methods
**Chemicals, enzymes and other reagents**		
Fructosamine Assay Kit	Abbexa	298916
Amplex™ Red Glucose Oxidase Assay Kit	Thermo Fisher Scientific	A22189
Collagenase Type II	WORTHINGTON	LS005176
Rosiglitazone	MedChem Express	HY-17386
Human recombinant Insulin	Sigma-Aldrich	I2643
STEMpro Accutase	Thermo Fisher Scientific	A1110501
Ru360	Sigma-Aldrich	557440
CGP-37157	Sigma-Aldrich	C8874
H-89	Santa Cruz Biotechnology	sc-3537
ATGLstatin	Selleck Chemicals	S7364
Triacsin C	Sigma-Aldrich	T4540
BAY60-7550	Sigma-Aldrich	SML2311
Norepinephrine (NE)	Sigma-Aldrich	AO937
BODIPY 493/503 (BD493)	Thermo Fisher Scientific	D3922
DAPI	Life Technologies	D21490
MitoTracker Deep Red FM (MTDR)	Thermo Fisher Scientific	M22426
Acetone/epon EMbed 812	EM Sciences	EMS #14120
Rhod-2 AM	Thermo Fisher Scientific	R1244
8Br-cAMP	Sigma-Aldrich	B5386
Ruthenium Red	Sigma-Aldrich	00541
NIM811	Med Chem Express	HY-P0025
Calcium Green™-1 dextran	Invitrogen	C3713
Fluo4AM	Invitrogen	F14202
UK-5099	Sigma-Aldrich	504817000
Human pancreatic lipase	Sigma-Aldrich	SRE0028
Free Fatty Acid Kit	Abcam	ab65341
Seahorse XF Base medium	Agilent Technologies	*103680-100*
Oligomycin A	Calbiochem	495455
Etomoxir	Sigma-Aldrich	236020
Antimycin A	Sigma-Aldrich	A8674
Carbonyl cyanide 4-(trifluoromethoxy)phenylhydrazone (FCCP)	Sigma-Aldrich	C2920
Paraformaldehyde	Thermo Fisher Scientific	J61899.AK
Hoechst 33342	Thermo Fisher Scientific	R37165
Valinomycin	Sigma-Aldrich	V0627
4–12% Bis-Tris precast gels	Thermo Fisher Scientific	NP0321
TLC plate	Sigma-Aldrich	60778
Free Glycerol Reagent	Sigma-Aldrich	F6428
BODIPY 558/568 C12	Thermo Fisher Scientific	D3835
Live Orange	Abberior GmbH	LVORANGE-0146-300STNLVORANGE
**Software**		
CLAMS Columbus Instruments	https://colinst.com/lp-clams	
Amide	https://amide.sourceforge.net/	
FIJI/ImageJ	https://fiji.sc/	
AIVIA 10.5.0 image analysis	https://www.aivia-software.com/	
KaleidaGraph	https://www.synergy.com/	
GraphPad Prism 9.01 & 10	https://www.graphpad.com/	
MAXCHELATOR program software	https://somapp.ucdmc.ucdavis.edu/pharmacology/bers/maxchelator/downloads.htm	
**Other**		
Tecnai T12 G2 TWIN TEM	Thermo Fisher Scientific	
TermoFisher Scientific (former FEI) Talos F200C	Thermo Fisher Scientific	
Gatan 794 MultiScan CCD camera	Gatan Inc	
Ceta 16 M CMOS camera	Thermo Fisher Scientific	
Zeiss LSM880 microscope	Leica Microsystems	
Olympus IX73 inverted microscope	Leica Microsystems	
Retiga 600 CCD Camera	Teledyne Vision Solutions	
Fluoroskan Ascent microplate reader	Thermo Fisher Scientific	
Seahorse XFe24	Agilent Technologies	
Seahorse XFe96	Agilent Technologies	
Operetta High-Content Imaging System	PerkinElmer	
xCell SureLock Mini-Cells	Novex	
ChemiDoc Molecular Imager	Bio-Rad Lab	
Genevac EZ-2 Plus Evaporating System	Genevac	
FastWell™ incubation chamber	Grace Biolabs Inc	

### Experimental animals

Experimental procedures conducted on mice were performed in accordance with animal welfare and in compliance with other related ethical regulations. The mice studies were conducted under an approved Institutional Animal Care and Use Committee (IACUC) protocol at the University of California, Los Angeles (UCLA) and Ben-Gurion University. The mice were congenic to the C57BL/6NJ background, fed standard chow diet, and maintained under controlled conditions (housing at 22 °C with a 12:12 h light:dark cycle). In all experiments, mice were age and gender matched. For in vivo experiments, age-matched male and female mice of 10–12 weeks old were used.

WT C57BL/6NJ mice, NCLX^KO^ (C57BL/6NJ-*Slc8b1*^*em1(IMPC)J*^/J), and *ob/ob* mice (B6.Cg-Lep^ob^/J) were purchased from Jackson Laboratories (Jackson Lab, Bar Harbor, ME). Whole-body CypD^KO^ and littermate wild-type (WT) mice were produced from heterozygous breeding pairs derived from a male CypD^KO^ mouse (stock #009071, B6;129-Ppif^tm1Jmol^/J; The Jackson Laboratory) (Baines et al, 2005) and a female WT control mouse (stock #101045, B6129SF2/J; The Jackson Laboratory). All mice were bred in our vivarium. Mice genotyping was performed on earpieces or clipped tails obtained during the weaning of pups. Genotyping was performed following the protocol of Jax Laboratories by real-time polymerase chain reaction, using a commercial vendor (Transnetyx). The following primers were used to genotype the mice:

NCLX forward primer—GGCTCCTGTCTTCCTCTGTG

NCLX reverse primer—GTGTCCATGGGCTTTTGTG.

CypD WT forward primer—CTC TTC TGG GCA AGA ATT GC

CypD Mutant forward primer—GGC TGC TAA AGC GCA TGC TCC

CypD common reverse primer—ATT GTG GTT GGT GAA GTC GCC.

B6.Cg-Lep^ob^/J (ob/ob mice) (Jackson lab, Bar Harbor, ME) mice were used for the isolation of cytoplasmic and peridroplet mitochondria as described previously, with some modifications (Benador et al, 2018; Ngo et al, 2021). All animal procedures were executed according to the Guide for Care and Use of Laboratory Animals of the NIH. Mice were fed standard chow (mouse diet 9 F, PMI Nutrition International, Brentwood, MO), maintained under controlled conditions (19–22 °C and a 12:12 h light-dark cycle), and all procedures were approved by the Animal Subjects Committee of the University of California, Los Angeles.

Brown adipocyte isolation was performed as described (Veliova et al, 2020) using WT C57BL/6J mice, WT C57BL/6NJ mice, and NCLX^KO^ (C57BL/6NJ-*Slc8b1*^*em1(IMPC)J*^/J).

### Primary brown adipocyte isolation

Primary BA (pBA) were generated by differentiating pre-adipocytes isolated from BAT as described in detail previously (Veliova et al, 2020). BAT was harvested from 3 to 4-week-old WT and NCLX^KO^. The tissue was dissected from interscapular, subscapular, and cervical regions, minced, and transferred to a collagenase digestion buffer (2 mg/mL Collagenase Type II in 100 mM HEPES, 120 mM NaCl, 4.8 mM KCl, 1 mM CaCl_2_, 4.5 mM Glucose, 1.5% BSA, pH 7.4) at 37 °C water incubator under constant agitation for 30 min with vortex every 5 min. Digested tissue was homogenized and strained through 100 µm and 40 µm strainers. Cold DMEM was added to tissue digest and centrifuged twice (the last included washing and resuspension in new DMEM at 200×*g* speed for 12 min at 4 °C). Finally, cell pellets (preadipocytes) were re-suspended 5 mL growth medium (DMEM supplemented with 20% newborn calf serum (NCS), 4 mM Glutamine, 10 mM HEPES, 0.1 mg/mL sodium ascorbate, 50 U/mL penicillin, 50 mg/mL streptomycin) and plated in six-well plates (Corning). Cells were incubated in 37 °C 8% CO_2_ incubator. Twenty-four hours after isolation, the cells were washed to remove debris and the medium was replaced. 72 h after isolation the cells were lifted using STEMPro Accutase, counted, and re-plated in differentiation media (growth media supplemented with 1 μM rosiglitazone maleate and 4 nM human recombinant insulin). Cells were differentiated for 7 days, and the medium was changed every other day.

Treatments in pBA were performed using the following compounds at the indicated concentrations: 10 µM Ru360 (57440, Sigma-Aldrich, St. Louis, MO); 10 µM CGP-37157 (Sigma-Aldrich, C8874); palmitate 200 µM (4:1 palmitate:BSA); 10 µM H-89 (Santa Cruz Biotechnology, sc-3537); 50 µM ATGLstatin (Selleck Chemicals, S7364); 5 µM Triacsin C (Sigma-Aldrich, T4540), 10 µM BAY60-7550 (Sigma-Aldrich, SML2311) and 1 μM NE (Sigma-Aldrich, AO937).

### Metabolic cages

Baseline metabolic rates were determined using Oxymax Comprehensive Lab Animal Monitoring System (CLAMS, Columbus Instruments) as previously described (Shum et al, 2021). Mice were housed for 5 days in CLAMS; 3 days for acclimation and 2 days to perform metabolic measurements. To measure brown adipocyte thermogenic capacity 1 mg/kg CL-316,243 was injected subcutaneously. Metabolic rates were measured at 30 °C.

RER was calculated with CLAX software (Columbus Instruments) as VCO_2_/VO_2_. Energy expenditure in cal/min was derived from the Lusk equation: (3.815 + 1.232 × RER) × VO_2_ with VO_2_ in ml/min. Data were normalized to lean mass. Body composition was measured using Echo MRI, using a known mass of canola oil for calibration purposes.

### Positron emission tomography

In vivo μPET/CT imaging was performed at the Crump Institute Preclinical Imaging Technology Center. *Ob/ob* Mice (vehicle and BAY60-treated) were fasted for 6 h prior to intravenous injection via tail vein with 80–85 µCi of [18 F]-FDG, a radioactive glucose analog, as reported previously (Assali et al, 2020). Following a 50 min conscious uptake of [18 F]-FDG, mice were anesthetized with 2% vaporized isoflurane to prepare for imaging. PET (10-min static, energy window 350–650 keV) and CT (voltage 50kVP, current 200 µA, 580 projections, 200 µm resolution) images were acquired on a G8 PET/CT scanner (Sofie Biosciences, Dulles, VA) 1-h post-injection. All PET images were reconstructed using the 3D-OSEM/MAP algorithm (24 subsets and 3 iterations) with random, attenuation, and decay correction. All CT images were reconstructed using a Modified Feldkamp Algorithm. Amide software was used to quantify PET signal intensity in the co-registered PET/CT image.

### Fructosamine measurements

Fructosamine Assay Kit (Abbexa 298916) was used to assess circulating fructosamine levels in serum from ob/ob vehicle and treated mice (20 µl of blood serum) following the manufacturer’s recommendations.

Fructosamine content per ml plasma was calculated using the following equation:

$${{{\rm{Fructosamine}}}} \, ({{{\rm{\mu }}}}{{{\rm{mol}}}}/{{{\rm{ml}}}}) =\frac{{{{\rm{Concentration}}}}\; {{{\rm{of}}}}\; {{{\rm{standard}}}}\times {{{\rm{Volume}}}}\; {{{\rm{of}}}}\; {{{\rm{standard}}}}}{{{{\rm{Volume}}}}\; {{{\rm{of}}}}\; {{{\rm{sample}}}}} \times \frac{{{{\rm{OD}}}}\; {{{\rm{sample}}}}{{-}}{{{\rm{OD}}}}\; {{{\rm{blank}}}}}{{{{\rm{OD}}}}\; {{{\rm{standard}}}}{{{-}}}{{{\rm{OD}}}}\; {{{\rm{blank}}}}}$$

### Glucose oxidase assay

Cultured BAs were incubated with a colorless HBSS media containing 5 mM glucose and 1% FBS. Cells were treated with BAY60 or a vehicle and stimulated with or without NE for 6 h. Media was collected, and glucose concentration was determined using Amplex™ Red Glucose Oxidase Assay Kit (Thermo Fisher A22189) following the manufacturer’s recommendations.

### Histology

Formalin-fixed and paraffin-embedded fat tissues were cut in 4 µm sections and stained with hematoxylin and eosin. Slides were scanned and digitized using an Aperio AT high-throughput scanner with a × 20 air objective and multilocular cell regions were identified by 2–3 independent observers. Four to five 1-mm^2^ regions of every section were analyzed for their average lipid droplet size using the Adiposoft plug-in for FIJI/ImageJ (https://fiji.sc/) filtered for a size range of 1–150 µm.

### Immunofluorescence sample preparation

Whole-mount preparations were generated from small pieces (< 5 mm) of formalin-fixed BAT adipose tissue as described (Acin-Perez et al, 2021). All staining procedures took place at 4 °C in a reaction tube on a rotating rack. Briefly, tissues were blocked in a solution of PBS, 1% Triton X-100, 2% BSA, 5% normal donkey serum, 0.1% sodium azide for 2 h, afterwards the solution was replaced with blocking buffer containing antibody against grp75 for 48 h. Subsequently, tissues were washed three times with PBS, 1% Triton, 30 min per wash, and incubated with a fluorescent conjugated secondary antibody, 1 μg/mL BODIPY 493/503 (BD493; Thermo Fisher Scientific, D3922), and DAPI (Life Technologies, D21490) in blocking buffer for 72–96 h. Following a final wash step, tissues were equilibrated in 70% glycerol and mounted between two #1.5 glass coverslips separated by a 1 mm deep FastWell™ incubation chamber (Grace Biolabs Inc., Bend, OR) for imaging purposes.

### Electron microscopy sample preparation and imaging

Chemically fixed samples were washed with 0.1 M cacodylate buffer and post fixed with 1% osmium tetroxide for 1 h. After, samples were washed with 0.1 M cacodylate buffer and incubated with 2% uranyl acetate in DDW for 1 h. Samples were subsequently dehydrated in a sequence of ethanol (30%, 50%, 70%, 90%, and 100% ethanol v/v), pure acetone and acetone/epon EMbed 812 (EM Sciences, EMS#14120). Afterwards, samples were embedded in fresh resin and polymerized in a mold for 48 h at 60 °C. Ultra-thin sections were prepared with an ultra-microtome (UC6, Leica), collected on copper grids and post-stained with 1% uranyl acetate. Ultra-thin sections were imaged with a Tecnai T12 G2 TWIN TEM (FEI) at 120 kV and Thermo Fisher Scientific (former FEI) Talos F200C at 200 kV. The images were taken with a Gatan 794 MultiScan CCD camera and Ceta 16 M CMOS camera, respectively.

### Peridroplet mitochondria and fat layer imaging

Imaging of fat layer was done using a Zeiss LSM880 microscope. Samples were stained with MitoTracker Deep Red FM (MTDR; Thermo Fisher Scientific, M22426) and BD493, subjected to the different treatments and embedded in 1% agarose.

### Fluorescence microscopy and image analysis

Whole Mount tissues were imaged using a Zeiss LSM 880 confocal microscope with Airyscan super-resolution detector using a ×40 Apochromat oil objective.

Super resolution live cell imaging and fat layer imaging were performed on a Zeiss LSM880 using a ×63 Plan-Apochromat oil-immersion lens and AiryScan super-resolution detector with humidified 5% CO_2_ chamber on a temperature-controlled stage (37 °C).

### Primary brown adipocyte imaging

#### Live imaging

Cells were differentiated in glass-bottom confocal plates (Greiner Bio-One, Kremsmünster, Austria). On day 7 of differentiation, cells were incubated with 1 µM BD493 and 400 nM MTDR or 100 nM Live Orange for 0.5–1 h. Dyes were removed before imaging and cells were imaged in regular culture media. BD493 was excited with 488 nm laser, MTDR was excited with 633 nm laser and Live Orange with 561-nm laser.

#### Imaging in fixed cells

Confocal microscopy was performed on fixed cells in glass-bottom dishes (MatTek, Ashland, MA) using the Zeiss LSM880 with a ×40 Plan-Apochromat oil-immersion lens at ×2.7 zoom and AiryScan super-resolution detector. Twenty slices at an interval of 1.06–1.2 microns were obtained to generate a 3D reconstruction of mitochondrial and lipid droplet structures. Mitochondria of primary brown adipocytes were stained with 1:200 primary antibody of TOMM20 (Sigma-Aldrich, WH0009804M1) at 4 °C overnight. The next day, cells were washed in PBS and incubated with 1:500 Anti-Mouse Alexa Fluor 568 antibodies (Thermo Fisher Scientifics, A11004) for 1 h at room temperature, and samples were kept in PBS. Lipid droplets were stained with 1 µM BODIPY 493/503 (BD493) for 45 min prior to imaging. BD493 was excited with 488 nm laser and TOM20 was excited with 561 nm laser.

### Image analysis

#### 3D analysis of mitochondrial and lipid droplet morphology

Between 40 and 60 cells per condition were collected for primary brown adipocytes. The cells were individualized as areas of interest using FIJI ImageJ software. Mitochondria and lipid droplets within areas of interest were individualized through the AIVIA 10.5.0 image analysis software (https://www.aivia-software.com/). Segmentation of mitochondria, lipid droplet, and background was performed by training a 3D pixel classifier in each of the 20 z-planes per image of a single cell. Lipid droplets were identified by training the classifier to BD493 images and mitochondria to TOM20 images. After training the 3D classifier to 10 randomized images, background was minimized by utilizing confidence segmentation. The trained classifier was further optimized by subjecting identified mitochondrial and lipid droplet objects to smart segmentation. On a scale of 1–10 (10 yielding greatest object partitioning) mitochondria were set at a detection of 5, partition of 7, and size range of 2–10; lipid droplets were set at a detection of 6, partition of 7, and size range of 2–10. Identified objects were then subjected to automated mitochondrial and lipid droplet surface area, volume, sphericity, and aspect ratio (the proportional relationship between width and height) measurement. Data are presented as symbols that represent the frequency distribution of lipid droplet volume size per cell. Mitochondrial swelling is expected to increase surface area and decrease the aspect ratio. Representative images shown were adjusted in brightness and contrast for better visualization.

#### Fluorescent Ca^2+^ imaging

Kinetic live-cell fluorescent imaging was performed to monitor Ca^2+^ transients using an IX73 inverted microscope (Olympus) equipped with pE-4000 LED light source and Retiga 600 CCD Camera. Images were acquired through a 20×/0.5 Zeiss Epiplan Neofluar objective using Olympus cellSens Dimension software.

Ca^2+^ imaging was performed in BA that were grown and attached onto coverslips, mounted in a chamber that allowed perfusion of cells and superfused with a Krebs-Ringer’s solution containing (in mM): 123 NaCl, 5.4 KCl, 0.8 MgCl_2_, 20 HEPES, 1.8 CaCl_2_, 15 D-Glucose, 2 Glutamine and 1% free-fatty acid Bovine Serum Albumin (BSA); pH was adjusted to 7.4 with NaOH. cells were washed and then loaded with Rhod-2 AM (1 μM) (Thermo Fisher, R1244) for 30 min at 37 °C. After loading cells were washed again three times followed by additional incubation of 30 min to allow for the de-esterification of the dye. mt-GCaMP6 was excited at 490 nm (Ca^2+^-sensitive wavelength) and fluorescence was collected through a 535 nm band-pass filter.

Mitochondrial Ca^2+^ response was triggered by switching the perfusion solution to Ca^2+^-free Ringer’s solution supplemented by NE (1.5 μM). In some experiments, cells were pretreated with BAY60-7550 at 10 μM. Background fluorescence subtraction was applied for all responses to eliminate noise. Traces of Ca^2+^ responses were analyzed and plotted using KaleidaGraph. The rate of ion transport was calculated from each graph (summarizing an individual experiment) by a linear fit of the change in the fluorescence (ΔF) for Ca^2+^ influx and efflux over time (ΔF/dt). Rates from n experiments (as mentioned in legends to the figures) were averaged, normalized to baseline and displayed in bar graph. Area under the curve (AUC) for NE-stimulated responses was calculated using GraphPad Prism 10.

#### Image analysis of LDs and PDM in live cells and fat layers

Mitochondria adjacent to LD analysis was performed in AIVIA (AIVIA, Leica Microsystems). Mitochondria smaller than 10 pixels in area were not included in final analyses. Mitochondrial area within 0.3 μm of lipid droplet edge were defined as “Mitochondria adjacent to LD” (PDM) while mitochondrial area beyond 0.3-μm peridroplet region were defined as CM. Calculation of “Mitochondria adjacent to LD” is the ratio PDM over Total Mitochondria area (CM area + PDM area).

### Peridroplet mitochondria isolation and quantification

All procedures were performed using pre-chilled equipment and solutions. Mitochondria (CM and PDM) were isolated as previously described (Acin-Perez et al, 2021; Benador et al, 2018; Ngo et al, 2021).

Quantification of PDM in the Fat cake (fat layer containing fat and mitochondria generated in the low-speed centrifugation step) and stripped fat cake (fat layer containing fat and no mitochondria) was performed as described (Acin-Perez et al, 2021).

The reconstituted in vitro assay to assess LD and PDM interaction was based on PDM quantification assay with modifications. Fat layer, stripped fat layer and mitochondria were stained with MTDR + BD493, BD493 or MTDR, respectively. The dyes were washed by centrifugation and the different fractions were combined in the reconstituted in vitro assay. For detachment assays, fat layer was incubated in the presence of the different compounds and/or reagents in either MAS (115 mM KCl, 10 mM KH_2_PO_4_, 2 mM MgCl_2_, 5 mM HEPES, 1 mM EGTA, 0.1% BSA), MAS plus substrates or cytosolic fraction. For attachment experiments, stripped fat layer was incubated with either CM or PDM in the presence of the different compounds and/or reagents in either MAS, MAS plus substrates or cytosolic fraction. Cytosolic fraction, non-containing mitochondria, was used to maximize attachment/detachment since it contains other soluble potential modulators of the LD-mitochondrial interaction. Incubations were performed at RT for 10 min. Samples were spun at 1000× *g* for 10 min and the fat layers were collected. Fluorescence was measured as described (Acin-Perez et al, 2021) to determine the amount of mitochondria bound to LD. All the reconstituted in vitro assays were performed at room temperature (RT). The staining was conducted on the unstripped fat layer (FL), stripped fat layer (sFL), and mitochondria, as indicated in Fig. 1A,B prior to any treatment. Only after staining, the samples were split to assess the effects of different substrates or calcium, and fluorescence was measured using a plate reader. Dye loading is always done before any treatment.

Modulators of attachment and detachment were added at the following final concentrations: pyruvate and malate (5 mM each); palmitoyl-carnitine (40 µM); BAY60 (10 µM); 8Br-cAMP (1 mM) (Sigma-Aldrich, B5386), Ca^2+^ (10 µM); Ruthenium Red (RR, 10 µM) (Sigma-Aldrich, 00541); NIM811 (10 µM) (Med Chem Express, HY-P0025).

Because the buffer contains EGTA, using the MAXCHELATOR program software, we corrected for the EGTA concentration (as well as pH and temperature) to adequately calculate and determine the final *free calcium* concentration stated in the text.

### Calcium retention assays in isolated CM and PDM

Isolated mitochondria were added to a Ca^2+^-free, respiratory buffer containing 10 mM succinate, 10 mM glutamate, 0.5 mM malate, 4 mM ADP and 10 µM Calcium Green (Invitrogen, C3713). A 50 µM CaCl_2_ bolus was added to the solution and mitochondrial Ca^2+^ retention capacity was monitored until it reached plateau. In parallel, 1 µM Ru360 was added to inhibit MCU, and mitochondrial Ca^2+^ retention capacity was blocked, confirming the specificity of the assay. Samples were excited using a 485 nm filter and emission was recorded using a 515 nm filter in a Fluoroskan Ascent microplate reader (Thermo Fisher Scientific).

### Measurements of intramitochondrial calcium concentration in isolated CM and PDM

Isolated mitochondria were loaded with membrane permeant Ca^2+^fluorophone Fluo4AM (10 µM final concentration) (Invitrogen, F14202) and incubated at RT for 20 min. The dye was washed off and mitochondrial were resuspended in respiratory buffer containing the different substrates. A 100 µM CaCl_2_ bolus was added sequentially to the solution and Fluo4AM fluorescence was monitored. The following substrate concentrations were used: pyruvate, malate and glutamate (5 mM each); Palmitoyl-carnitine (40 µM); ADP (4 mM) and UK-5099 (10 µM) (Sigma-Aldrich, 504817000). Samples were excited using a 485 nm filter and emission was recorded using a 515 nm filter in a Fluoroskan Ascent microplate reader (Thermo Fisher Scientific).

### In vitro lipolysis assay in fat layer and stripped fat layer

Supernatant samples were collected from reconstituted in vitro assays performed at room temperature (RT). The assay was conducted on unstripped fat layer (FL), stripped fat layer (sFL), and their subsequent treatments at 37 °C. In some groups, reconstituted samples were treated with human pancreatic lipase (Sigma-Aldrich, SRE0028) at 1:1000 of 1 mg/mL for 20 min at 37 °C. Human pancreatic lipase was reconstituted to 1/mg/ml with tris-buffered saline containing 1% BSA and 1 mM calcium chloride, pH 7.7. Fatty acid release was measured by the Free Fatty Acid Kit (Abcam, ab65341). Samples loaded in a 96-well plate as duplicates were read in a Tecan Spark 10 M multimode plate reader at a fluorescence of EX/EM = 535 nm/587 nm. Background fluorescence was subtracted to eliminate noise. Static lipolysis rates from n experiments (as mentioned in legends to the figures) were averaged and displayed in bar graph. Significance was calculated using GraphPad Prism 10.

### Respirometry in primary brown adipocytes

Respirometry in primary brown adipocytes were performed as previously described (Veliova et al, 2020). Pre-treatments with BAY60-7550 at 10 µM were performed in brown adipocyte culture media. Prior to respirometry measurements, culture media was replaced with respirometry media (Seahorse XF Base medium; Agilent Technologies, Santa Clara, CA, 103680-100) supplemented with the indicated amount of glucose and 3 mM glutamine and incubated for 30–45 min at 37 °C (without CO2). Oxygen consumption rates were measured using the Seahorse XFe24 extracellular flux analyzer (Agilent Technologies). The following compounds were used for injections during the assay: 1 µM norepinephrine (Levophed), 4 µM oligomycin A (Calbiochem, 495455), 10 µM etomoxir (Sigma-Aldrich, St. Louis, MO, 236020), 4 µM antimycin A (Sigma-Aldrich, A8674), 2 µM Carbonyl cyanide 4-(trifluoromethoxy) phenylhydrazone (FCCP; Sigma-Aldrich, C2920). After the assay, cells were fixed using 4% paraformaldehyde (Thermo Fisher Scientific, J61899.AK). To normalize data to cell number, fixed cells were stained with 1 µg/mL Hoechst 33342 (Thermo Fisher Scientific R37165) and nuclei were counted using the Operetta High-Content Imaging System (PerkinElmer).

### Respirometry in isolated mitochondria and homogenates

Freshly prepared mitochondria and homogenates were loaded into a Seahorse XF96 microplate in 20 μL of MAS containing substrates and respiration was assessed as described (Acin-Perez et al, 2021). To assess Ca^2+^ dependent sensitivity in CM and PDM, mitochondria were seeded in the SH plate in the presence of substrates and compounds (NIM811, RR). After centrifugation, MAS containing Ca^2+^ and the different concentrations was added and respirometry was performed.

### Mitochondrial content quantification

Mitochondrial content was assessed in tissue homogenates using the mitochondrial dye MTDR as previously described (Acin-Perez et al, 2020).

### Mitochondrial swelling assay

Swelling assays were modified from previously described protocols (Bhosale and Duchen, 2019; Li et al, 2018; Wong et al, 2012). Isolated CM and PDM from BAT were loaded into a transparent, flat-bottom 96-well plate at a concentration of 25 µg per well. Mitochondria were loaded in 1-2 µL total volume. A volume of 90 µL KCl buffer (125 mM KCl, 2 mM K_2_HPO_4_, 1 mM MgCl_2_, 20 mM HEPES; pH to 7.4) in the presence of either glutamate plus malate (5 mM each) or palmitoyl-carnitine plus malate (40 µM and 5 mM, respectively). To trigger swelling, final concentration of 500 µM of Ca^2+^ or 200 nM of Valinomycin (Sigma-Aldrich, V0627) were added to each well for the final data set. No more than two columns (16 wells) were loaded at one time. The plate was immediately read in a Tecan Spark 10 M multimode plate reader at an absorbance of 540 nm over 14 min. Mitochondrial swelling was measured as the ΔAbs540nm by subtracting the first absorbance time point from the 14-min time-point.

### Protein gel electrophoresis and immunoblotting

#### SDS-PAGE

Cells and tissue lysates (20–30 μg) and mitochondria (10–20 μg) were loaded into 4–12% Bis-Tris precast gels (Thermo Fisher Scientific, NP0321), and gel electrophoresis was performed in xCell SureLock Mini-Cells (Novex) under constant voltage of 80 V for 15 min (to clear stacking) and 120 V for 60 min.

#### Immunoblotting

Proteins were transferred to methanol-activated PVDF membrane in xCell SureLock Mini-Cells under 30 V constant voltage for 1 h at 4 °C. Blots were blocked with 3% BSA in PBST (1 mL/L Tween-20/PBS) and incubated with primary antibody diluted in 1% BSA/PBST overnight at 4 °C. The next day, blots were washed 3 ×10 min in PBST, probed with Alexa Fluor 488 (Thermo Fisher Scientific A21206), DyLight 800 anti-rabbit (Thermo Fisher Scientific, SA5-10036), DyLight 680 anti-mouse (Thermo Fisher Scientific, 35518) or HRP anti-rabbit conjugated (Cell Signaling Technology, 7074S) secondary antibodies diluted in blocking solution for 1 h at room temperature, and rinsed again 3 ×10 min in PBST. Detection was achieved using ChemiDoc Molecular Imager (Bio-Rad). Band densitometry was quantified using ImageJ Gel Plugin (NIH). We used the following antibodies: SDHA (Thermo Fisher Scientific, 459200); ATP5A1 (Thermo Fisher Scientific, 439800); UQCRC2 (ProteinTech, 14742-1-AP); Plin1 (Abcam, ab61682); PKA substrate Ab (Cell Signaling Technology, 9621S); CypD (Abcam, ab110324); Plin5 (PROGEN Biotechnik, GP44); OPA1 (BD Transduction Laboratories, 612606); Tom20 (Santa Cruz Biotechnology, sc-11415); grp75 (Abcam, ab53098); PDE2A (Santa Cruz Biotechnology, sc-271394); VDAC1 (Santa Cruz Biotechnology, sc-8828); and vinculin (Sigma-Aldrich, V9131).

### Total glycerol assessment

Primary brown adipocytes were differentiated for 7 days. For NE treatment, cells were pre-treated with indicated compounds for 30 min following addition of 1 µM NE for 6 h. Total glycerol released into the media was measured using Free Glycerol Reagent (Sigma-Aldrich, F6428) according to manufacturer’s instructions.

### Thin-layer chromatography

Primary brown adipocytes were differentiated for 7 days. Cells were incubated with 1 µM BODIPY 558/568 C12 (BODIPY C12; Thermo Fisher Scientific, D3835) overnight. Then BODIPY C12 was washed out and cells were incubated with the different compounds. For NE treatment, cells were pre-treated with indicated compounds for 30 min following addition of 1 µM NE for 6 h.

Intra-cellular lipids were extracted in 500 µL chloroform. Chloroform was evaporated using the Genevac EZ-2 Plus Evaporating System (Genevac). Lipids were then dissolved in 15 µL chloroform and 1 µL was spotted on a TLC plate (Silica gel on TLC Al foils, Sigma-Aldrich, 60778). Lipids were resolved based on polarity in a developer solution containing ethylacetate and cyclohexane in a 2.5:1 ratio. TLC plates were imaged on the ChemiDoc MP imaging system (Bio-Rad Lab). Band densitometry was quantified using FIJI (ImageJ, NIH).

### Statistics

Unless otherwise mentioned in the figure legends, statistical analysis was performed with GraphPad Prism® 9.01 using one-way or two-way analysis of variance (ANOVA). Corrections for multiple comparisons were made by Tukey post-hoc test when appropriate. Differences were considered statistically different at *P* < 0.05. Individual points in a graph denote individual experiments or biological replicates.

## Supplementary information

Appendix

Peer Review File

Source data Fig. 1

Source data Fig. 2

Source data Fig. 3

Source data Fig. 4

Source data Fig. 5

Source data Fig. 6

Figure EV1 Source Data

Figure EV2 Source Data

Figure EV3 Source Data

Figure EV4 Source Data

Expanded View Figures

## Data Availability

Raw data corresponding to PET-CT images can be found at https://zenodo.org/records/19922569. The source data of this paper are collected in the following database record: biostudies:S-SCDT-10_1038-S44318-026-00827-8.
