# Supplementary material for: Mitochondrial calcium regulates lipid metabolism by modulating tethering of mitochondria to lipid droplets
Source: EMBO J. 2026 Jul 3;45(14):4820–48. doi: 10.1038/s44318-026-00827-8 (PMC13373242; doi:10.1038/s44318-026-00827-8)
Supplement: Supplementary file 1 — Appendix [file 44318_2026_827_MOESM1_ESM.pdf]

Appendix for Mitochondrial Calcium Regulates Lipid Metabolism By Modulating Tethering of Mitochondria to Lipid Droplets

Table of contents

Appendix Table S1. Summary of RBA and RDA experiments presented in Figure 1, 2 and EV1 (page 2).

Appendix Table S2. Summary of imaging experiments in pBA from Figures 2, 4, 5 and EV2 (page 3).

Appendix Table S3. Summary of RBA and RDA experiments presented in Figure 3 (page 4).

Appendix Table S4. Summary of RBA and RDA experiments presented in Figure 4 and 5 (page 4).

Appendix Table S1. Summary of RBA and RDA experiments presented in Figure 1, 2 and EV1.

Panel	Manipulated Parameter	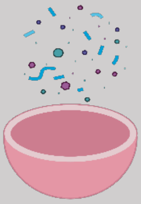 Cytosol Source	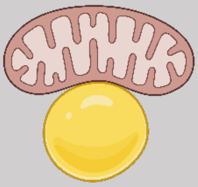 Mitochondrial Source	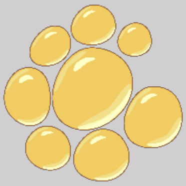 Fat Layer Source	Purpose
**Figure 1D (RBA)**	Mitochondrial source and incubation media	MAS buffer vs BAT – RT cytosol	CM and PDM RT BAT	sFL: RT BAT	To compare the binding capacity of CM and PDMs in different incubation media.
**Figure 1E (RBA)**	Fat Layer Source	RT BAT	PDM: RT BAT	sFL: RT BAT vs WAT	To determine the characteristics of BAT and WAT fat layers in promoting mitochondrial adherence.
**Figure 1F (RBA)**	PDM source	RT BAT	PDM: RT BAT vs RT WAT	sFL: RT BAT	To investigate the binding capacity of BAT vs WAT PDMs to BAT fat layer.
**Figure 1G (RBA)**	BAT PDM	BAT - RT	PDM: 4C vs RT vs TN BAT	sFL: RT BAT	To compare the binding capacity of PDMs from cold-stimulated vs. RT-housed mice, controlling for cytosol and fat layer.
**Figure 1H (RBA)**	BAT Cytosol	4C vs RT vs TN BAT	PDM: RT BAT	sFL: RT BAT	To investigate the role of cytosol in mitochondria-LD tethering, controlling for PDM and fat layer sources.
**Figure 1I (RBA)**	Mitochondrial source and mitochondrial fuels	MAS buffer	CM and PDM RT BAT	sFL: RT BAT	To investigate the role of different fuels in promoting mitochondrial attachment to LD.
**Figure 1J (RBA)**	Calcium entry in mitochondria	RT BAT	PDM: RT BAT	sFL: RT BAT	To investigate the role of calcium accumulation in the mitochondrial matrix in preventing PDM attachment to LD.
**Figure 2A (RDA)**	Calcium entry in mitochondria	RT BAT	PDM: RT BAT	FL: RT BAT	To investigate the role of calcium accumulation in the mitochondrial matrix in promoting PDM detachment from LD.
**Figure EV1A (RBA)**	Fat layer coating protein	MAS buffer	RT BAT	sFL: RT BAT	To demonstrate the role of LD-coating proteins in PDM attachment to LD.
**Figure EV1C (RBA)**	Cytosol source	RT BAT vs RT WAT	PDM: RT BAT	sFL: RT BAT	To investigate the role of BAT vs WAT cytosols in promoting PDM attachment to LD.

Appendix Table S2. Summary of imaging experiments in pBA from Figures 2, 4, 5 and EV2.

Panel	Manipulated Parameter	Purpose
**Figure 2F-H and Figure EV2A-B**	Control NE stimulation	To establish the conditions to compare the different treatments for lipolysis (LD average size) and PDM levels upon basal and NE conditions
**Figure 2F-H and Figure EV2A-B**	Calcium entry in mitochondria	To determine the contribution of intramitochondrial calcium to LD size and PDM levels when MCU is blocked with Ru360.
**Figure 2F-H and Figure EV2A-B**	Use of different fuels by pBA	To investigate the contribution of increasing the source of FA with palmitate to basal and NE response in lipolysis and PDM amount
**Figure 2F-H and Figure EV2A-B**	Amount of fatty acid release	To block the release of FA using an ATGL inhibitor (ATGLstatin) and study the contribution to PDM levels when lipolysis is blocked.
**Figure 4L-M**	NCLX activity (inhibition)	To investigate the role NCLX by blocking its activity with CGP in pBA response to NE
**Figure 5G-J**	NCLX activity (activation)	To investigate the role NCLX by modulating its activity (promoting NCLX phosphorylation with BAY60) in pBA response to NE
**Figure EV3C-E**	Calcium entry in mitochondria and lipolysis	Testing the effect of intramitochondrial calcium and lipogenesis inhibition in LD size distribution upon NE stimulation

Appendix Table S3. Summary of RBA and RDA experiments presented in Figure 3.

Panel	Manipulated Parameter	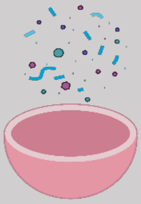 Cytosol Source	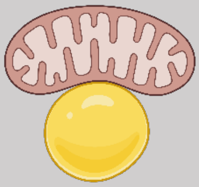 Mitochondrial Source	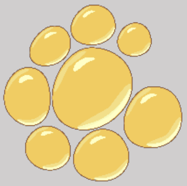 Fat Layer Source	Purpose
**Figure 3C (RDA)**	Mitochondrial calcium and mt-PTP activity	BAT RT cytosol	BAT PDM RT	BAT FL RT	To investigate if calcium mediated detachment is mt-PTP dependent.
**Figure 3E (RDA)**	Different concentrations of Mitochondrial calcium	BAT RT cytosol	BAT PDM RT	BAT FL RT	To compare differences in calcium mediated PDM detachment to mechanically stripped PDM.

Appendix Table S4. Summary of RBA and RDA experiments presented in Figure 4 and 5.

Panel	Manipulated Parameter	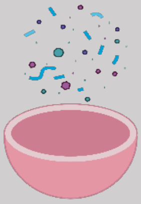 Cytosol Source	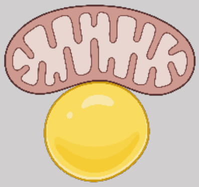 Mitochondrial Source	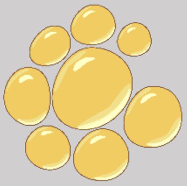 Fat Layer Source	Purpose
**Figure 4J (RBA)**	PDM from WT vs $NCLX^{KO}$ mice at RT and 4°C	WT RT cytosol	WT or $NCLX^{KO}$ at RT or 4°C	BAT sFL RT	To compare PDM attachment to LD depending on genotypes and temperature.
**Figure 4K (RDA)**	Mitochondrial calcium and NCLX activity	BAT RT cytosol	BAT PDM RT	BAT FL RT	To investigate the role of NCLX in detachment by its pharmacological inhibition with CGP.
**Figure 5F (RBA)**	Mitochondrial calcium and NCLX activity	BAT RT cytosol	BAT PDM RT	BAT FL RT	To investigate the role of NCLX-PDE2 in attachment by its activation with BAY60.
