# Supplementary figures and images for: Mitochondrial calcium regulates lipid metabolism by modulating tethering of mitochondria to lipid droplets
Source: EMBO J. 2026 Jul 3;45(14):4820–48. doi: 10.1038/s44318-026-00827-8 (PMC13373242; doi:10.1038/s44318-026-00827-8)

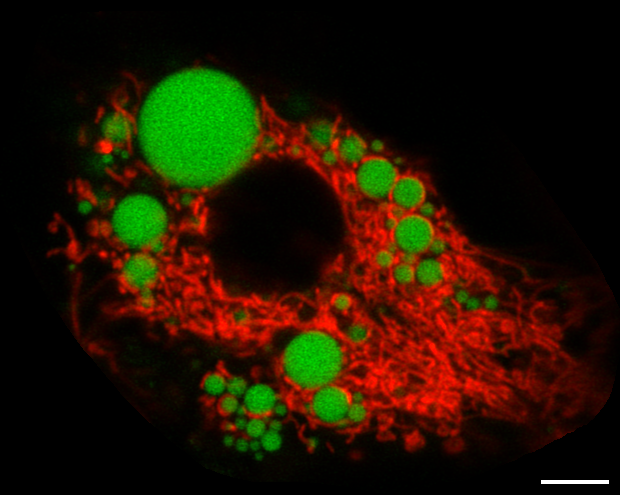

Supplement: Supplementary file 4 — Source data Fig. 2 [file 44318_2026_827_MOESM4_ESM.zip › Figure 2/Figure 2F/RR Image 66 0617 RR.png]

## Slide 1
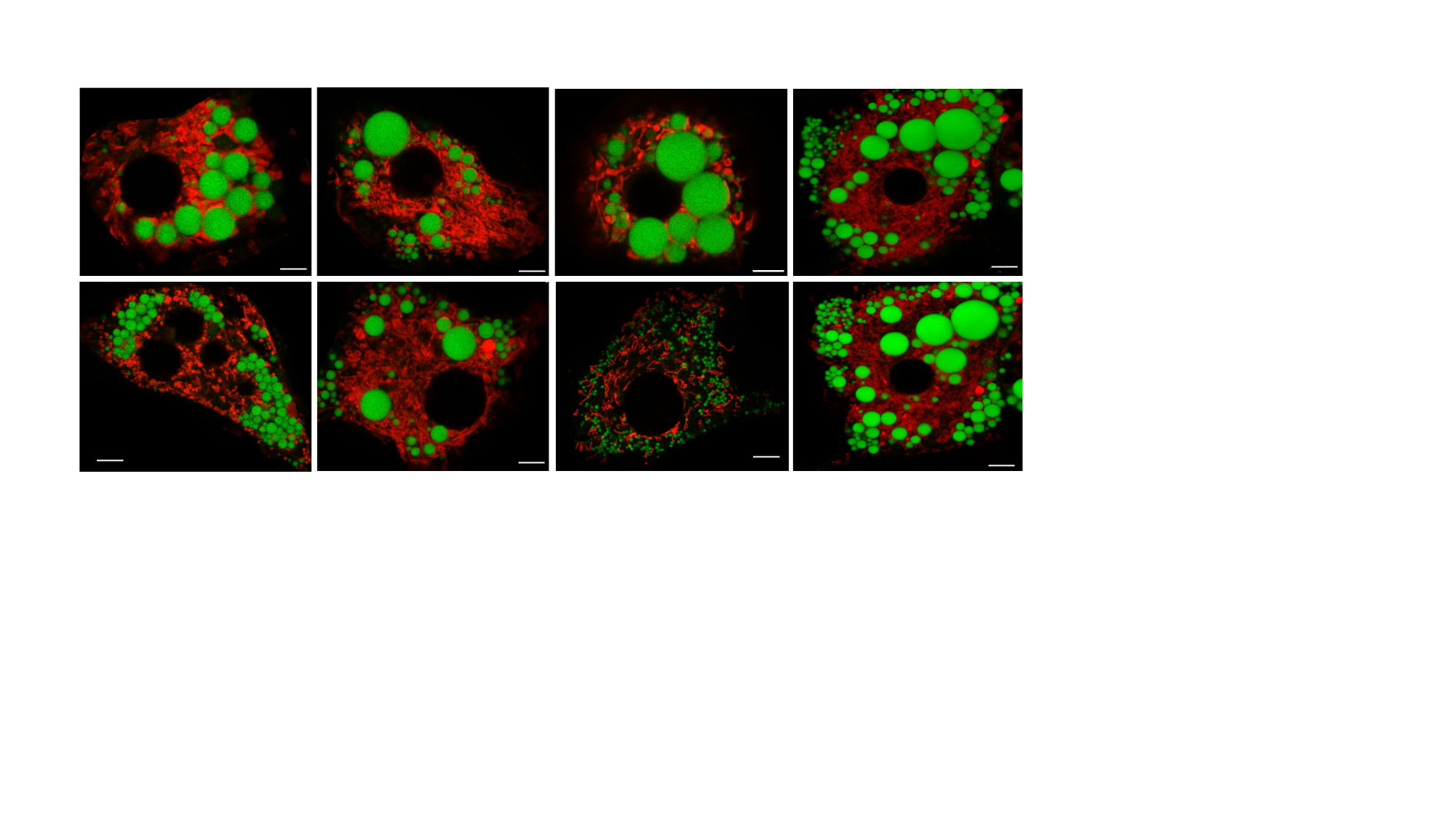

Supplement: Supplementary file 4 — Source data Fig. 2 [file 44318_2026_827_MOESM4_ESM.zip › Figure 2/Figure 2F/Figure 2F image composite.pptx]

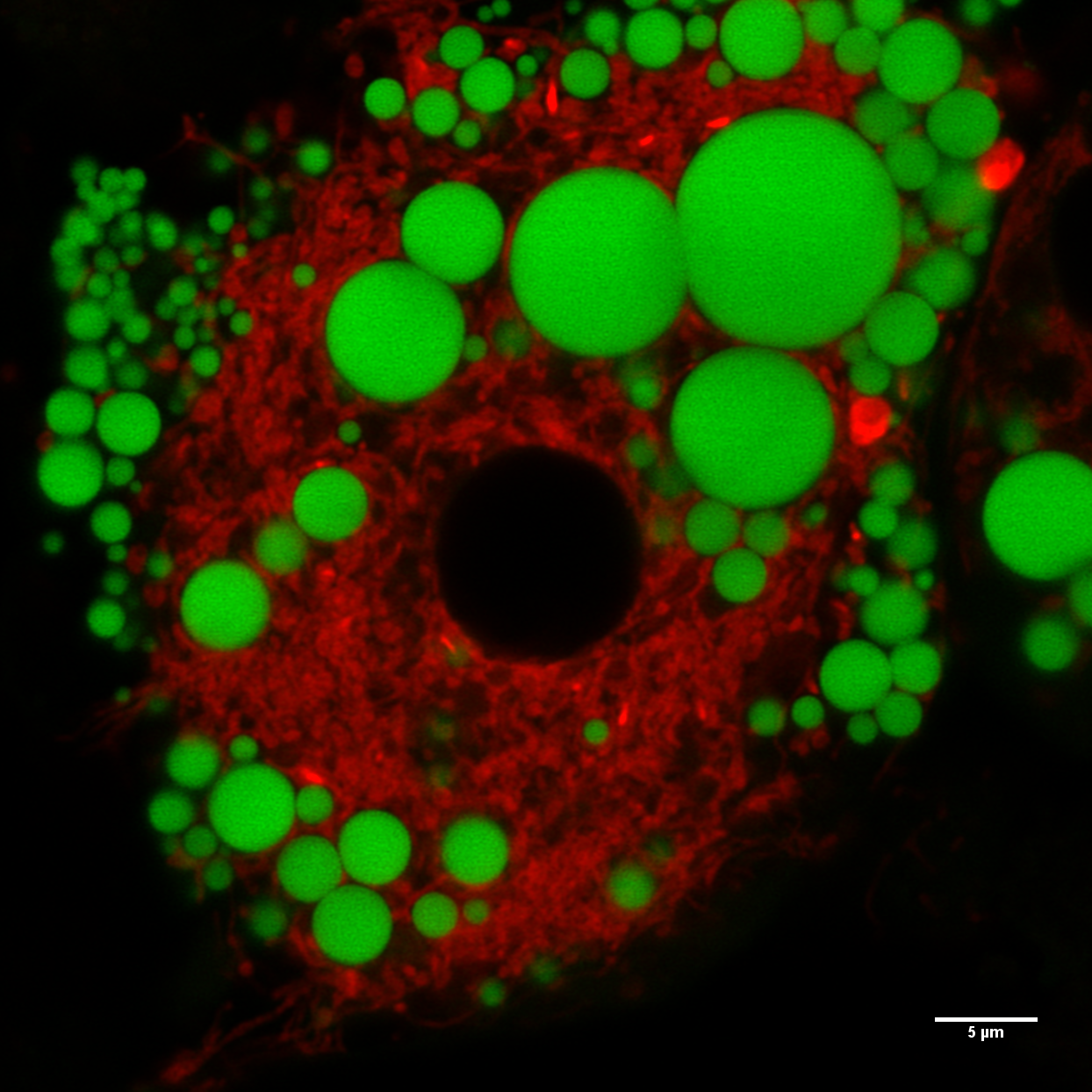

Supplement: Supplementary file 4 — Source data Fig. 2 [file 44318_2026_827_MOESM4_ESM.zip › Figure 2/Figure 2F/Atglstatin 08.png]

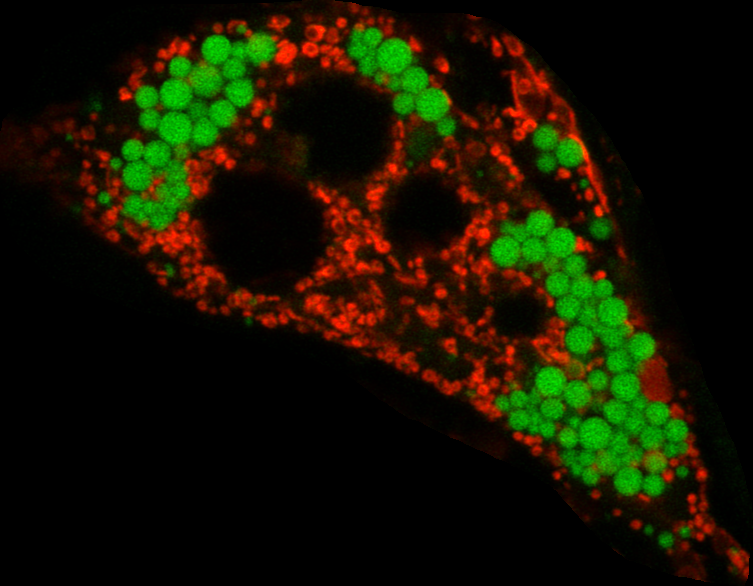

Supplement: Supplementary file 4 — Source data Fig. 2 [file 44318_2026_827_MOESM4_ESM.zip › Figure 2/Figure 2F/unt NE Image 56-Airyscan Processing-1.tif (RGB).tif]

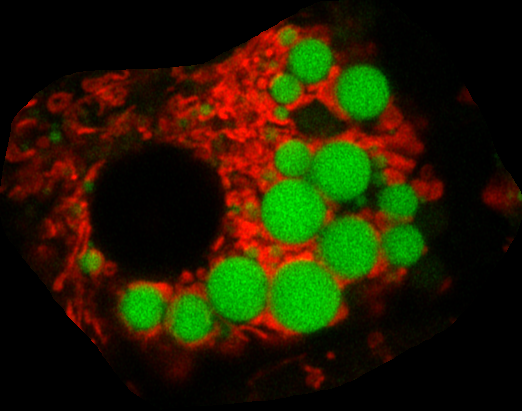

Supplement: Supplementary file 4 — Source data Fig. 2 [file 44318_2026_827_MOESM4_ESM.zip › Figure 2/Figure 2F/unt rotated Image 16-Airyscan Processing-1.tif (RGB).tif]

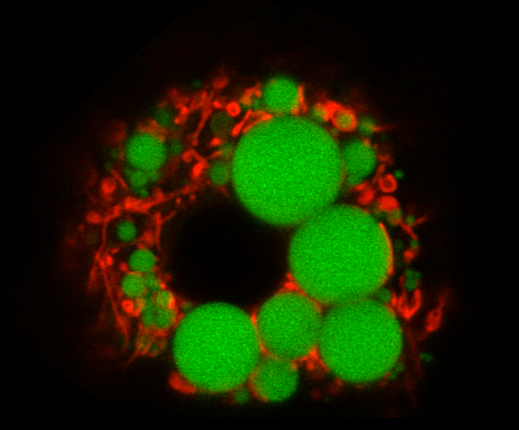

Supplement: Supplementary file 4 — Source data Fig. 2 [file 44318_2026_827_MOESM4_ESM.zip › Figure 2/Figure 2F/Palm Image 34-Airyscan Processing-2.tif (RGB).tif]

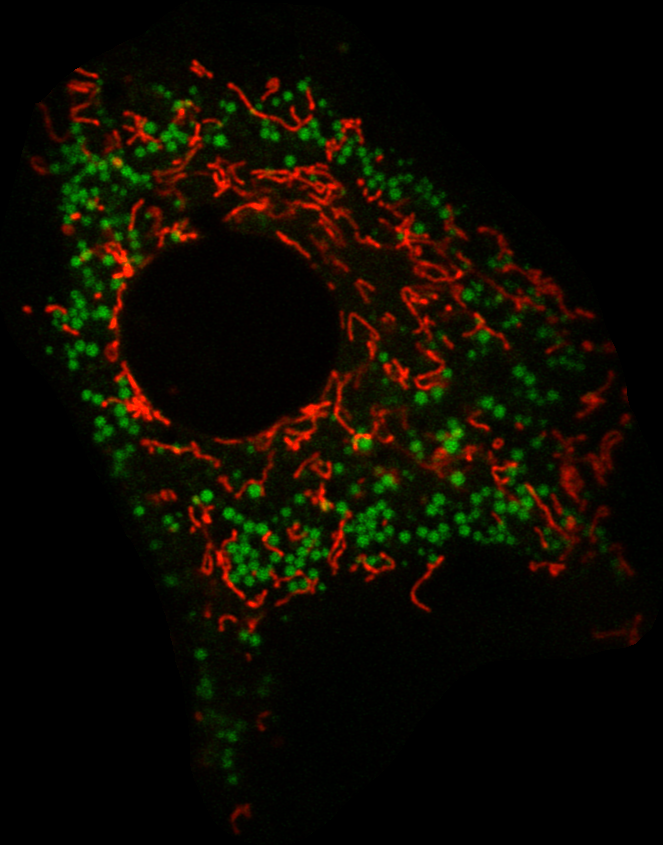

Supplement: Supplementary file 4 — Source data Fig. 2 [file 44318_2026_827_MOESM4_ESM.zip › Figure 2/Figure 2F/Palm NE rotated Image 29-Airyscan Processing-1.tif (RGB).tif]

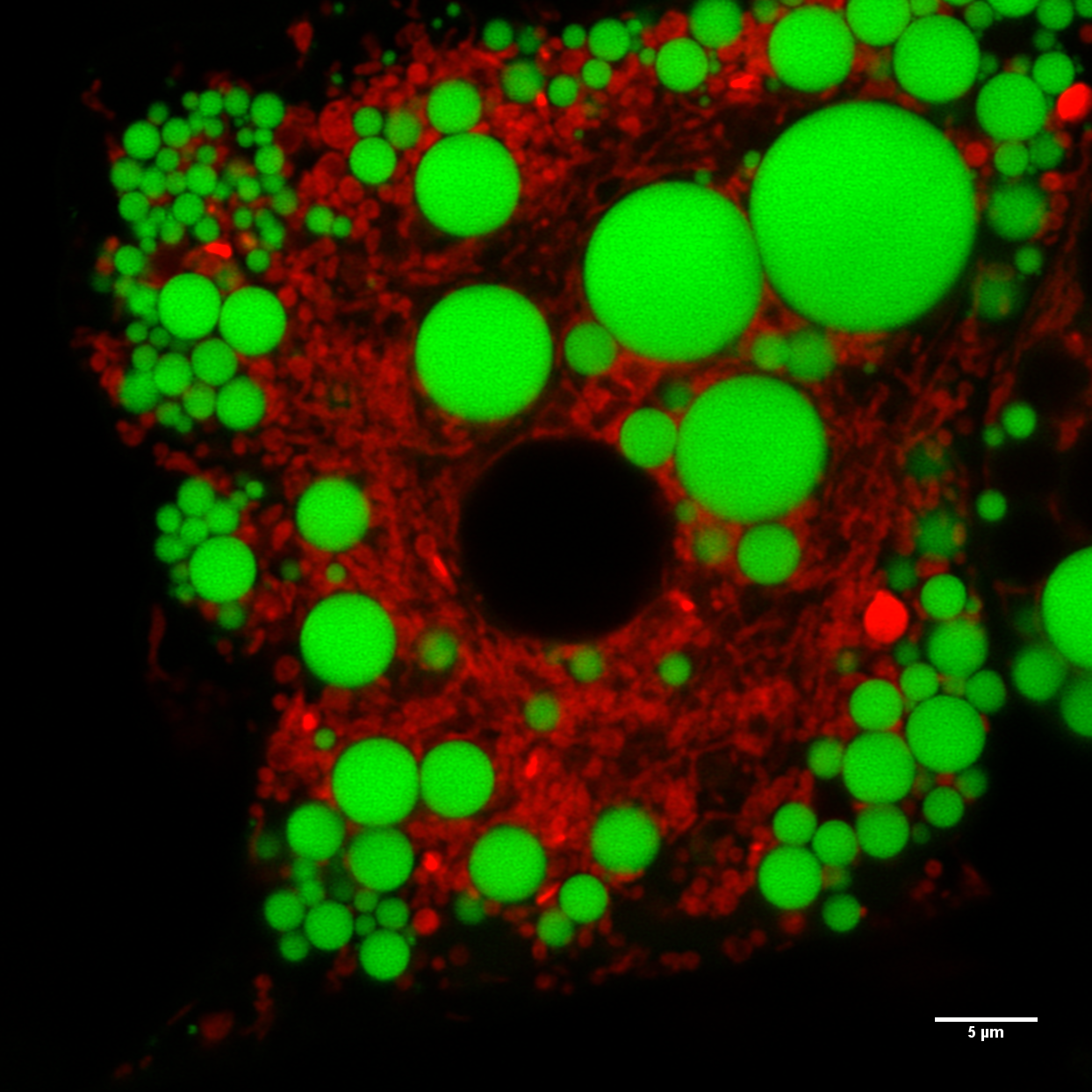

Supplement: Supplementary file 4 — Source data Fig. 2 [file 44318_2026_827_MOESM4_ESM.zip › Figure 2/Figure 2F/Atglstatin NE 08.png]

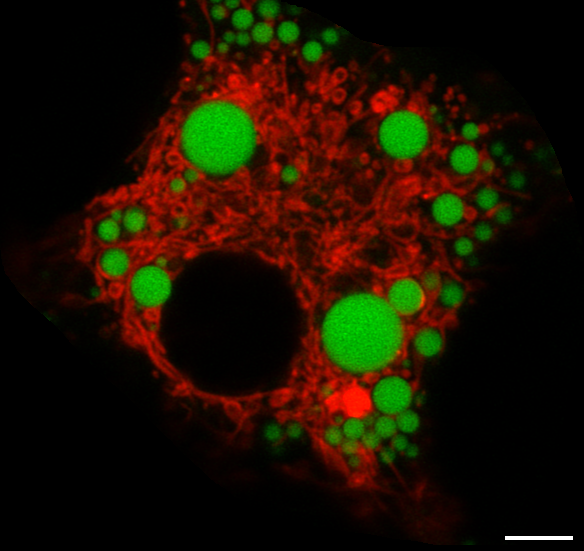

Supplement: Supplementary file 4 — Source data Fig. 2 [file 44318_2026_827_MOESM4_ESM.zip › Figure 2/Figure 2F/RR NE Image 93 0617 RR NE.png]

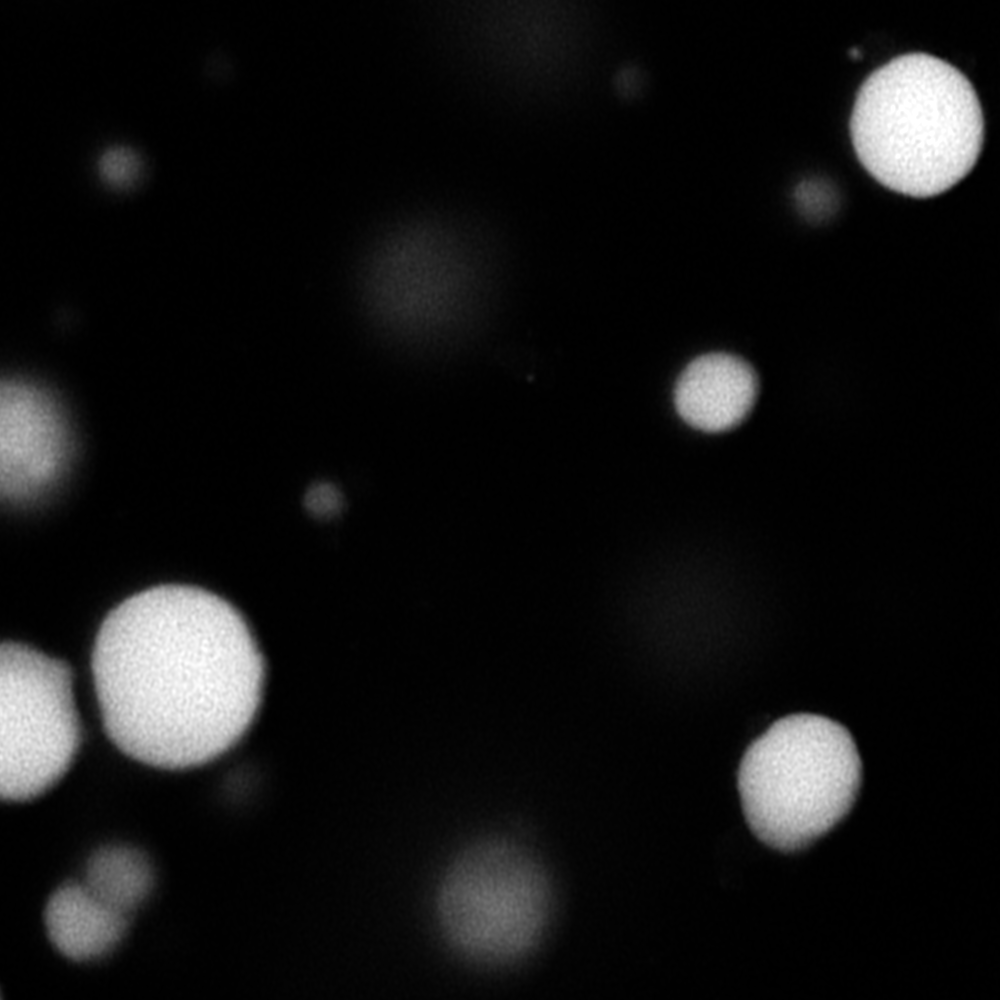

Supplement: Supplementary file 4 — Source data Fig. 2 [file 44318_2026_827_MOESM4_ESM.zip › Figure 2/Figure 2B/Image 105_Out.czi - Z=0 unstrip.tif]

## Slide 1
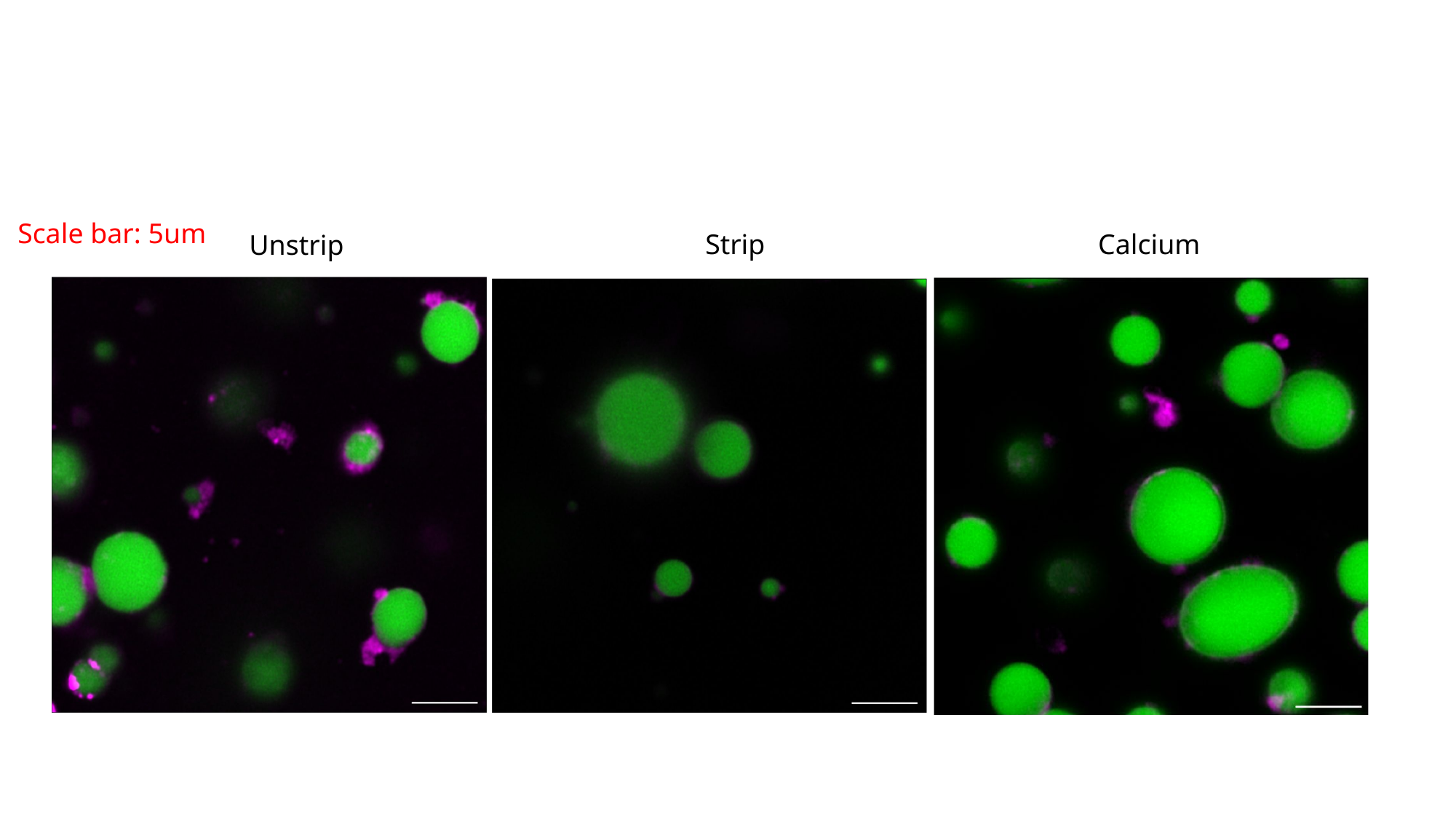

#
Scale bar: 5um
Strip
Calcium
Unstrip

Supplement: Supplementary file 4 — Source data Fig. 2 [file 44318_2026_827_MOESM4_ESM.zip › Figure 2/Figure 2B/Mito in magenta and LD in green pic.pptx]

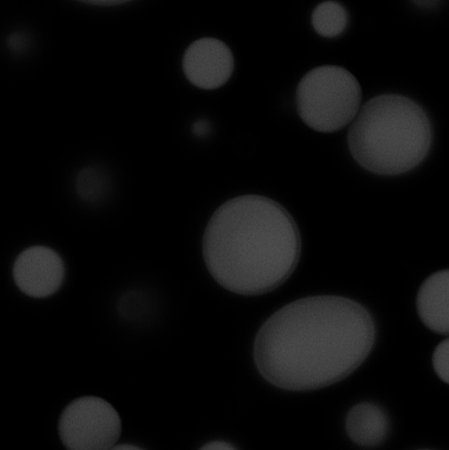

Supplement: Supplementary file 4 — Source data Fig. 2 [file 44318_2026_827_MOESM4_ESM.zip › Figure 2/Figure 2B/Image 36_Out-1-1 scale bar 5um.tif]

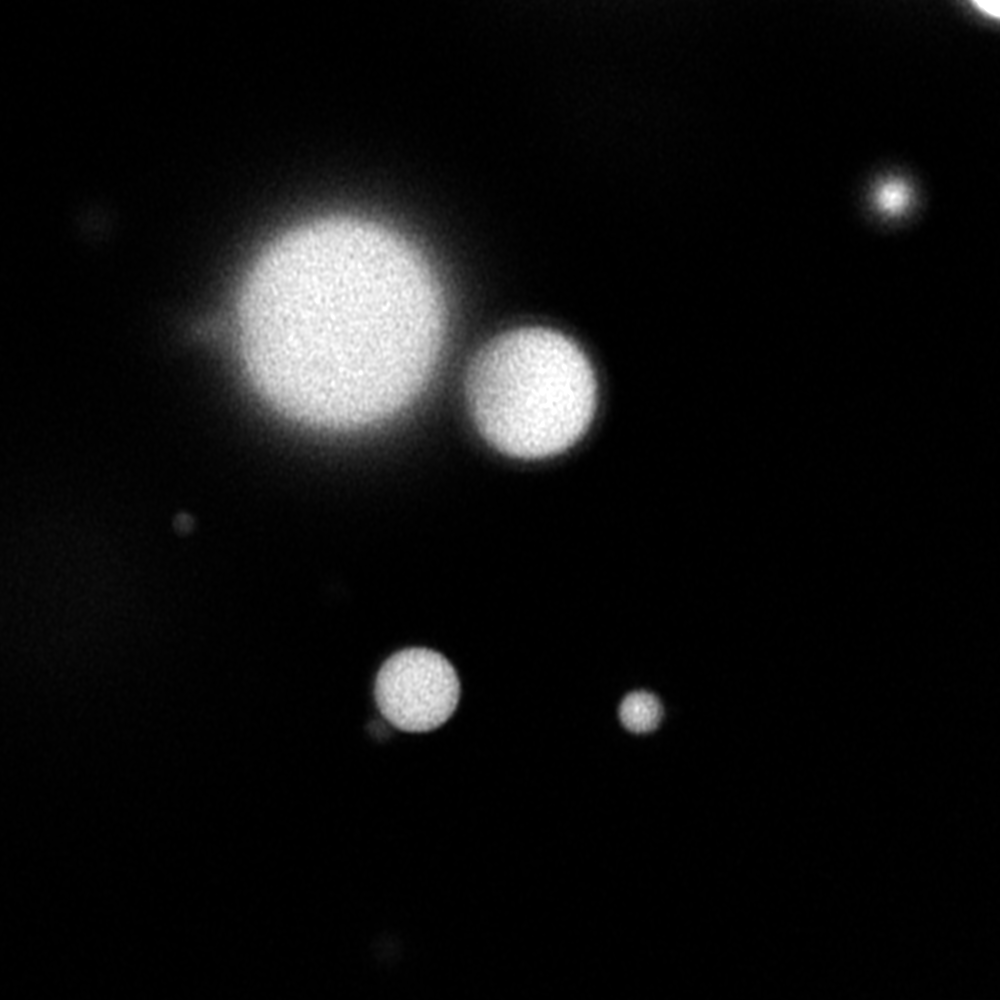

Supplement: Supplementary file 4 — Source data Fig. 2 [file 44318_2026_827_MOESM4_ESM.zip › Figure 2/Figure 2B/Image 89_Out.czi - Z=0 scale bar 5um strip.tif]

## Slide 1
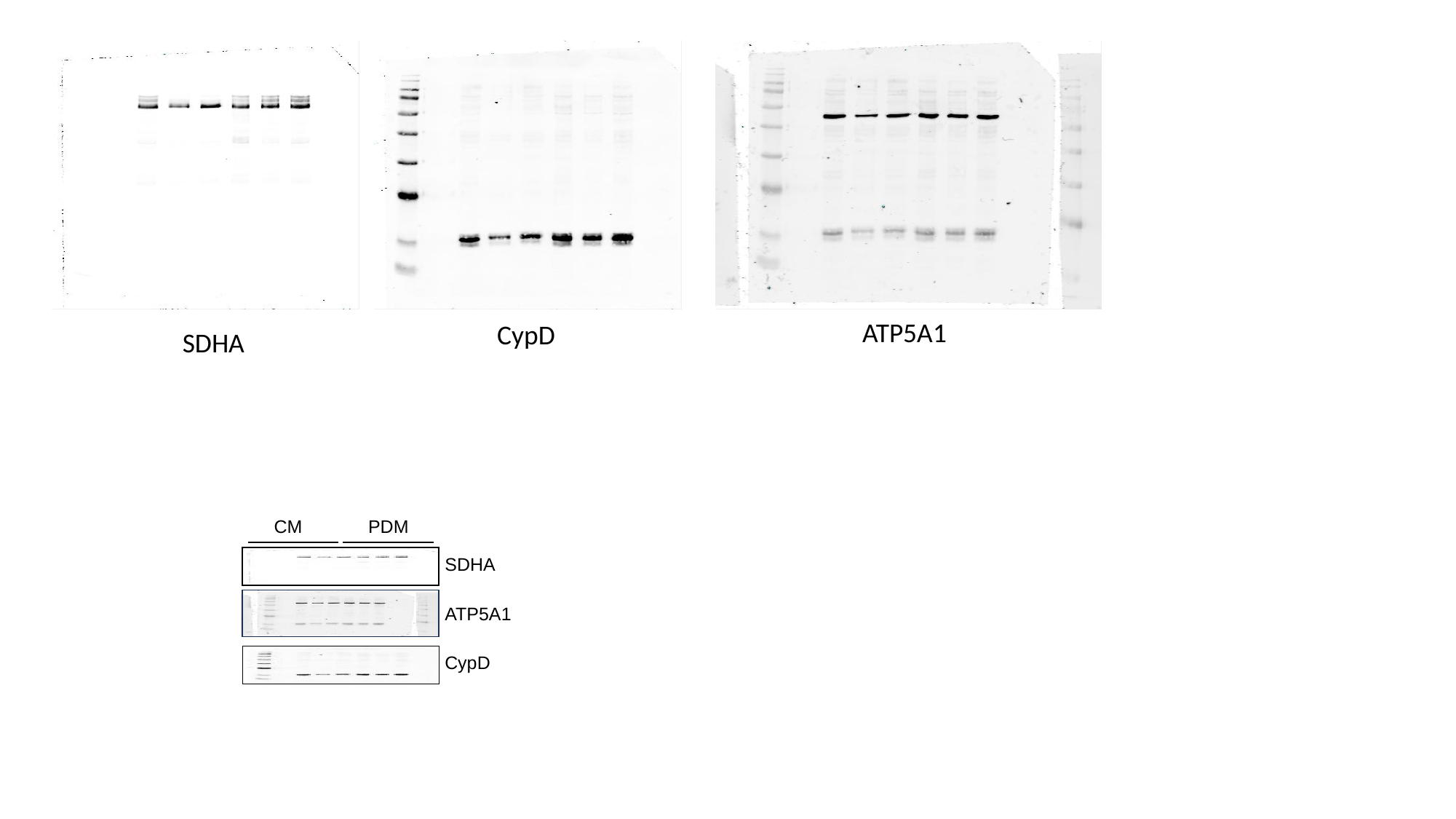

ATP5A1
CypD
SDHA
CM PDM
SDHA
ATP5A1
CypD

Supplement: Supplementary file 5 — Source data Fig. 3 [file 44318_2026_827_MOESM5_ESM.zip › Figure 3/Figure 3P blot.pptx]

## Slide 1
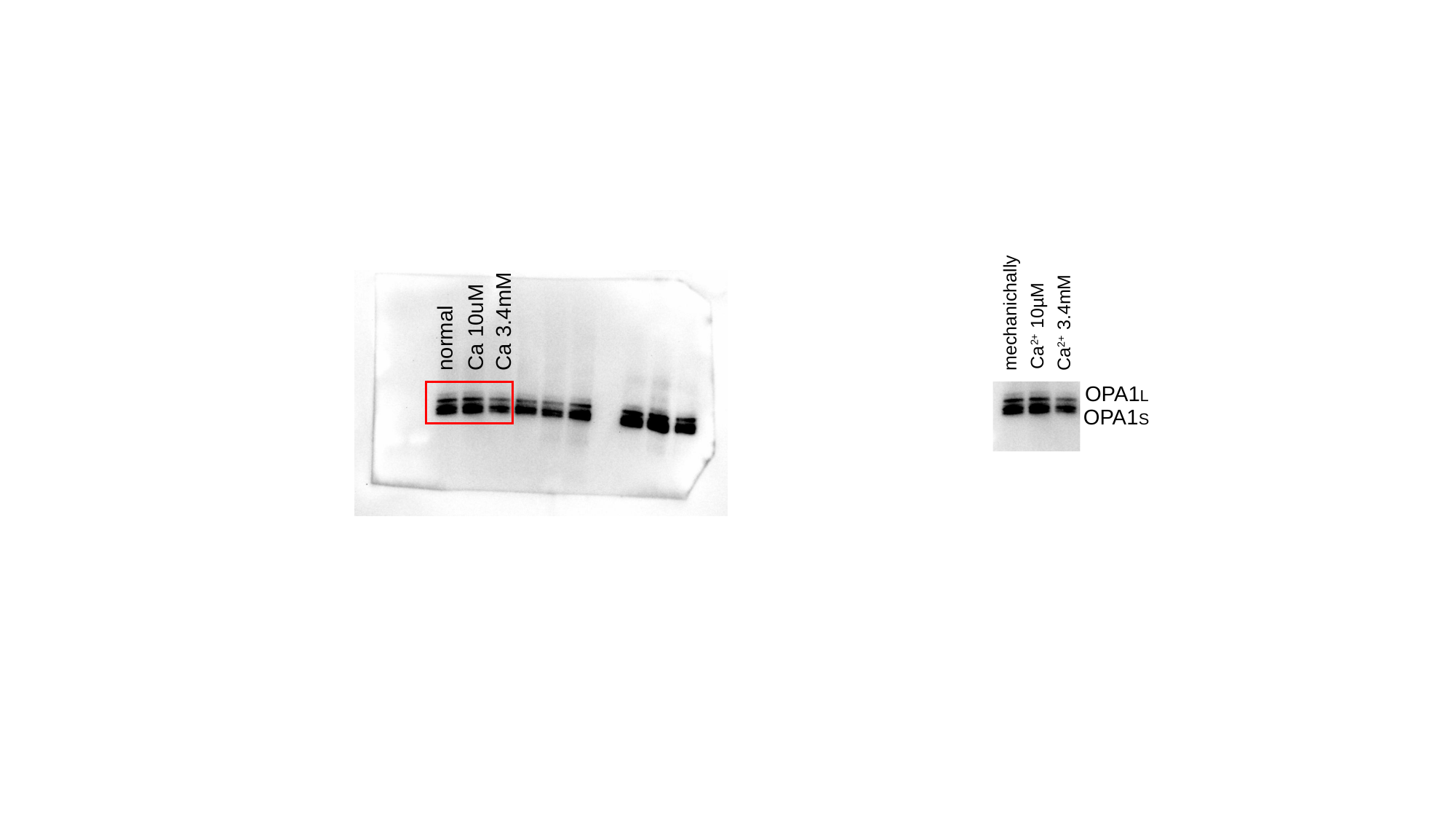

mechanichally
Ca2+ 3.4mM
Ca2+ 10µM
OPA1L
OPA1S
Ca 3.4mM
Ca 10uM
normal

Supplement: Supplementary file 5 — Source data Fig. 3 [file 44318_2026_827_MOESM5_ESM.zip › Figure 3/Figure 3G blot.pptx]

## Slide 1
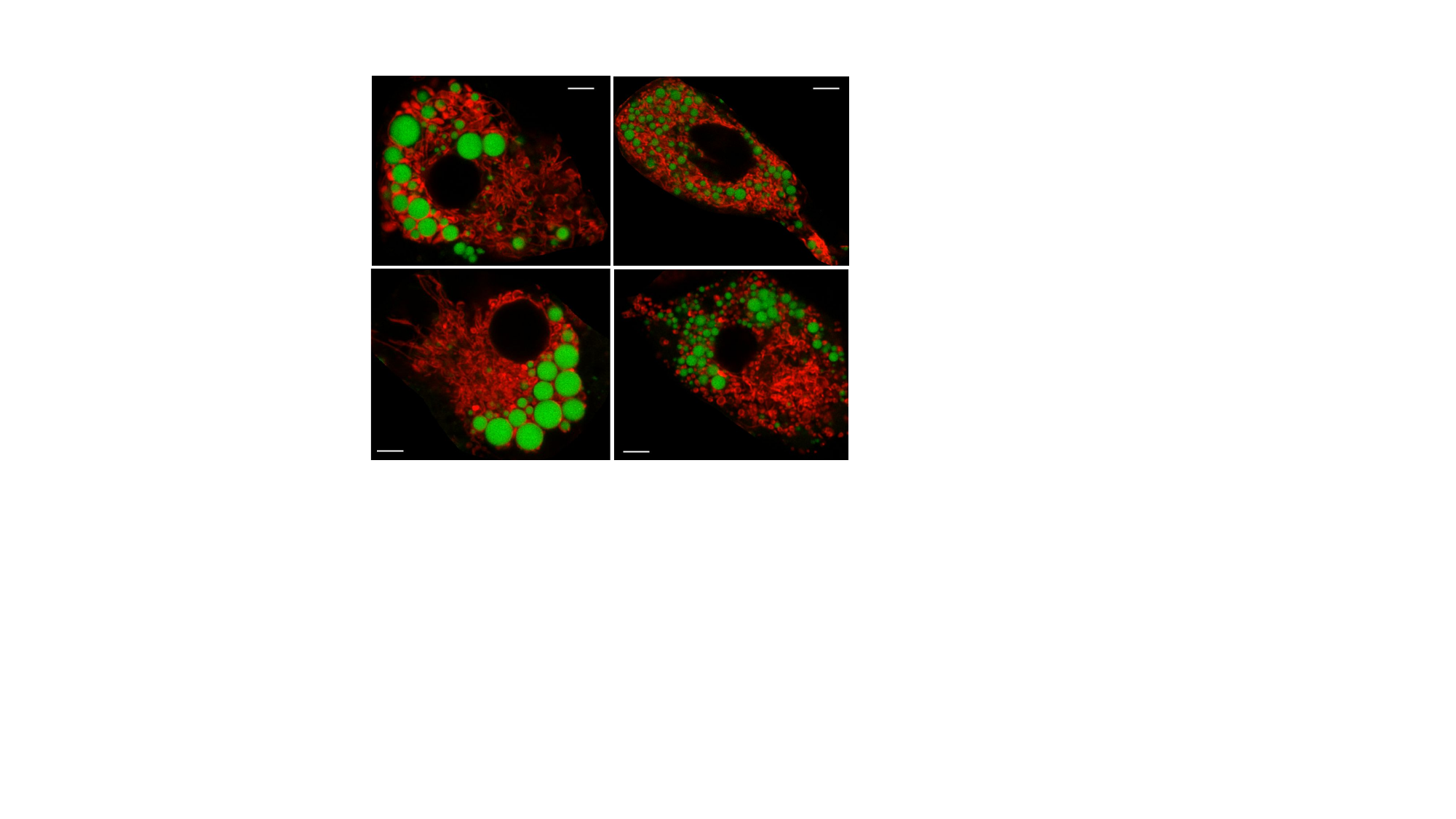

Supplement: Supplementary file 6 — Source data Fig. 4 [file 44318_2026_827_MOESM6_ESM.zip › Figure 4/Figure 4L/Figure 4L imaging composite.pptx]

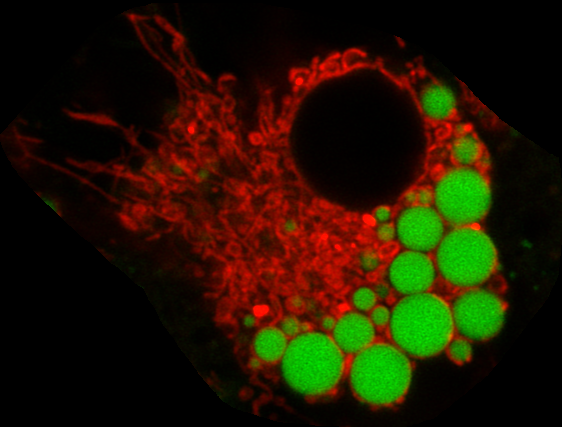

Supplement: Supplementary file 6 — Source data Fig. 4 [file 44318_2026_827_MOESM6_ESM.zip › Figure 4/Figure 4L/CGP Image 113-Airyscan Processing-2.tif (RGB).tif]

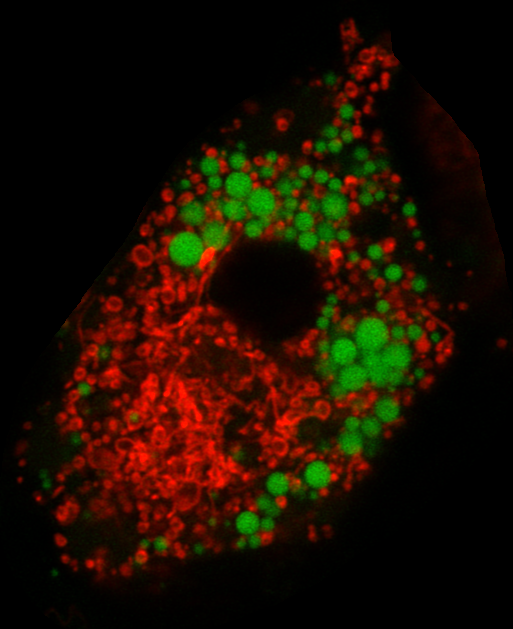

Supplement: Supplementary file 6 — Source data Fig. 4 [file 44318_2026_827_MOESM6_ESM.zip › Figure 4/Figure 4L/CGP NE rotated Image 101-Airyscan Processing-2.tif (RGB).tif]

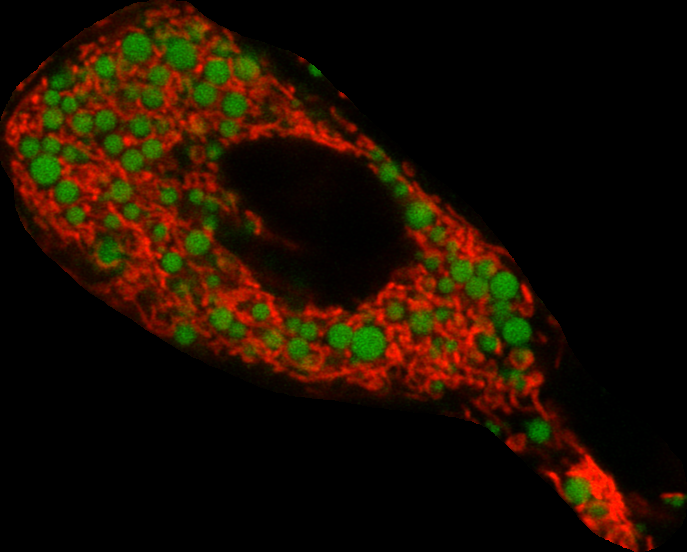

Supplement: Supplementary file 6 — Source data Fig. 4 [file 44318_2026_827_MOESM6_ESM.zip › Figure 4/Figure 4L/unt NE Image 58-Airyscan Processing-1.tif (RGB).tif]

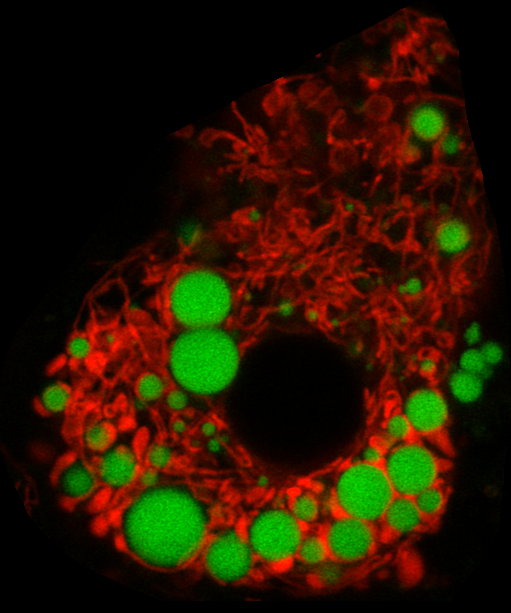

Supplement: Supplementary file 6 — Source data Fig. 4 [file 44318_2026_827_MOESM6_ESM.zip › Figure 4/Figure 4L/unt rotated Image 8-Airyscan Processing-1.tif (RGB).tif]

## Slide 1
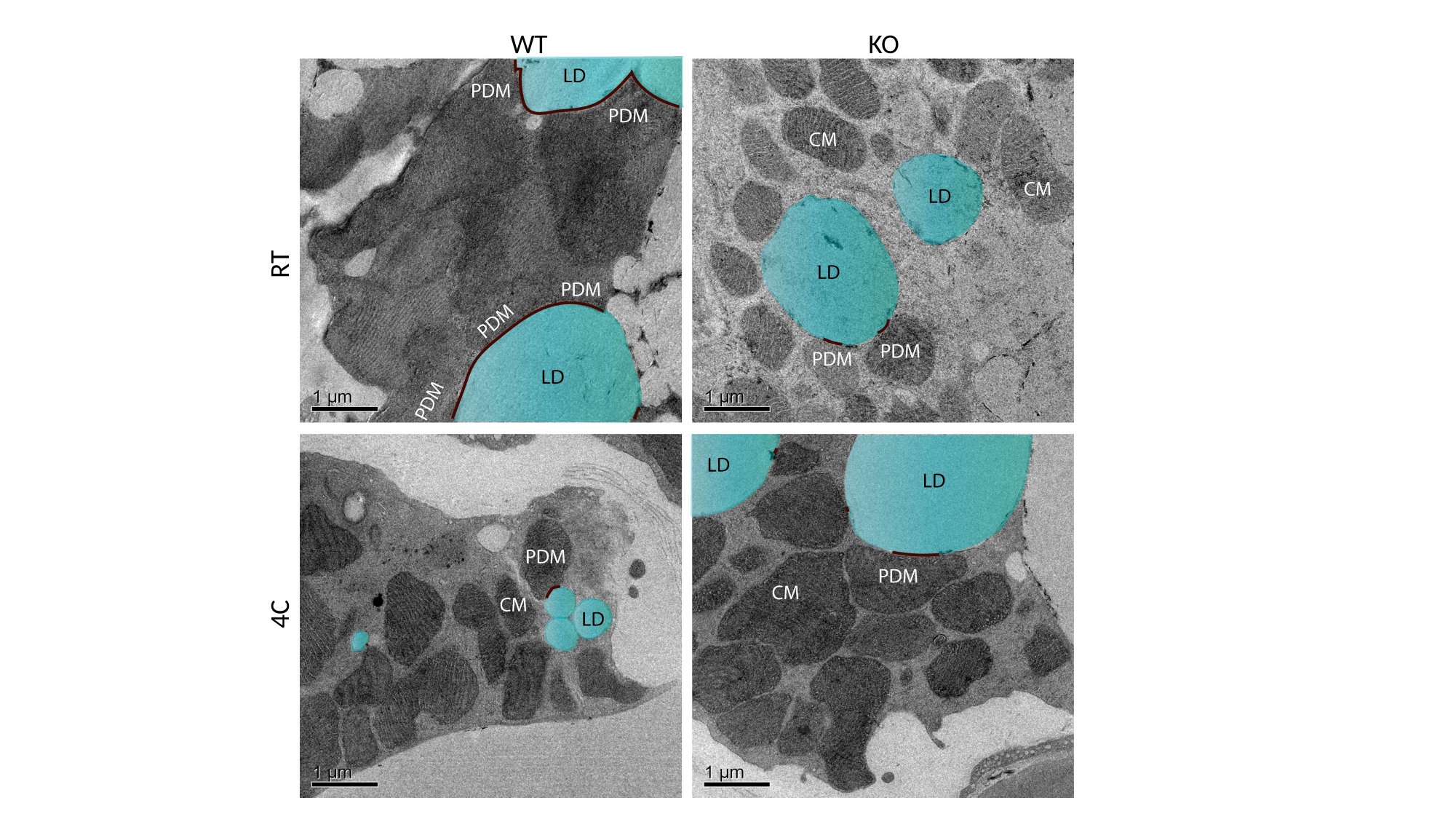

WT KO
4C RT

Supplement: Supplementary file 6 — Source data Fig. 4 [file 44318_2026_827_MOESM6_ESM.zip › Figure 4/Figure 4F/representative pic TEM.pptx]

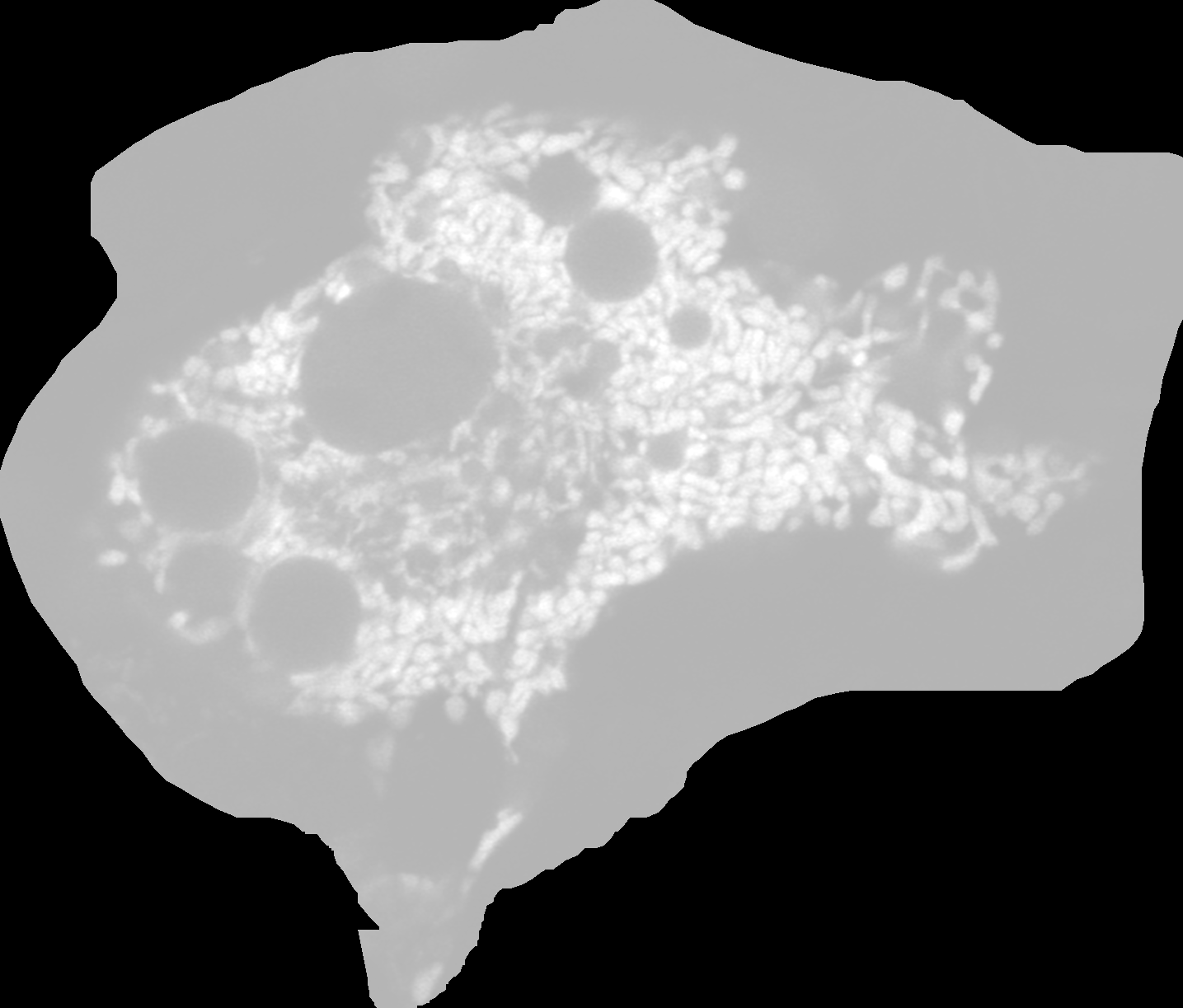

Supplement: Supplementary file 6 — Source data Fig. 4 [file 44318_2026_827_MOESM6_ESM.zip › Figure 4/Figure 4A/stim-1/NE-stim_timeseries_Out-0002-scale-histo-notxt.tif]

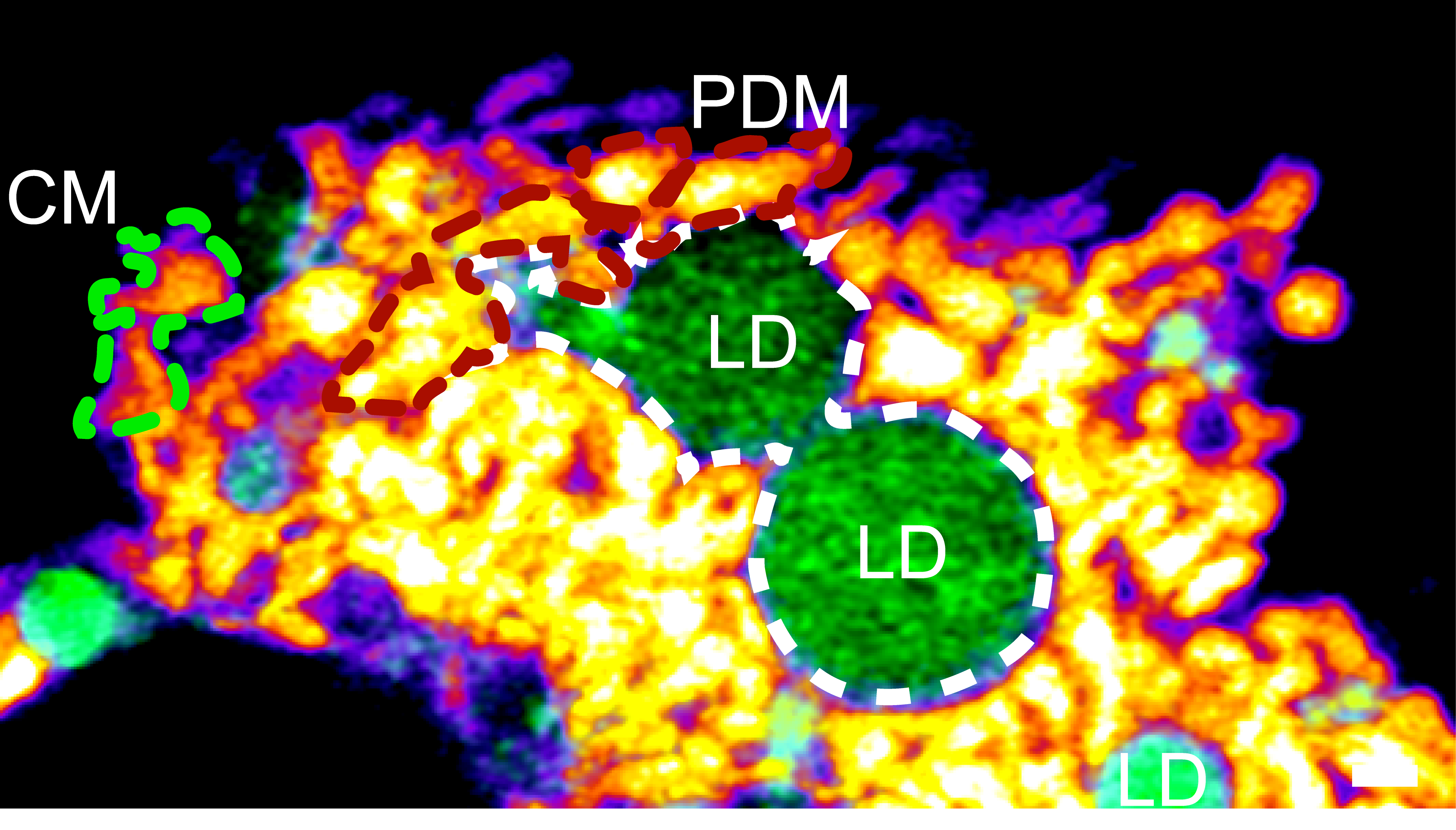

Supplement: Supplementary file 6 — Source data Fig. 4 [file 44318_2026_827_MOESM6_ESM.zip › Figure 4/Figure 4A/stim-1/NE-stim_timeseries_Out.czi - NE-stim_timeseries #7-1-bodipy-1-zoom-scale1.tif-1.tif]

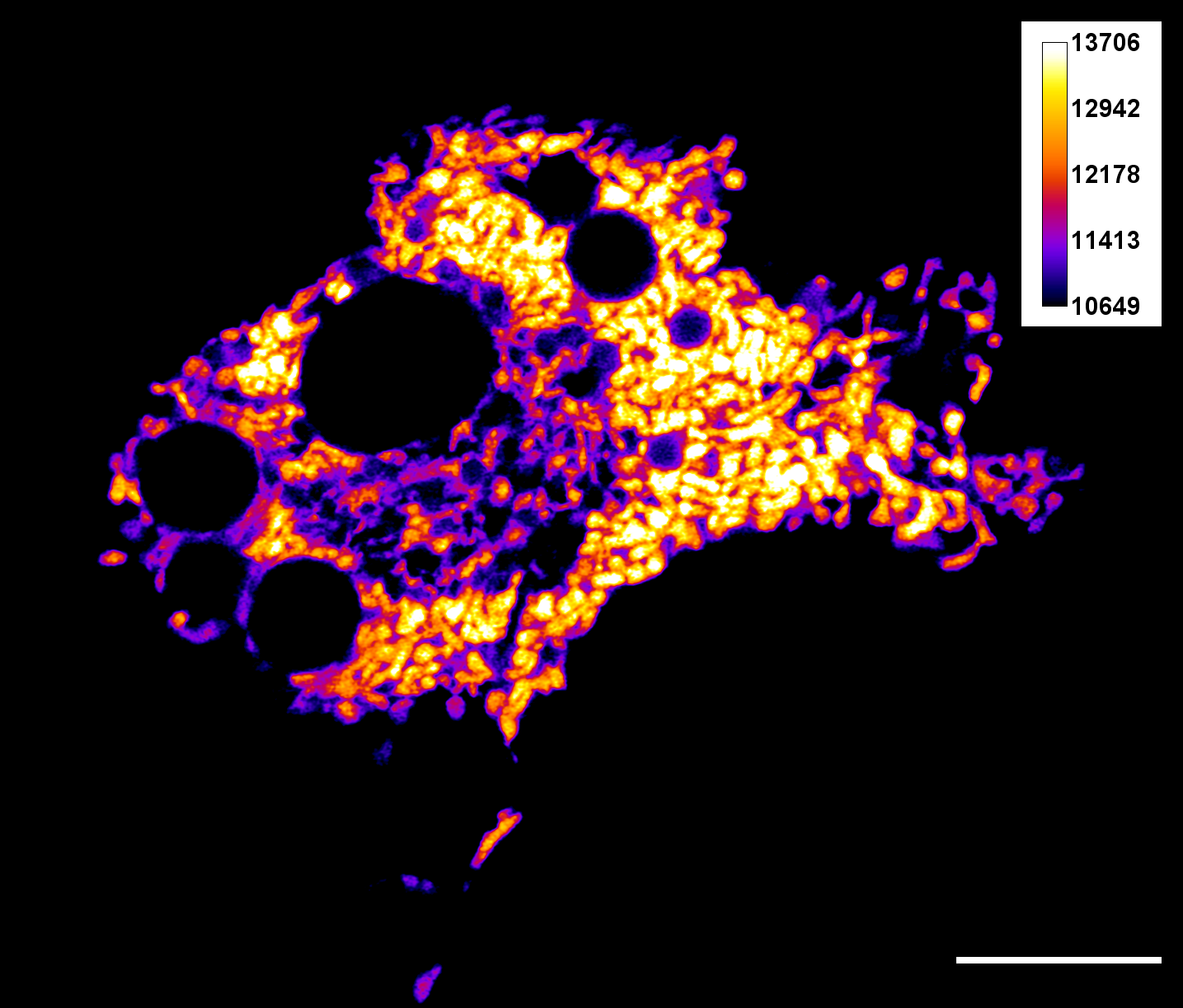

Supplement: Supplementary file 6 — Source data Fig. 4 [file 44318_2026_827_MOESM6_ESM.zip › Figure 4/Figure 4A/stim-1/NE-stim_timeseries_Out-0002-scale-histo-notxt-1rgb.tif]

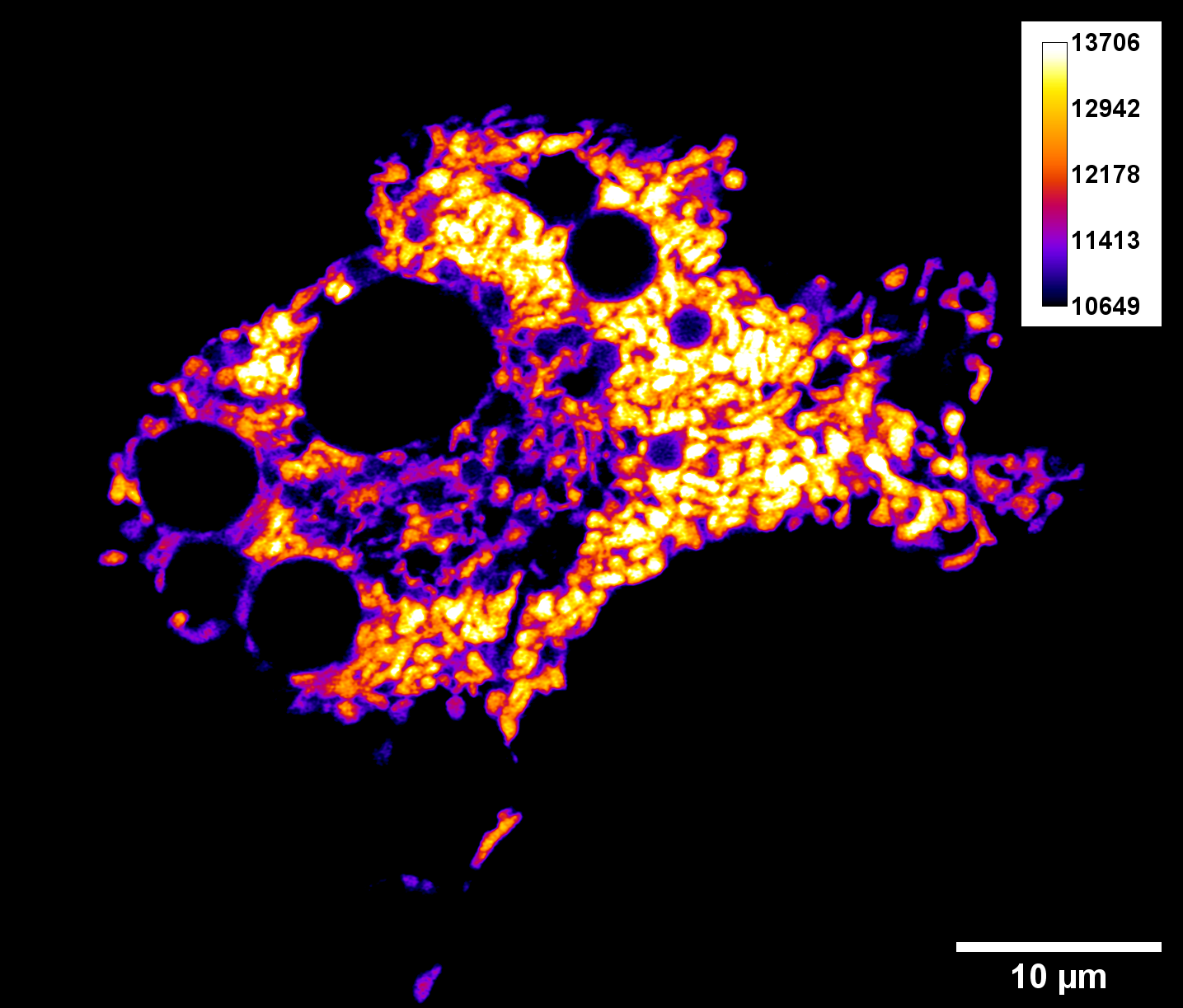

Supplement: Supplementary file 6 — Source data Fig. 4 [file 44318_2026_827_MOESM6_ESM.zip › Figure 4/Figure 4A/stim-1/NE-stim_timeseries_Out-0002-scale-histo-rgb-2.tif]

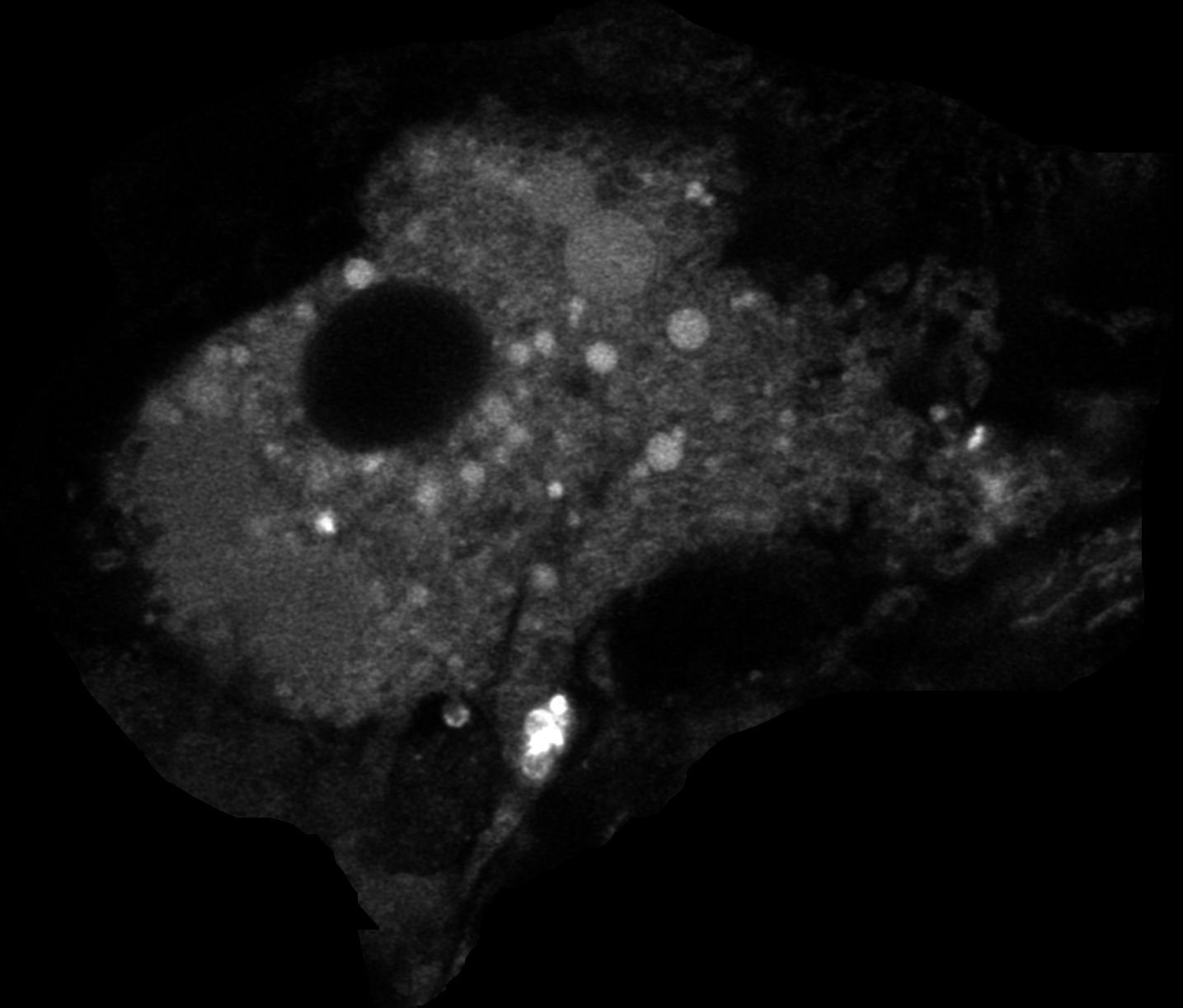

Supplement: Supplementary file 6 — Source data Fig. 4 [file 44318_2026_827_MOESM6_ESM.zip › Figure 4/Figure 4A/stim-1/NE-stim_timeseries_Out.czi - NE-stim_timeseries #7-1-bodipy-2-SELECT.tif]

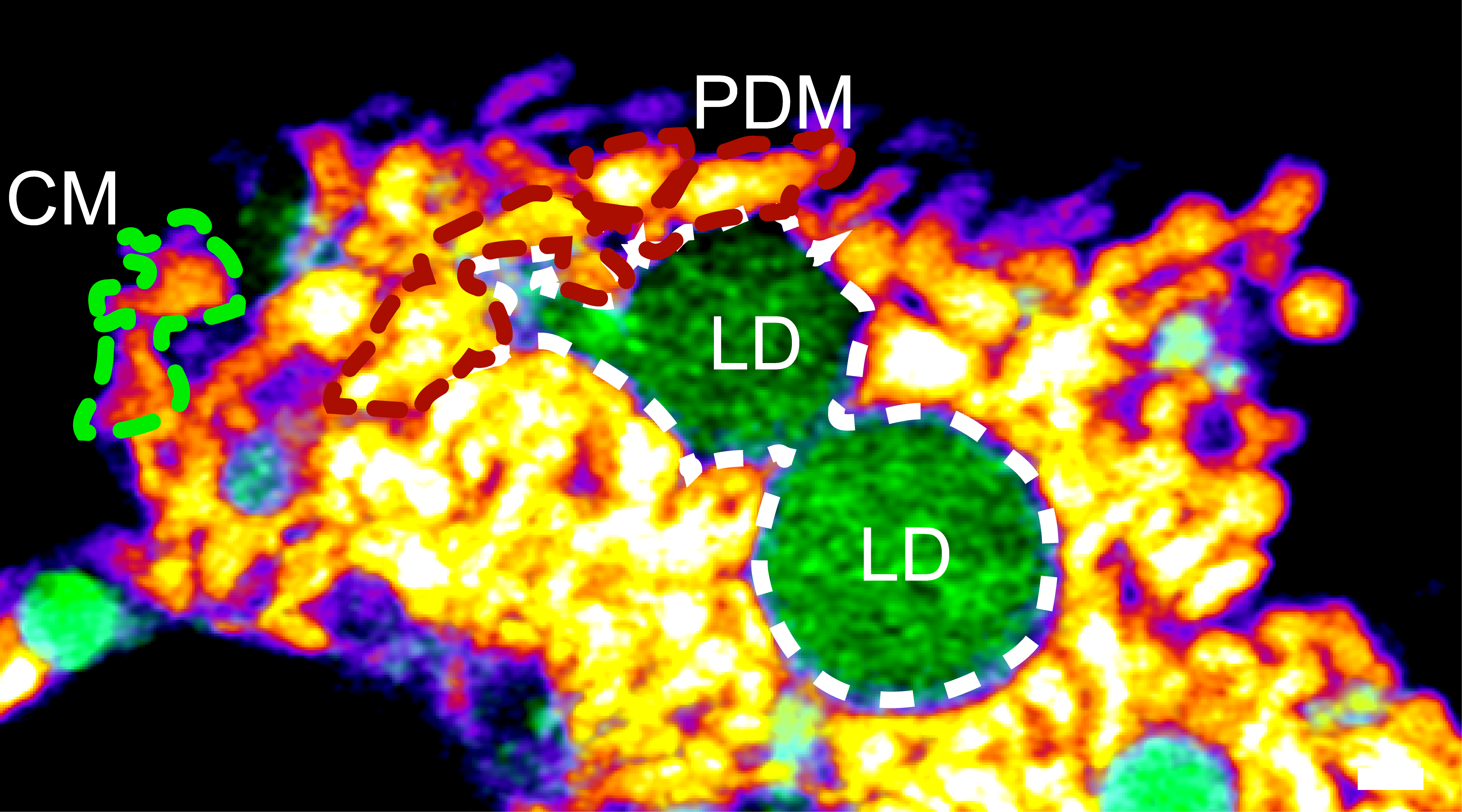

Supplement: Supplementary file 6 — Source data Fig. 4 [file 44318_2026_827_MOESM6_ESM.zip › Figure 4/Figure 4A/stim-1/NE-stim_timeseries_Out.czi - NE-stim_timeseries #7-1-bodipy-1-zoom-scale1.tif-2.tif]

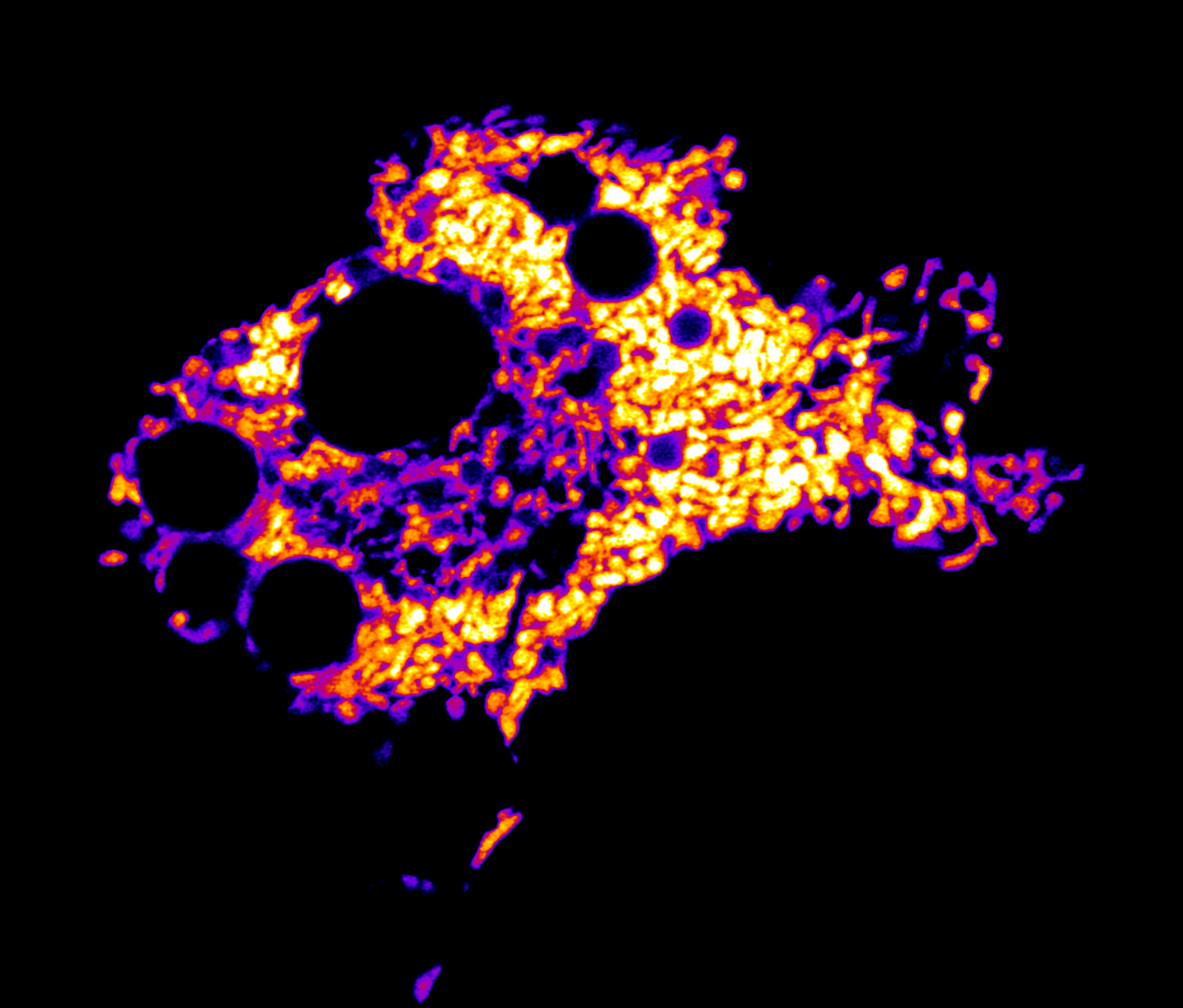

Supplement: Supplementary file 6 — Source data Fig. 4 [file 44318_2026_827_MOESM6_ESM.zip › Figure 4/Figure 4A/stim-1/NE-stim_timeseries_Out-0002-scale-histo-rgb.tif]

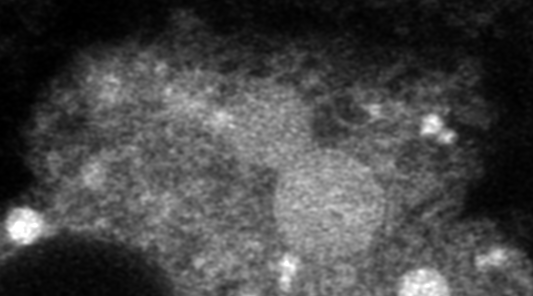

Supplement: Supplementary file 6 — Source data Fig. 4 [file 44318_2026_827_MOESM6_ESM.zip › Figure 4/Figure 4A/stim-1/NE-stim_timeseries_Out.czi - NE-stim_timeseries #7-1-bodipy-1-zoom-scale1.tif]

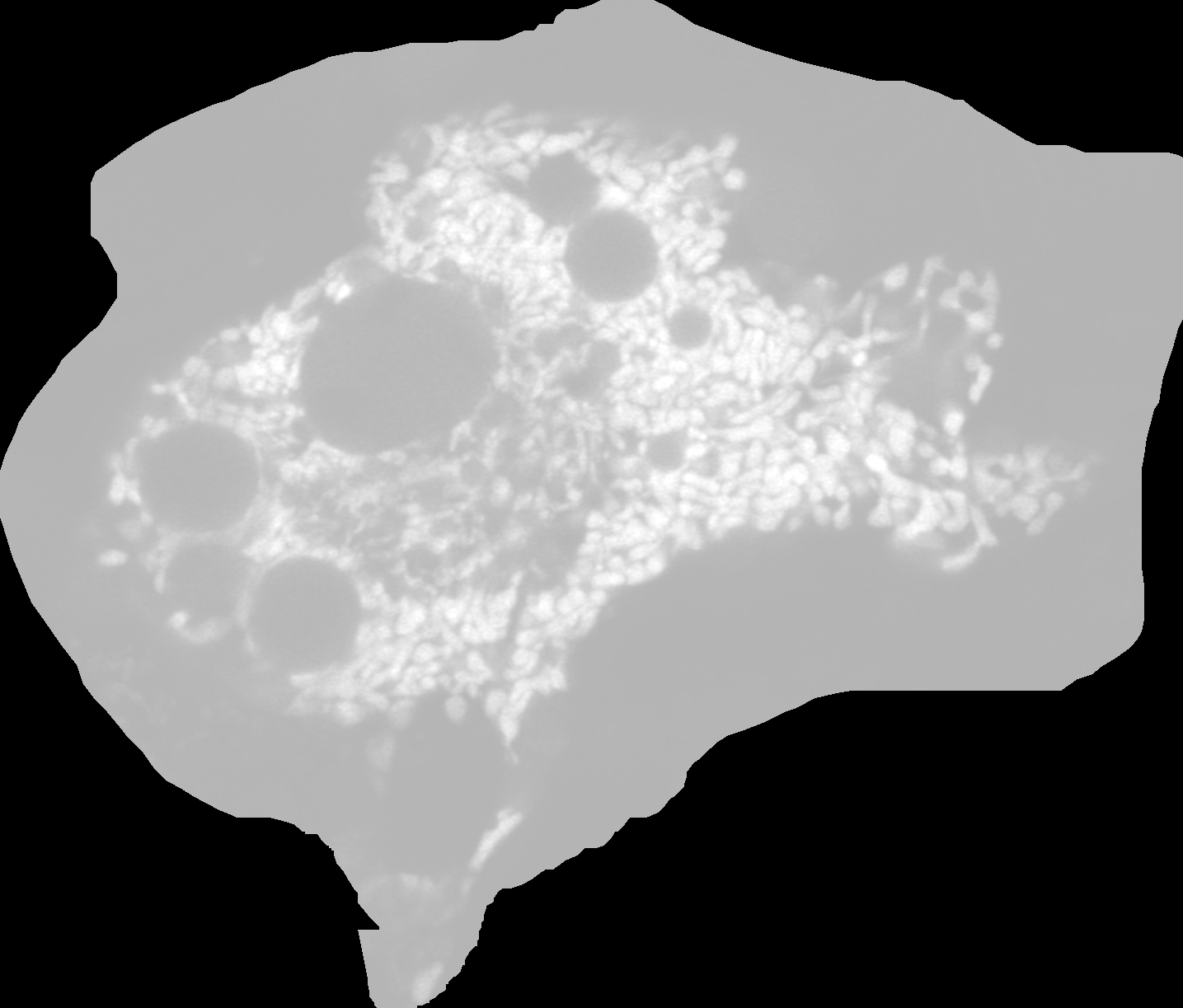

Supplement: Supplementary file 6 — Source data Fig. 4 [file 44318_2026_827_MOESM6_ESM.zip › Figure 4/Figure 4A/stim-1/NE-stim_timeseries_Out-0002-scale-histo.tif]

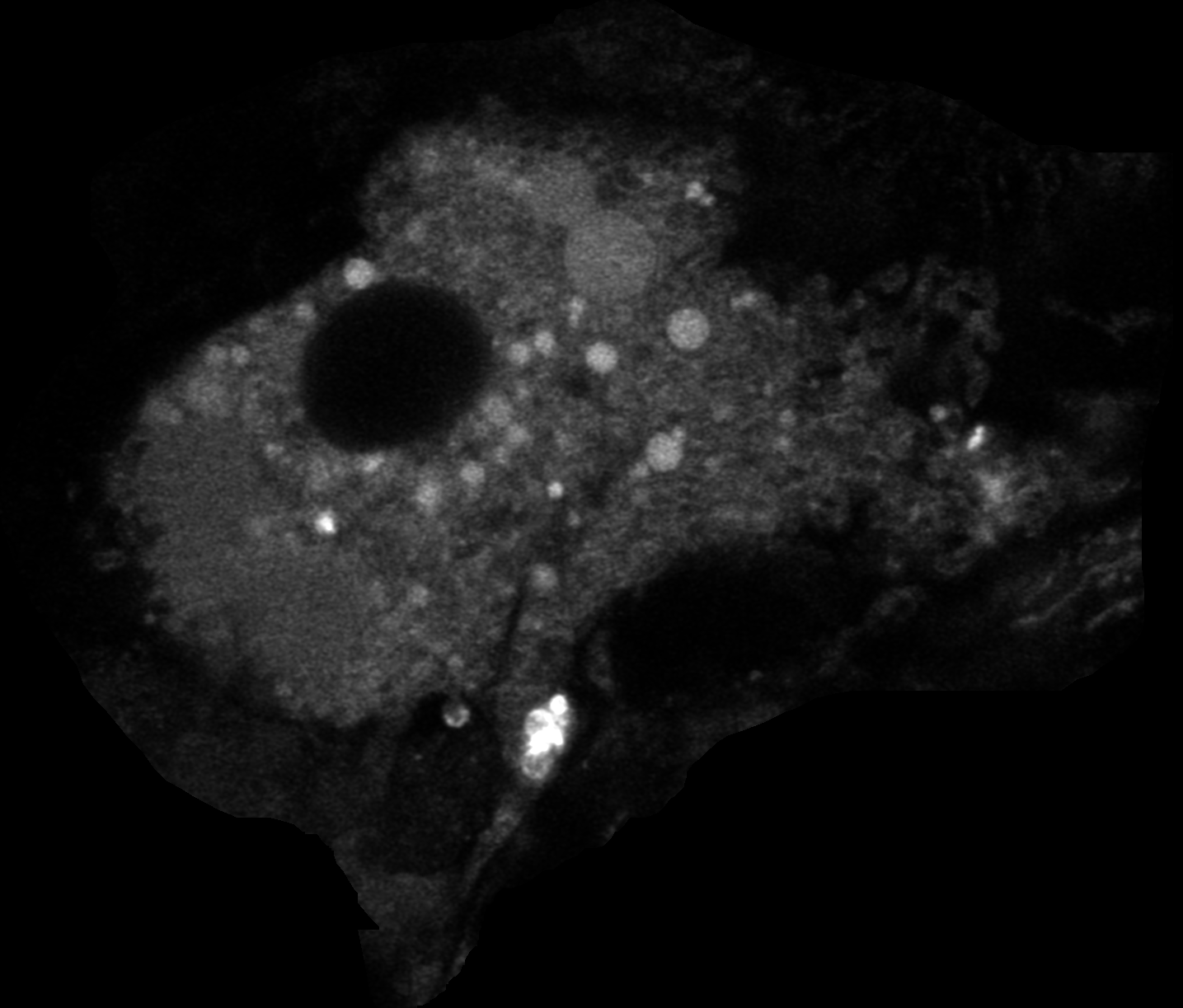

Supplement: Supplementary file 6 — Source data Fig. 4 [file 44318_2026_827_MOESM6_ESM.zip › Figure 4/Figure 4A/stim-1/NE-stim_timeseries_Out.czi - NE-stim_timeseries #7-1-bodipy-2.tif]

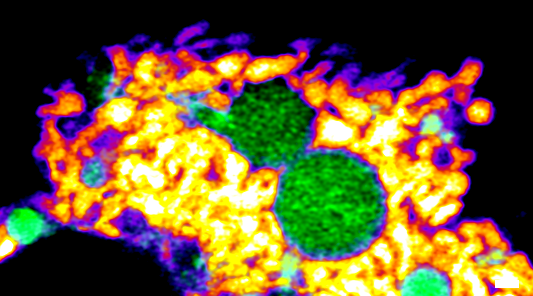

Supplement: Supplementary file 6 — Source data Fig. 4 [file 44318_2026_827_MOESM6_ESM.zip › Figure 4/Figure 4A/stim-1/NE-stim_timeseries_Out.czi - NE-stim_timeseries #7-1-bodipy-1-zoom-scale1.tif (RGB).tif]

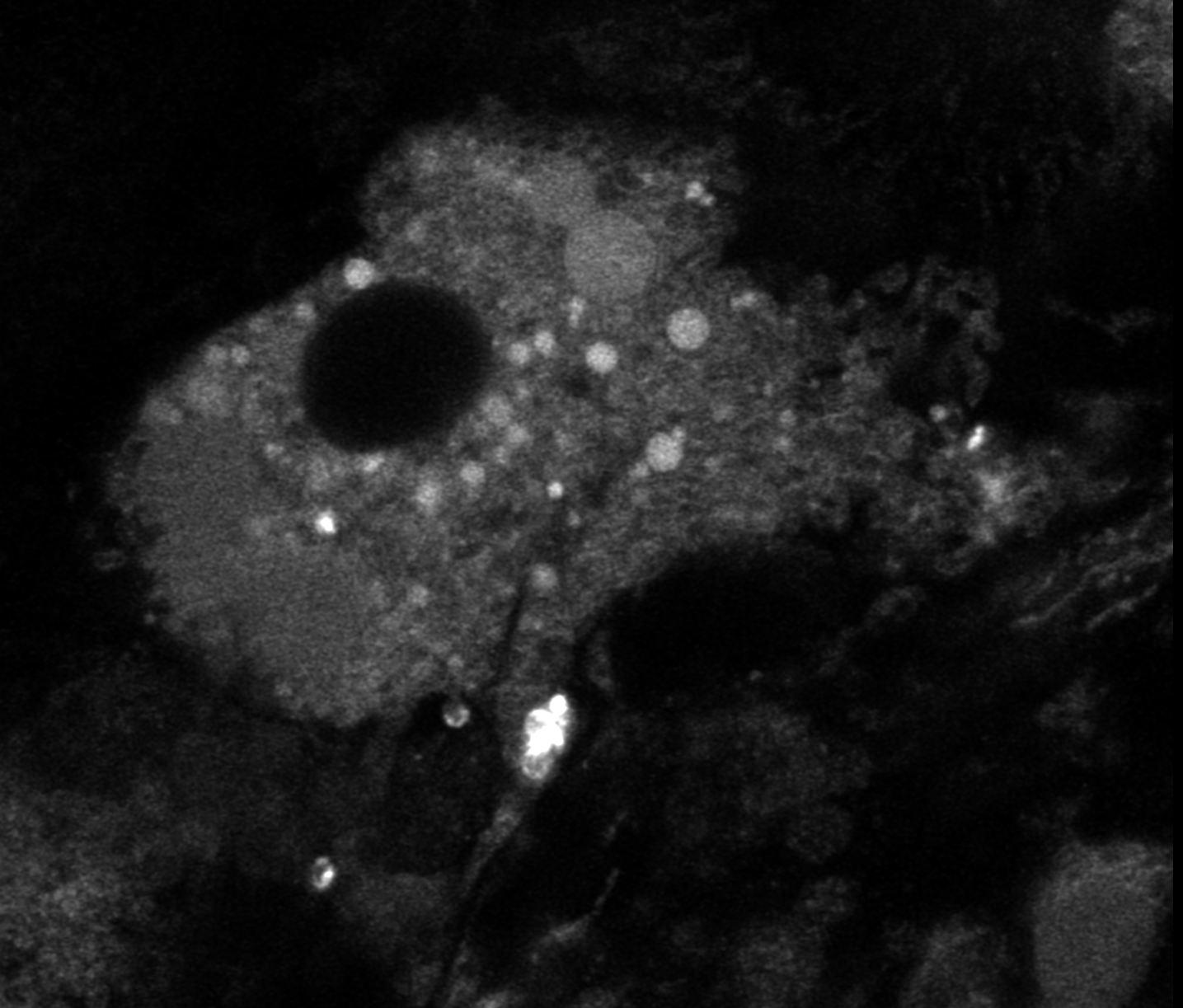

Supplement: Supplementary file 6 — Source data Fig. 4 [file 44318_2026_827_MOESM6_ESM.zip › Figure 4/Figure 4A/stim-1/NE-stim_timeseries_Out.czi - NE-stim_timeseries #7-1-bodipy-1.tif]

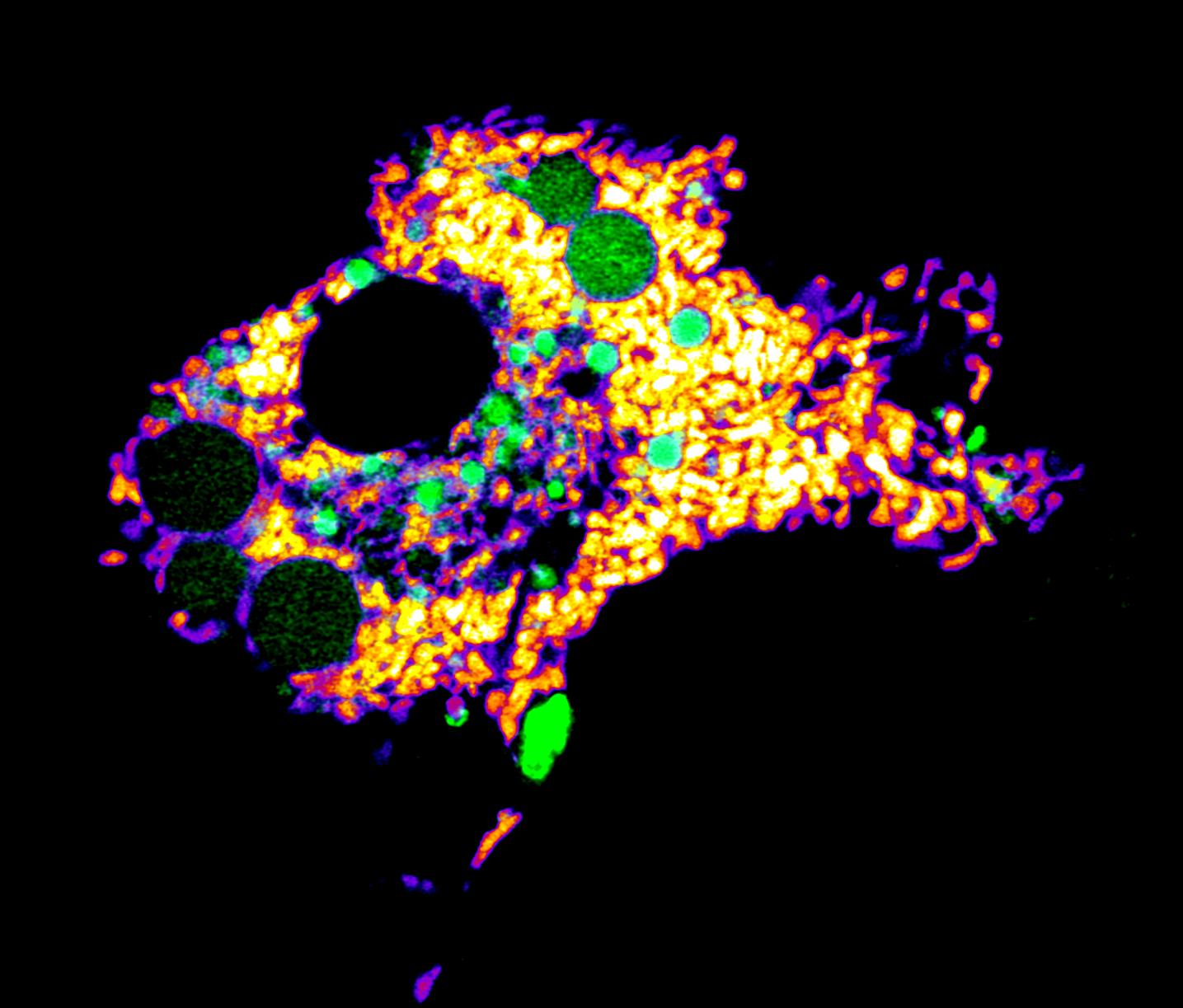

Supplement: Supplementary file 6 — Source data Fig. 4 [file 44318_2026_827_MOESM6_ESM.zip › Figure 4/Figure 4A/stim-1/NE-stim_timeseries_Out.czi - NE-stim_timeseries #7-1-bodipy-2.tif (RGB).tif]

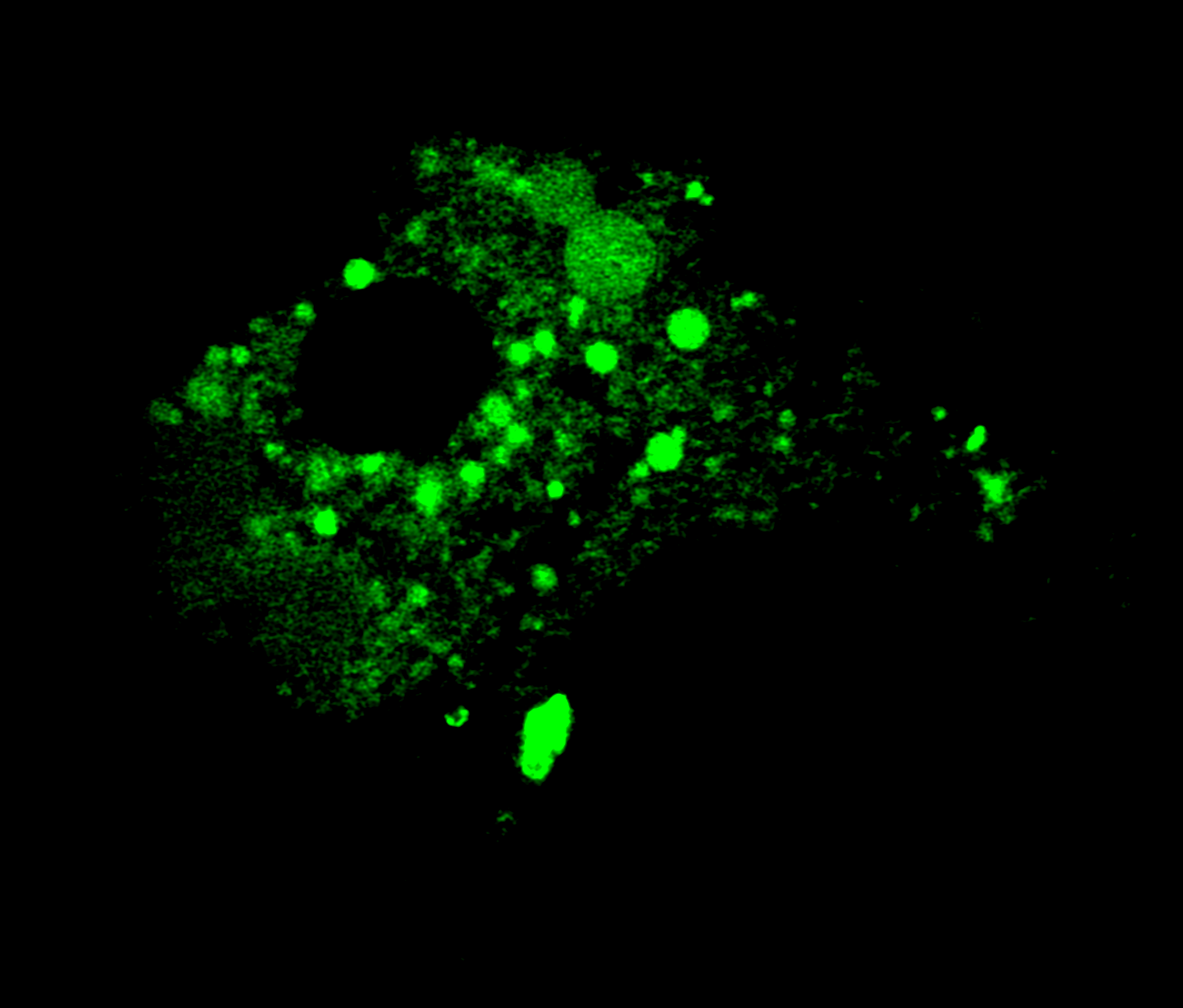

Supplement: Supplementary file 6 — Source data Fig. 4 [file 44318_2026_827_MOESM6_ESM.zip › Figure 4/Figure 4A/stim-1/NE-stim_timeseries_Out-0001-rgb.tif]

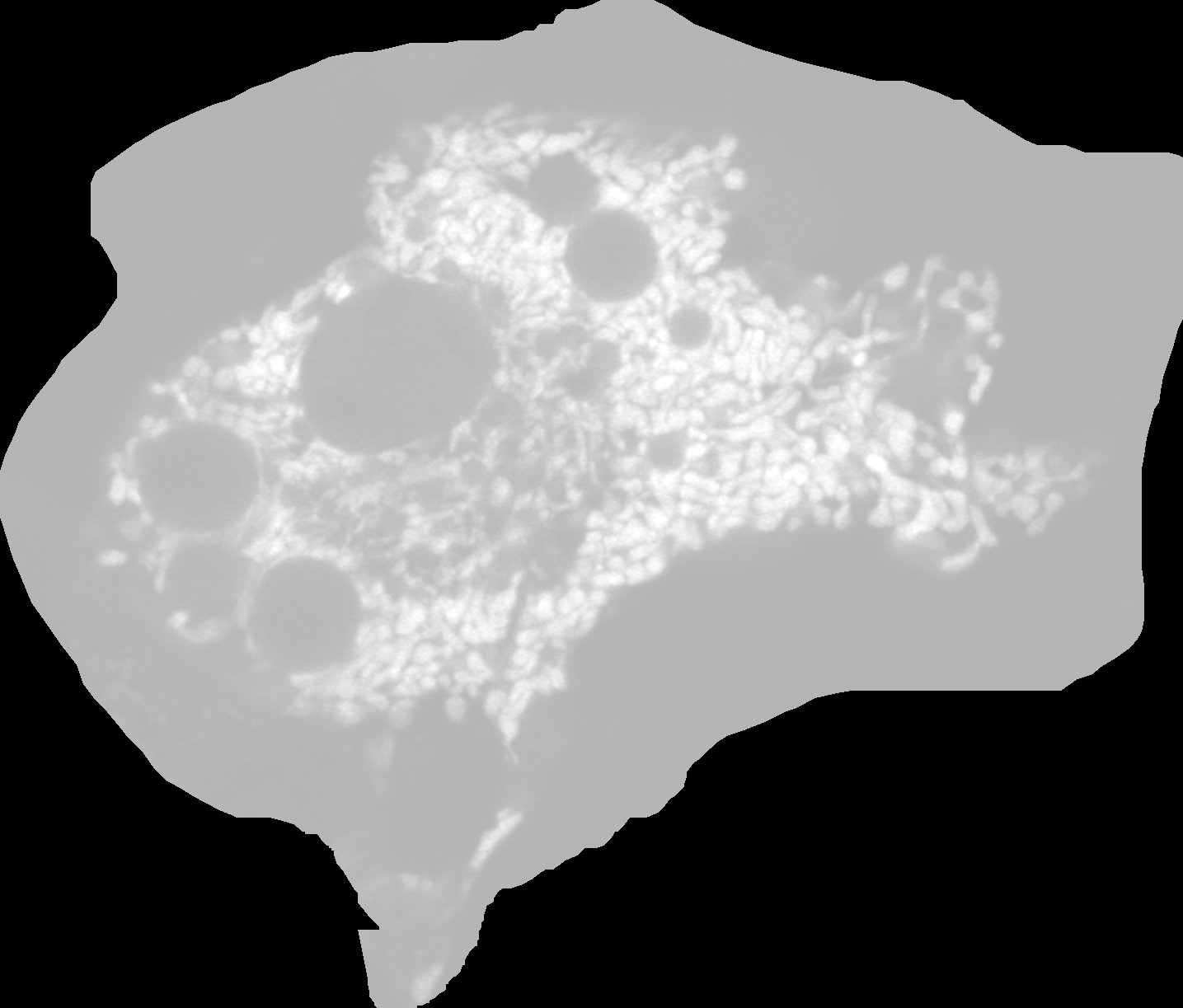

Supplement: Supplementary file 6 — Source data Fig. 4 [file 44318_2026_827_MOESM6_ESM.zip › Figure 4/Figure 4A/stim-1/NE-stim_timeseries_Out-0002.tif]

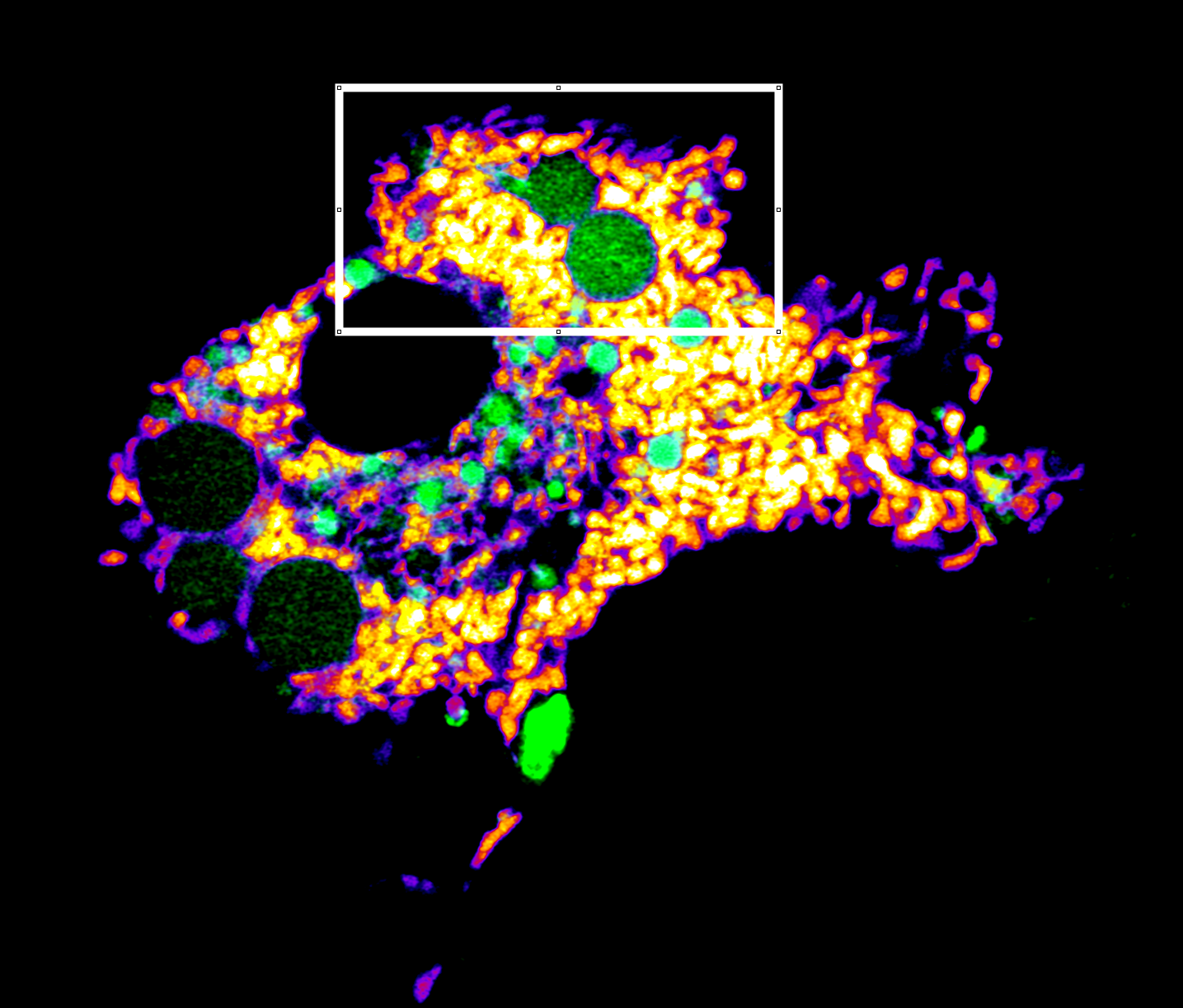

Supplement: Supplementary file 6 — Source data Fig. 4 [file 44318_2026_827_MOESM6_ESM.zip › Figure 4/Figure 4A/stim-1/NE-stim_timeseries_Out.czi - NE-stim_timeseries #7-1-bodipy-2-SELECT.tif (RGB)-2.tif]

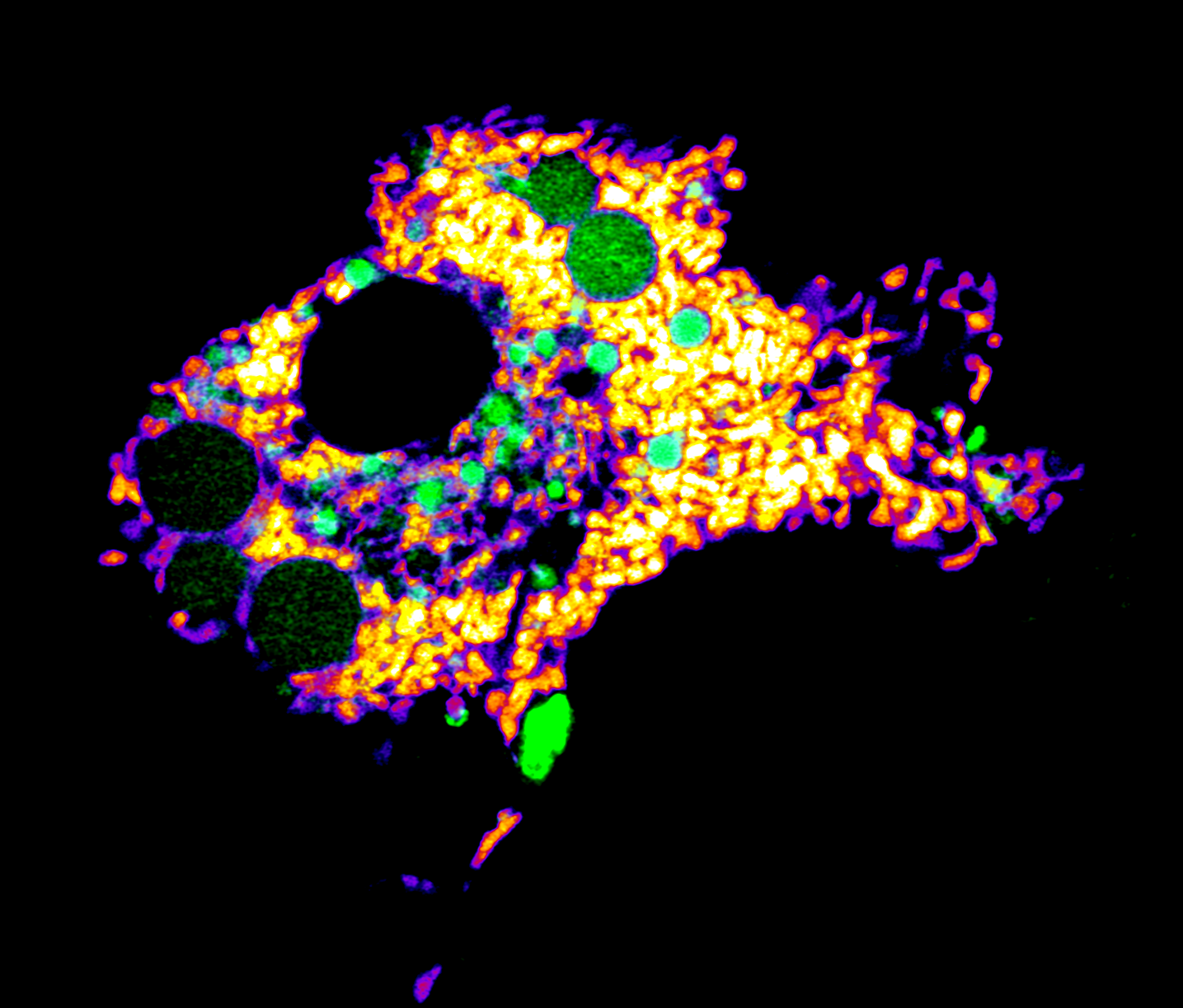

Supplement: Supplementary file 6 — Source data Fig. 4 [file 44318_2026_827_MOESM6_ESM.zip › Figure 4/Figure 4A/stim-1/NE-stim_timeseries_Out.czi - NE-stim_timeseries #7-1-bodipy-2-SELECT.tif (RGB).tif]

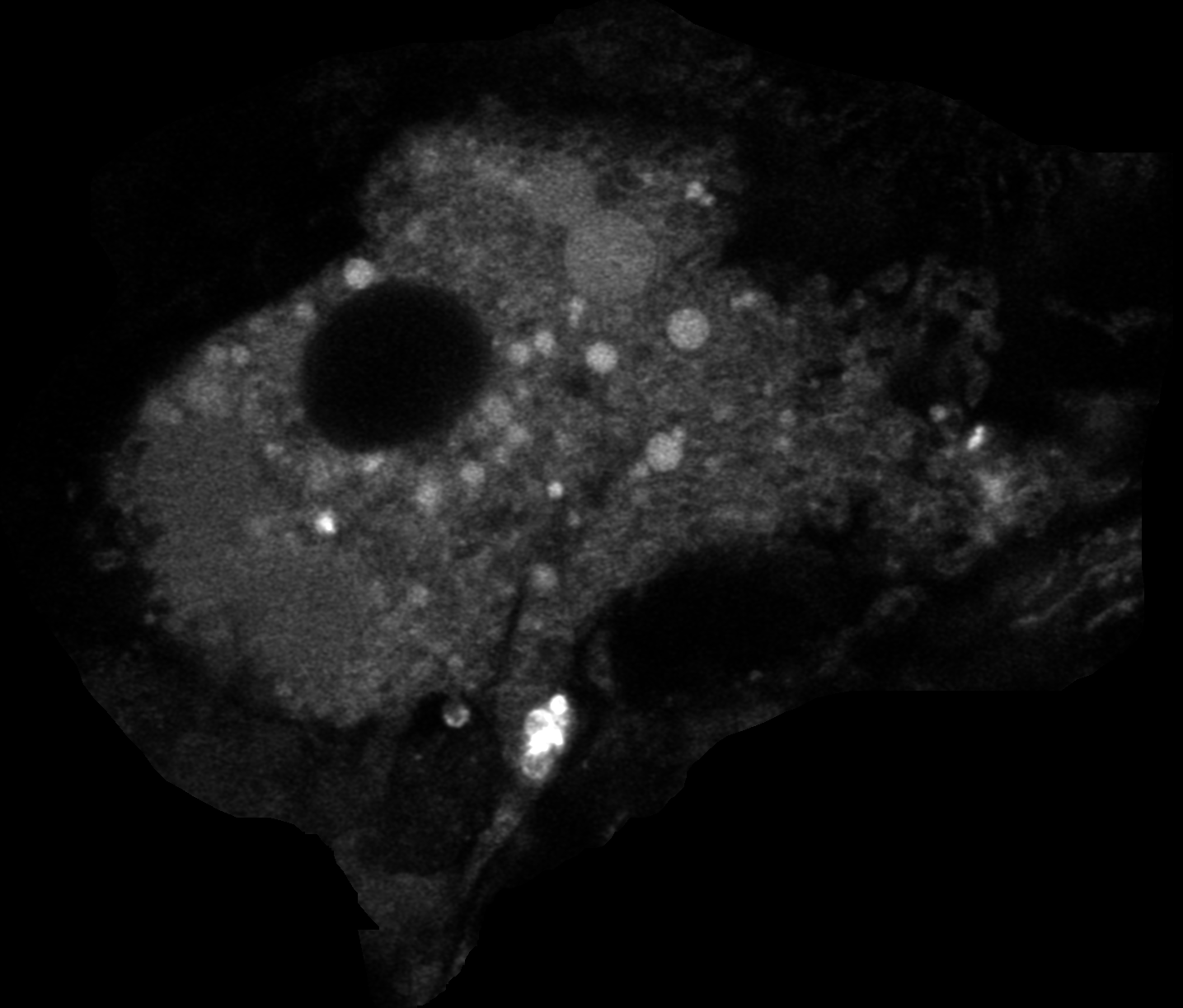

Supplement: Supplementary file 6 — Source data Fig. 4 [file 44318_2026_827_MOESM6_ESM.zip › Figure 4/Figure 4A/stim-1/NE-stim_timeseries_Out-0001.tif]

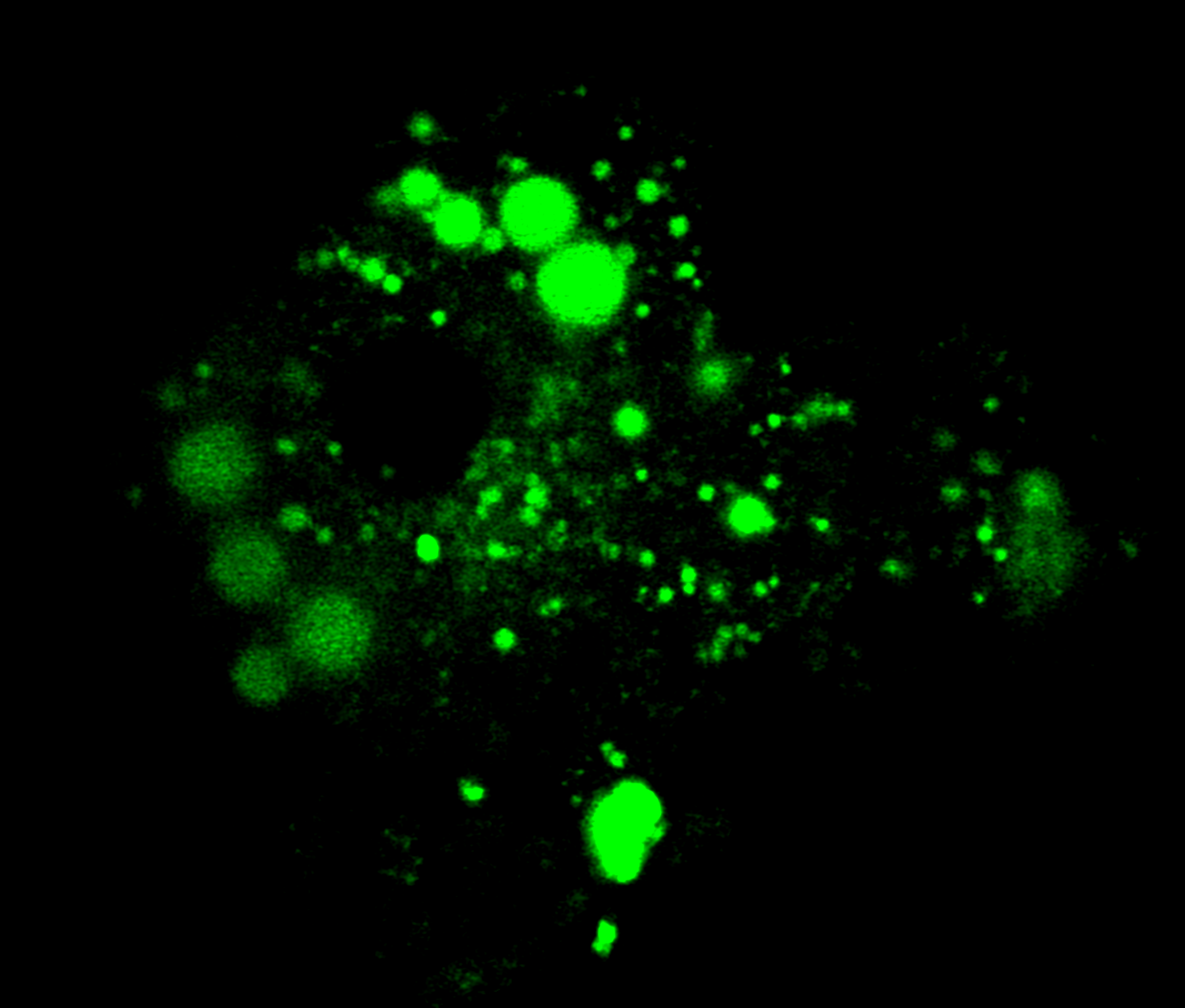

Supplement: Supplementary file 6 — Source data Fig. 4 [file 44318_2026_827_MOESM6_ESM.zip › Figure 4/Figure 4A/basal/baseline_Out-0001-rgb.tif]

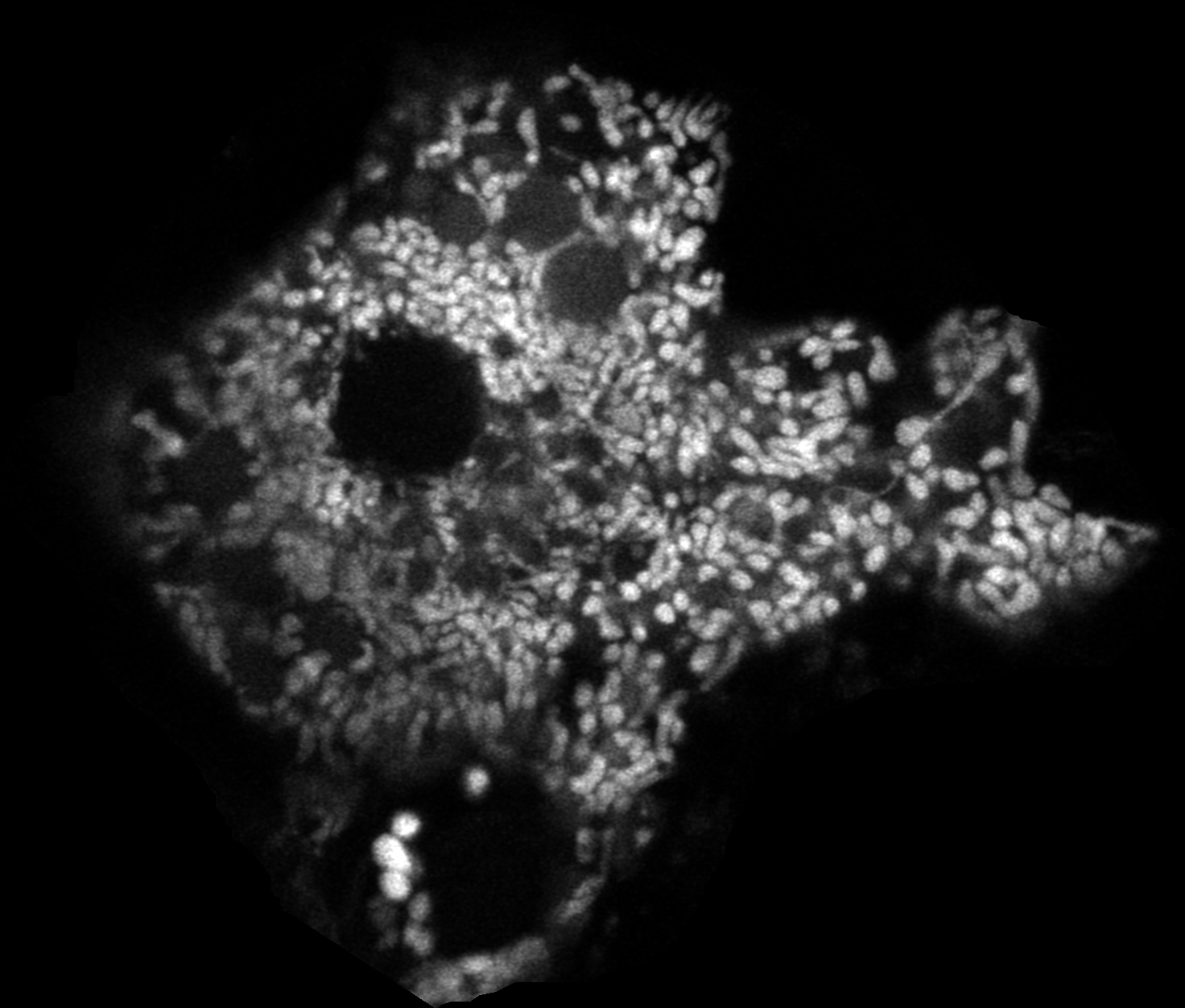

Supplement: Supplementary file 6 — Source data Fig. 4 [file 44318_2026_827_MOESM6_ESM.zip › Figure 4/Figure 4A/basal/baseline_Out-0002-scale-histogram.tif]

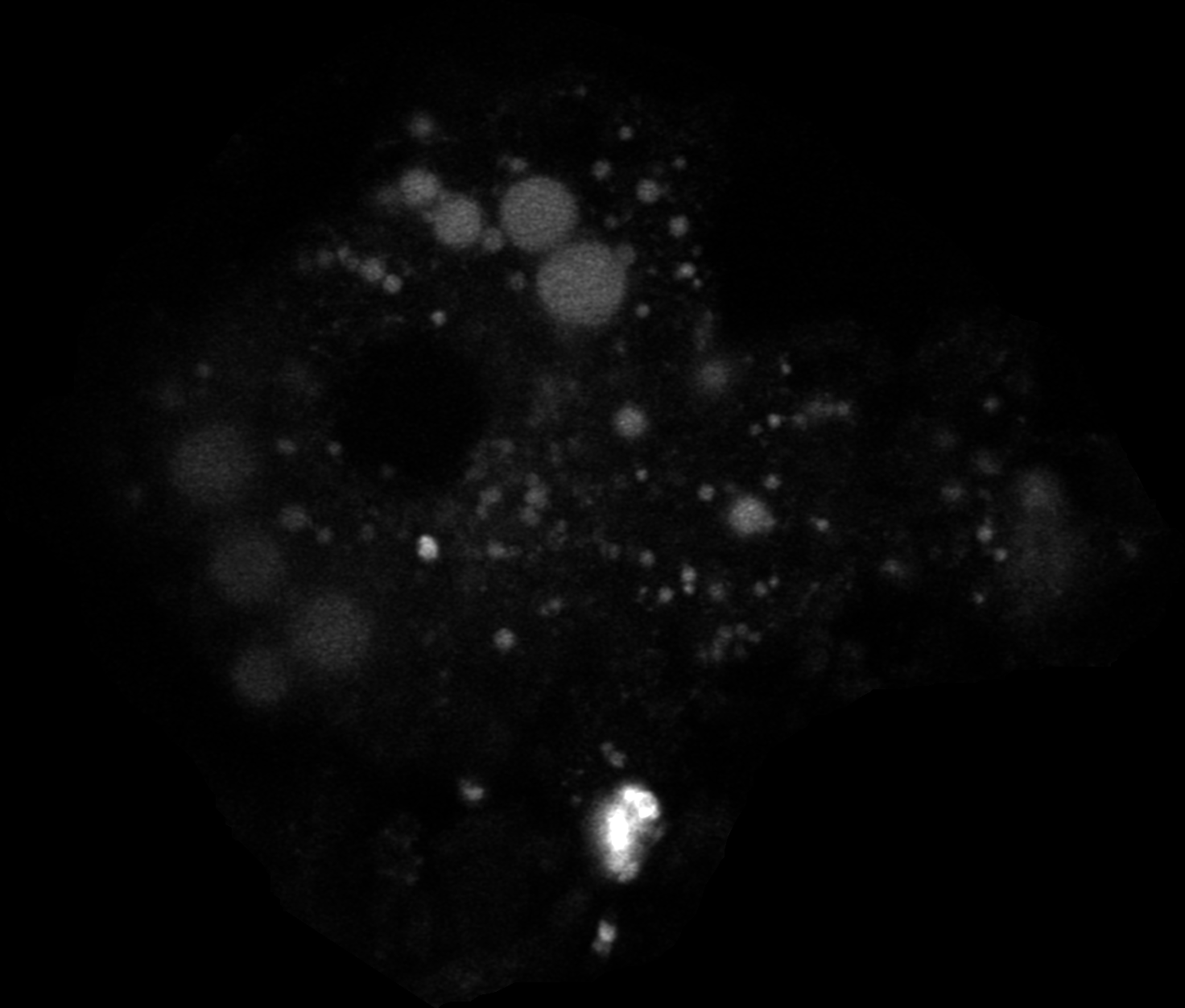

Supplement: Supplementary file 6 — Source data Fig. 4 [file 44318_2026_827_MOESM6_ESM.zip › Figure 4/Figure 4A/basal/baseline_Out-0001.tif]

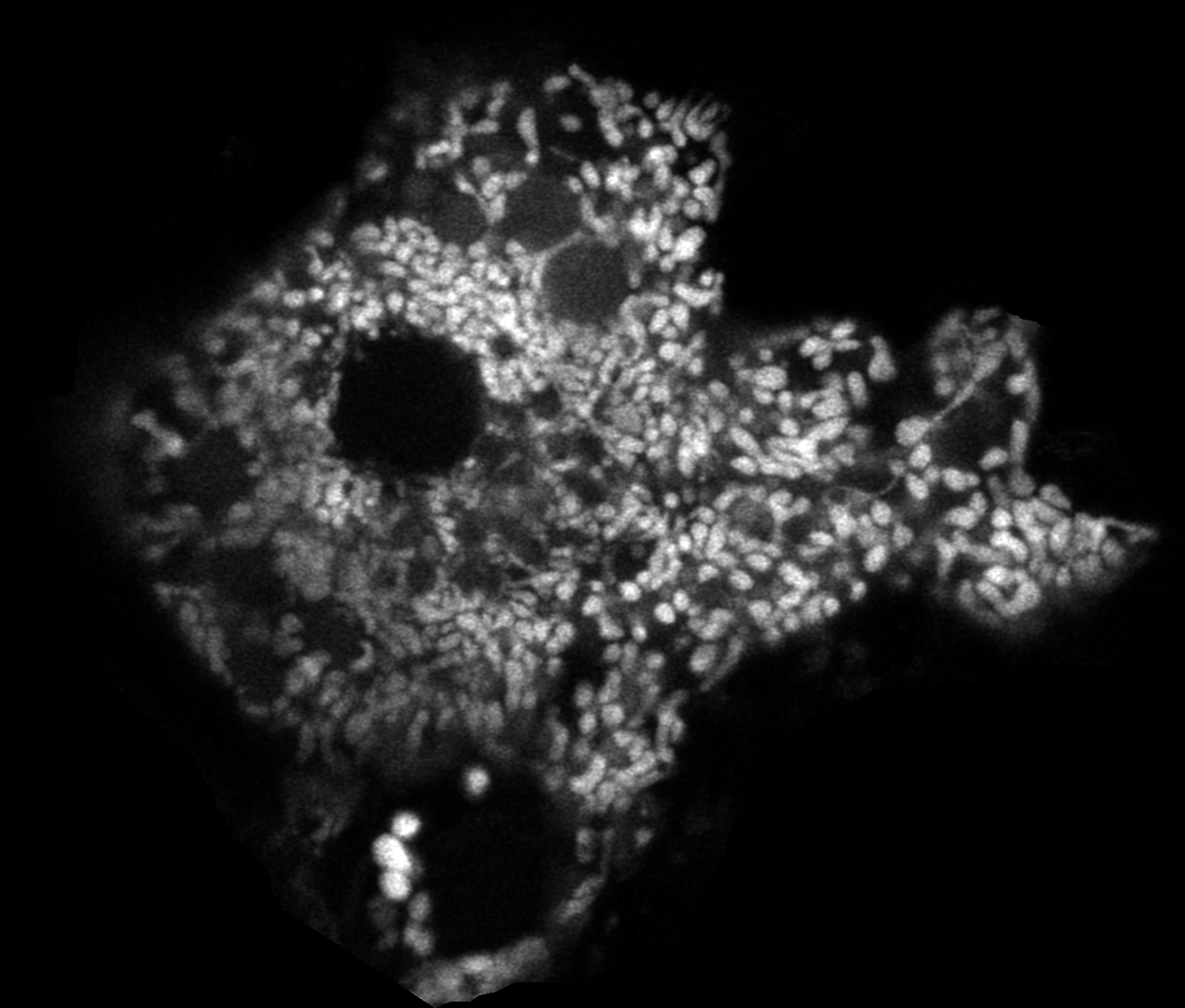

Supplement: Supplementary file 6 — Source data Fig. 4 [file 44318_2026_827_MOESM6_ESM.zip › Figure 4/Figure 4A/basal/baseline_Out-0002.tif]

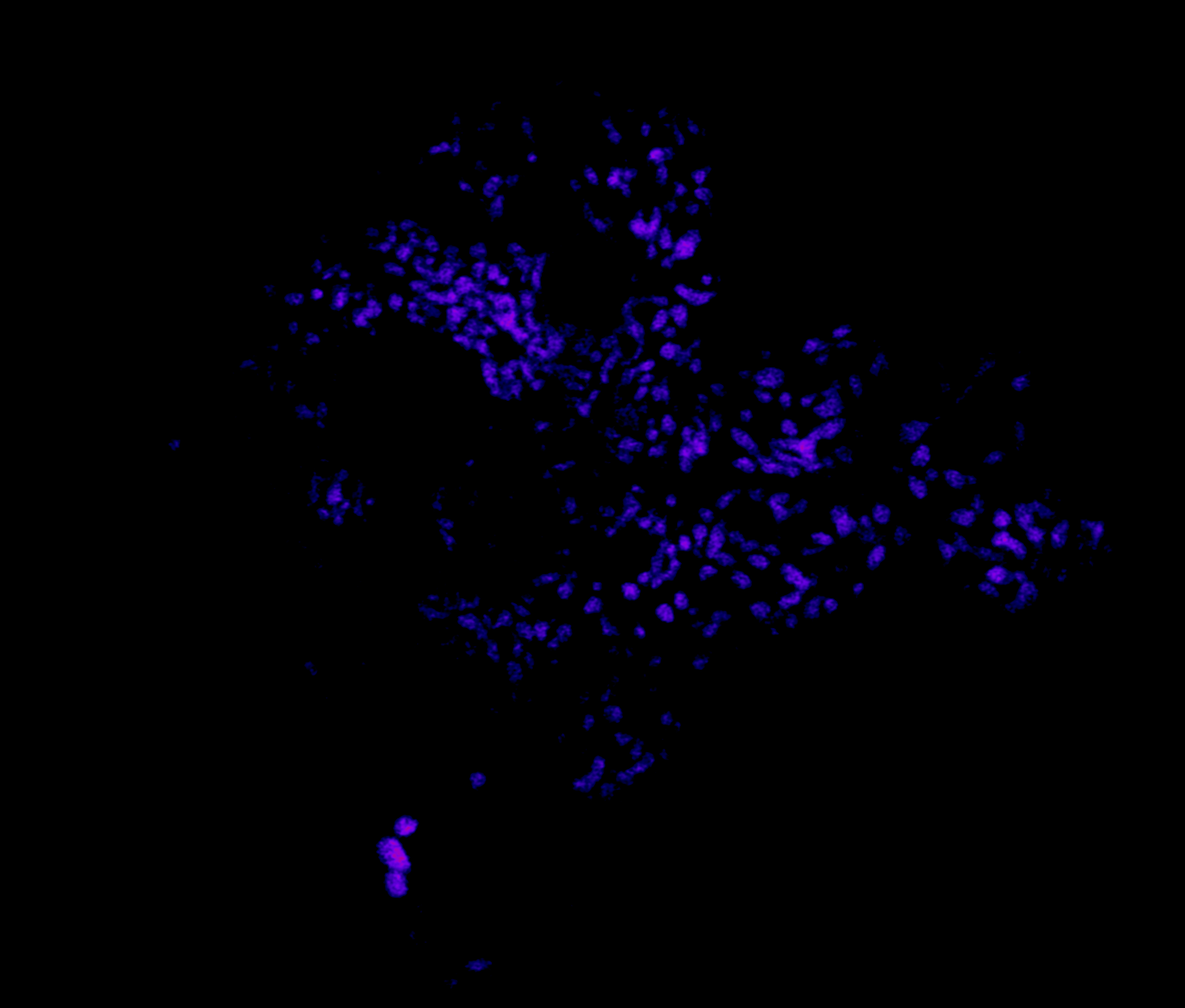

Supplement: Supplementary file 6 — Source data Fig. 4 [file 44318_2026_827_MOESM6_ESM.zip › Figure 4/Figure 4A/basal/baseline_Out-0002-scale-histogram-rgb.tif]

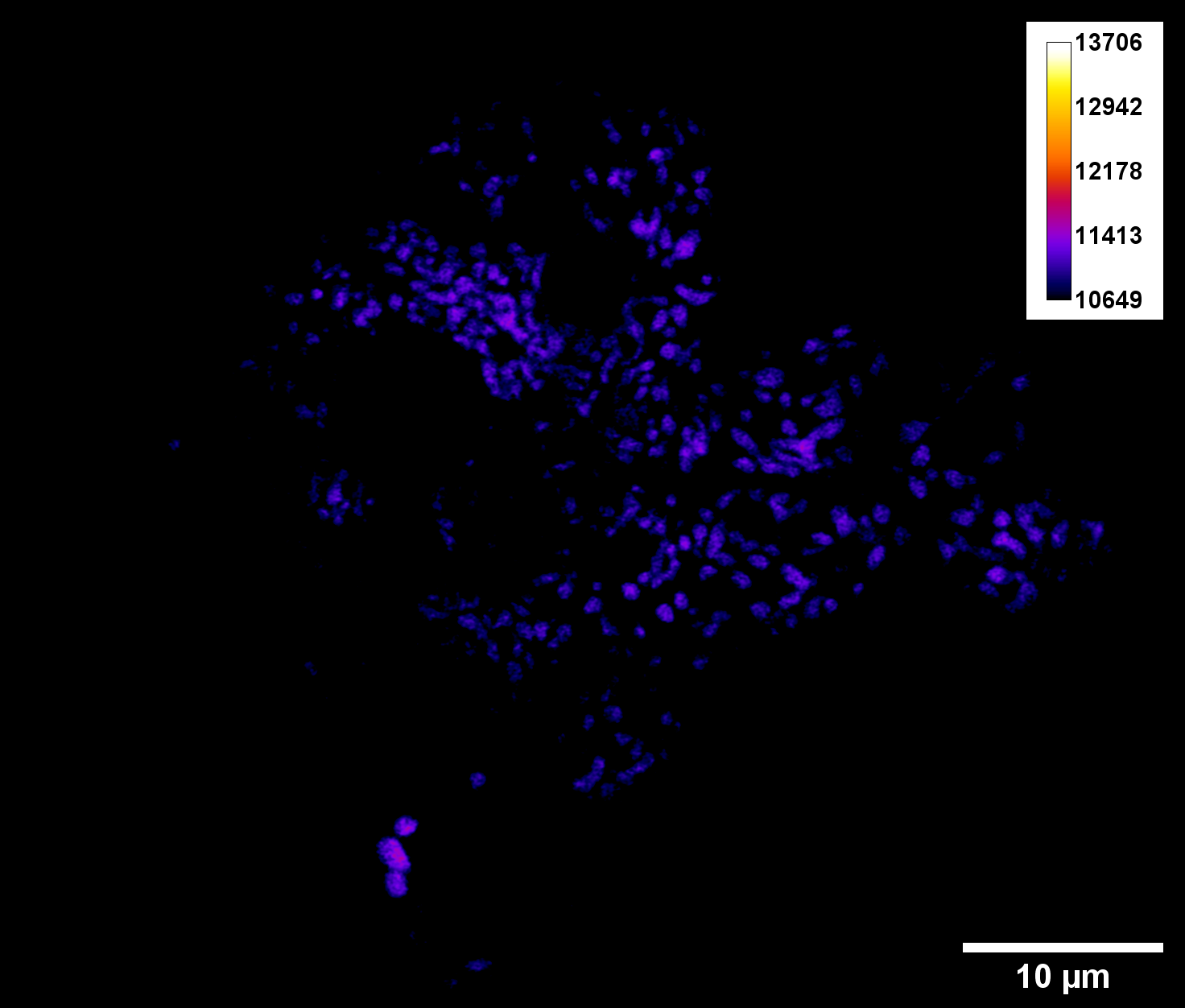

Supplement: Supplementary file 6 — Source data Fig. 4 [file 44318_2026_827_MOESM6_ESM.zip › Figure 4/Figure 4A/basal/baseline_Out-0002-scale-histogram-RGB-FL.tif]

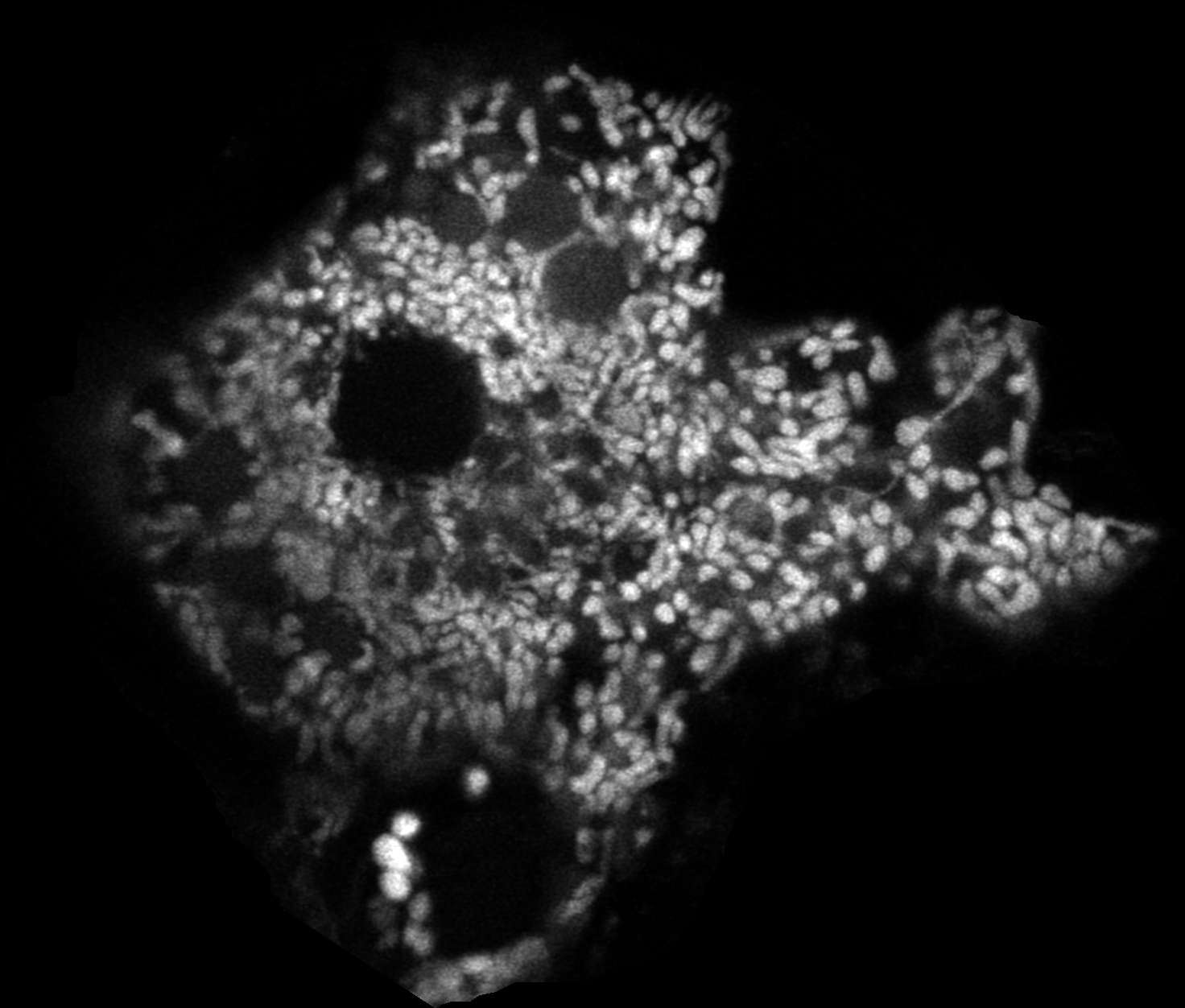

Supplement: Supplementary file 6 — Source data Fig. 4 [file 44318_2026_827_MOESM6_ESM.zip › Figure 4/Figure 4A/basal/baseline_Out-0002-scale-histogram-.tif]

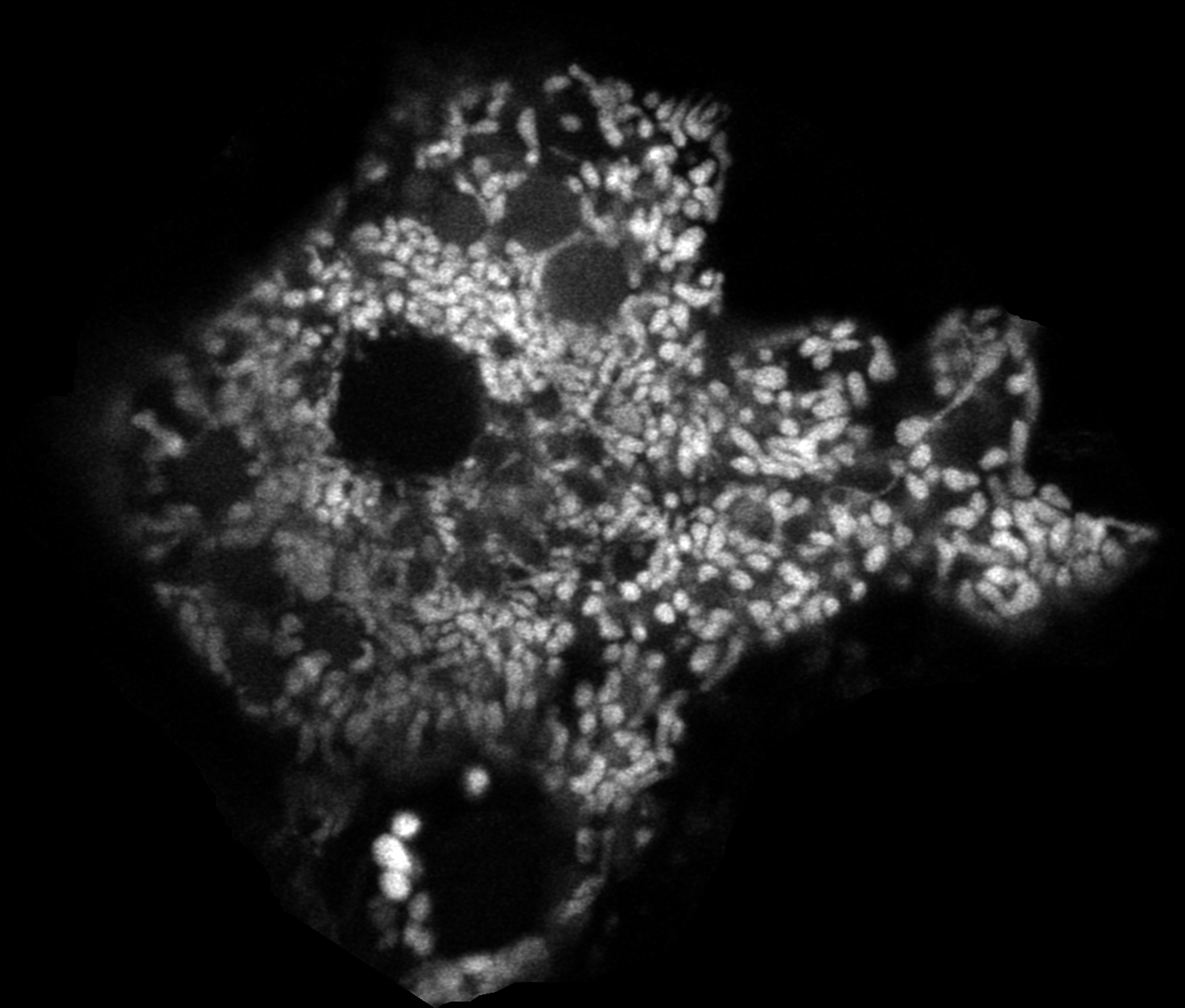

Supplement: Supplementary file 6 — Source data Fig. 4 [file 44318_2026_827_MOESM6_ESM.zip › Figure 4/Figure 4A/basal/baseline_Out-0002-scale.tif]

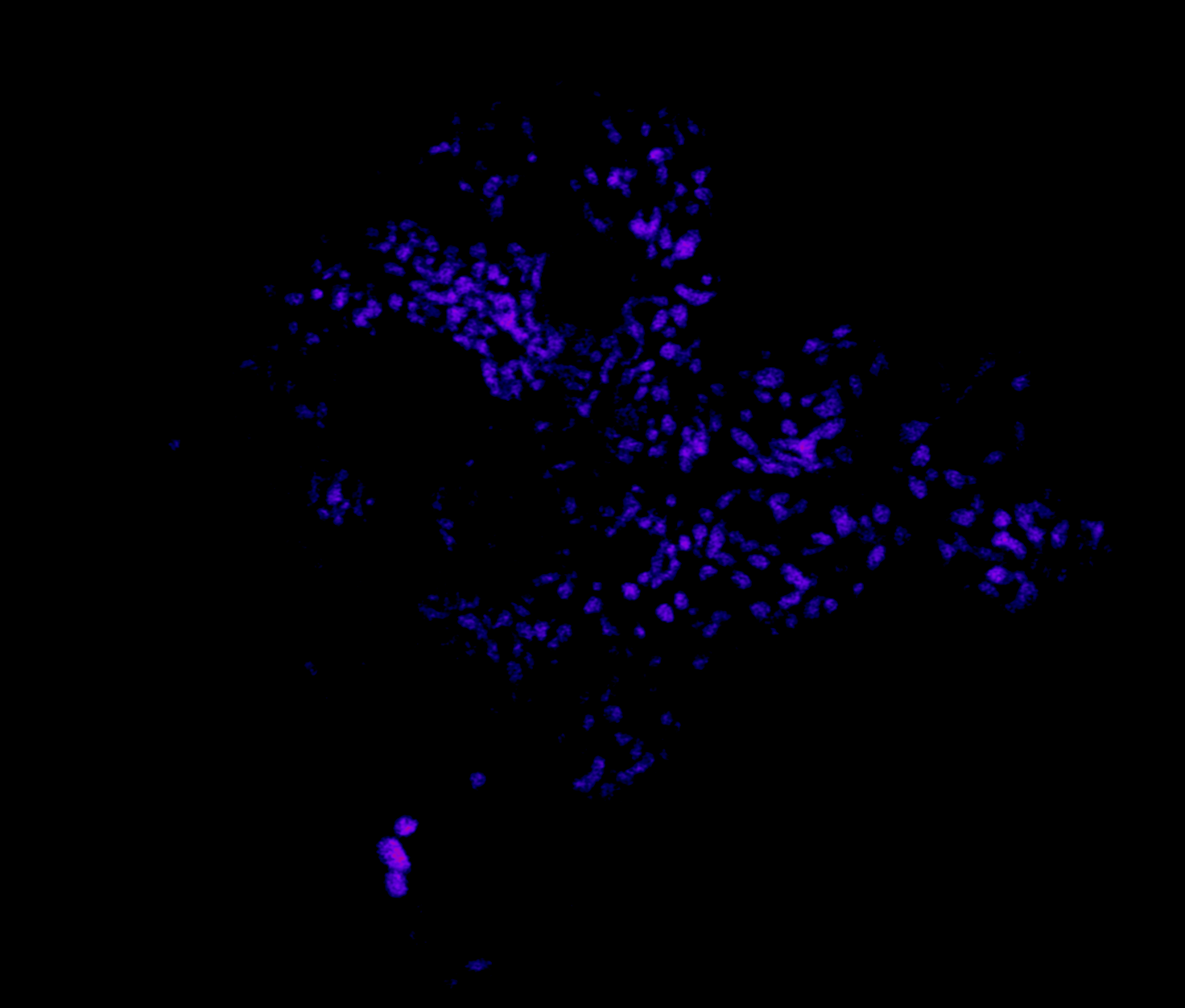

Supplement: Supplementary file 6 — Source data Fig. 4 [file 44318_2026_827_MOESM6_ESM.zip › Figure 4/Figure 4A/basal/baseline_Out-0002-scale-rgb.tif]

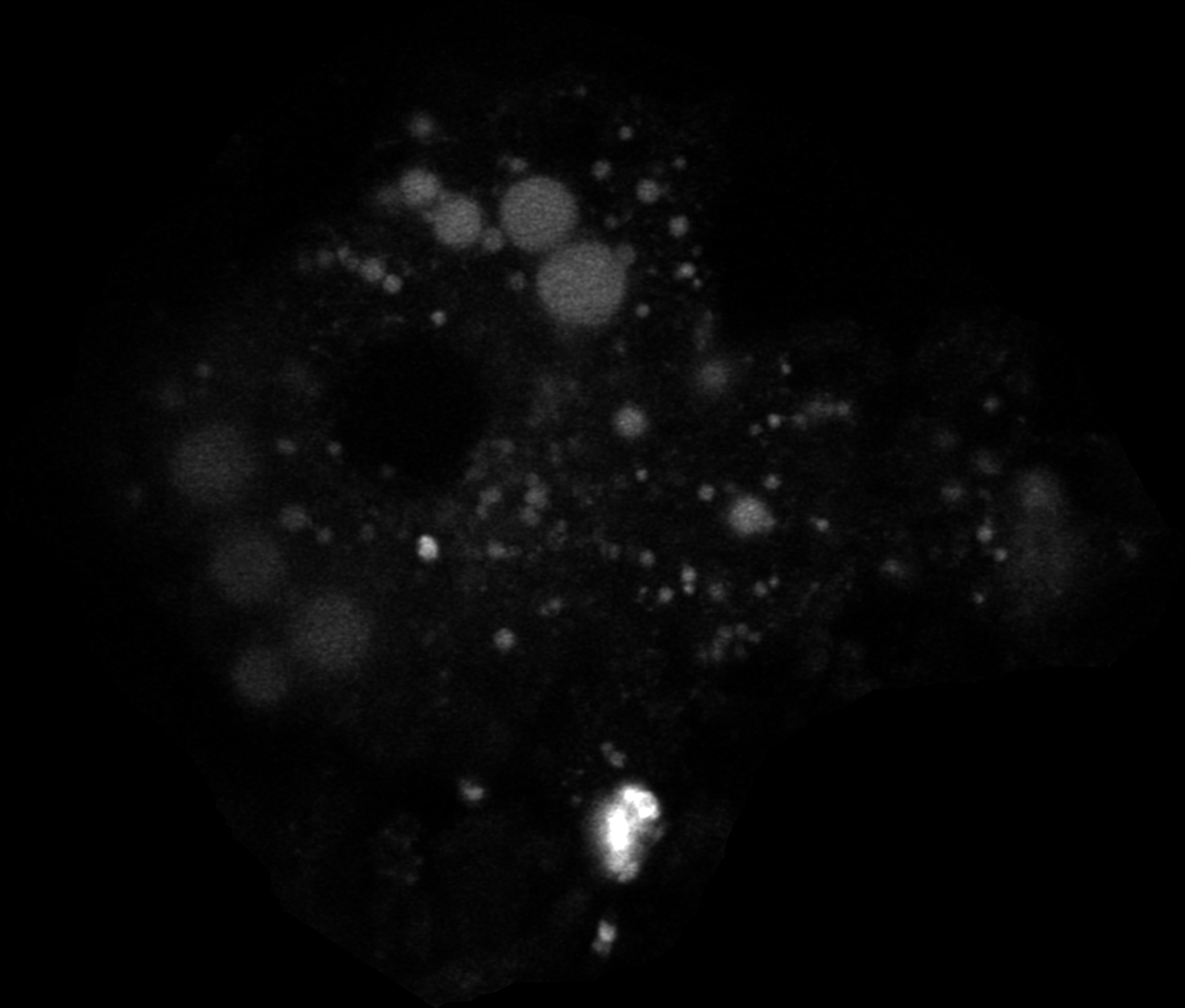

Supplement: Supplementary file 6 — Source data Fig. 4 [file 44318_2026_827_MOESM6_ESM.zip › Figure 4/Figure 4A/basal/Composite-tif.tif]

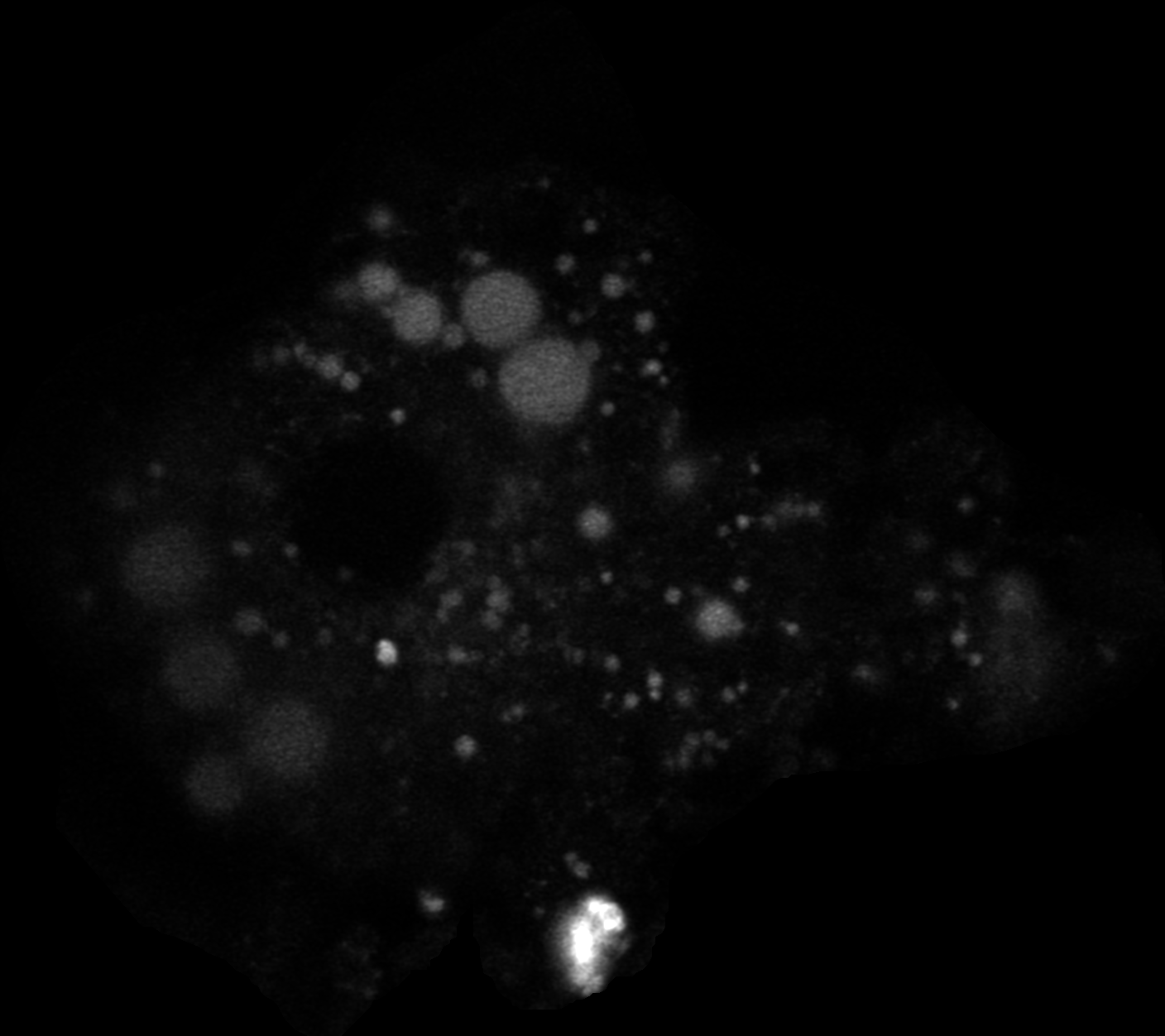

Supplement: Supplementary file 6 — Source data Fig. 4 [file 44318_2026_827_MOESM6_ESM.zip › Figure 4/Figure 4A/basal/baseline_Out.czi - baseline #7-bodipy-1.tif]

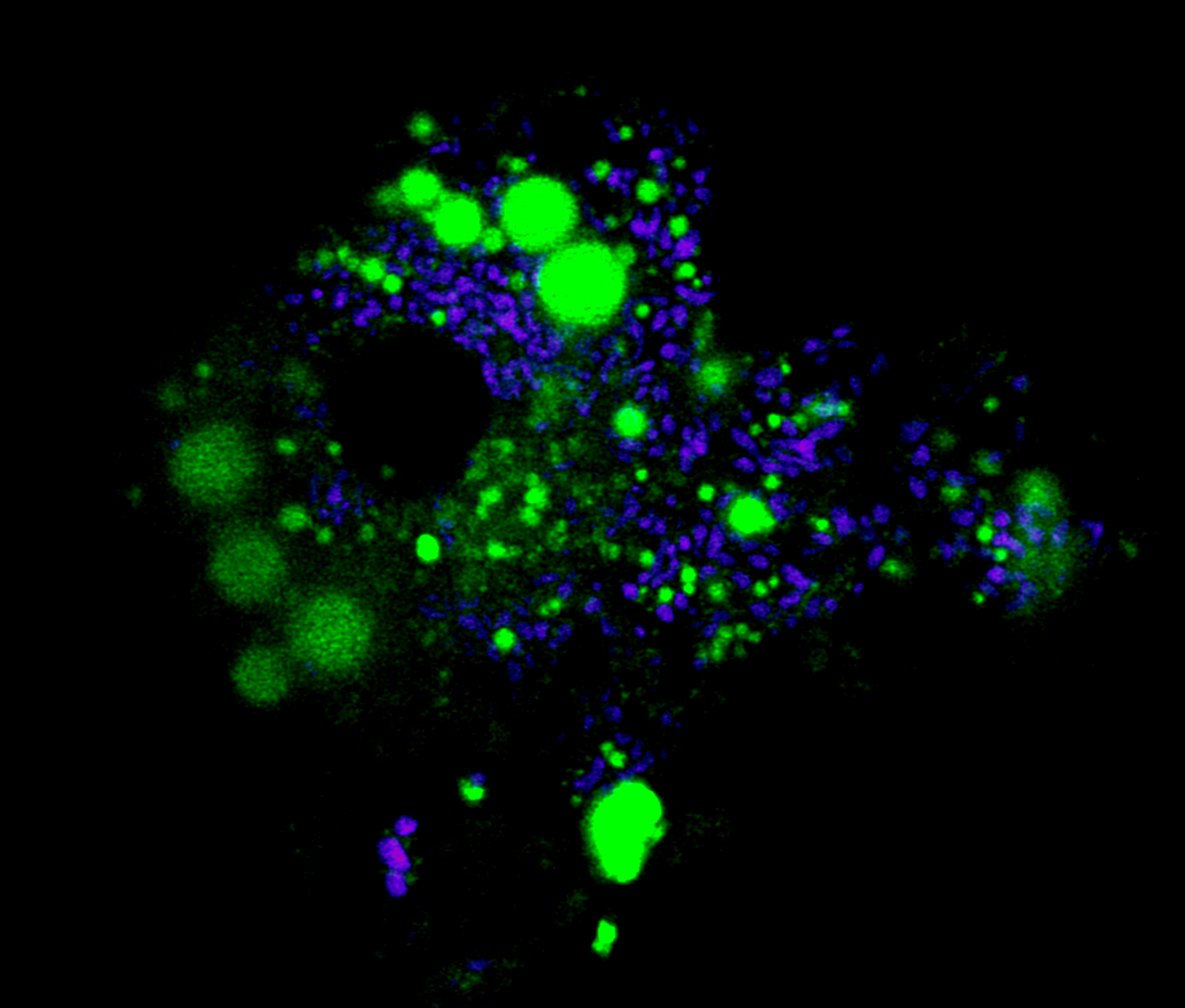

Supplement: Supplementary file 6 — Source data Fig. 4 [file 44318_2026_827_MOESM6_ESM.zip › Figure 4/Figure 4A/basal/Composite (RGB).tif]

## Slide 1
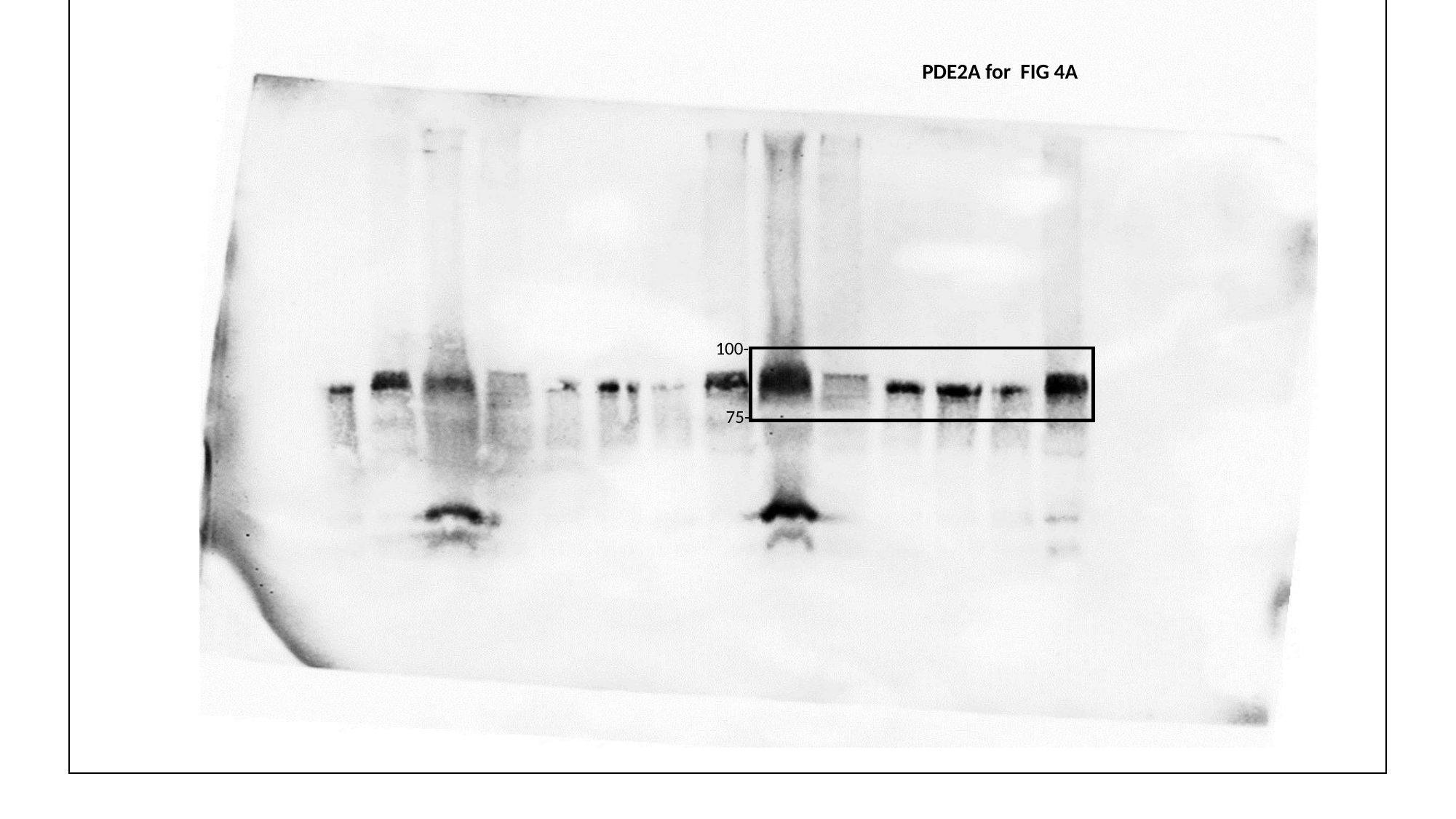

PDE2A for FIG 4A
100-
75-

## Slide 2
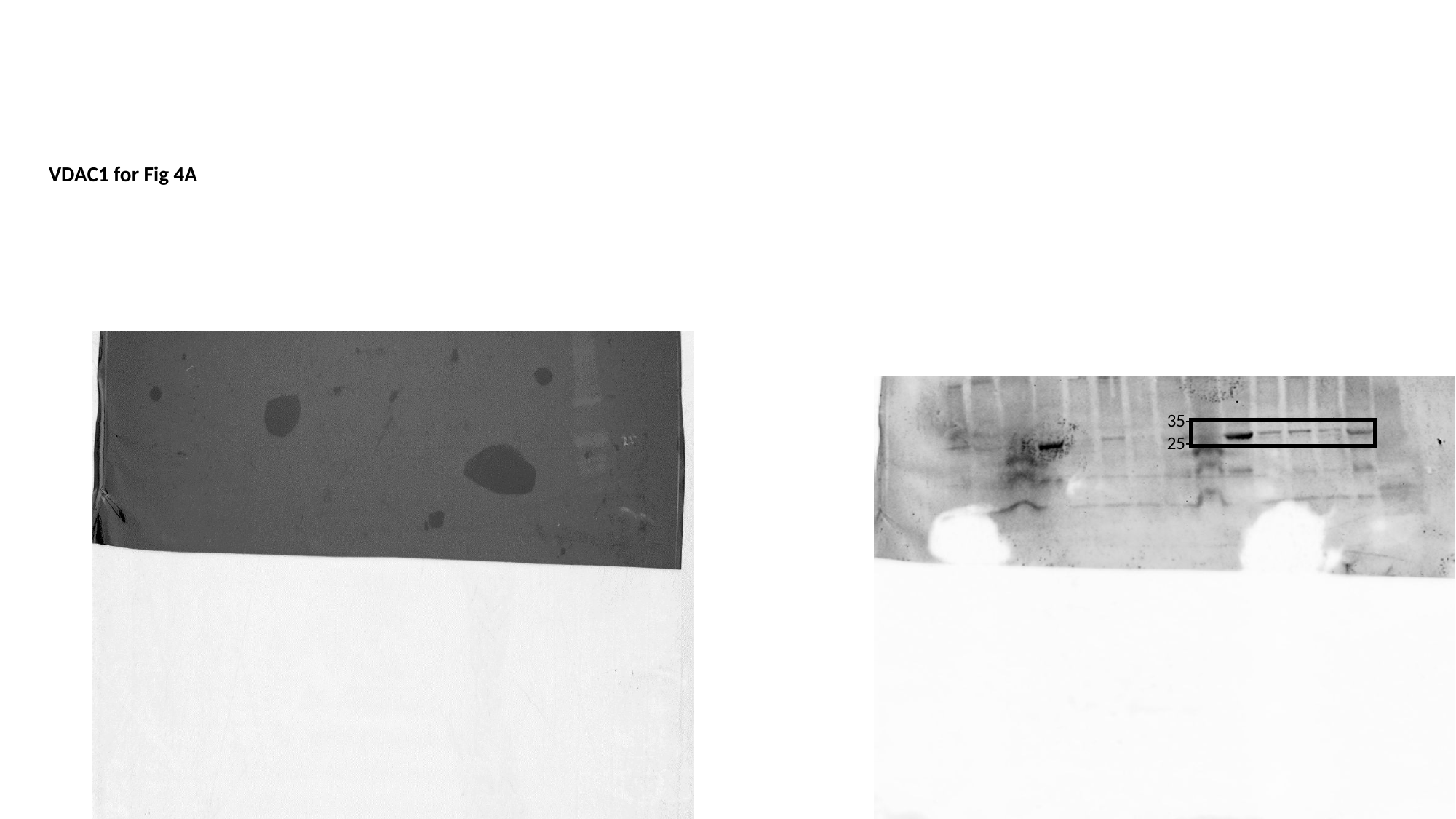

#
VDAC1 for Fig 4A
35-
25-

## Slide 3
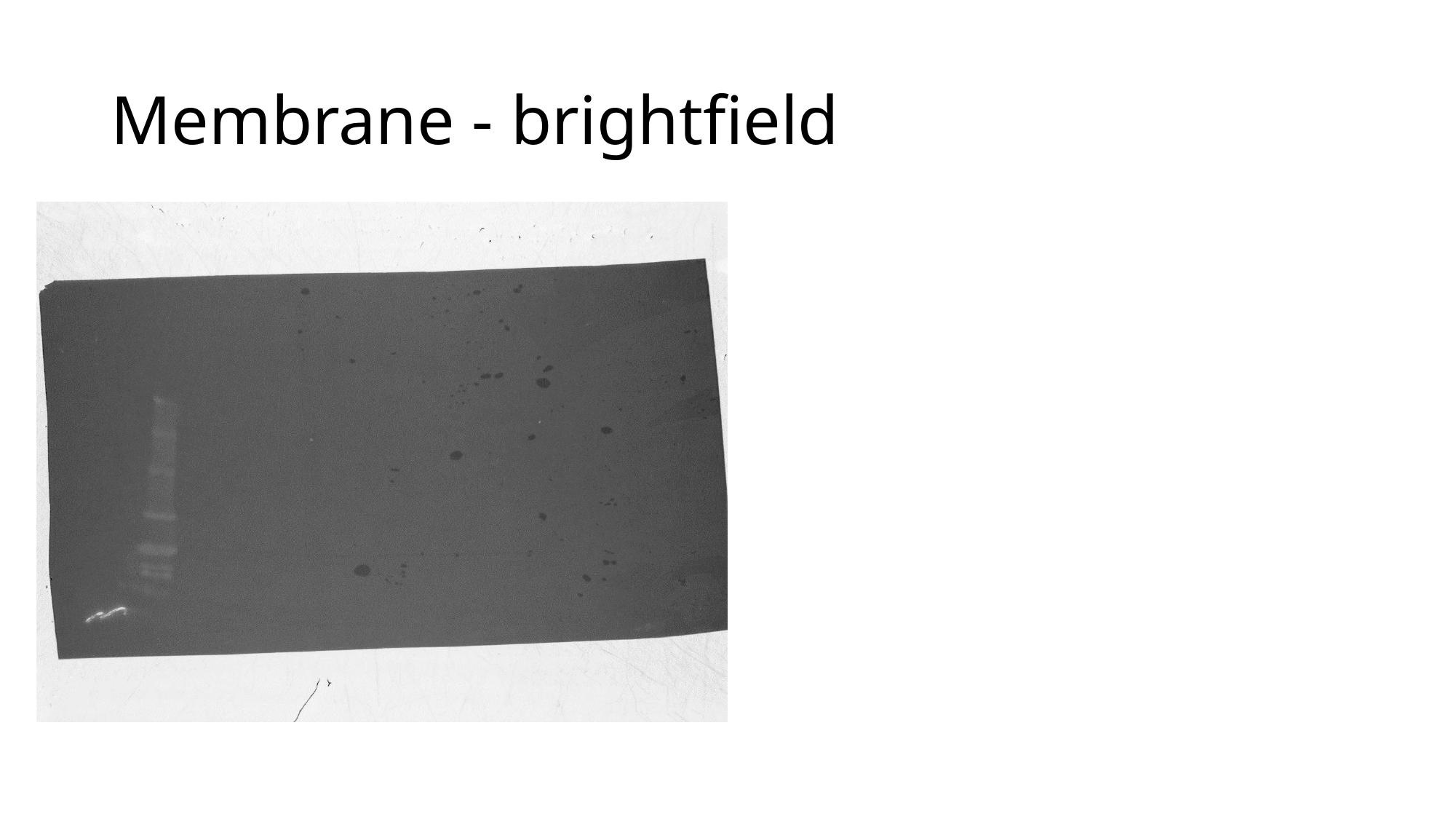

# Membrane - brightfield

Supplement: Supplementary file 7 — Source data Fig. 5 [file 44318_2026_827_MOESM7_ESM.zip › Figure 5/Figure 5A blot.pptx]

## Slide 1
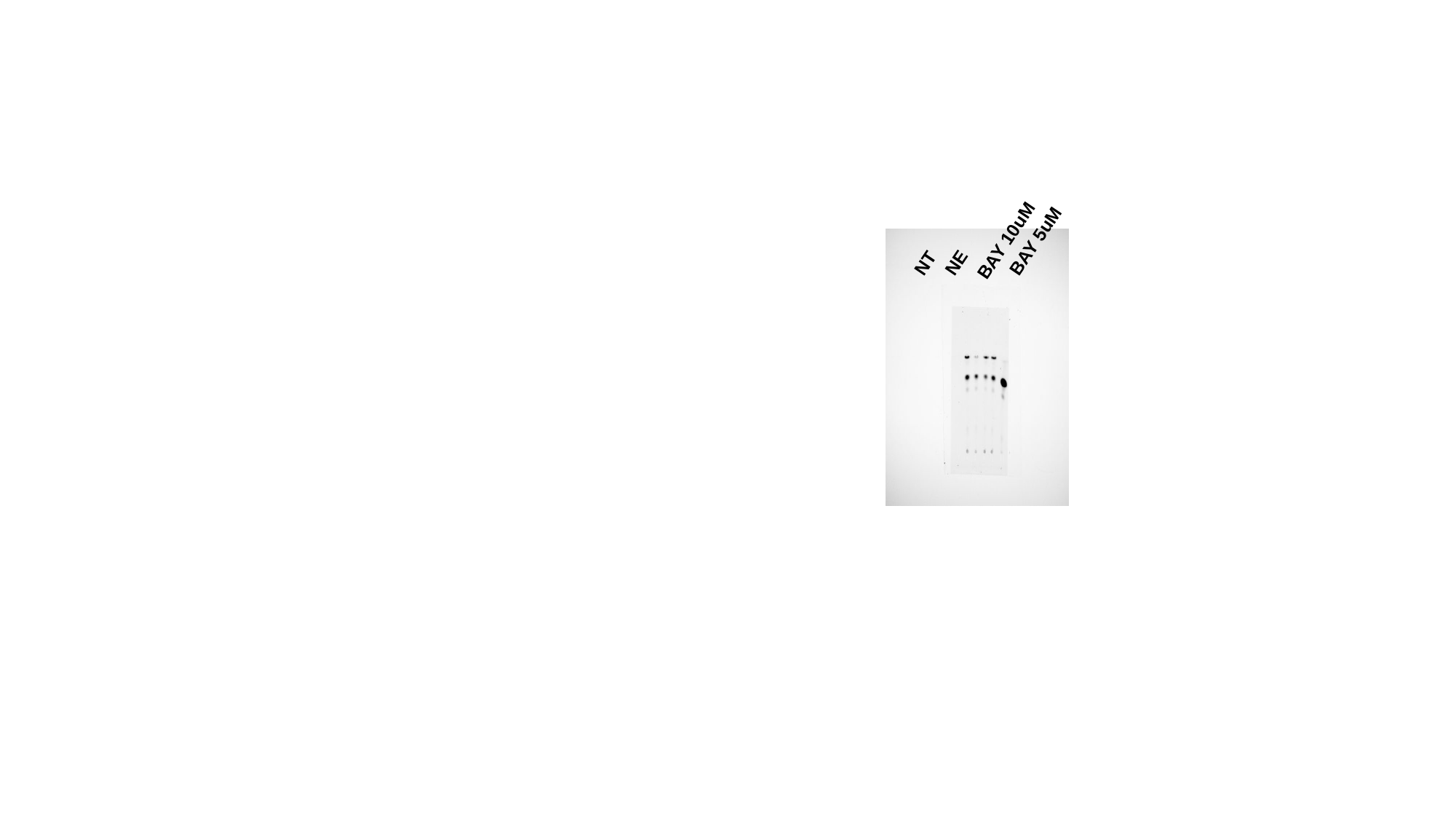

BAY 10uM
BAY 5uM
NE
NT

Supplement: Supplementary file 7 — Source data Fig. 5 [file 44318_2026_827_MOESM7_ESM.zip › Figure 5/Figure 5L TLC.pptx]

## Slide 1
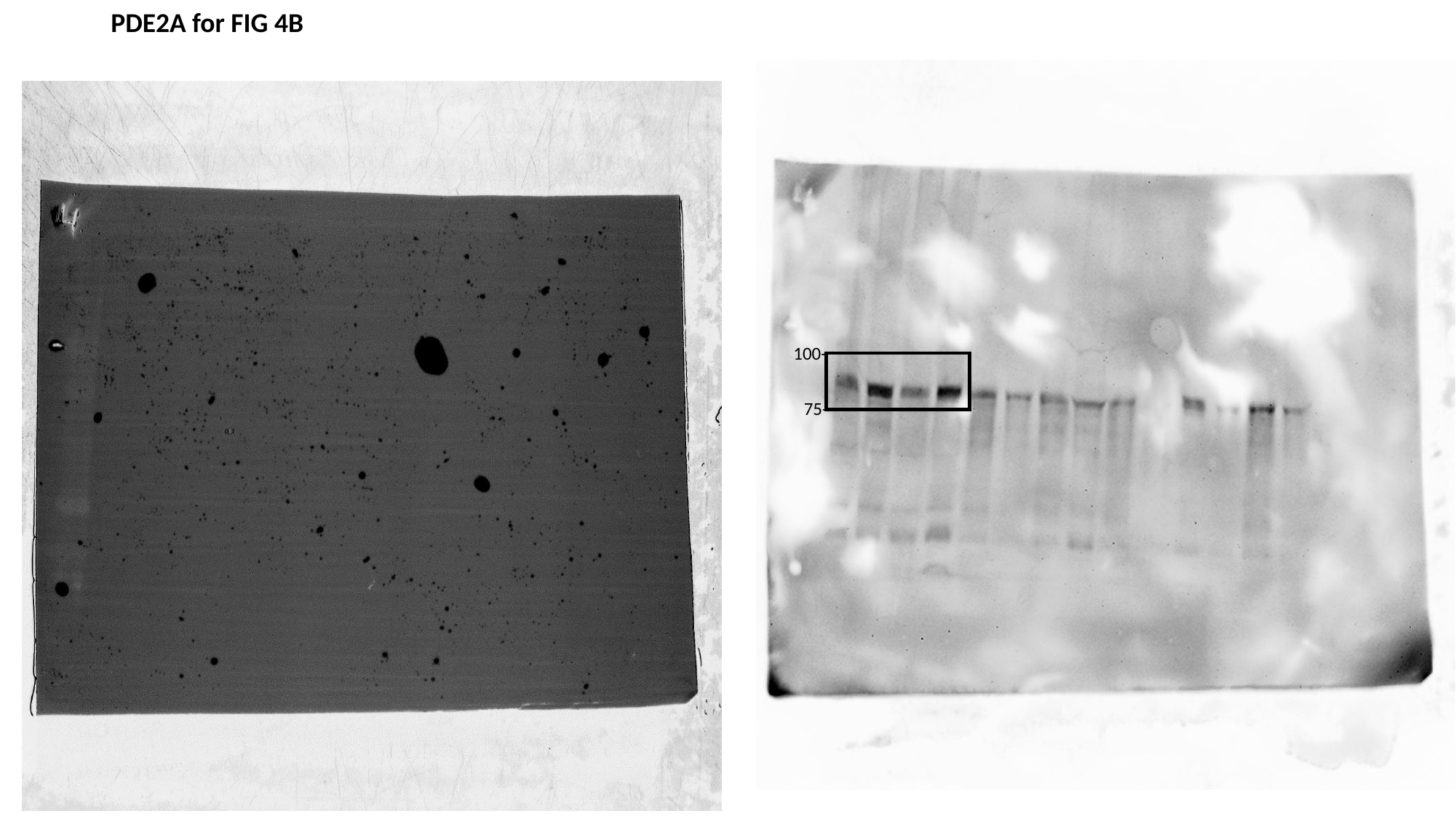

PDE2A for FIG 4B
#
100-
75-

## Slide 2
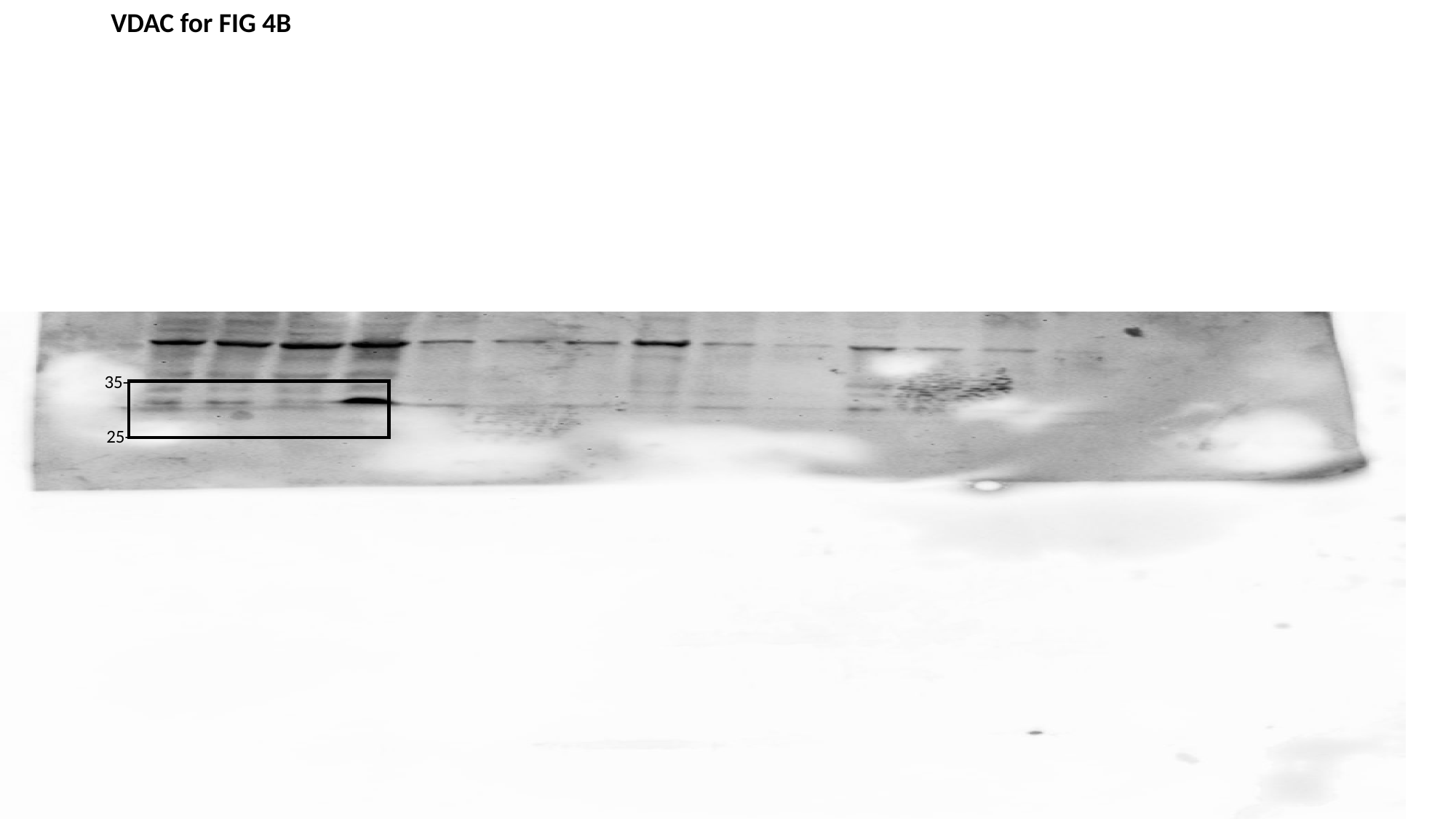

VDAC for FIG 4B
 35-
25-

## Slide 3
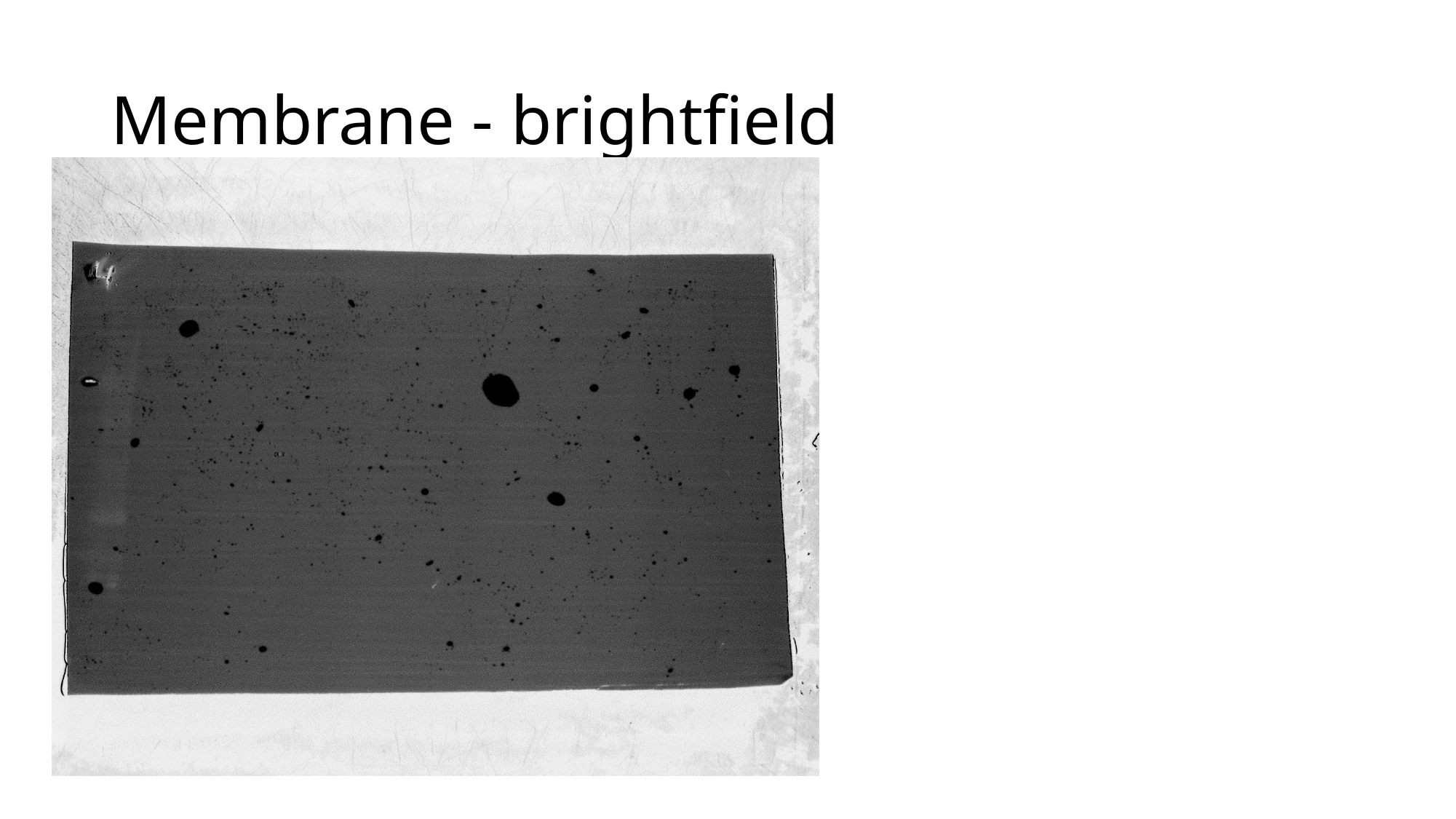

# Membrane - brightfield

Supplement: Supplementary file 7 — Source data Fig. 5 [file 44318_2026_827_MOESM7_ESM.zip › Figure 5/Figure 5B blot.pptx]

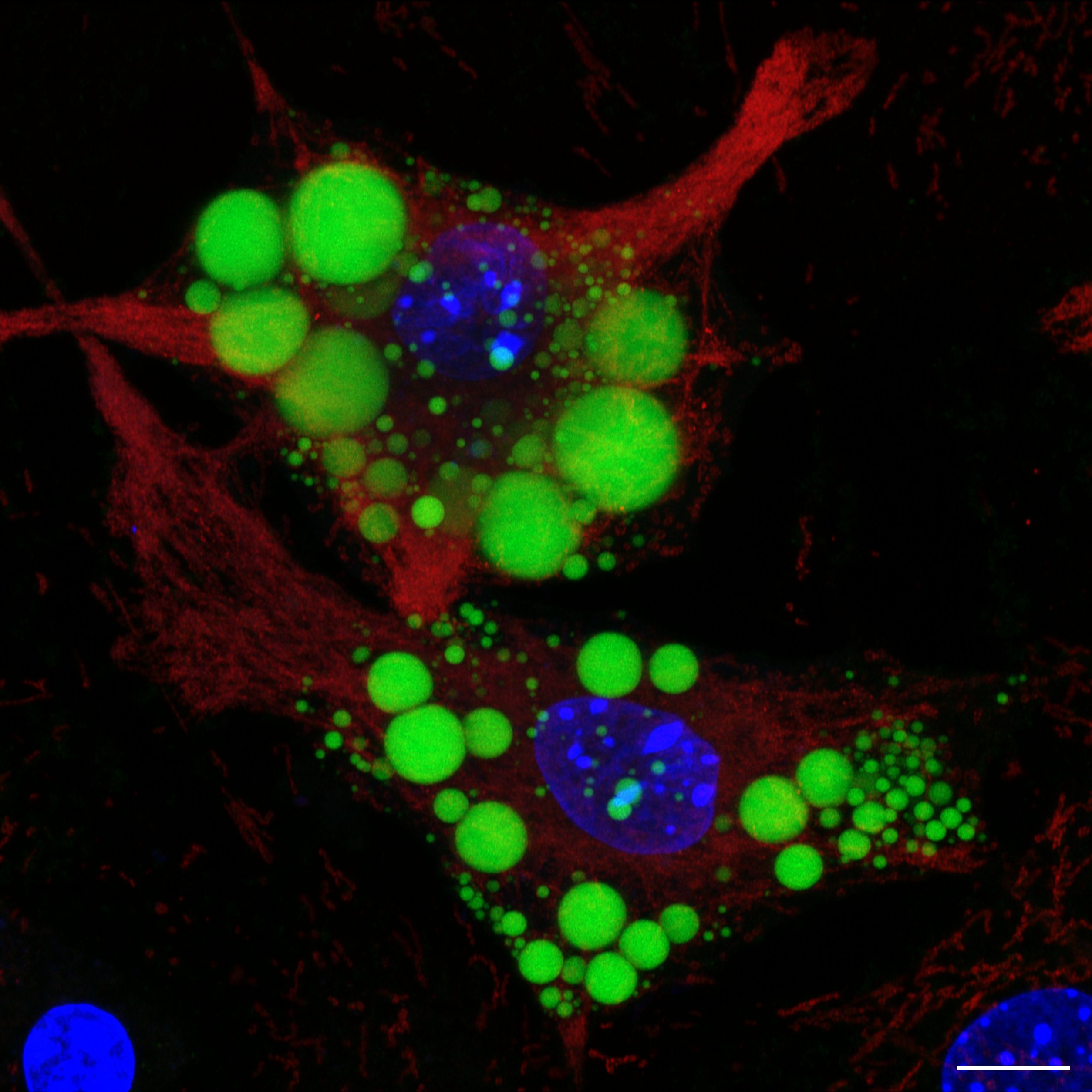

Supplement: Supplementary file 7 — Source data Fig. 5 [file 44318_2026_827_MOESM7_ESM.zip › Figure 5/Figure 5I/BAY60 Image 45_Q1_Out.jpg]

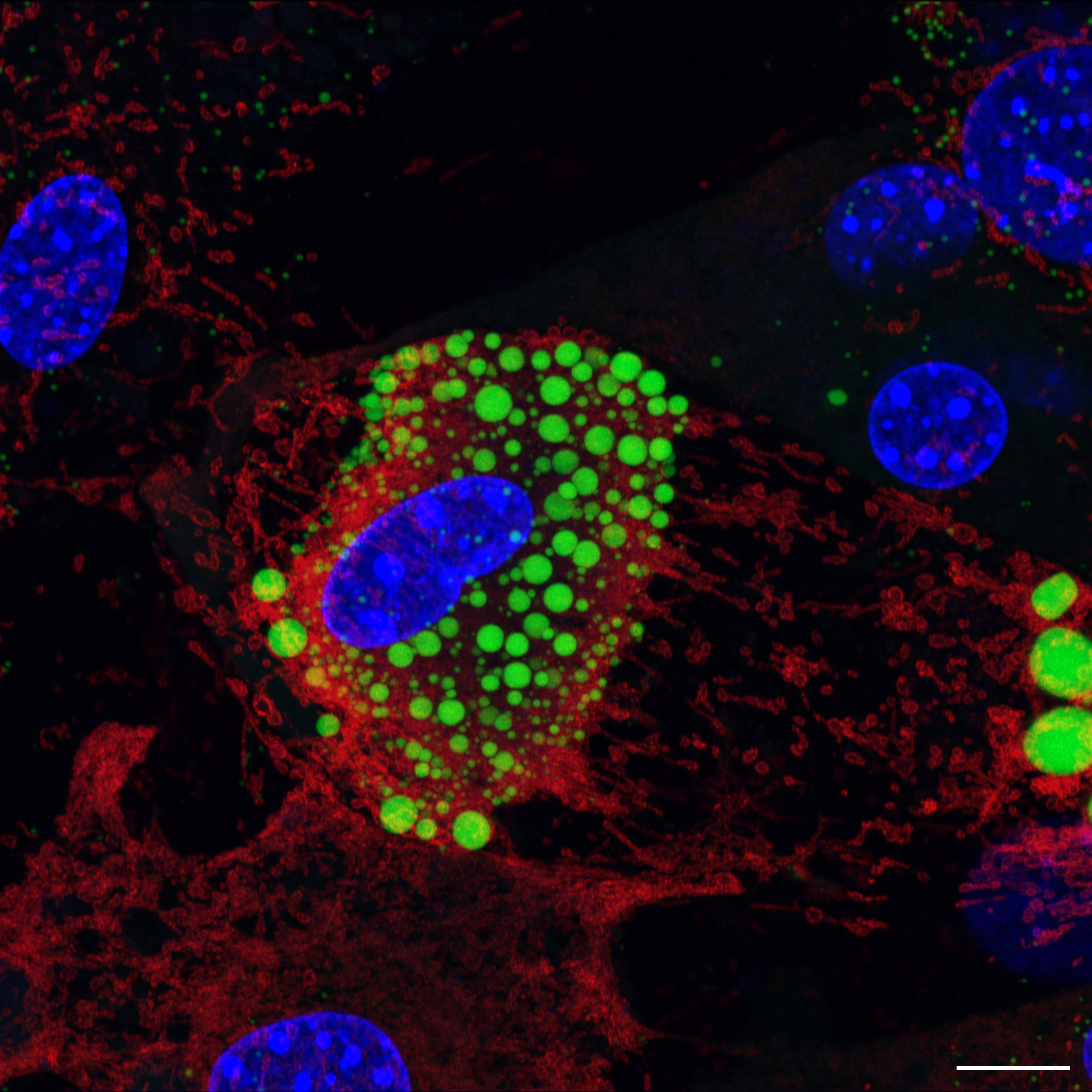

Supplement: Supplementary file 7 — Source data Fig. 5 [file 44318_2026_827_MOESM7_ESM.zip › Figure 5/Figure 5I/NE Image 26_Out.jpg]

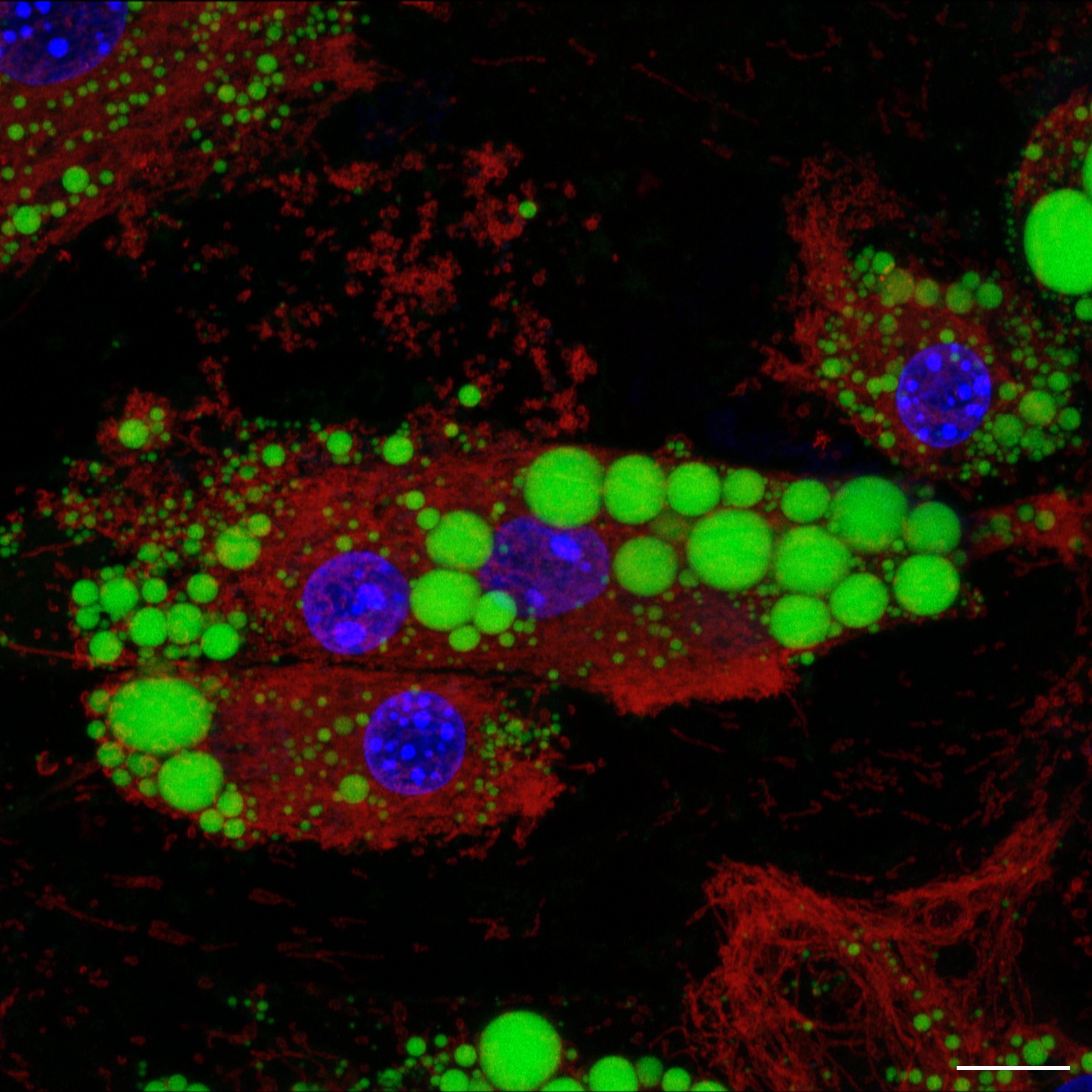

Supplement: Supplementary file 7 — Source data Fig. 5 [file 44318_2026_827_MOESM7_ESM.zip › Figure 5/Figure 5I/unt Image1_Out.jpg]

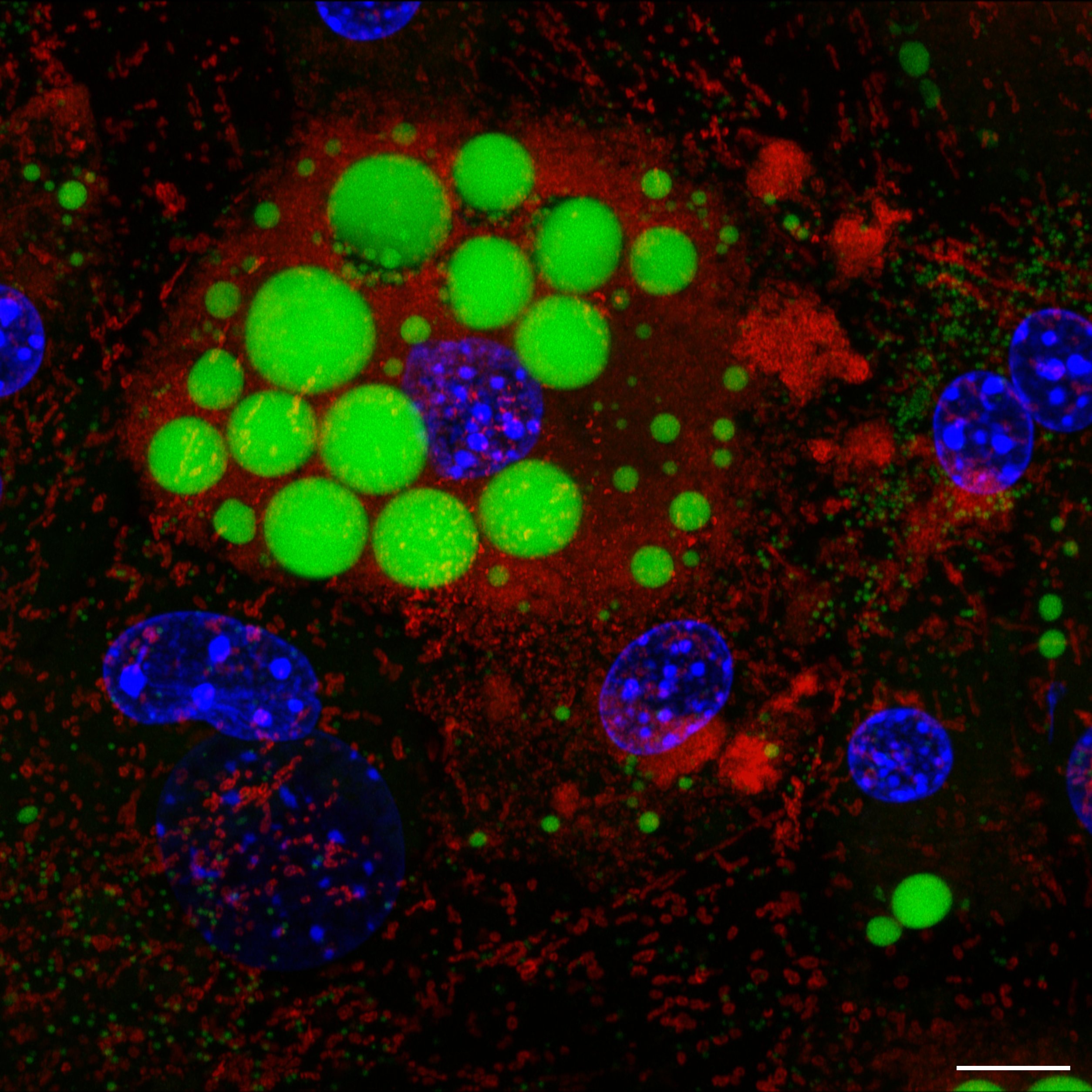

Supplement: Supplementary file 7 — Source data Fig. 5 [file 44318_2026_827_MOESM7_ESM.zip › Figure 5/Figure 5I/BAY60 NE Image 60_Out.jpg]

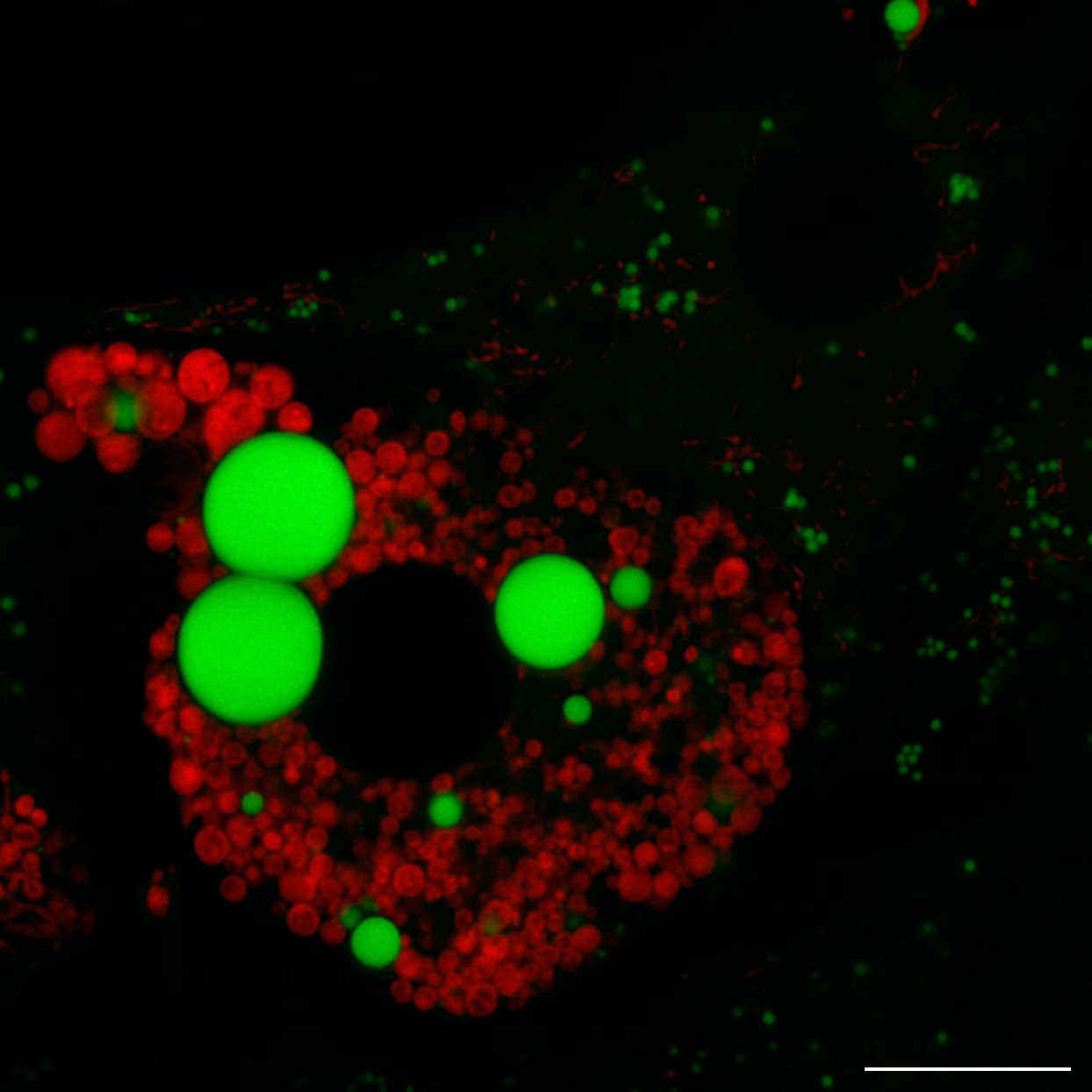

Supplement: Supplementary file 7 — Source data Fig. 5 [file 44318_2026_827_MOESM7_ESM.zip › Figure 5/Figure 5G/unt NE_Out.czi - nt #2.jpg]

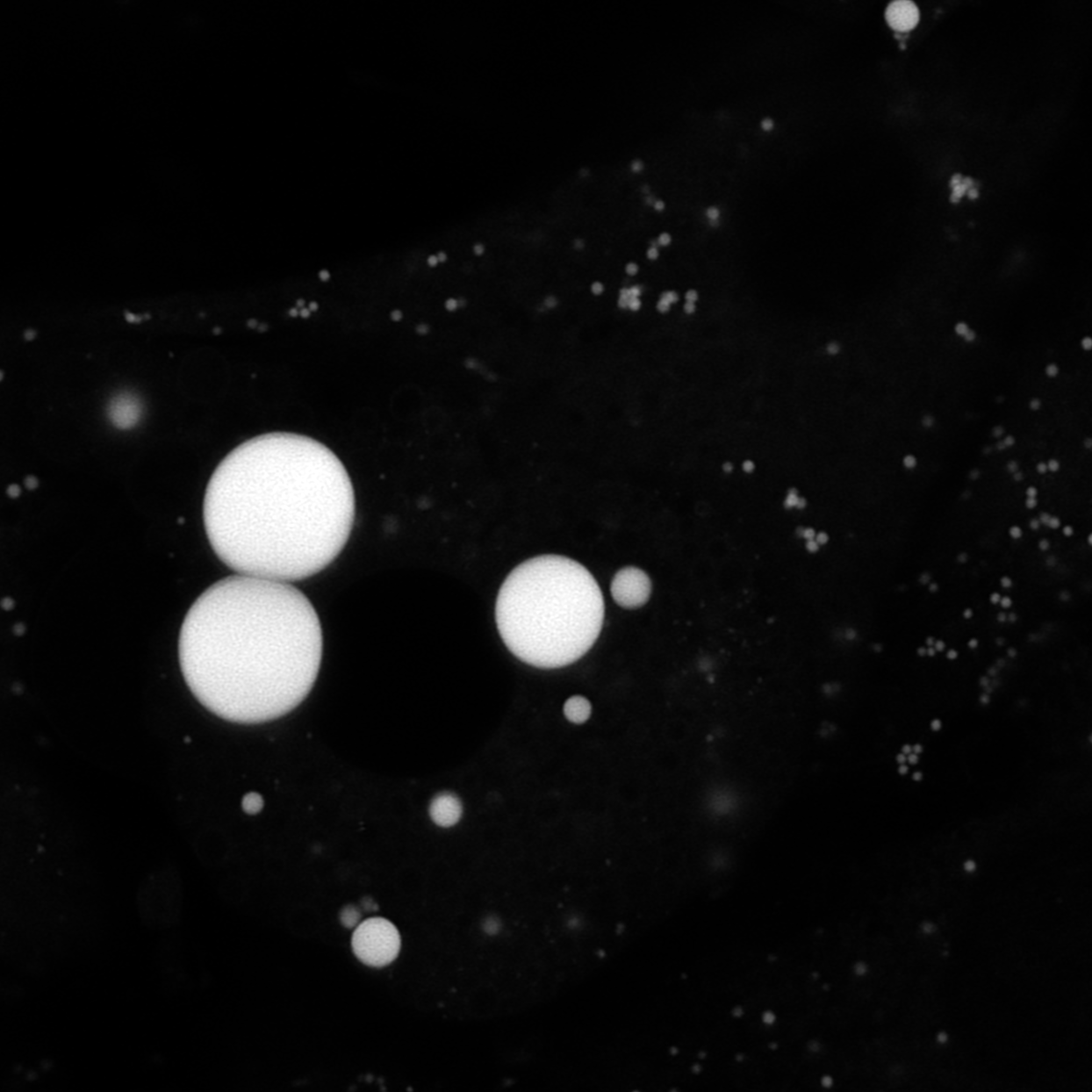

Supplement: Supplementary file 7 — Source data Fig. 5 [file 44318_2026_827_MOESM7_ESM.zip › Figure 5/Figure 5G/unt NE_Out.czi - nt #2.tif]

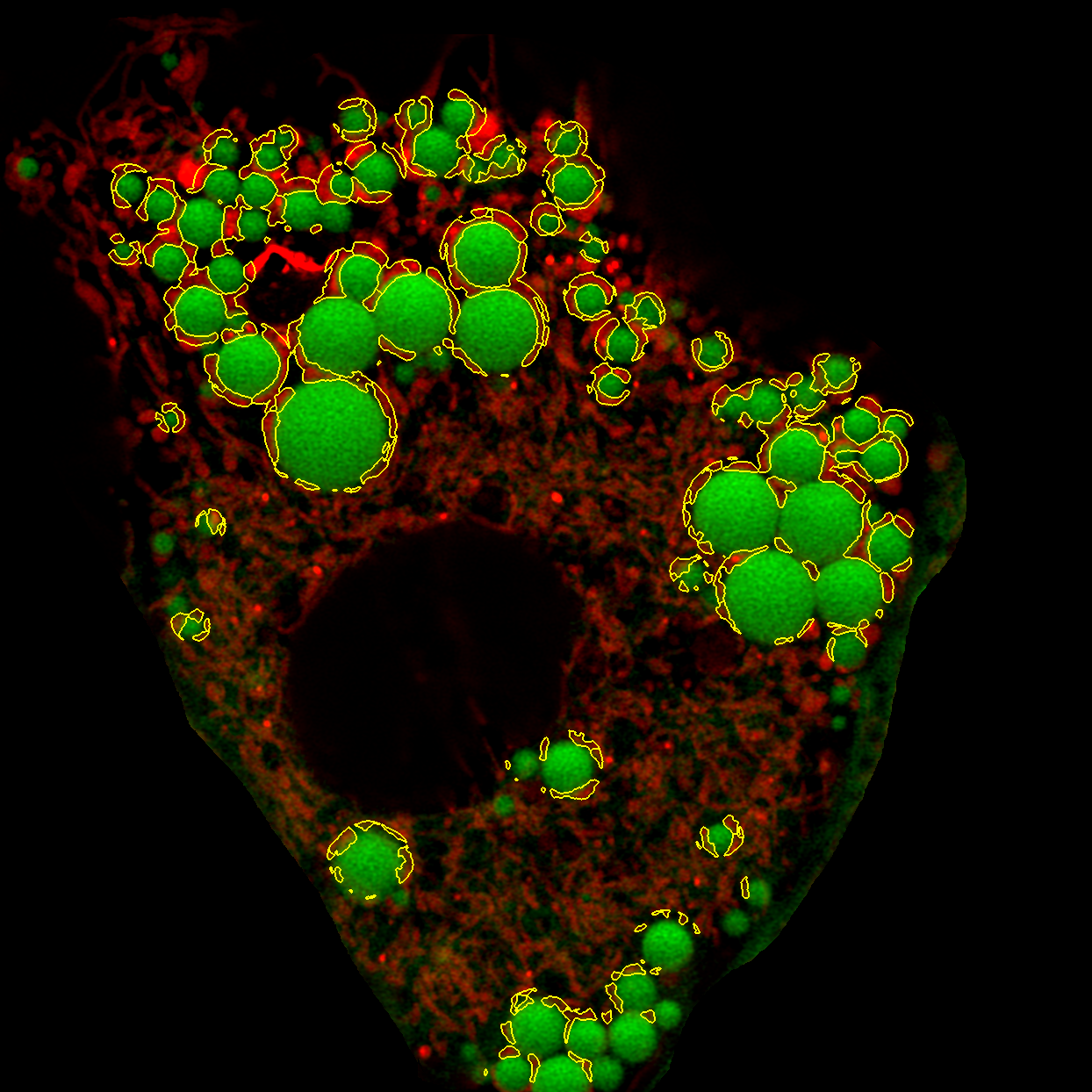

Supplement: Supplementary file 7 — Source data Fig. 5 [file 44318_2026_827_MOESM7_ESM.zip › Figure 5/Figure 5G/unt nt5_Out.czi - nt5 #3b.png]

## Slide 1
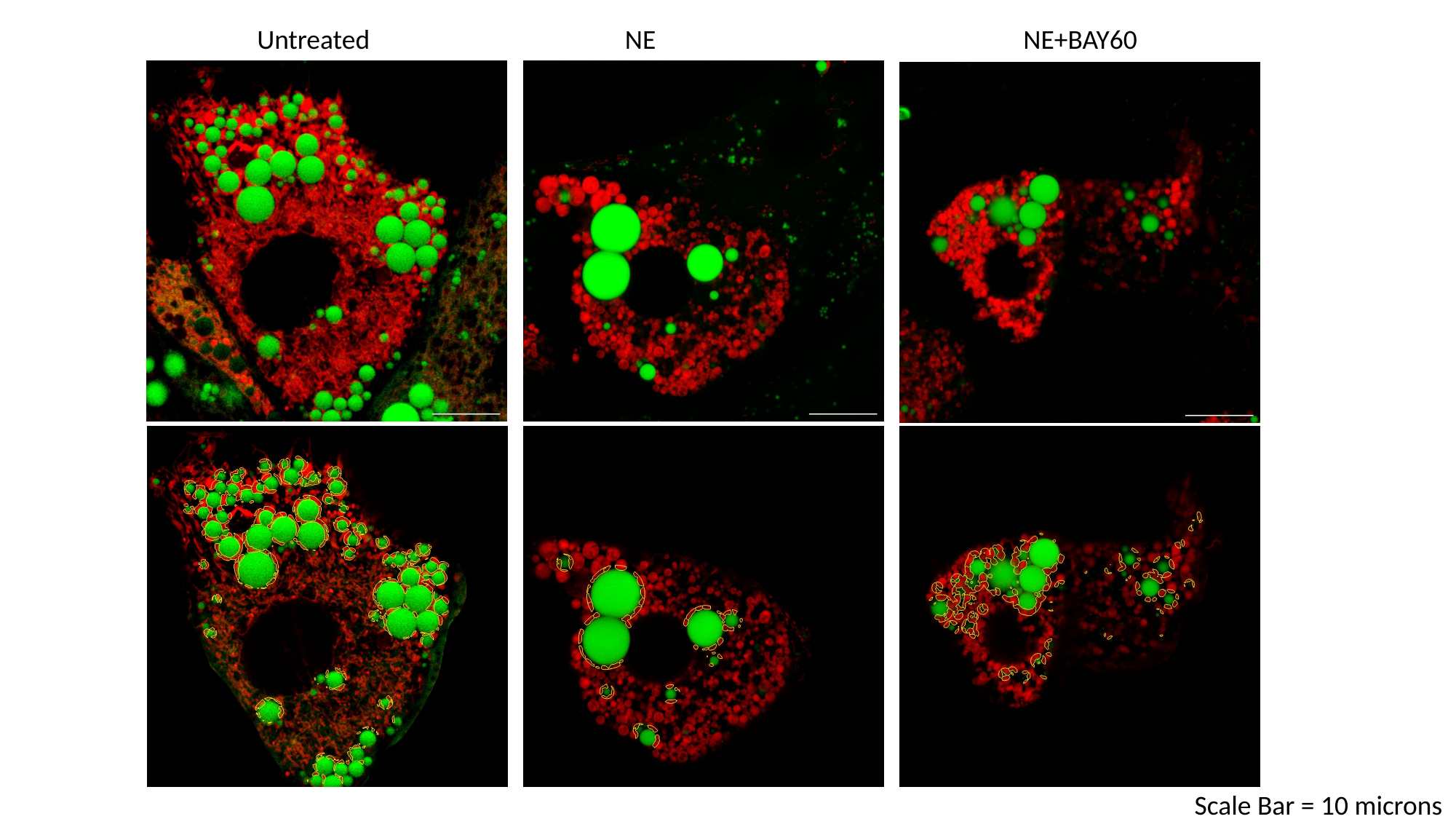

Untreated
NE
NE+BAY60
Scale Bar = 10 microns

Supplement: Supplementary file 7 — Source data Fig. 5 [file 44318_2026_827_MOESM7_ESM.zip › Figure 5/Figure 5G/Compiled_Images 5G.pptx]

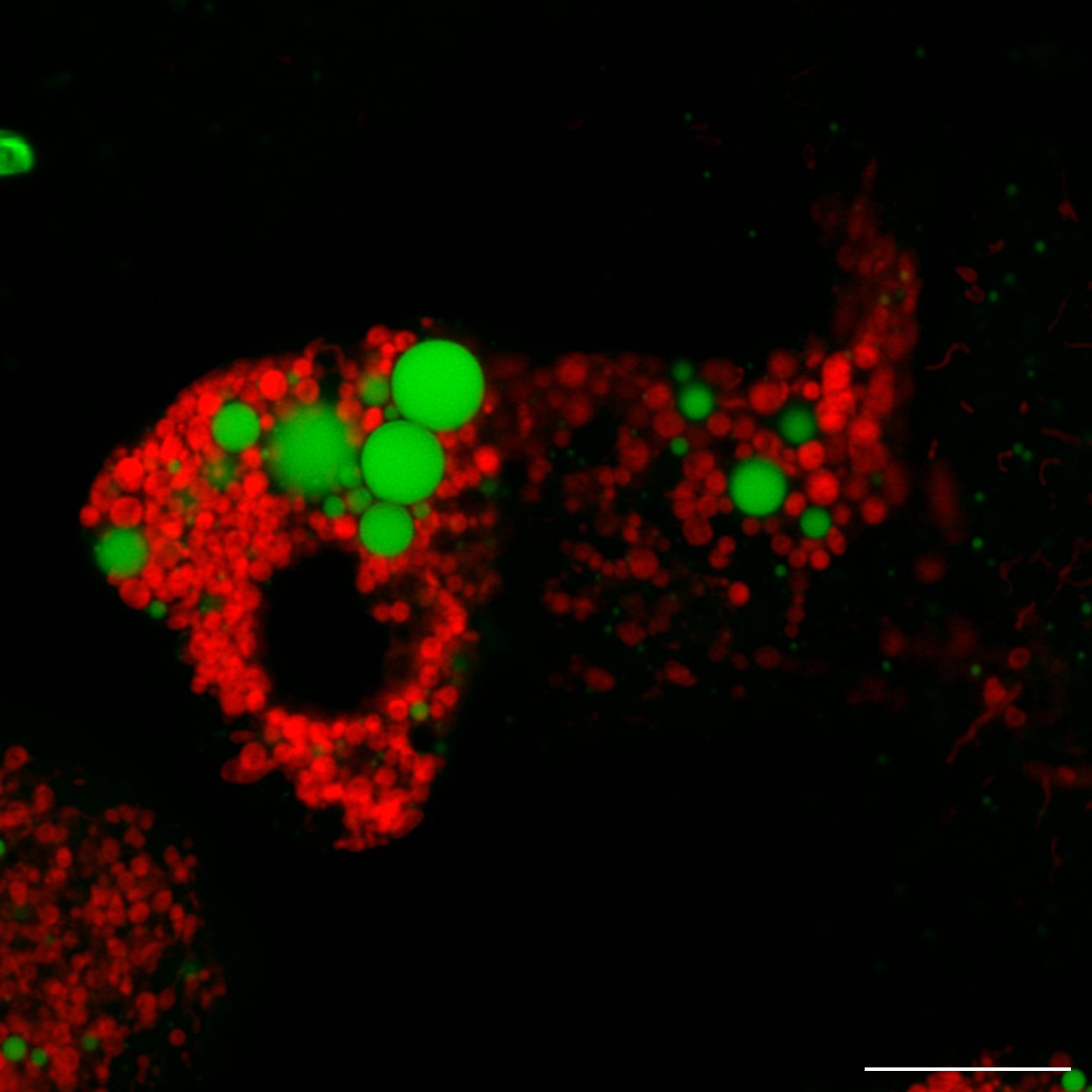

Supplement: Supplementary file 7 — Source data Fig. 5 [file 44318_2026_827_MOESM7_ESM.zip › Figure 5/Figure 5G/BAY60 NE Out.czi.jpg]

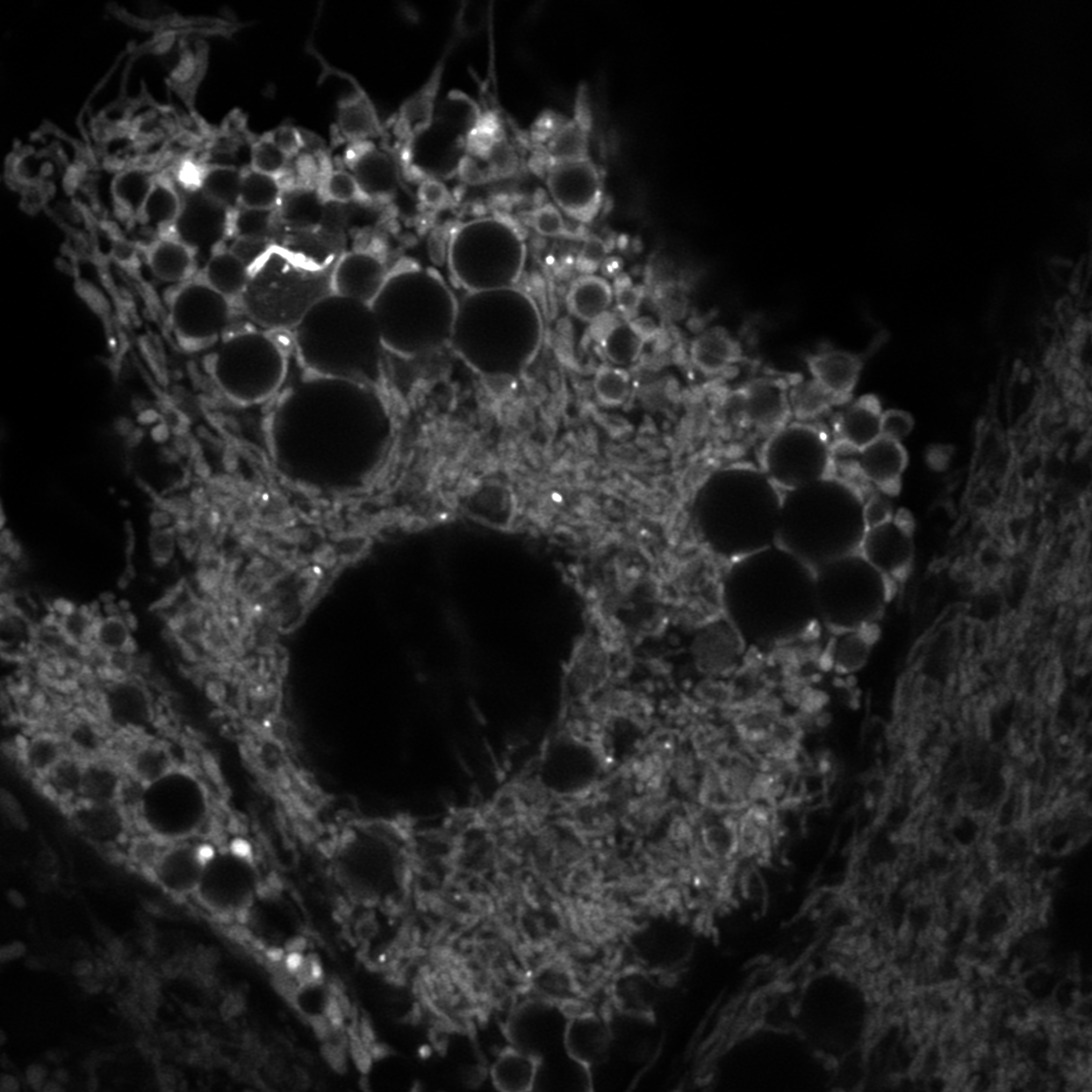

Supplement: Supplementary file 7 — Source data Fig. 5 [file 44318_2026_827_MOESM7_ESM.zip › Figure 5/Figure 5G/unt nt5_Out.czi - nt5 #3b_reverse.tif]

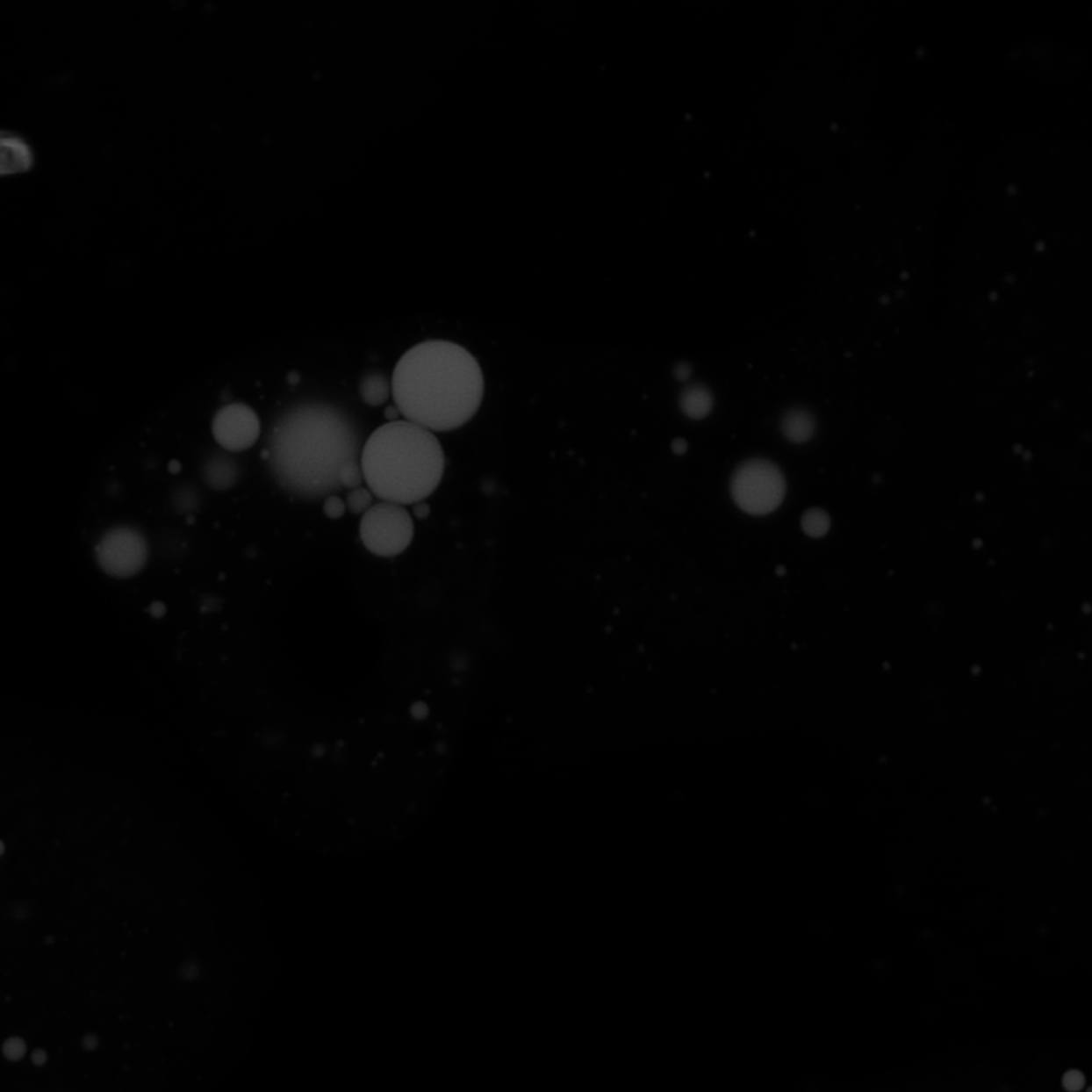

Supplement: Supplementary file 7 — Source data Fig. 5 [file 44318_2026_827_MOESM7_ESM.zip › Figure 5/Figure 5G/BAY60 NE Out.czi #4.tif]

## Slide 1
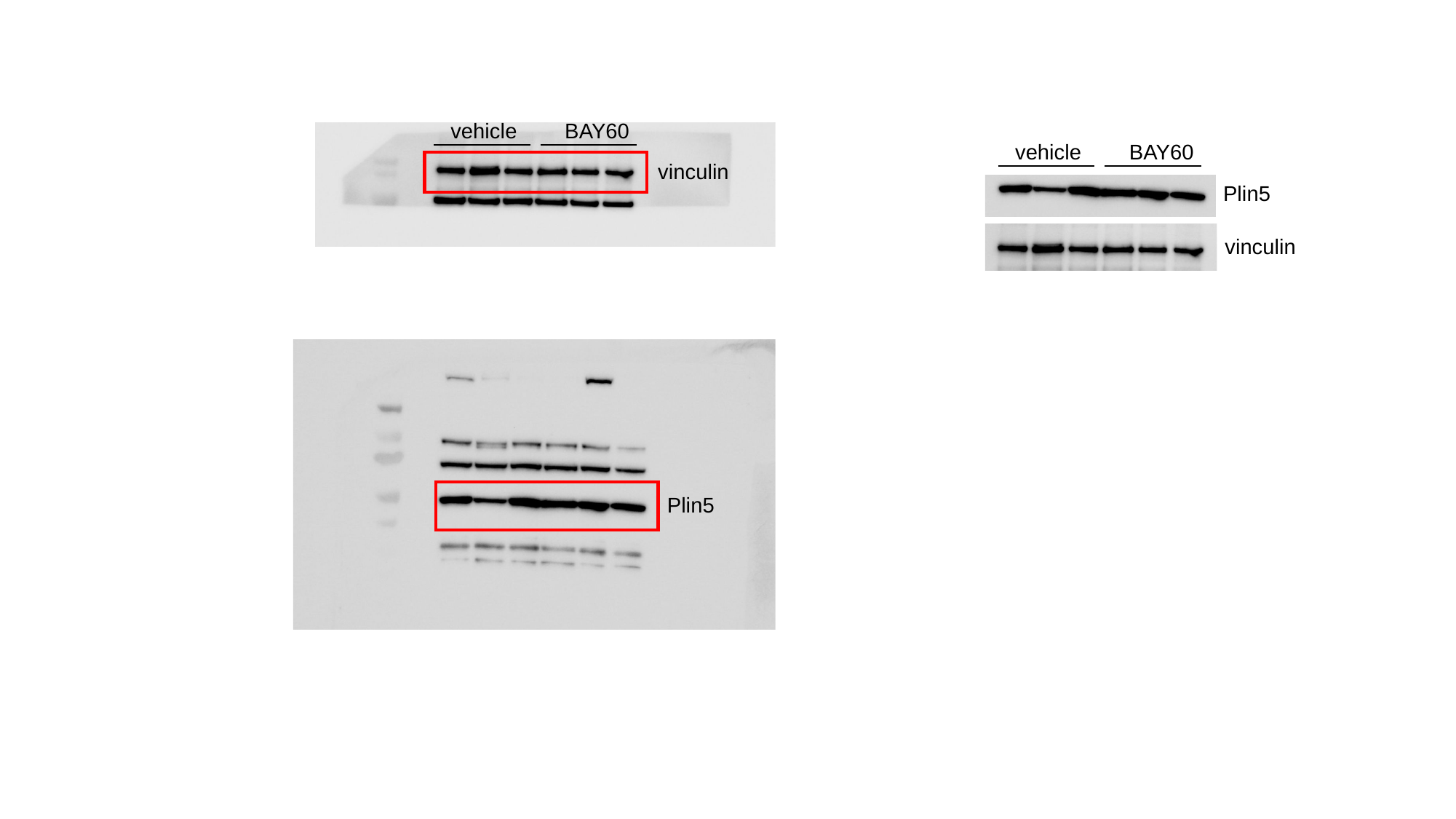

vehicle BAY60
vehicle BAY60
Plin5
vinculin
vinculin
Plin5

Supplement: Supplementary file 8 — Source data Fig. 6 [file 44318_2026_827_MOESM8_ESM.zip › Figure 6/Figure 6C blot.pptx]

## Slide 1
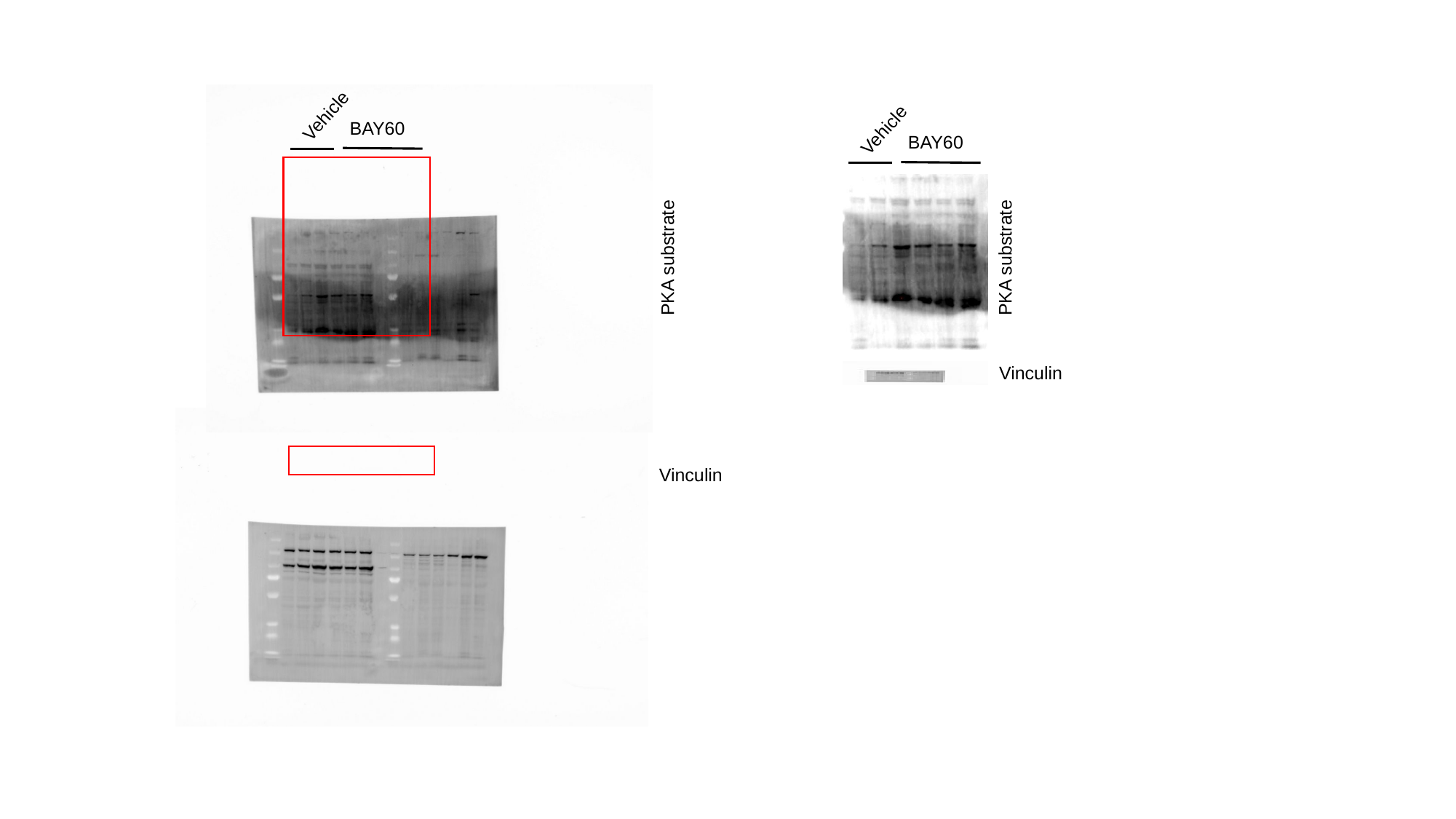

Vehicle
BAY60
PKA substrate
Vinculin
Vehicle
BAY60
PKA substrate
Vinculin

Supplement: Supplementary file 8 — Source data Fig. 6 [file 44318_2026_827_MOESM8_ESM.zip › Figure 6/Figure 6A blot.pptx]

## Slide 1
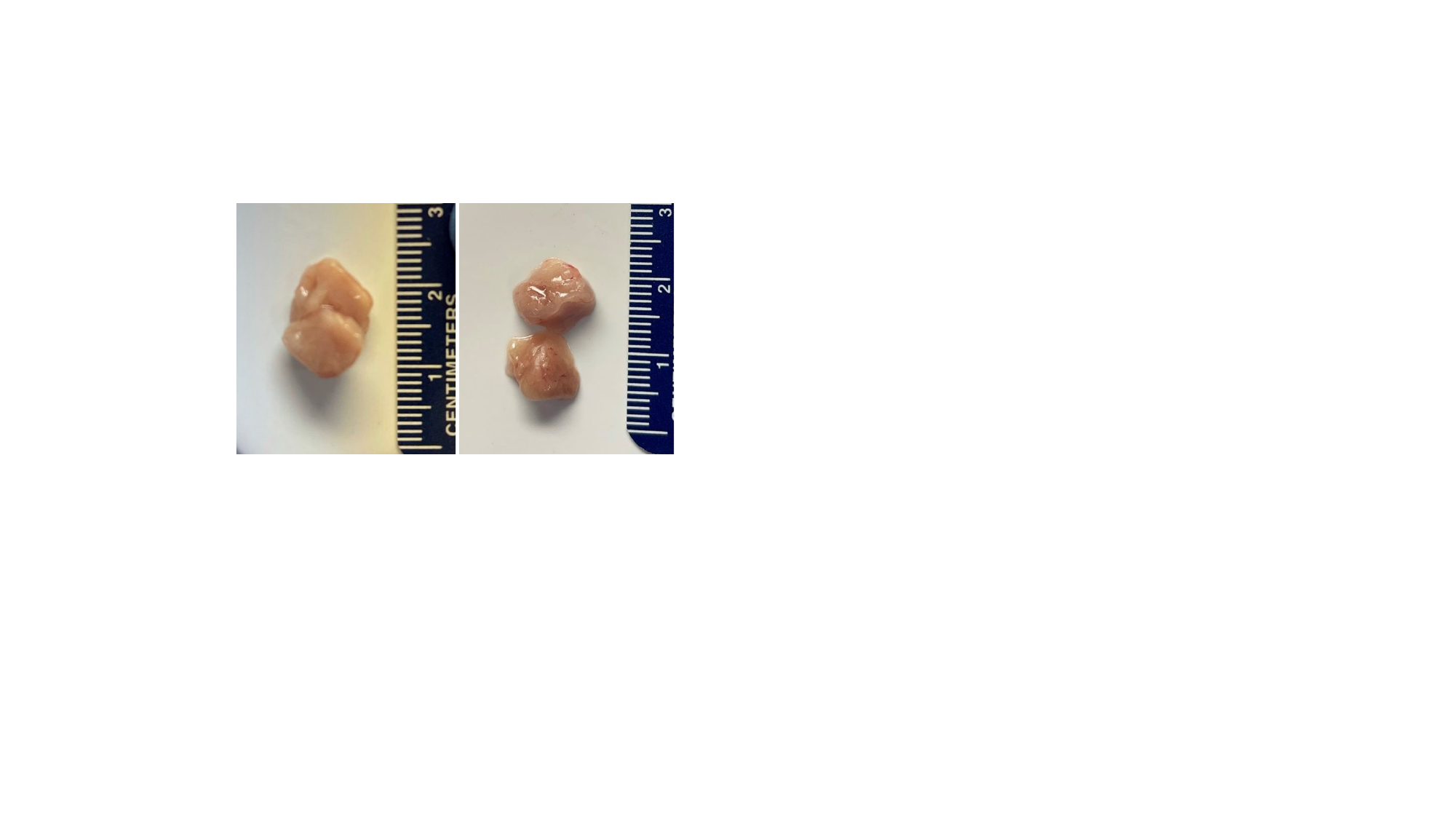

Supplement: Supplementary file 8 — Source data Fig. 6 [file 44318_2026_827_MOESM8_ESM.zip › Figure 6/Figure 6K.pptx]

## Slide 1
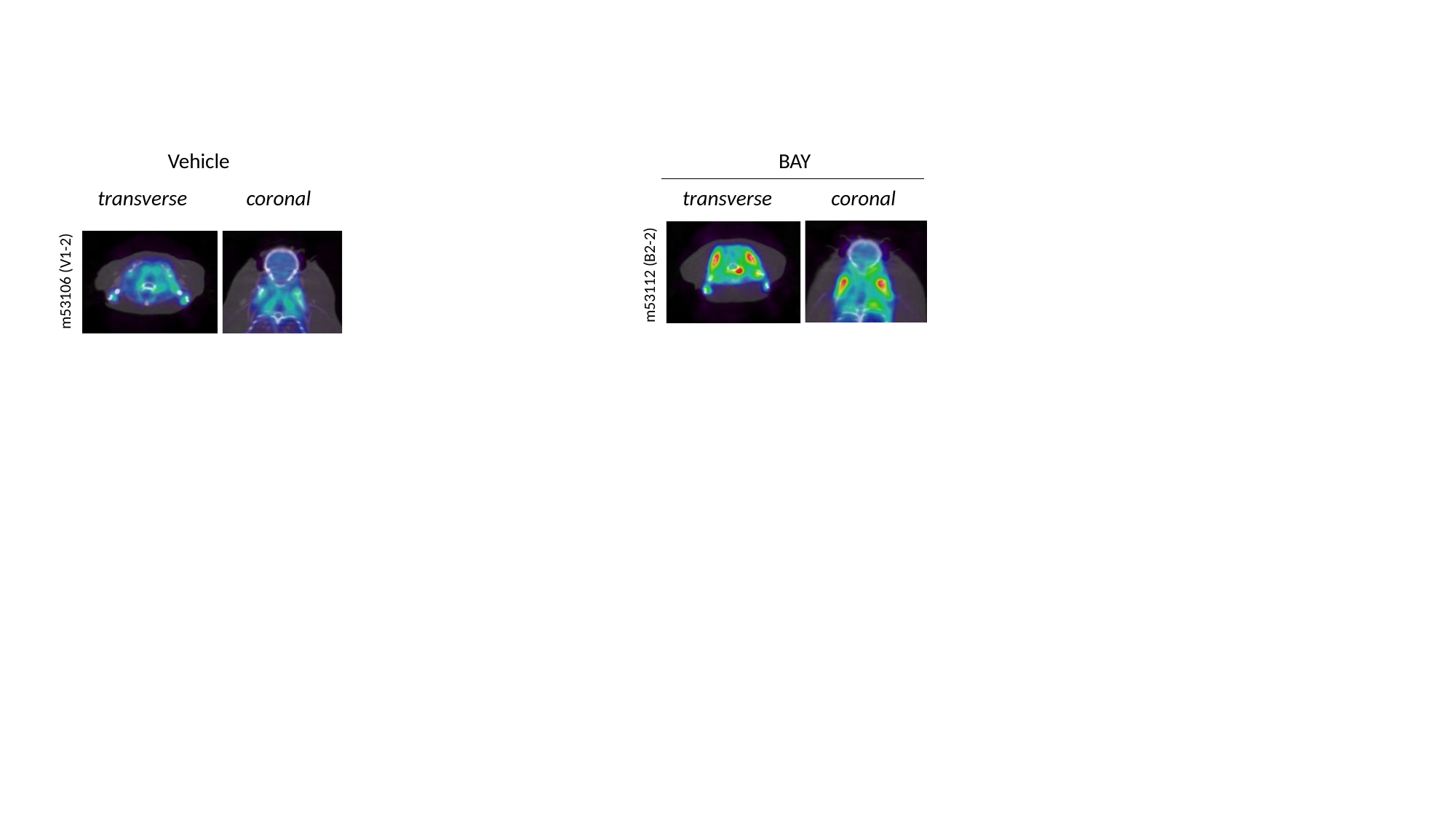

Vehicle
BAY
transverse
coronal
transverse
coronal
m53112 (B2-2)
m53106 (V1-2)

Supplement: Supplementary file 8 — Source data Fig. 6 [file 44318_2026_827_MOESM8_ESM.zip › Figure 6/Figure 6H/raw data PET CT 6H.pptx]

## Slide 1
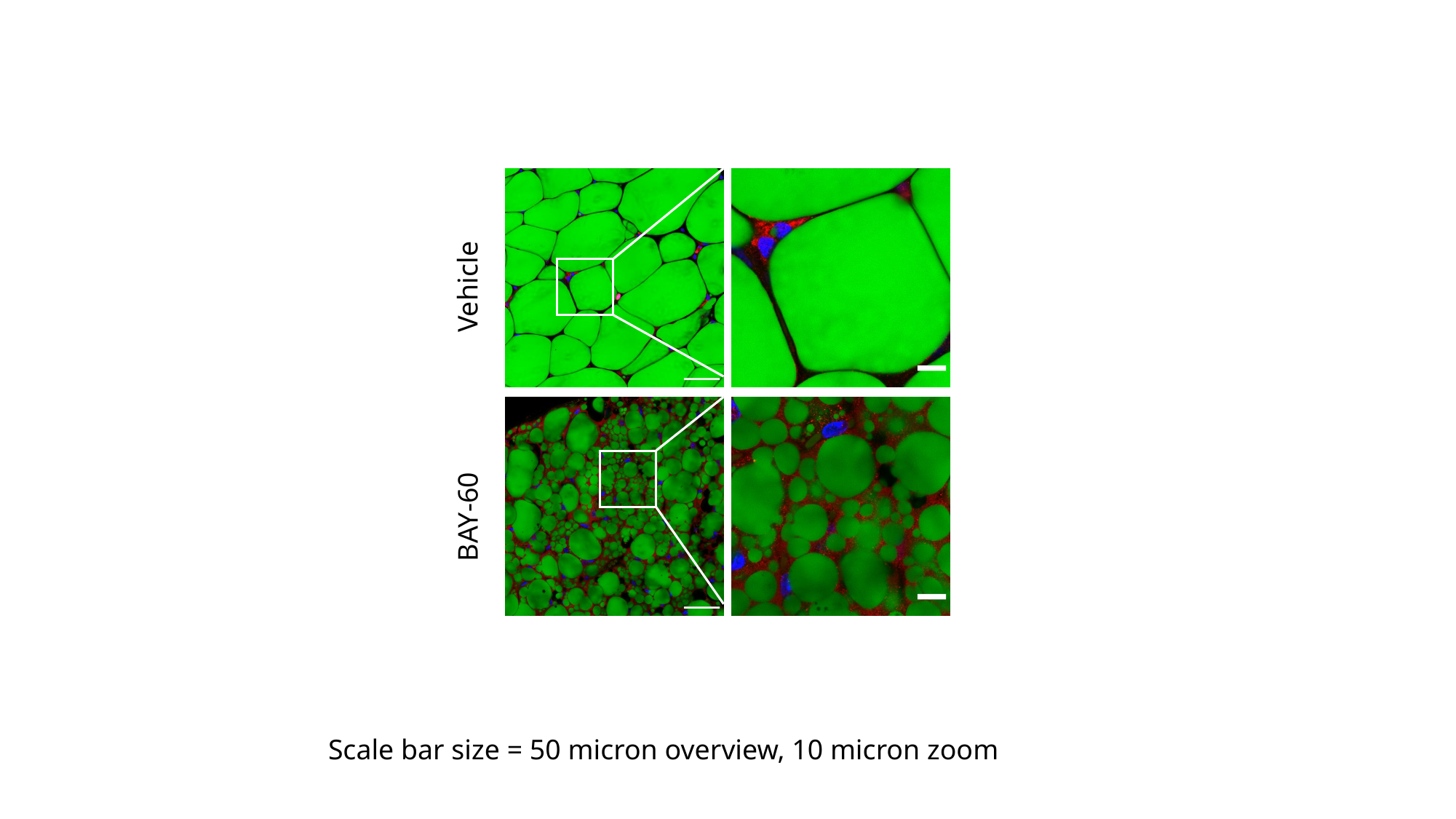

Vehicle
BAY-60
Scale bar size = 50 micron overview, 10 micron zoom

Supplement: Supplementary file 8 — Source data Fig. 6 [file 44318_2026_827_MOESM8_ESM.zip › Figure 6/Figure 6N/obob BAY-60 BAT updated whole mount fluorescence images.pptx]

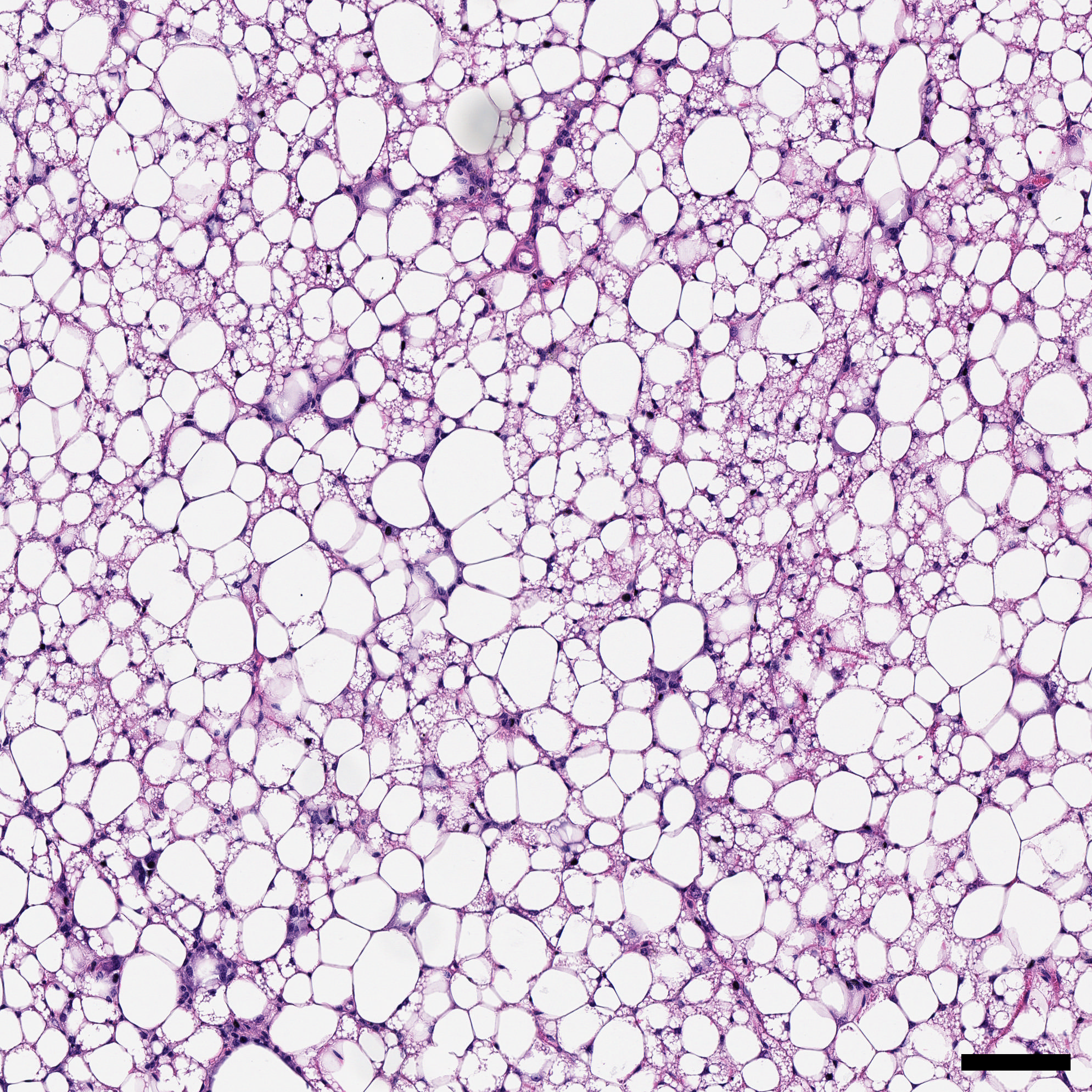

Supplement: Supplementary file 8 — Source data Fig. 6 [file 44318_2026_827_MOESM8_ESM.zip › Figure 6/Figure 6M/BAY60 139713_B3-3_BAT.svs - Series 1-4.tif_RGB.tif]

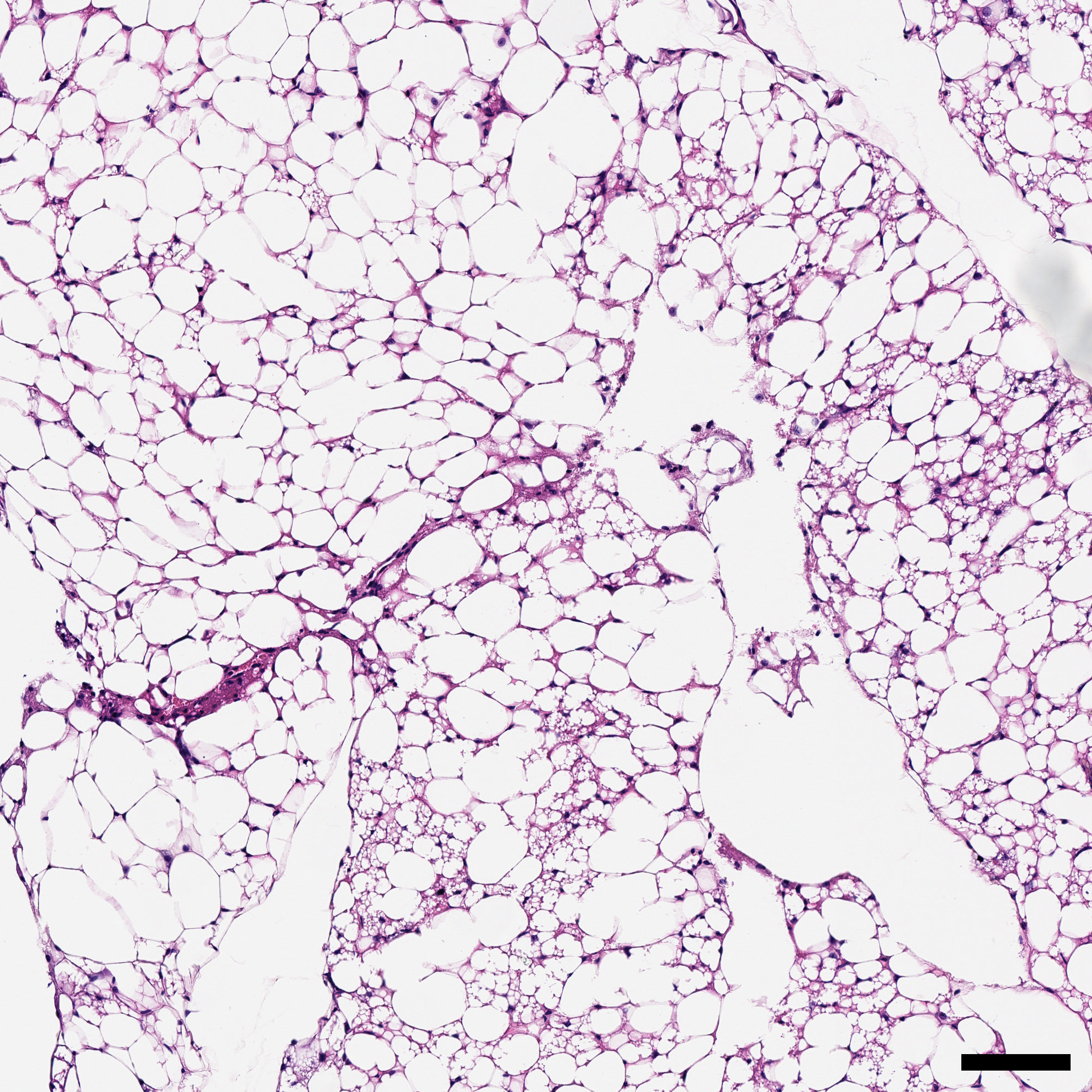

Supplement: Supplementary file 8 — Source data Fig. 6 [file 44318_2026_827_MOESM8_ESM.zip › Figure 6/Figure 6M/Vehicle 139709_V4-2_BAT.svs - Series 1-3.tif_RGB.tif]

## Slide 1
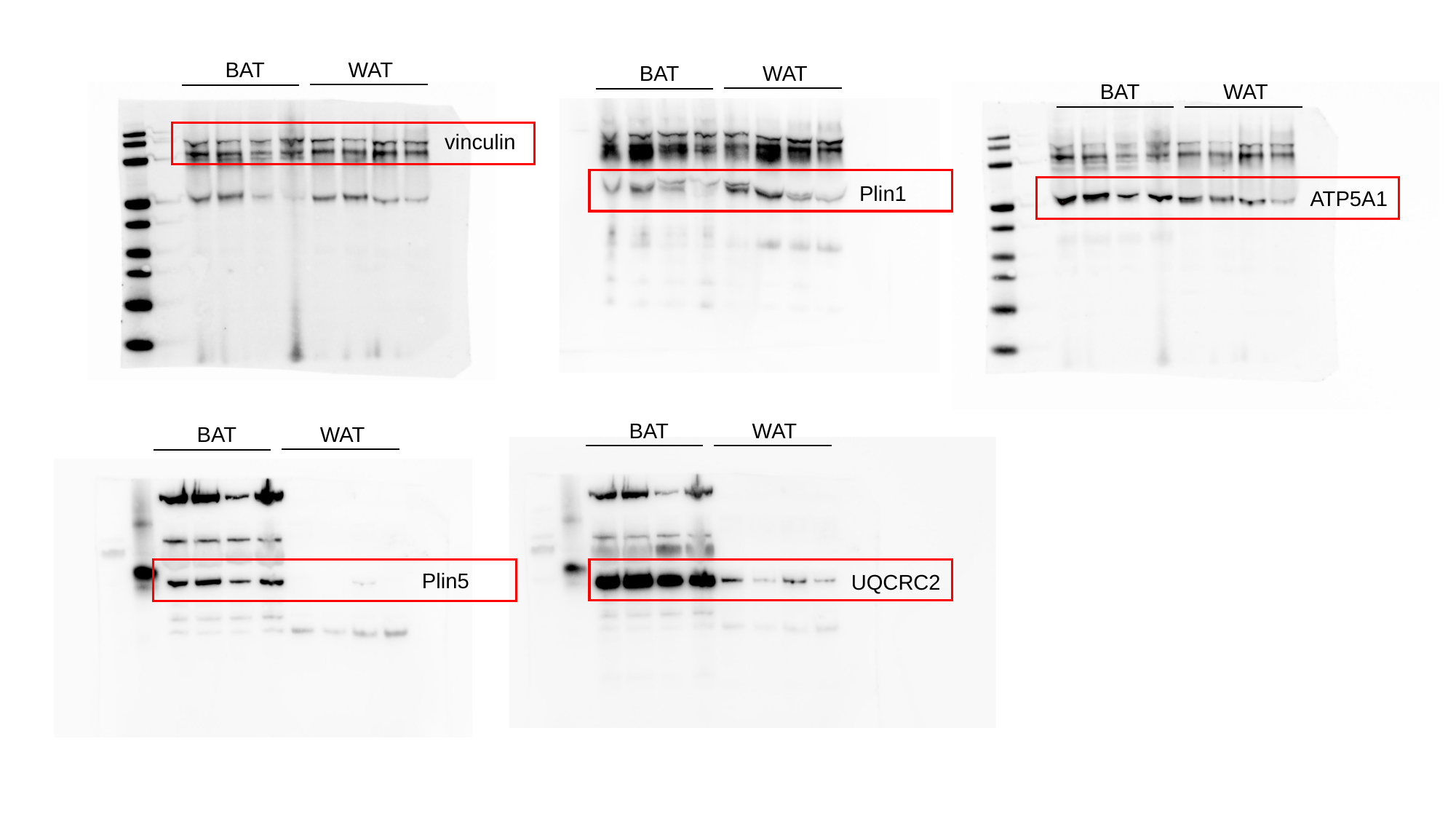

BAT WAT
BAT WAT
BAT WAT
vinculin
Plin1
ATP5A1
BAT WAT
BAT WAT
Plin5
UQCRC2

Supplement: Supplementary file 9 — Figure EV1 Source Data [file 44318_2026_827_MOESM9_ESM.zip › Figure EV1/Figure EV1B blot.pptx]

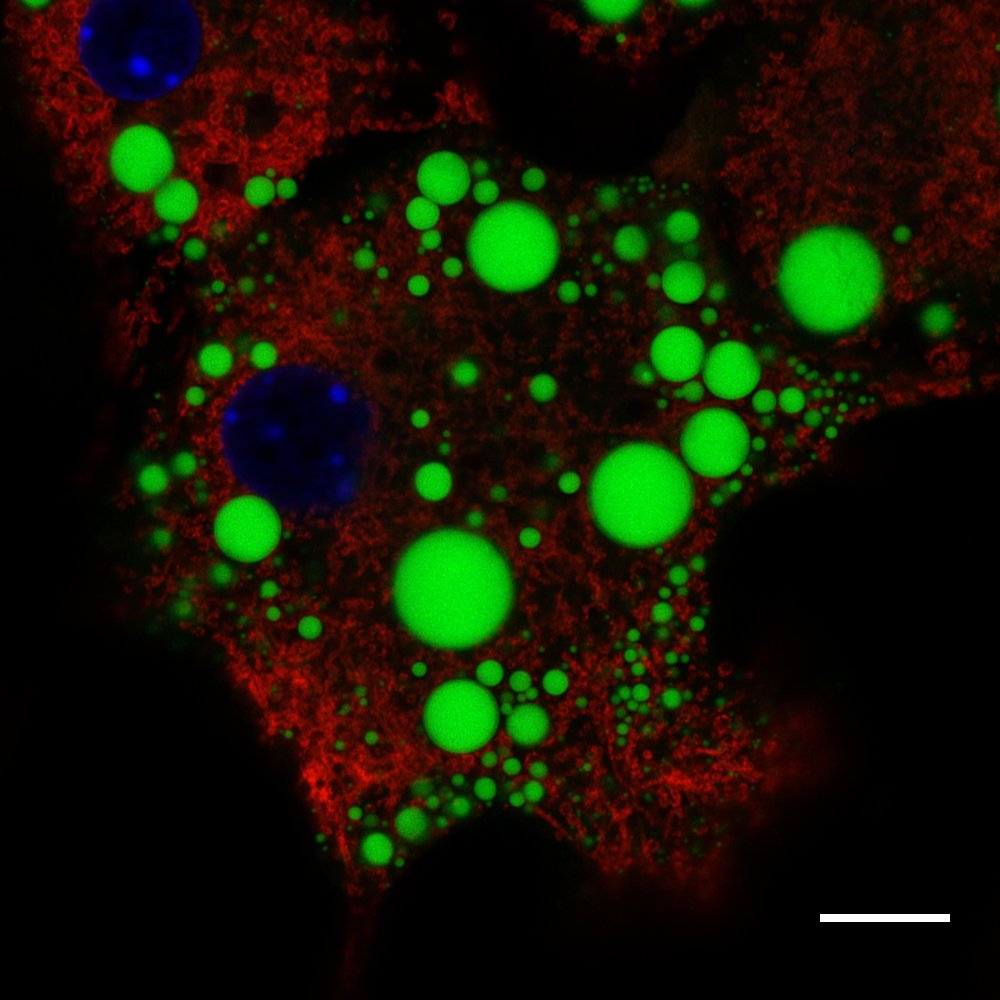

Supplement: Supplementary file 10 — Figure EV2 Source Data [file 44318_2026_827_MOESM10_ESM.zip › Figure EV2/Figure EV2C/Triac NE_Image 18_Out.jpg]

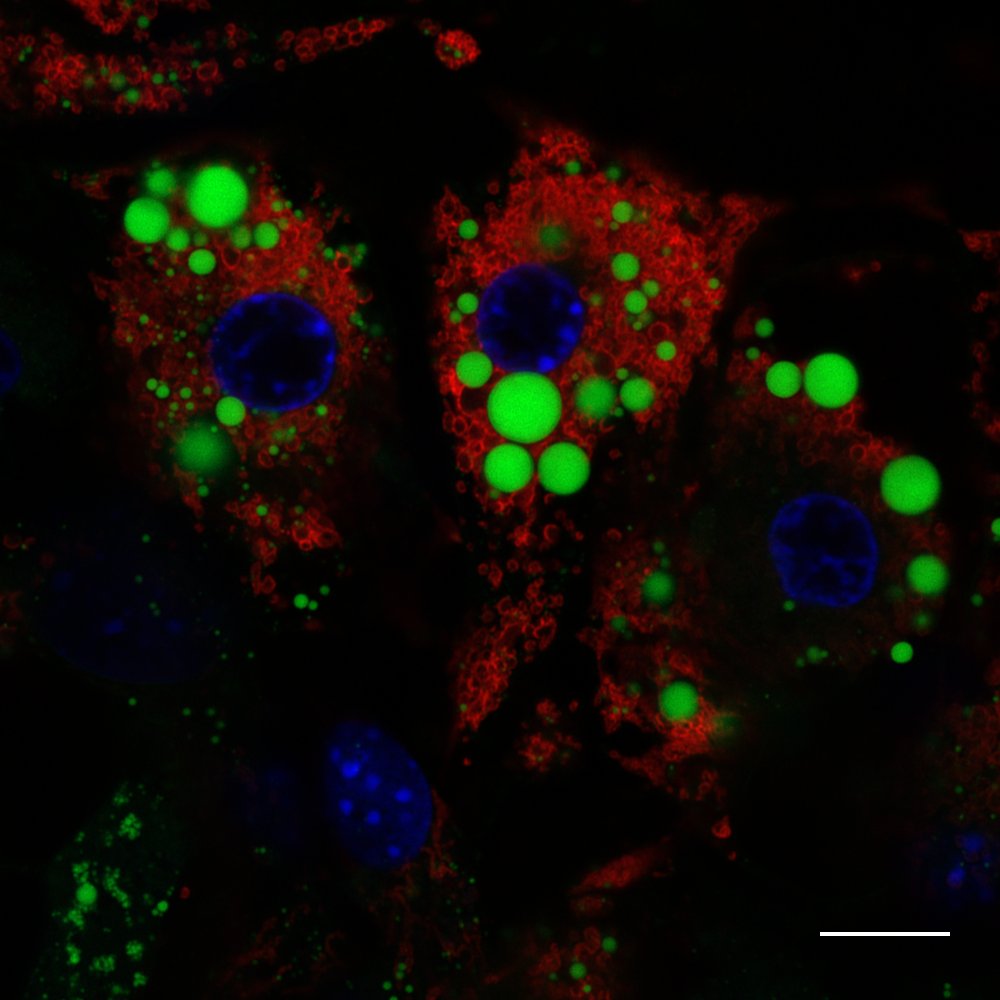

Supplement: Supplementary file 10 — Figure EV2 Source Data [file 44318_2026_827_MOESM10_ESM.zip › Figure EV2/Figure EV2C/unt NE_Image 2_Out.jpg]

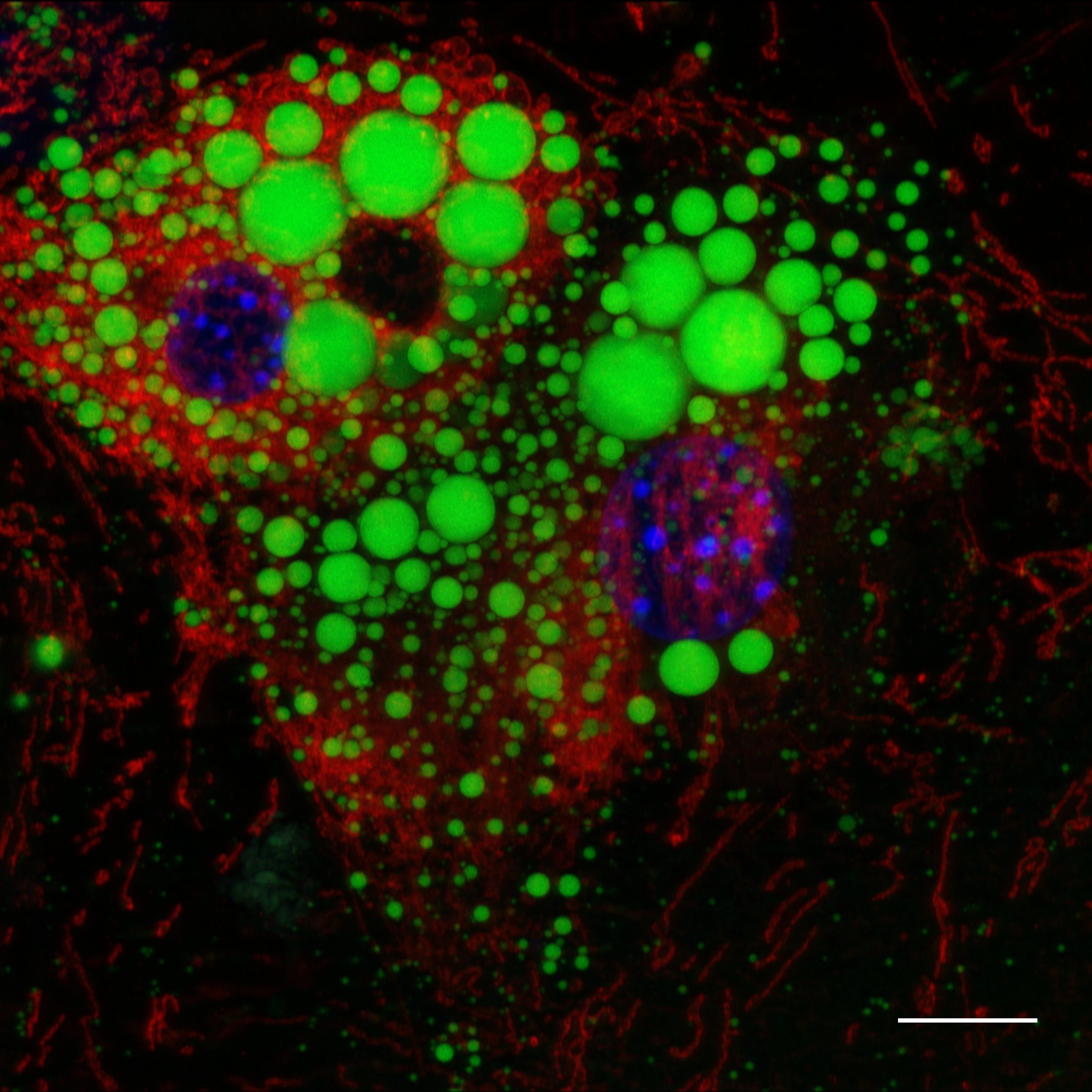

Supplement: Supplementary file 10 — Figure EV2 Source Data [file 44318_2026_827_MOESM10_ESM.zip › Figure EV2/Figure EV2C/Ru360 NE_Image 47_Zstack.jpg]

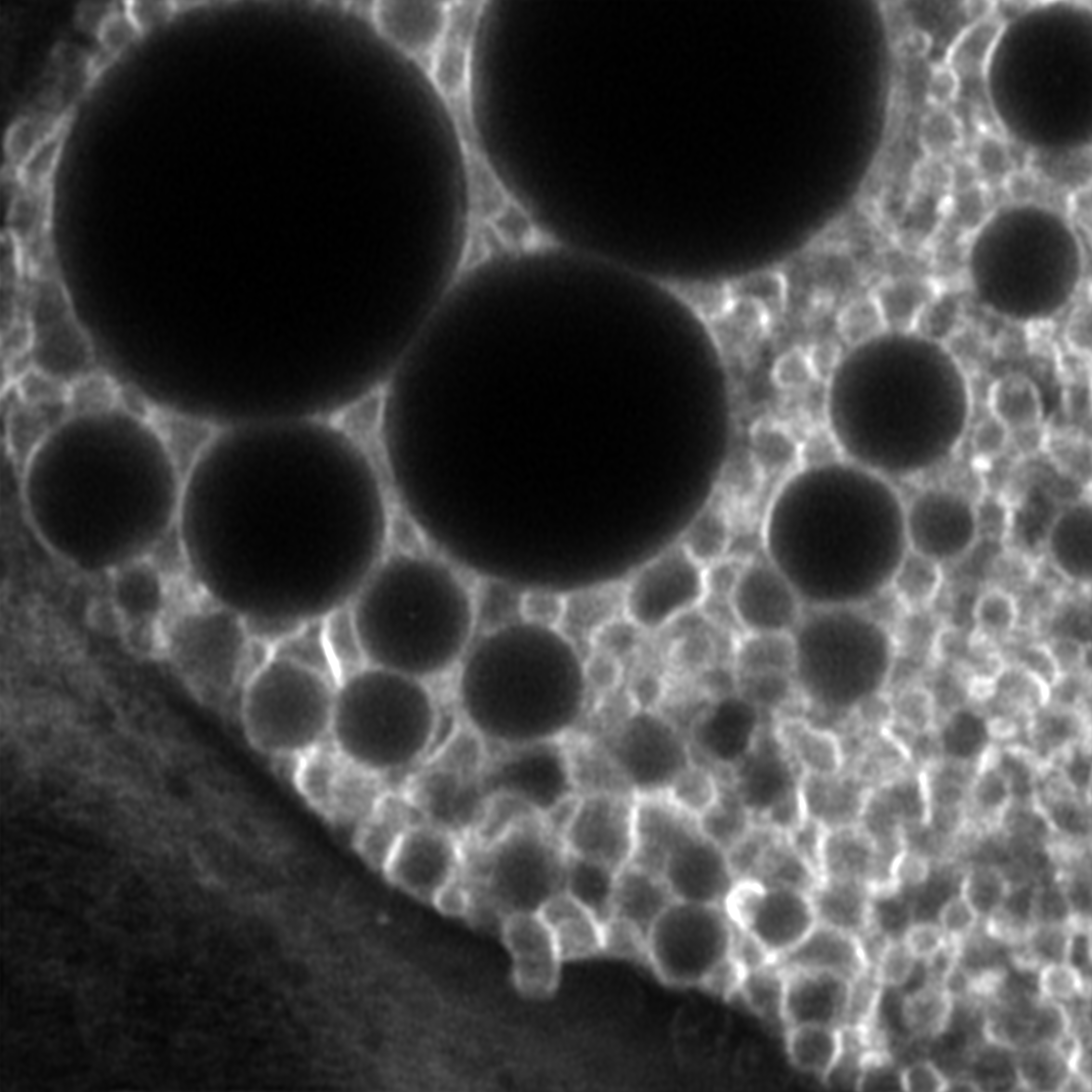

Supplement: Supplementary file 11 — Figure EV3 Source Data [file 44318_2026_827_MOESM11_ESM.zip › Figure EV3/Figure EV3A/90minValino.tif]

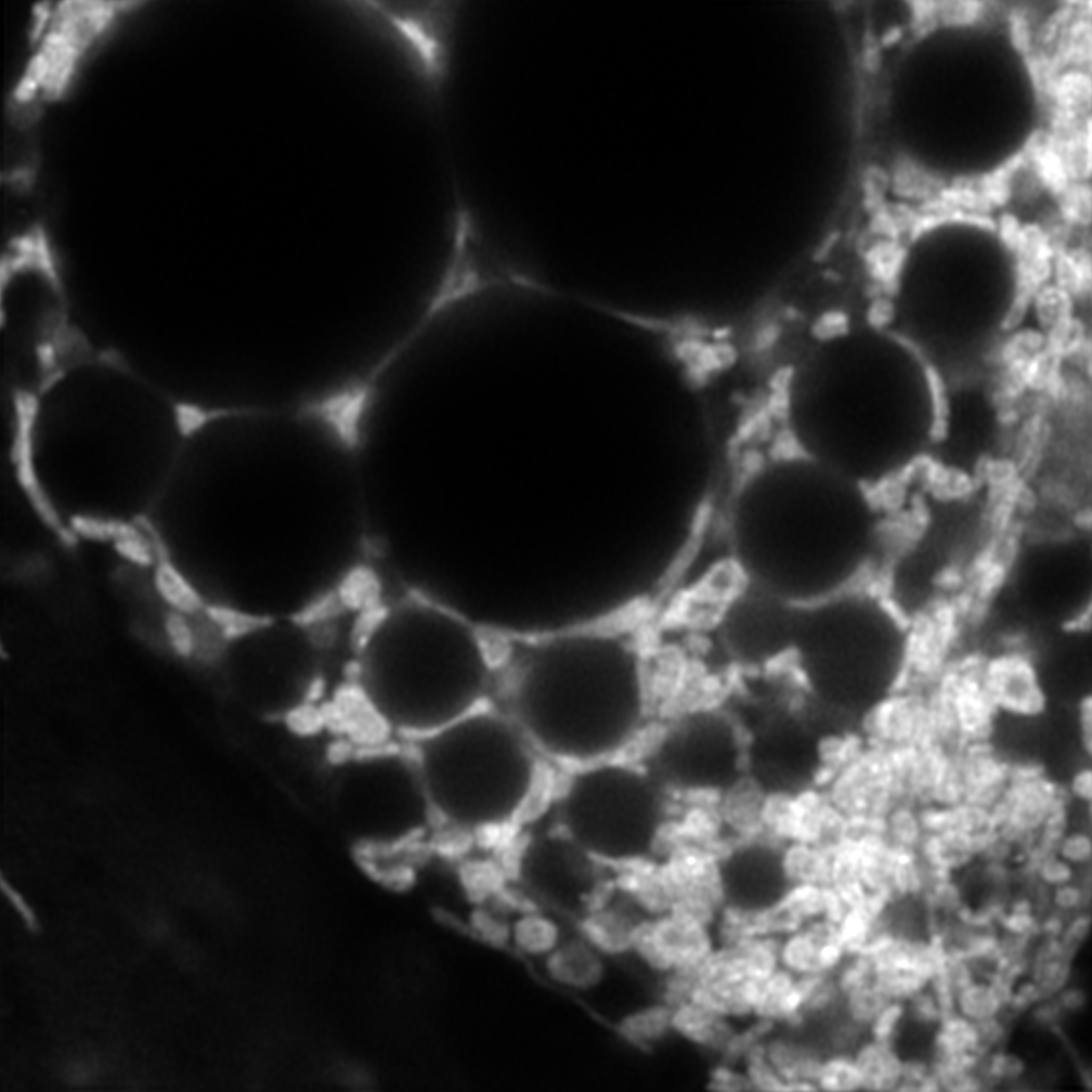

Supplement: Supplementary file 11 — Figure EV3 Source Data [file 44318_2026_827_MOESM11_ESM.zip › Figure EV3/Figure EV3A/Pre-treat.tif]

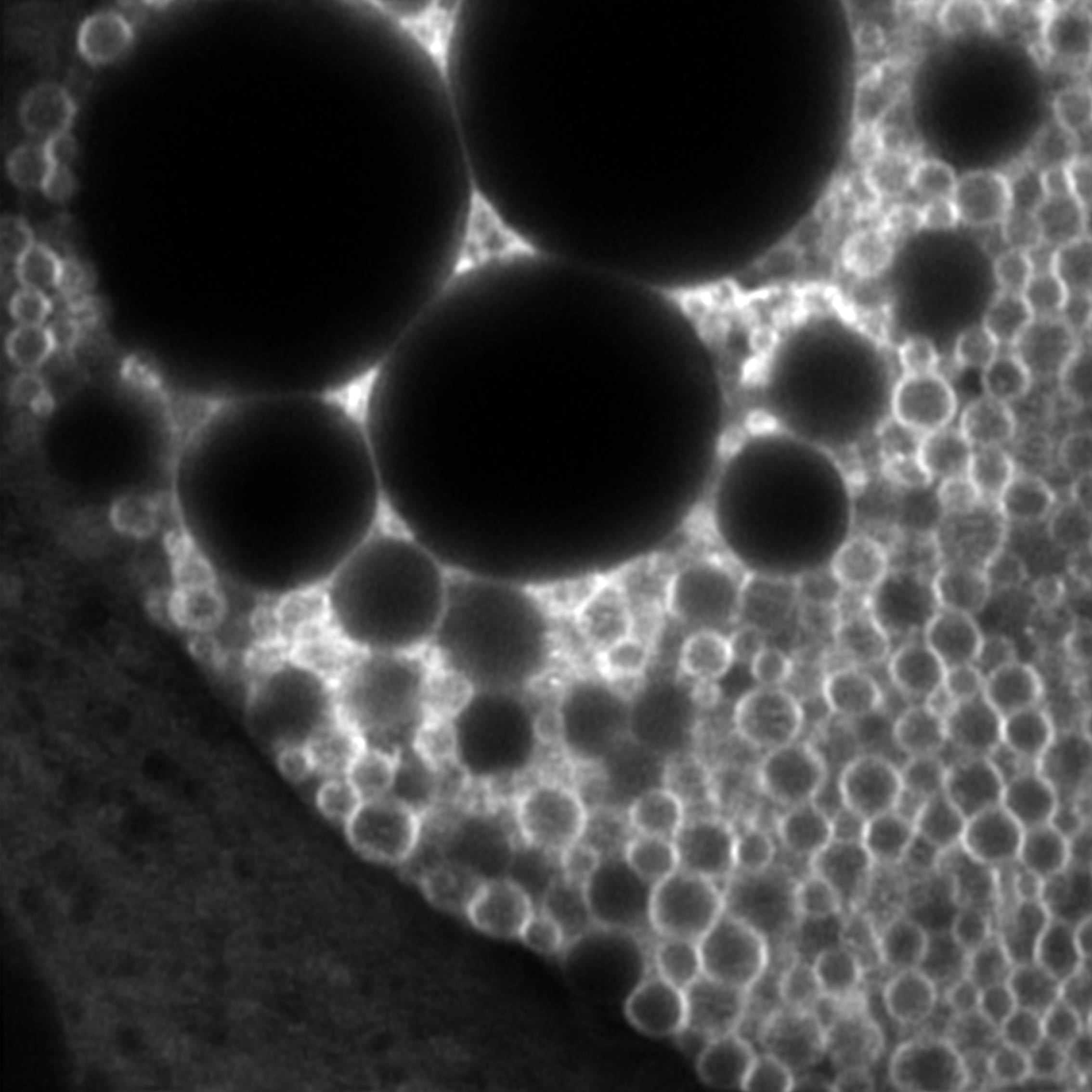

Supplement: Supplementary file 11 — Figure EV3 Source Data [file 44318_2026_827_MOESM11_ESM.zip › Figure EV3/Figure EV3A/20minValino.tif]
