# Supplementary material for: Mitochondrial calcium regulates lipid metabolism by modulating tethering of mitochondria to lipid droplets
Source: EMBO J. 2026 Jul 3;45(14):4820–48. doi: 10.1038/s44318-026-00827-8 (PMC13373242; doi:10.1038/s44318-026-00827-8)
Supplement: Supplementary file 7 — Source data Fig. 5 [file 44318_2026_827_MOESM7_ESM.zip › Figure 5/Figure 5I/Compiled_Representative_Images_And_Source 5I.pptx]

## Slide 1
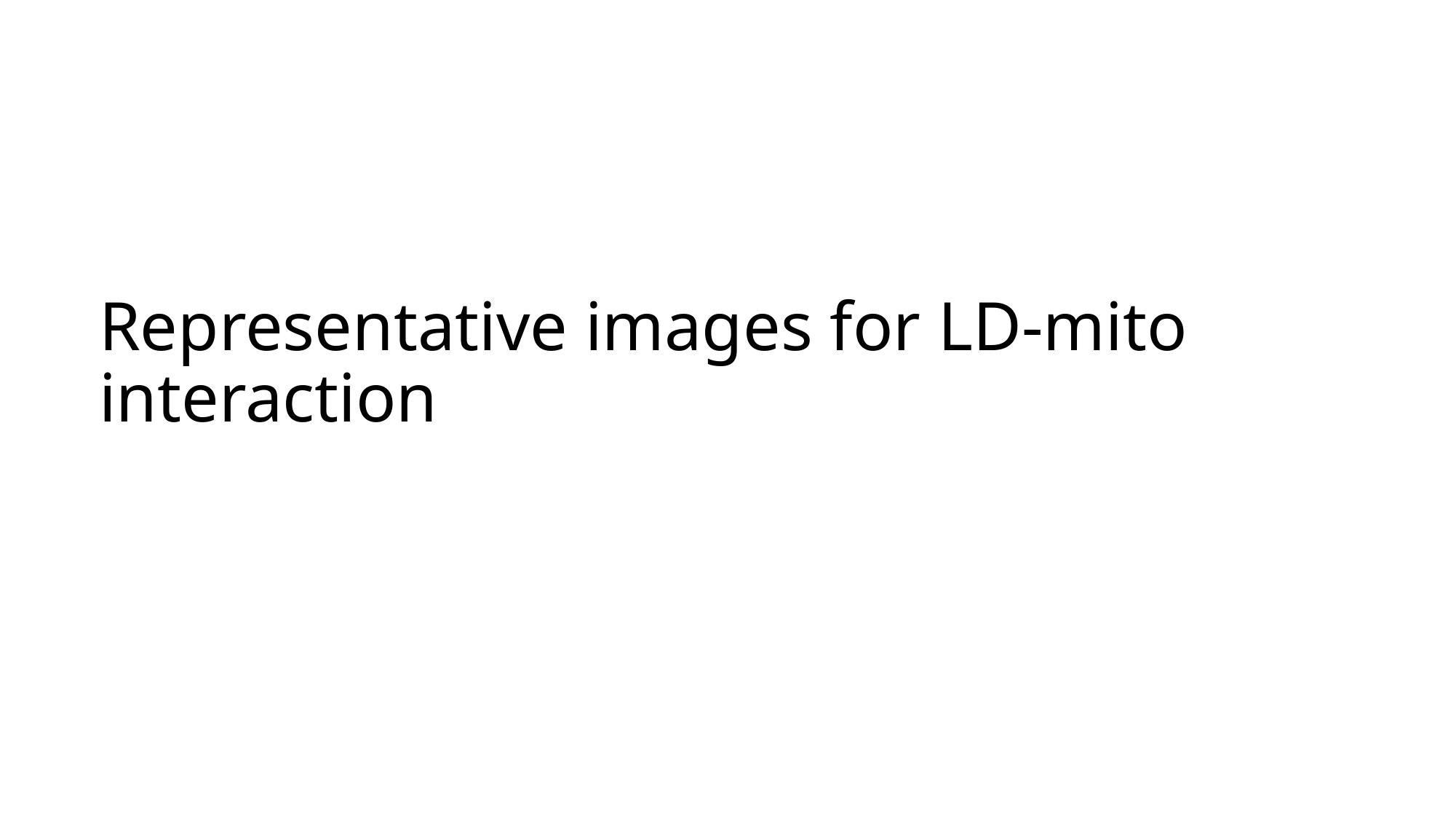

# Representative images for LD-mito interaction

## Slide 2
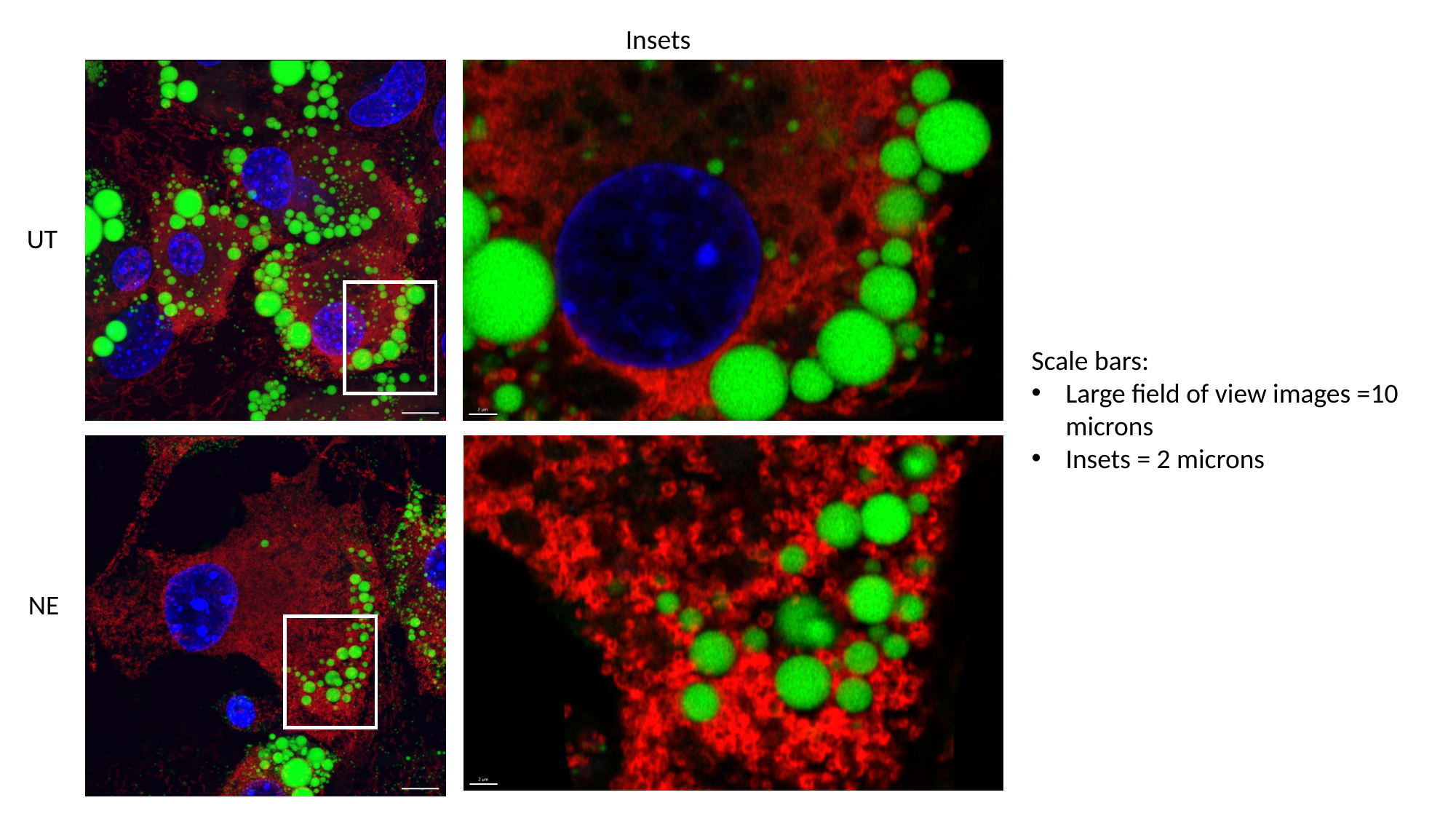

Insets
UT
Scale bars:
Large field of view images =10 microns
Insets = 2 microns
NE

## Slide 3
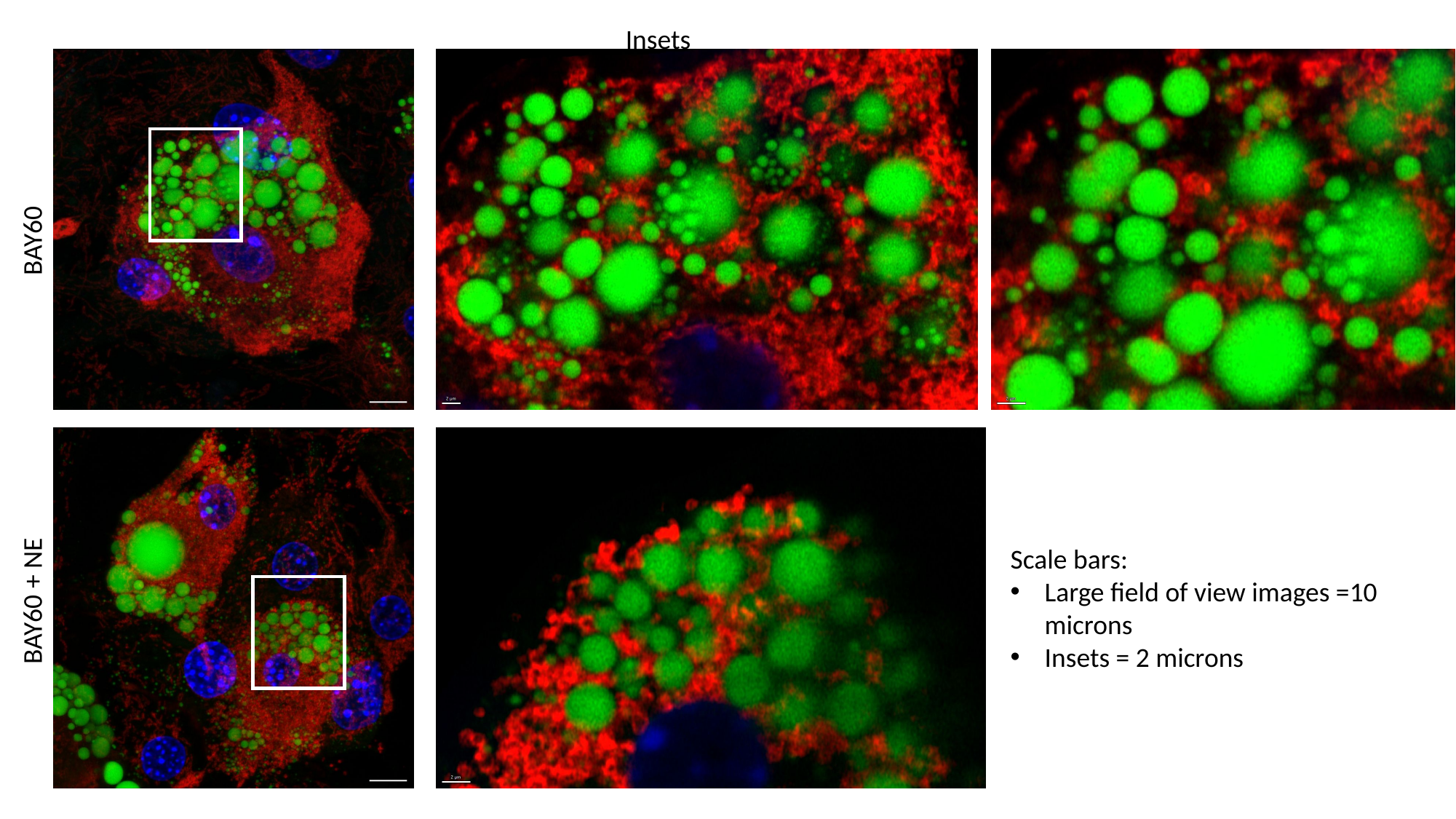

Insets
BAY60
Scale bars:
Large field of view images =10 microns
Insets = 2 microns
BAY60 + NE

## Slide 4
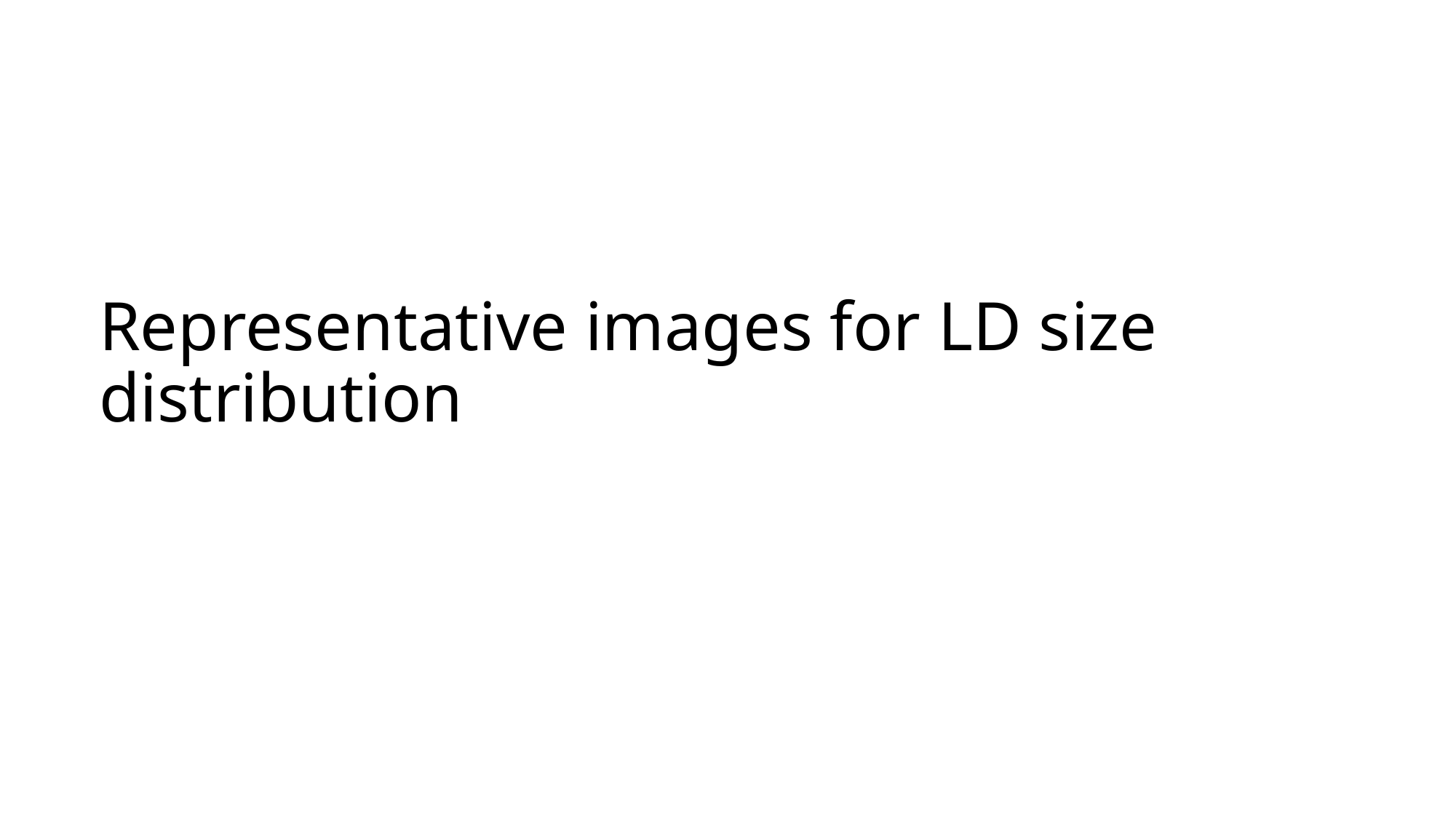

# Representative images for LD size distribution

## Slide 5
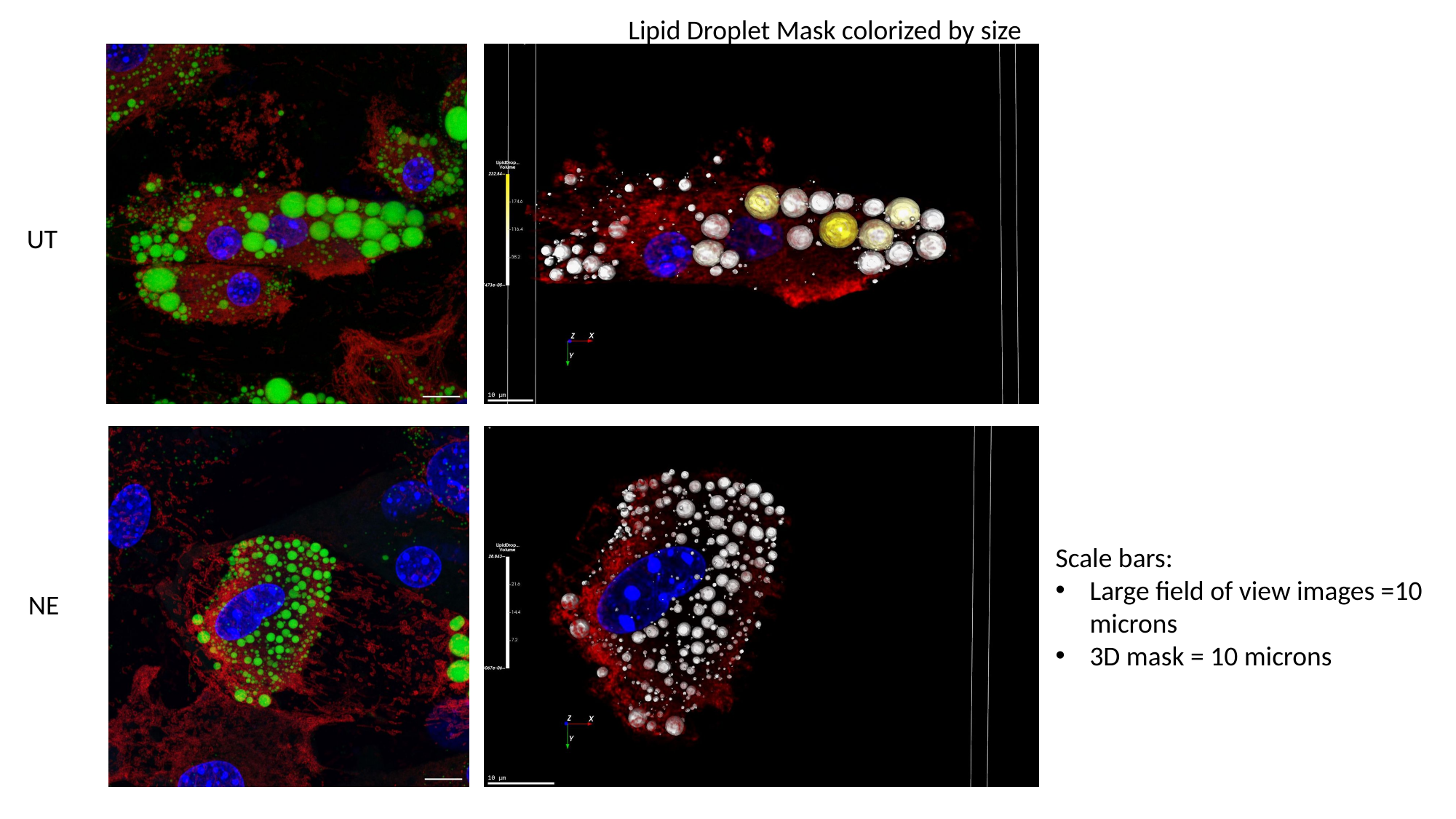

Lipid Droplet Mask colorized by size
UT
Scale bars:
Large field of view images =10 microns
3D mask = 10 microns
NE

## Slide 6
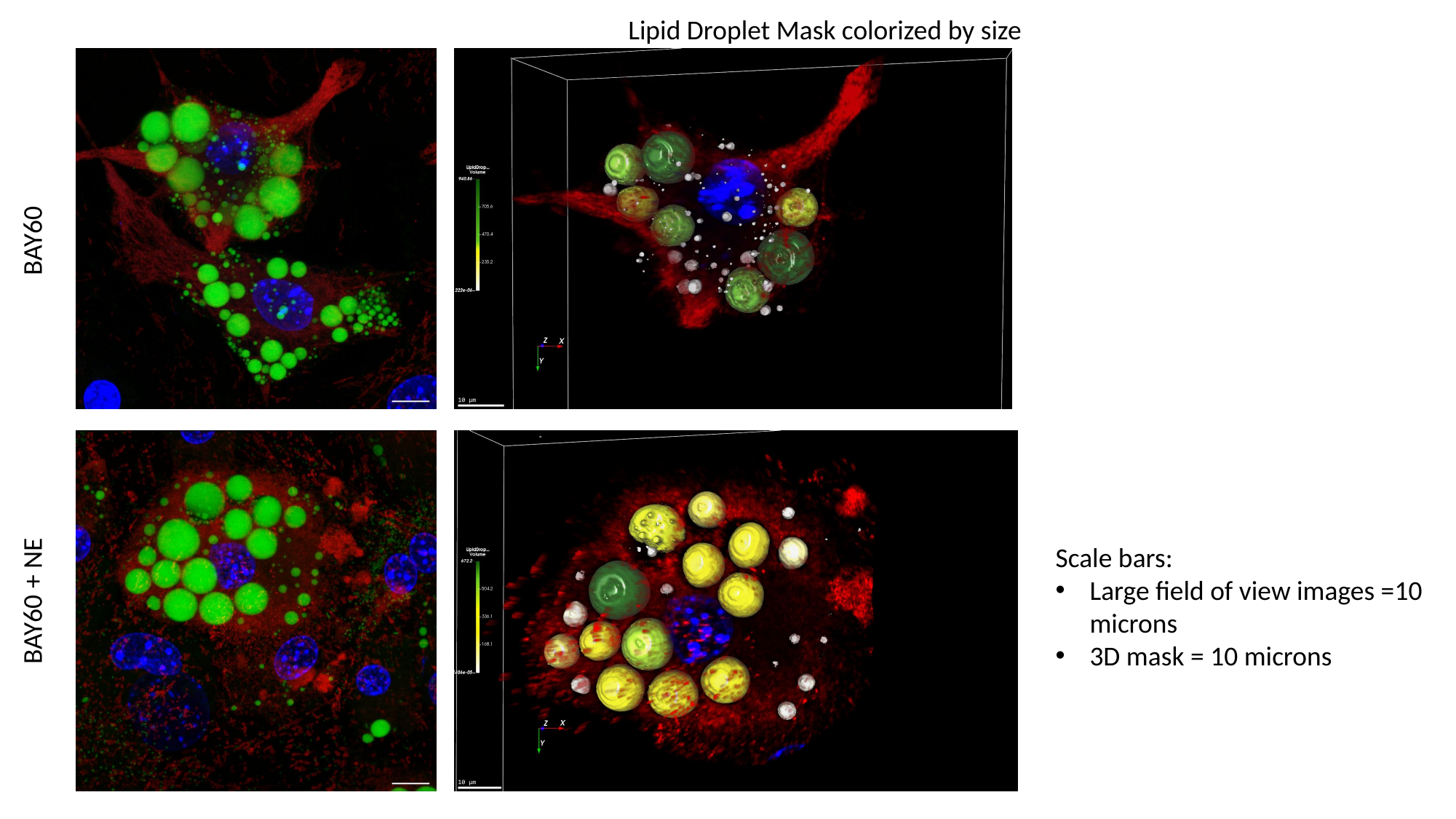

Lipid Droplet Mask colorized by size
BAY60
Scale bars:
Large field of view images =10 microns
3D mask = 10 microns
BAY60 + NE
