# Supplementary material for: Mitochondrial calcium regulates lipid metabolism by modulating tethering of mitochondria to lipid droplets
Source: EMBO J. 2026 Jul 3;45(14):4820–48. doi: 10.1038/s44318-026-00827-8 (PMC13373242; doi:10.1038/s44318-026-00827-8)
Supplement: Supplementary file 11 — Figure EV3 Source Data [file 44318_2026_827_MOESM11_ESM.zip › Figure EV3/Figure EV3C/embj0033-0418.pdf]

Article

SOURCE DATA · TRANSPARENT PROCESS · OPEN ACCESS

THE EMBO JOURNAL

Hormone-induced mitochondrial fission is utilized by brown adipocytes as an amplification pathway for energy expenditure

Jakob D Wikstrom[1,2,†], Kiana Mahdaviani[1,†], Marc Liesa[1,‡], Samuel B Sereda[1,‡], Yaguang Si[1], Guy Las[1,3], Gilad Twig[1,4], Natasa Petrovic[2], Cristina Zingaretti[5], Adam Graham[6], Saverio Cinti[5], Barbara E Corkey[1], Barbara Cannon[2], Jan Nedergaard[2] & Orian S Shirihai[1,3,*]

Abstract

Adrenergic stimulation of brown adipocytes (BA) induces mitochondrial uncoupling, thereby increasing energy expenditure by shifting nutrient oxidation towards thermogenesis. Here we describe that mitochondrial dynamics is a physiological regulator of adrenergically-induced changes in energy expenditure. The sympathetic neurotransmitter Norepinephrine (NE) induced complete and rapid mitochondrial fragmentation in BA, characterized by Drp1 phosphorylation and Opa1 cleavage. Mechanistically, NE-mediated Drp1 phosphorylation was dependent on Protein Kinase-A (PKA) activity, whereas Opa1 cleavage required mitochondrial depolarization mediated by FFAs released as a result of lipolysis. This change in mitochondrial architecture was observed both in primary cultures and brown adipose tissue from cold-exposed mice. Mitochondrial uncoupling induced by NE in brown adipocytes was reduced by inhibition of mitochondrial fission through transient Drp1 DN overexpression. Furthermore, forced mitochondrial fragmentation in BA through Mfn2 knock down increased the capacity of exogenous FFAs to increase energy expenditure. These results suggest that, in addition to its ability to stimulate lipolysis, NE induces energy expenditure in BA by promoting mitochondrial fragmentation. Together these data reveal that adrenergically-induced changes to mitochondrial dynamics are required for BA thermogenic activation and for the control of energy expenditure.

Keywords brown adipose tissue; energy expenditure; mitochondrial dynamics
Subject Categories Metabolism; Membrane & Intracellular Transport
DOI 10.1002/embj.201385014 | Received 21 March 2013 | Revised 19 November 2013 | Accepted 21 November 2013 | Published online 15 January 2014
EMBO Journal (2014) 33, 418–436

See also: **AW Gao & RH Houtkooper** (March 2014)

Introduction

Compelling evidence for the existence of brown adipocytes in humans suggests brown adipose tissue metabolism as a potential mechanism for energy dissipation, weight loss and improved glucose tolerance (Nedergaard *et al*, 2007; Cypess *et al*, 2009; van Marken Lichtenbelt *et al*, 2009; Virtanen *et al*, 2009; Zingaretti *et al*, 2009). While various successful approaches for the expansion of brown adipose tissue (BAT) or the browning of white adipocytes have been suggested (Wu *et al*, 2012), these are unlikely to have a major impact on energy expenditure if the brown adipocyte is not stimulated. Studies performed in rodents show that diet-induced BAT energy expenditure is not influenced by nutrient availability directly but rather by adrenergic stimulation (Bachman *et al*, 2002; Betz *et al*, 2012). This emphasizes that any benefit from BAT expansion is largely dependent on the ability to stimulate the β-adrenergic receptor or its downstream targets.

Mitochondria are dynamic organelles that undergo fusion and fission events. Proteins that mediate mitochondrial fusion include Mitofusin 1 & 2 for outer membrane fusion and Opa1 for inner membrane fusion. Fission is mediated by outer membrane Fis1 and Mff, which recruit Drp1 as the molecular motor of fission. We have previously demonstrated that mitochondria go through continuous cycles of selective fusion and fission, referred to as the "mitochondrial life cycle", to maintain the quality of its function (Twig *et al*, 2008b, Twig *et al* 2010; Molina *et al*, 2009; Mouli *et al*, 2009). Moreover, others and we have found that changes in mitochondrial architecture can represent an adaptation of mitochondria to respire according to the bioenergetic needs of the cell (Liesa & Shirihai, 2013). Conditions requiring high mitochondrial ATP synthesis capacity and/or efficiency, such as limited nutrient availability, are associated with mitochondrial elongation (Gomes *et al*, 2011; reviewed in Liesa & Shirihai, 2013). On the other hand, conditions of excess energy sup-

1 Department of Medicine, Boston University School of Medicine, Boston, MA, USA
2 Department of Molecular Biosciences, The Wenner-Gren Institute, Stockholm University, Stockholm, Sweden
3 Department of Clinical Biochemistry, Faculty of Medicine, Ben Gurion University of the Negev, Beer-Sheva, Israel
4 Department of Medicine and the Dr. Pinchas Bornstein Talpiot Medical Leadership Program, Chaim Sheba Medical Center, Tel-Hashomer, Israel
5 Department of Experimental and Clinical Medicine, University of Ancona, Ancona, Italy
6 Center for Nanoscales System, Harvard University, Cambridge, MA, USA
*Corresponding author. Tel: +1 617 230 8570; Fax: +1 617 638 7124; E-mail: shirihai@bu.edu
†,‡These authors contributed equally.

© 2014 The Authors. This is an open access article under the terms of the Creative Commons Attribution License, which permits use, distribution and reproduction in any medium, provided the original work is properly cited.

ply and relatively low ATP demand, such as β-cells exposed to excess nutrients, acutely induce mitochondrial fragmentation (Wikstrom *et al*, 2007; Liesa & Shirihai 2013; Molina *et al*, 2009). Indeed, increased mitochondrial reactive oxygen species (ROS) production and an increase in uncoupling are common features of various conditions, not directly linked to changes in nutrient availability, that lead to mitochondrial fragmentation (Kim *et al*, 2008; Parone *et al*, 2008). This raises the possibility that mitochondrial fragmentation supports uncoupled respiration and thus increases energy expenditure by promoting shifting nutrient oxidation towards heat production, rather than towards mitochondrial ATP synthesis. To test this hypothesis, we explored a system where a robust shift from coupled to uncoupled respiration and increased energy expenditure can occur. The brown adipocyte offers a unique system where transition to uncoupling can occur within minutes and in a physiological rather than pathological context. Therefore, it represents a good model for studying the regulation of energy expenditure induced by hormones. In this regard, this study tested the hypothesis that NE induces changes to mitochondrial architecture that serve as an amplification pathway for uncoupling in brown adipocytes.

Results

Norepinephrine potentiates the effect of free fatty acids on energy expenditure

Thermogenesis in brown adipocytes is activated through an adrenergic signaling pathway, which stimulates the release of free fatty acids (FFAs) from lipid droplets (Cannon & Nedergaard, 2004). Released FFAs then directly activate Ucp1, with Long Chain Fatty Acids (LCFA, such as oleate or palmitate) being the most efficient FA activating Ucp1 *in vitro* (Cannon *et al*, 1977; Fedorenko *et al*, 2012). However, *in vivo* and in the absence of adrenergic stimulation, circulating FFAs alone cannot induce the activation of thermogenesis in BAT (Betz *et al*, 2012). Thus, we hypothesized that in addition to the increase in cellular FFA, adrenergic stimulation activates other essential mechanisms that allow for the activation of energy expenditure by FFA. Such pathways, we rationalized, would only be required in the context of intact cells, but not in isolated mitochondria, where FFA alone are strong activators of Ucp1. To test this hypothesis, we generated an experimental system where FFAs were exogenously supplied, and the effect of suboptimal concentrations of norepinephrine (NE) on energy expenditure was tested. Primary brown adipocytes (BA) were differentiated in culture from pre-adipocytes isolated from mouse inter-scapular brown adipose tissue (See Materials and Methods). Cultures of differentiating BA are plated immediately after isolation from the tissue and cannot be re-plated before function is tested. As a result these cultures tend to vary in cell density and maturation capacity, also reflected in variations in basal oxygen consumption rates. To address this variability we have normalized the responses to adrenergic stimuli by reporting the fold increase in oxygen consumption. Fold increase is calculated as the stimulus-induced fold increase in absolute oxygen consumption rates (OCR, pmols O_2/min) over the pre-stimulus value. Both pre- and post-stimuli OCR values take into account the post antimycin OCR value as a reference point (zero).

We first determined the optimal concentration of palmitate that can acutely increase respiration, and thus energy expenditure, in BA (0.4 mM at a ratio of FFA:BSA 4:1) (Supplementary Fig 1A). While used at optimal concentration, this palmitate-induced energy expenditure was not as efficient in inducing mitochondrial depolarization as 1 μM NE, suggesting that the level of uncoupling produced is insufficient to exceed respiratory capacity (Fig 1A). If NE contribution to energy expenditure goes beyond the induction of lipolysis, we expected NE to potentiate the capacity of exogenous FFAs to induce energy expenditure. However, since NE induces the release of endogenous FFAs by lipolysis, we rationalized that to uncover any synergistic interaction between NE and exogenous FFA one would have to establish conditions in which NE is provided at concentrations that do not elicit maximal lipolysis (suboptimal).

To determine suboptimal concentrations of NE, we titrated NE concentrations by measuring energy expenditure as total oxygen consumption rates (OCR). Based on this titration we chose 1 μM NE as a suboptimal concentration given that it elicited < 50% of the energy expenditure induced by 10 μM NE. In support of this notion, NE at 1 μM only yielded a mild depolarization, estimated at < 5 mV (Fig 1A; Supplementary Fig 1B). Remarkably, the effect of optimal concentration of palmitate was potentiated by NE and lead to a six-fold larger depolarization (~30 mV), thereby indicating that NE and palmitate synergize in eliciting depolarization (Fig 1A and B). OCR measurements revealed that this synergistic effect of palmitate on depolarization reflected an increase in uncoupling as it was accompanied by increased oxygen consumption (Fig 1C). Oleate and NE demonstrated a synergistic effect similar to NE and palmitate but with a greater amplitude. NE plus oleate induced a higher degree of $\Delta\psi_m$ depolarization than did NE plus palmitate (Fig 1B and E), but with a similar increase in respiration (Fig 1D).

Reversible mitochondrial fragmentation in stimulated brown adipocytes

The synergistic effect of NE plus FFAs suggested that adrenergic stimulation may facilitate energy expenditure through an additional mechanism beyond the induction of lipolysis.

We examined the possibility that NE induced changes in mitochondrial architecture that could contribute to BAT thermogenesis. Since mitochondrial morphology and dynamics have not been previously described in live BA, we first examined mitochondrial architecture and its relation to the lipid droplets (Fig 2A). The mitochondrial network appeared dense and tightly associated with the droplets. To examine the inter-relationship between function and architecture, BA mitochondria co-stained with TMRE (red) and MTG (Mitotracker Green) were imaged before and after stimulation with NE and palmitate. NE stimulation induced mitochondrial fragmentation (small and spherical mitochondria, partially swelled), while palmitate alone did not elicit any detectable morphology changes (Fig 2C, D and F). This change in mitochondrial morphology was not accompanied by changes in mitochondrial mass, as shown by the lack of changes to total levels of the mitochondrial protein porin (Supplementary Fig 2B). NE-induced fragmentation in primary cultures (Fig 2C) was also demonstrated by scanning electron microscopy of BA cryo-sections (Supplementary Fig 2B). Furthermore, similar changes in architecture were also detected by confocal immunofluorescence and wide-field immunohistochemistry

© 2014 The Authors

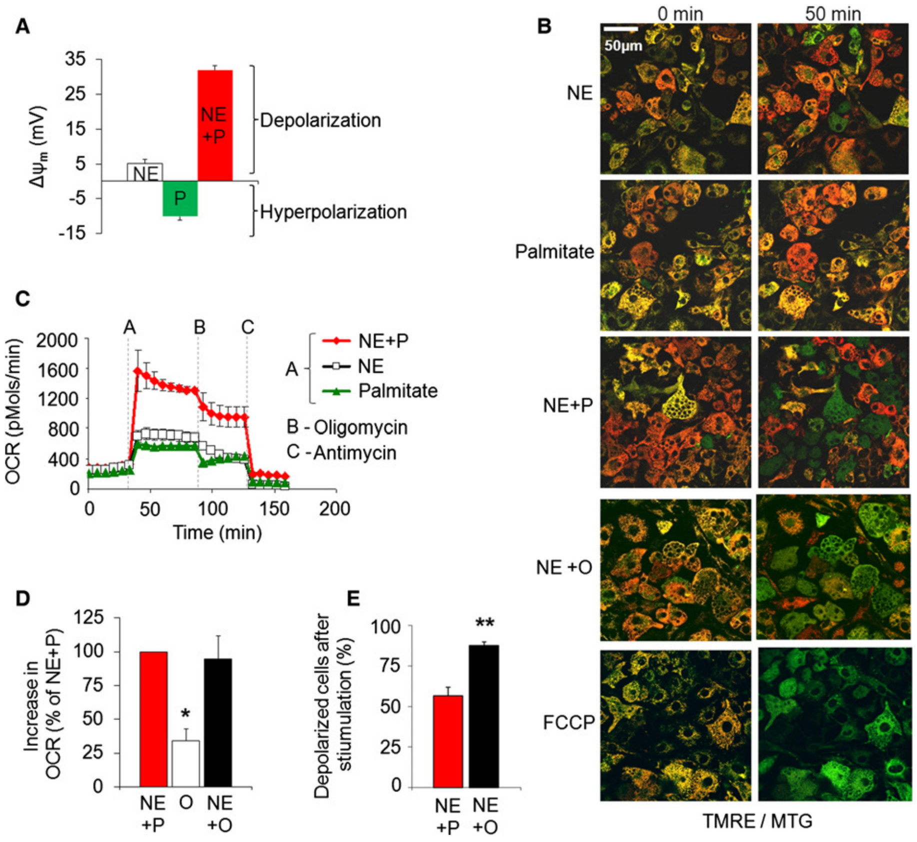

Figure 1. Norepinephrine plus palmitate or oleate synergistically increase thermogenesis in cultured brown adipocytes.

Primary mouse brown adipocytes (BA) were stimulated with a suboptimal concentration of NE (1 μM) in combination with optimal concentrations of free fatty acids complexed to BSA (0.4 mM at 4:1 ratio FFA:BSA).

- A Quantification of relative change in $\Delta\psi_m$ in response to palmitate, NE or their combination. Note that depolarization results in a less negative $\Delta\psi_m$ values in mV. *n* = 17–21 cells per condition. NE = norepinephrine; P = palmitate; NE + P = norepinephrine plus palmitate.
- B Effect of NE plus palmitate or oleate on mitochondrial $\Delta\psi_m$ depolarization. Images of multiple cells stained with TMRE/MTG (only merged images shown). Cells with depolarized mitochondria lose red TMRE staining and become green while cells that maintain $\Delta\psi_m$ show yellow-orange mitochondrial staining. Scale bar, 50 μm.
- C Combination of NE plus palmitate evokes a synergistic increase in oxygen consumption rates. Oxygen consumption rates (OCR, pmols O_2/min) were measured under unstimulated and stimulated conditions as indicated. Dotted lines mark the point of stimulation. Quantification of multiple experiments (not shown) showed that when normalized to NE alone (100%; *n* = 13), NE plus palmitate stimulated oxygen consumption by 294% (*n* = 15; s.e. 26%; *P*-value < 0.0001) and palmitate alone only by 113% (*n* = 15; s.e. 16%; *P*-value > 0.05). Note that in intact brown adipocytes addition of oligomycin may reduce energy expenditure due to reduced availability of ATP required for nutrient utilization. As such, post oligomycin oxygen consumption is not exclusively dependent on mitochondrial uncoupling, and thus it may be underestimating the contribution of mitochondrial proton leak to respiration.
- D Comparing the effect of oleate (O) and palmitate (P) on BAT OCR. When combined with NE, oleate elicits similar respiratory response as compared to NE plus palmitate. Data are showing fold increase in OCR and are normalized to NE plus palmitate response (*n* = 5 each condition). **P* < 0.01.
- E Comparing the effect of oleate and palmitate on mitochondrial depolarization. When combined with NE, oleate is a more efficient inducer of $\Delta\psi_m$ depolarization as compared to NE plus palmitate. Quantification of the fraction of cells that undergo $\Delta\psi_m$ depolarization is shown. Depolarization was defined as a 30% drop in TMRE fluorescence intensity. *n* = 24 (NE + palmitate), *n* = 27 (NE + oleate). ***P* < 0.01.

of mitochondrial TOMM20 in interscapular BAT (iBAT) from cold-exposed mouse (Fig 2H). Specifically, BAT mitochondria from mice under thermo-neutrality were elongated and tubular, whereas BAT mitochondria from mice were spherical and large after 4 h cold exposure, consistent with fragmentation and swelling (Figs 2H and 3E). An increase in the average individual mitochondrion size observed under EM was clearer in the confocal images of TOMM20 immunofluorescence (Figs 2H, 3E and F). The remarkable enlargement of mitochondria *in vivo* was not apparent in NE-treated BA in culture. This raised the possibility that the full effect consisting of fragmentation and swelling requires the synergistic interaction of NE and FFAs. Remarkably, in addition to fragmentation, the combination of NE plus palmitate increased the diameter of each small mitochondrial sphere (Fig 2E and G, 3A–C). In primary BA, mitochondria were visualized with MTG exclusively, as TMRE completely disappeared due to large $\Delta\psi_m$ depolarization (Fig 2E). Thus, adrenergic BA activation caused a transition in mitochondrial shape from elongated units to fragmented, and then to spherical shapes, visualized as "rings" of MTG staining (Fig 3A). The observation that FFA alone did not elicit any fragmentation indicates that NE-induced lipolysis is unlikely to be part of the pathway by which NE mediates fragmentation.

 © 2014 The Authors

Since MTG covalently and primarily binds to the mitochondrial inner membrane (Presley *et al*, 2003), the MTG rings observed after depolarization under the combination of NE and palmitate could represent "donuts" resulting from fusion of mitochondrial tips, instead of swelled or spherical mitochondria (Fig 3D). Donut mitochondria are typically observed in cells treated with the uncoupler FCCP (Liu & Hajnoczky, 2011). To differentiate between the two possibilities, the mitochondrial matrix was labeled with matrix-targeted DsRed and photoactivatable (PA)-GFP. The combination of the two probes allowed for the visualization of the boundaries of individual mitochondria (Twig *et al*, 2006). The appearance of spheres using these fluorescent probes confirmed that the observed

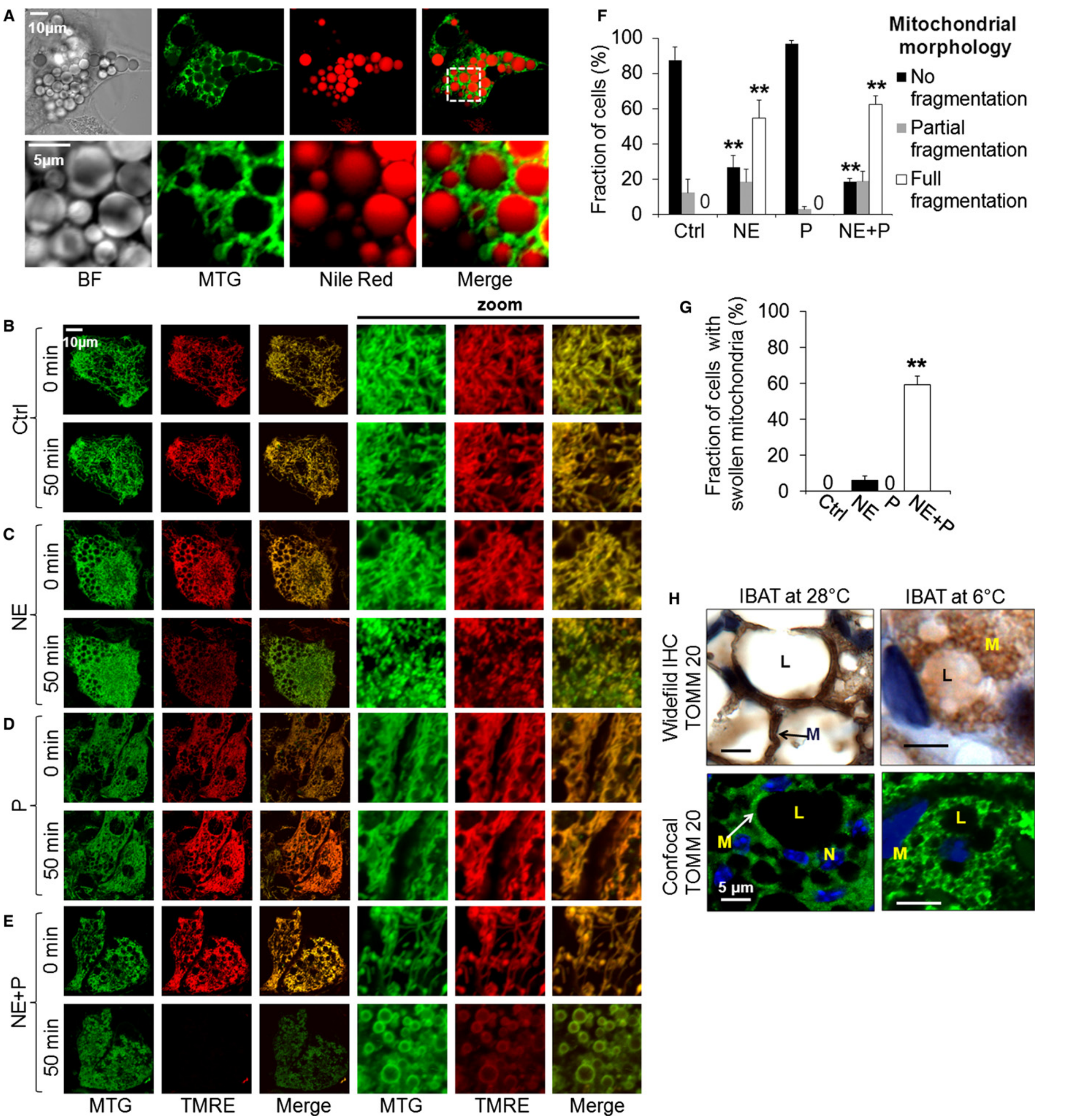

circular mitochondria were indeed large sphere-shaped mitochondria and not donut-like mitochondria (Fig 3B and D). In contrast, the uncoupler FCCP induced true donut-shaped structures in primary BA (Fig 3D).

Ultrastructural analysis of mitochondria from cold-exposed mice iBAT reflected the confocal microscopy images showing robust architectural changes including a transition to circular structures with large diameters and intact cristae structure (Fig 3E and F). The fragmented round mitochondria in transmission EM represent spheres rather than cut tubules in a transverse orientation, as demonstrated by scanning EM (Fig 3G).

Change in architecture is not associated with cell death

Mitochondrial fragmentation and enlargement (swelling) has previously been associated with depolarization and apoptosis. To determine whether the fragmentation and depolarization in BA could represent apoptosis, we treated cells with NE plus palmitate for 2 h and studied them 24 h later, as apoptosis can take up to 20 h to complete. Examination of BA after 24 h revealed complete recovery of mitochondrial $\Delta\psi$, as well as of mitochondrial morphology (Supplementary Fig 3A). To test for functional recovery, primary BA were treated once with NE plus palmitate for a period of 2 h, and then tested for their ability to increase respiration to NE that was added 24 h afterwards. First treatment with NE + palmitate did not affect mitochondrial oxygen consumption and $\Delta\psi_m$ response to a second treatment with NE (Supplementary Fig 3B and C, and Supplementary Table I).

Fragmentation and depolarization spread across the cell in an organized manner

To obtain a better understanding of the depolarization, fragmentation and mitochondrial spheroid formation processes, we characterized the temporal and spatial relationship of these mitochondrial events. Time-lapse imaging of multiple primary BAs showed that mitochondrial depolarization and fragmentation occurred in a spatially organized manner that started at one pole of the cell and gradually propagated to its opposite side, until transition had encompassed the entire cell (Fig 4A and B). This propagation is visually apparent by the change in mitochondrial TMRE/MTG fluorescence from yellow/orange (hyperpolarized mitochondria, high TMRE/MTG ratio) to green (depolarized mitochondria). Fragmentation and depolarization events took place within minutes (initial depolarization: 3.6 min, s.e. 0.7; full fragmentation 5.3 min, s.e. 0.7). The average speed of the wave was calculated to be 12 μm/min under NE plus palmitate (s.e. 2.0). After the wave had passed and mitochondria had undergone full fragmentation, additional depolarization took place (Fig 4C and D), thus suggesting a potential role for mitochondrial fragmentation in uncoupling and thermogenesis.

We also imaged the recovery of $\Delta\psi_m$ after activation with NE followed by taking an extended time-lapse movie after washing out the NE from the media. During the recovery phase, repolarization occurred in a reversed wave pattern (Fig 4F).

Mitochondrial fusion is inhibited in stimulated brown adipocytes

In several cell types, mitochondria were shown to undergo continuous cycles of fusion and fission. Mitochondrial fragmentation can be the result of increased mitochondrial fission, decreased mitochondrial fusion or both. However, the occurrence of mitochondrial fusion and its response to adrenergic stimulation have not been reported in primary brown adipocytes so far. To test for the occurrence of fusion in BA, individual mitochondria were photo-labeled with $_{mt}$PAGFP and tracked over time (Twig *et al*, 2006, 2008a). Z-stack-imaging and 3D reconstructions ensured that mitochondria did not travel out of focus and enabled us to determine when two mitochondria were separated physically. Fusion events appeared as sudden increases in $_{mt}$PAGFP area, indicating that the $_{mt}$PAGFP of a photo-converted mitochondrion spread into the matrix of a neighboring one (Supplementary Fig 4). This is the first time mitochondrial dynamics is demonstrated in BA.

To examine the rate of mitochondrial fusion under basal and stimulated conditions, we used the $_{mt}$PAGFP fusion assay (Karbowski *et al*, 2004; Molina *et al*, 2009). In brief, $_{mt}$PAGFP in an area of 15–20% of the cell was photo-converted and the cell was imaged over time for up to 70 min (Fig 5A). In this assay cellular mitochondrial fusion activity is measured as the rate at which the photo-converted green signal is shared across the mitochondrial

◀ **Figure 2. Brown adipocyte stimulation induces mitochondrial fragmentation.**

A Mitochondrial morphology and lipid droplets. BA stained with the mitochondrial inner membrane dye, MTG, and the lipid droplet dye, Nile Red. Note the close proximity of the multilocular lipid droplets (red) and mitochondria (green). BF = bright field. White square indicates area of zoom. Scale bar, 10 and 5 μm for the zoom images.

B–E Brown adipocytes stained with the mitochondrial dyes TMRE and MTG and imaged before (0 min) and after (50 min) stimulation. Note the differences in mitochondrial morphology; NE induces mitochondrial fragmentation, while NE plus palmitate also induce swelling. Palmitate (P) alone does not induce any morphological changes. Ctrl = control. Scale bar, 10 μm.

F Quantification of mitochondrial fragmentation in response to stimulation. Mitochondrial morphology in each cell was classified into one of the following three subtypes: fully fragmented, partially fragmented or not fragmented. The fraction of the cell population that shows each morphology subtype is presented. Zero labels indicate bars where quantification yielded zero. Note that NE alone or in combination with palmitate reduces the number of BA with normal morphology, while increasing the number of BA with partial or full fragmentation. n = 3–4 independent experiments with 40–49 cells per condition. $^{**}P < 0.01$.

G Quantification of mitochondrial swelling. Mitochondrial swelling in each cell was assessed as being present or absent. Zero indicates bars where quantification yielded zero. Note that only NE in combination with palmitate induces mitochondrial swelling. n = as in (F). $^{**}P < 0.01$.

H Immunohistochemistry (IHC) images of the mitochondrial protein TOMM20 in interscapular BAT (iBAT) from warm- and cold-adapted animals. In the wide-field images TOMM20/mitochondria show brown labeling, while in the confocal images TOMM20/mitochondria show green labeling (Alexa 488); in both sets of images nuclei are blue (Hoescht staining). Arrows indicate mitochondria (M = mitochondrion; L = lipid droplet; N = nucleus). Note the fragmented and swollen mitochondria in iBAT from the cold exposed animal. Scale bar, 5 μm.

 © 2014 The Authors

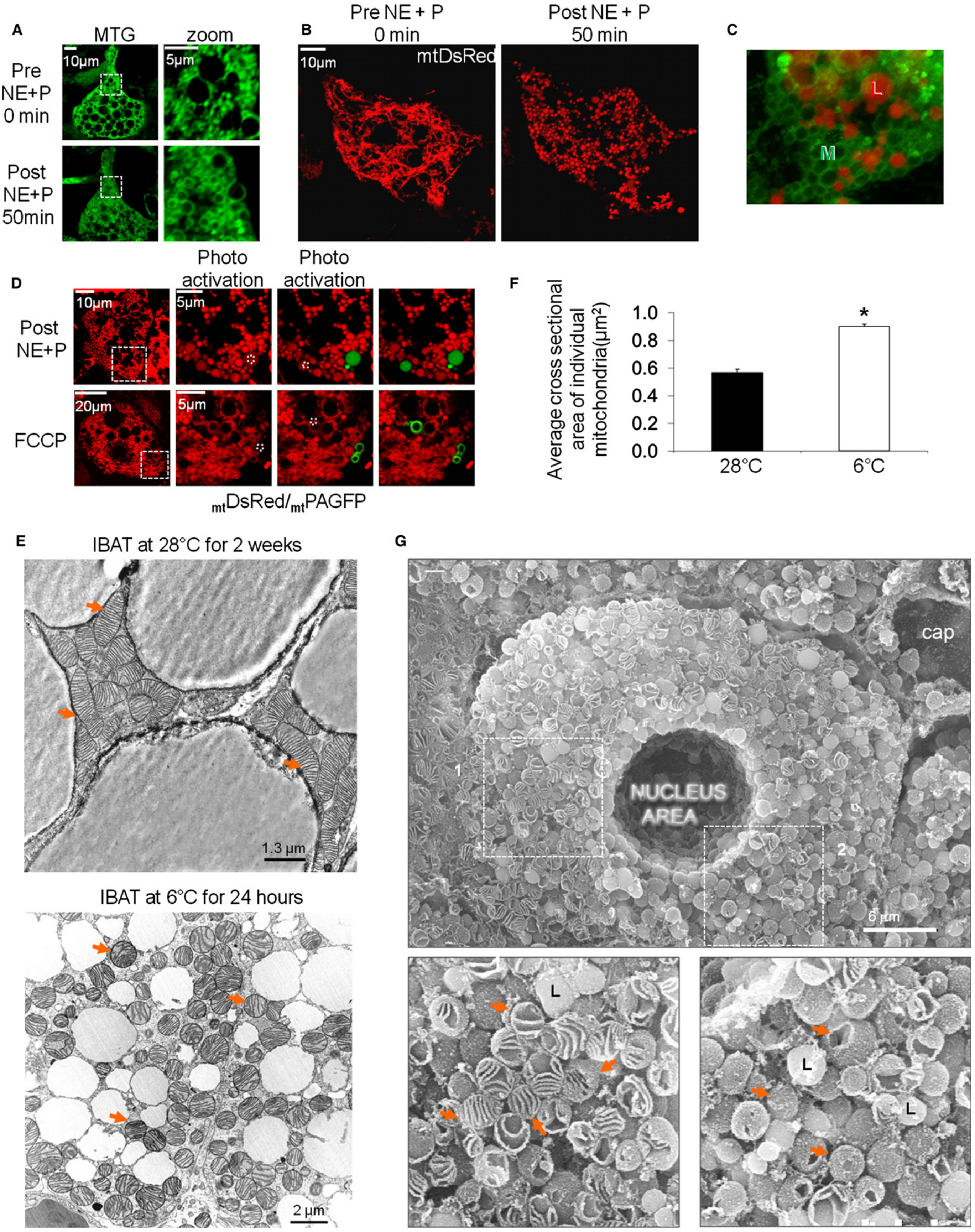
A
MTG
zoom
Pre NE+P 0 min
Post NE+P 50min
10μm
5μm
B
Pre NE + P 0 min
Post NE + P 50 min
10μm
mtDsRed
C
L
M
D
Photo activation
Photo activation
Post NE+P
FCCP
10μm
5μm
20μm
5μm
mtDsRed/mtPAGFP
F
Average cross sectional area of individual mitochondria(μm²)
1.0
0.8
0.6
0.4
0.2
0.0
*
28°C
6°C
E
IBAT at 28°C for 2 weeks
1.3 μm
IBAT at 6°C for 24 hours
2 μm
G
cap
NUCLEUS AREA
1
2
6 μm
L
L
L

population, a phenomenon that is accompanied by the reduction in its average intensity (Fig 5D). $_{mt}$PAGFP fluorescence intensity was measured and found to decrease rapidly and spread over a larger area in control as well as palmitate-stimulated cells (Fig 5A and D). In comparison, when cells were stimulated with either NE alone or in combination with palmitate, $_{mt}$PAGFP fluorescence decay was significantly slower and spreading throughout each cell was almost non existent (Fig 5B and C). In addition, mitochondrial movement appeared to be diminished. Quantification of the decay rate in multiple experiments showed significant decrease in fusion rates of NE alone and NE plus palmitate-stimulated cells. Palmitate alone did not alter mitochondrial dynamics (Fig 5D).

Mechanism of mitochondrial fusion inhibition during brown adipocyte stimulation of energy expenditure

Opa1 is a mitochondrial pro-fusion protein that exists in several forms. FCCP-induced depolarization was previously shown to induce rapid processing of long Opa1 to short Opa1 forms that do not mediate fusion per se (Guillery *et al*, 2008). We hypothesized that mitochondrial depolarization during BA activation results in Opa1 cleavage. This was tested by Western blot analysis of total lysates from BA before and after activation. In the basal state, we identified Opa1 pro-fusion forms (long, ~100 KDa) and the short forms (fusion-deficient, ~85 KDa) (Fig 5E). In primary BA acutely stimulated with NE plus palmitate, the lower band showed a significant increase while decrease in the top band was of borderline significance ($P = 0.054$) (Fig 5F and G). Hence, NE plus palmitate induce Opa1 cleavage, thereby increasing the proportion of fusion-deficient short forms. In agreement with these data, inhibition of fatty acid-induced depolarization using the lipolysis inhibitor, Orlistat, prevented Opa1 cleavage. This is shown by the absence of changes in the proportion of the shorter versus the longer Opa1 forms measured by Western blot of total lysates from primary BA (Fig 7D and F). This result shows that depolarization mediated by released FFA from lipolysis is required for Opa1 cleavage.

Role of mitochondrial fission in NE-induced fragmentation

The fast transition from elongated to fragmented mitochondria strongly suggested that reduced mitochondrial fusion keeps BA mitochondria fragmented for the duration of the activation but may not account for the initial induction of fragmentation upon adrenergic stimulation. We rationalized that mitochondrial fission contributed to this component. To determine the contribution of mitochondrial fission to NE-induced fragmentation, we studied the mitochondrial fission protein, Drp1, in BA activation. Drp1 is a cytosolic protein recruited to the mitochondrial outer membrane upon activation. We found a sharp increase in Drp1 punctae co-localization with mitochondria following BA activation with NE, suggesting increased Drp1-mediated fission (Fig 6A). Drp1 fission activity is primarily regulated by post-translational modifications. Phosphorylation of the ubiquitously expressed human Drp1 isoform in Ser600 has been shown to promote Drp1-mediated fission (Han *et al*, 2008). The mechanism by which Ser600 phosphorylation increases fission is through increased Drp1 recruitment from the cytosol to the mitochondria as punctae (Han *et al*, 2008). Importantly, this serine residue is present in mouse Drp1, suggesting that phosphorylation is a mechanism conserved among these species.

While total Drp1 content did not change during 1 h stimulation of BA with NE in primary mouse BA, there was a marked increase in the phosphorylation of the mouse serine equivalent to human Drp1 Ser600 (Fig 6B and C).

Ser600 of the brain-specific (non-ubiquitous) Drp1 isoform is phosphorylated by Protein Kinase A (PKA) (Chang & Blackstone, 2007; Cereghetti *et al*, 2008). During adrenergic activation of thermogenesis, β3-adrenergic receptors induce the activation of PKA via cAMP dependent pathway (Freedman & Lefkowitz, 1996; Hall, 2004). Since we find that the synergistic response to NE and FFA is mediated through the β3-adrenergic receptor pathway (Supplementary Fig 5A–C), we rationalized that PKA may play a role in the induction of fragmentation during BA activation. To test this hypothesis, we treated primary BA with the PKA inhibitor H89 and measured Drp1 phosphorylation in the equivalent serine to

Figure 3. Characterization of sphere-shaped mitochondria in activated brown adipocytes.

A BA stained with MTG, imaged before and after NE plus palmitate stimulation. Note transition from filamentous mitochondria to fragmented sphere-like mitochondria. Depolarized mitochondria retained MTG dye which binds to inner membrane proteins leaving the matrix unstained. Scale bars, 10 and 5 μm for the zoom images.

B BA expressing matrix-targeted DsRed and imaged before and after stimulation with NE plus palmitate. Note the transition to fragmented and spheroid mitochondria. Scale bar, 10 μm.

C Orientation of lipid droplets (red) to the sphere-shaped mitochondria (green). MTG and Nile Red staining of a BA 50 min after treatment with NE plus palmitate.

D Fragmented mitochondria are spheres and not donuts. BA expressing $_{mt}$DsRed and $_{mt}$PAGFP (both targeted to matrix) were treated with NE plus palmitate or FCCP for 50 min. Dotted white squares represent areas of photo-conversion with a 2-photon laser at 750 nm which gives 350 nm at the focal plane. When $_{mt}$PAGFP is photo-converted it fills the entire lumen of an individual mitochondrion thus defining its borders. Note that for NE plus palmitate treatment, $_{mt}$DsRed and $_{mt}$PAGFP fill the entire mitochondrial structures, indicating sphere-like morphology, and not donut-like as is the case for FCCP. White squares indicate zoom area. Scale bar, 10 and 5 μm for the zoom images on the top and 20 and 5 μm for the zoom images on the bottom.

E Transmission electron microscopy (TEM) of mitochondrial morphology in iBAT from warm- and cold-adapted animals. Arrows indicate mitochondria. Note the fragmented and swollen mitochondria in BAT from the cold-exposed animal. Scale bar, 1.3 μm for the iBAT at 28°C image, and 2 μm for the iBAT at 6°C images.

F Quantification of mean mitochondrial area (in μm^2) of 25 TEM images taken from BAT of 2 animals per group (not the same animals shown in Fig 2E). *$P < 0.05$.

G Scanning electron microscopy of iBAT from mice exposed to 6°C for 24 h. Scanning EM confirms that round-shaped mitochondria observed in TEM are not transverse sliced tubules but rather spheres. Red arrows point to mitochondria of which some have remained sealed and some have broke open. Lipid droplets are labeled L and mitochondria are indicated with arrows. Scale bar, 6 μm.

© 2014 The Authors

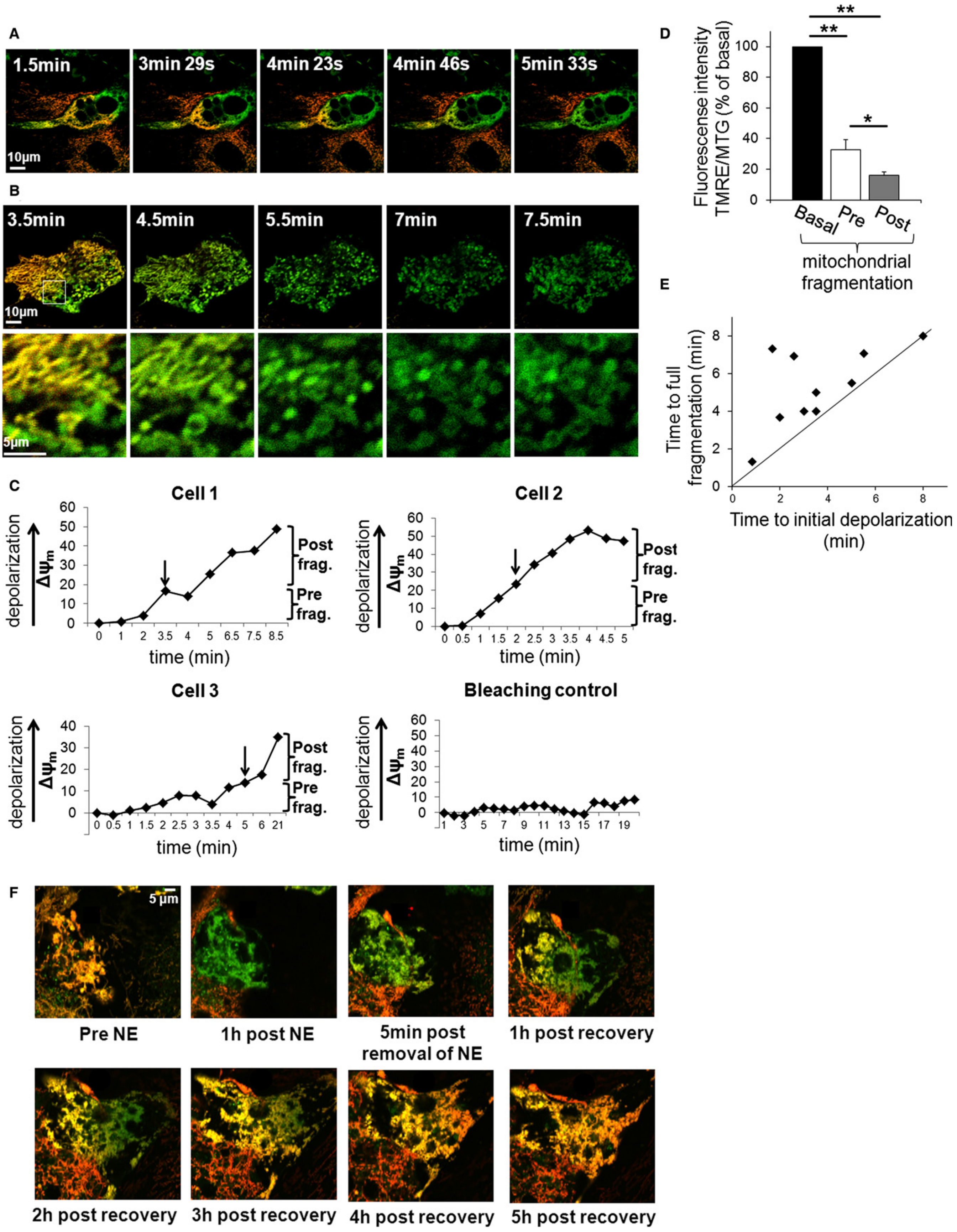
A
1.5min
3min 29s
4min 23s
4min 46s
5min 33s
10μm
B
3.5min
4.5min
5.5min
7min
7.5min
10μm
5μm
C
Cell 1
Cell 2
Cell 3
Bleaching control
depolarization
ΔΨm
time (min)
Post frag.
Pre frag.
D
Fluorescense intensity TMRE/MTG (% of basal)
Basal
Pre
Post
mitochondrial fragmentation
E
Time to full fragmentation (min)
Time to initial depolarization (min)
F
5 μm
Pre NE
1h post NE
5min post removal of NE
1h post recovery
2h post recovery
3h post recovery
4h post recovery
5h post recovery

human Ser600. PKA inhibition blunted NE-mediated Drp1 phosphorylation of this serine residue (Fig 6B and C), mitochondrial fragmentation and depolarization (Fig 6D–F) of primary mouse BA, thus demonstrating that NE-mediated increase in PKA activation promotes Drp1 phosphorylation in the equivalent serine to Ser600, and likely activates its fission activity as reported in other cell types (Han *et al*, 2008). In all, these data show that NE increases mitochondrial fission through activation of Drp1, which is associated with Drp1 phosphorylation that requires PKA activity.

Activation of Drp1-mediated mitochondrial fission in stimulated brown adipocytes is upstream of mitochondrial depolarization

Previous work demonstrated that FCCP is able to induce mitochondrial fission by increasing Drp1 recruitment to the mitochondria (Cereghetti *et al*, 2008). Given that depolarization is the first event during BA activation (Fig 4E), we wanted to test whether depolarization was required for the initial fragmentation mediated by Drp1. To inhibit endogenous mitochondrial depolarization, we treated BA with an inhibitor of lipolysis, Orlistat. Inhibition of lipolysis blunts FFA release from the lipid droplets and is expected to prevent Ucp1 activation. We found that inhibition of lipolysis by Orlistat prevented mitochondrial depolarization, as shown by TMRE/MTG staining, but failed to prevent fragmentation (Fig 7A–C). This result suggests that NE activates Drp1-mediated fission (through phosphorylation) independently of a decrease in $\Delta\psi_m$. As we described in Fig 1B, the response to suboptimal NE concentrations is heterogeneous with some cells not depolarizing. Consistent with these findings, Drp1 phosphorylation in the residue sensitive to PKA activity (equivalent to Ser600) was not prevented when lipolysis was inhibited by Orlistat treatment (Fig 7D and E).

Mitochondrial fragmentation is required for uncoupled respiration in hormonally stimulated BA

To determine the functional role of mitochondrial fragmentation in NE-induced energy expenditure in BA, fission was inhibited using transient Drp1 Dominant Negative mutant (DN, K38A) overexpression. The capacity of BA to respond to NE alone or in combination with FFA was quantified by measuring stimulated oxygen con sumption rates (OCR) and mitochondrial membrane potential depolarization. Overexpression of Drp1 DN was limited to 2 days to avoid compromising respiratory chain function, cell differentiation or viability. Indeed, Drp1 DN overexpression for 2 days did not change maximal respiratory capacity or Ucp1 expression levels and lipid droplet number (Supplementary Figs 6A–C, 6G and H). Drp1 DN induced mitochondrial elongation and hyperfusion in unstimulated BA, as shown by very long mitochondrial filaments and enlarged mitochondrial structures, as previously reported for other cell types (ball and thread structures) (Twig *et al*, 2008a) (Fig 8A and B). Drp1 DN blunted mitochondrial fragmentation induced by NE plus palmitate (Fig 8A), thus supporting a role for Drp1 in the observed fission.

Remarkably, Drp1 DN-expressing cells exhibited a marked decrease in the respiratory response (Fig 8C–F). Consistent with reduced BA activation of energy expenditure, Drp1 DN caused a marked decrease in the number of cells in which $\Delta\psi_m$ depolarization was detectable (Fig 8G). Thus, both respiration and $\Delta\psi_m$ measurements confirmed that inhibition of fragmentation by Drp1 DN decreased the ability of BA to induce mitochondrial uncoupling after activation.

Enhanced BA activation in a model of forced fragmentation

Based on the effect of Drp1 DN on NE-induced respiration and the synergistic effect of NE plus FFA, we hypothesized that mitochondrial fragmentation was enhancing the capacity of FFA to induce uncoupled respiration in the BA. A prediction of this hypothesis is that increased mitochondrial fragmentation will enhance the capacity of exogenous FFA to activate energy expenditure in BA. To test this prediction, we reduced the rate of mitochondrial fusion by knocking down the pro-fusion protein Mitofusin 2 (Mfn2) using transduction of adenovirus encoding for 5 different microRNAs against Mfn2 or scrambled at the UTR of GFP (Supplementary Fig 6D) (Sebastian *et al*, 2012). Mfn2 knock down (KD) BA showed fragmented and swollen mitochondrial morphology

◀ **Figure 4. Subcellular spatial and temporal features of the induction of uncoupling, fragmentation and swelling.**

A A wave of mitochondrial depolarization precedes fragmentation. $\Delta\psi_m$ depolarization in BA stained with TMRE/MTG and stimulated with NE plus palmitate. Time lapse imaging was started after the cell started to depolarize at one pole. Mitochondria that depolarize lose red TMRE staining and become green. Note how the cell depolarizes from one pole to the other in a wave-like fashion. Time indicates time lapsed after NE plus palmitate stimulation. Scale bar, 10 μm.

B A second example as shown in (A). Note that $\Delta\psi_m$ depolarization precedes fragmentation which in turn precedes swelling. Scale bars, 10 and 5 μm for the zoom images.

C Time chart of $\Delta\psi_m$ depolarization reveals that depolarization continues after mitochondrial fragmentation is completed. Cells 1–3 represent the different types of responses that were measured following stimulation with NE plus palmitate. Bleaching control represents a cell that was not stimulated in which we tested the effect of imaging on $\Delta\psi_m$; note that there is no major change. Arrows indicate time of full mitochondrial fragmentation. Note that depolarization continues after fragmentation. Note that Cell 1 shows fast depolarization and fragmentation while cell 3 has a delayed response.

D Summary of depolarization occurring pre- and post-mitochondrial fragmentation. Note that both values are in comparison to the TMRE fluorescence intensity prior to depolarization. $^{**}P < 0.01$, $^{*}P < 0.05$. $n = 8$.

E Quantification of initial $\Delta\psi_m$ depolarization versus fragmentation. To determine the timing of depolarization versus fragmentation, BA were time-lapse imaged and analyzed for time to depolarization and fragmentation ($n = 10$). Cells above diagonal line fragment after depolarization. Note that $\Delta\psi_m$ depolarization always precedes fragmentation.

F $\Delta\psi_m$ repolarization in BA stained with TMRE/MTG. The cell was stimulated with NE for 1 h, followed by wash out recovery. Time lapse imaging was started prior to stimulation. Mitochondria that depolarize lose red TMRE staining and become green. Note that the cell repolarizes from one side to the other; slower albeit similar to depolarization. Scale bar, 5 μm.

 © 2014 The Authors

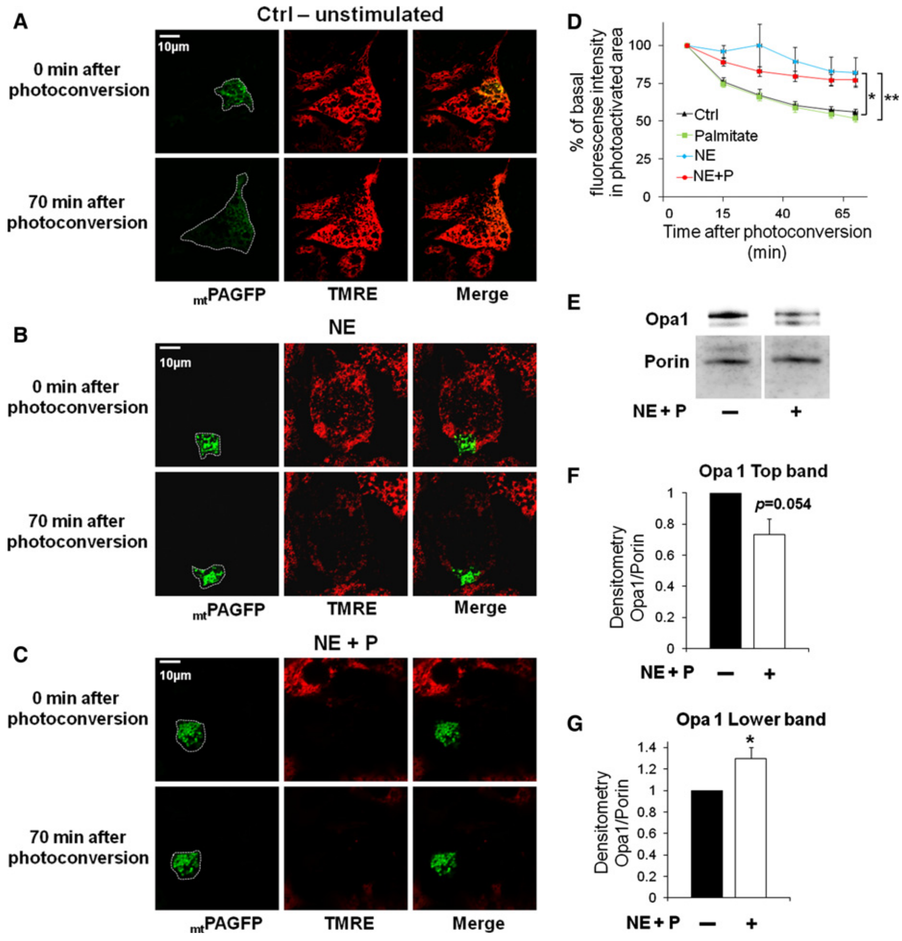

Figure 5. Mitochondrial fusion decreases in activated brown adipocytes.

Cells expressing $_{mt}$PAGFP and stained with TMRE. Fusion of the photo-converted fraction (10%) with the rest of the mitochondrial network dilutes activated $_{mt}$PAGFP and leads to a reduction in fluorescence intensity. Images are representative z-projections. Time 0 indicates the time of photo-conversion. Photo-conversion was performed 30 min after stimulation with NE and/or palmitate or control media.

A Mitochondrial fusion assay in an unstimulated cell. Note the decrease in fluorescence intensity and increase in $_{mt}$PAGFP area at 70 min. White line indicates area of $_{mt}$PAGFP in the cell. Scale bar, 10 μm.

B Mitochondrial fusion assay in NE-stimulated cell. Note that mitochondrial fusion and movement are negligible. Scale bar, 10 μm.

C Mitochondrial fusion assay in NE plus palmitate-stimulated cell. Note that this cell showed depolarized $\Delta\psi_m$ rapidly after stimulation and that mitochondrial fusion and movement was abolished as in (B). Scale bar, 10 μm.

D Fusion kinetics is decreased in NE as well as NE plus palmitate-treated cells. Decreased fluorescence intensity over time after $_{mt}$PAGFP conversion. Control and stimulated cells were photo-converted in a portion of the mitochondrial network. The dilution over time of $_{mt}$PAGFP across the mitochondrial network was used as a measure of mitochondrial fusion. n = 30 (control), n = 15 (palmitate), n = 11 (NE), n = 26 (NE plus palmitate). *$P < 0.05$, **$P < 0.01$.

E Western blot showing Opa1 long (top band) and short (lower band) forms 50 min after NE plus palmitate stimulation. Porin is used as a loading control. Note that the top band decreases in intensity while the lower band increases with stimulation.

F Densitometry quantification of Opa1 long form (top band) normalized to porin. n = 4 per condition.

G Densitometry quantification of Opa1 short form (lower band) normalized to porin. n = 4 per condition. *$P < 0.05$.

Source data are available online for this figure.

under unstimulated conditions (Fig 9A and B). As compared to control (scrambled) BA, Mfn2 knock-down (KD) BA showed the same increase in oxygen consumption and mitochondrial depolarization when induced by NE (Fig 9C–E). These results suggest that forced fragmentation induced by Mfn2 KD does not alter the capacity of NE to induce uncoupling. The lack of effect on NE-induced energy expenditure was consistent with comparable lipolysis rates detected in Mfn2 KD BA after NE stimulation (Fig 9F), as well as similar level of mitochondrial depolarization (data not shown).

We then proceeded to test whether fragmentation induced by Mfn2 KD could, on its own, synergize with FFA even in the absence of NE. Specifically, we tested the feasibility that fragmentation induced by Mfn2 KD could elicit energy expenditure when exposed to low concentrations of FFA, that are similar to those found in the plasma of mice after a meal.

© 2014 The Authors

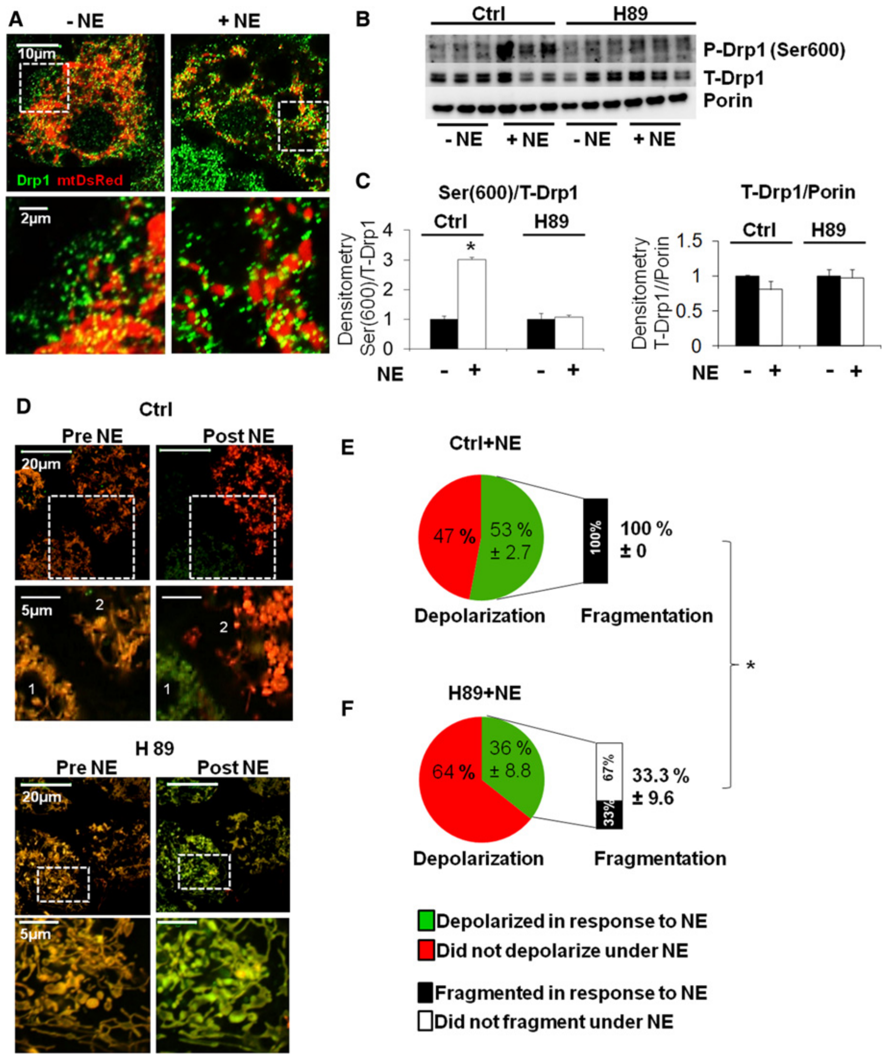

Figure 6. Mitochondrial fragmentation in activated brown adipocytes is controlled by PKA with Drp1 as downstream target.

A Immunostaining of Drp1 in control versus NE-stimulated (50 min) cells. Note the change in mitochondrial morphology and the increase in green Drp1 punctae on mitochondria with stimulation. White dashed square shows the region of the cell where higher resolution image was taken from. Mitochondria show red fluorescence through the expression of matrix targeted Ds-Red protein. Scale bars, 10 and 2 μm for the zoom images.

B Western blot for total and phosphorylated Drp1 (Ser600) in BA treated with PKA inhibitor (H89, 5 μM) and control. Porin is used as a loading control. Note that the increase in Ser600 phosphorylation with NE stimulation (50 min) is inhibited by H89. *n* = 3 per condition.

C Quantification of Western blot analyses for phosphorylated Drp1 (Ser600) and total Drp1 shown in (B), normalized to total (T-) Drp1 and Porin respectively. $^*P < 0.0002$.

D Fragmentation depends on PKA, but not on $\Delta\psi_m$ depolarization. Brown adipocytes stained with the mitochondrial dyes TMRE and MTG and imaged before (0 min) and after (50 min) stimulation with NE (only merged images shown). BA showing mitochondrial depolarization lose red TMRE staining and become green, while cells that maintain $\Delta\psi_m$ show yellow-orange mitochondria. BA pre-incubated with a low concentration (5 μM) of the PKA inhibitor H89 are compared to control BA (vehicle treated). Note that mitochondrial fragmentation induced by NE is inhibited under PKA inhibition. Note that a cell in the control group (labeled 1) depolarized and fragmented in response to NE, while a cell labeled 2 also fragmented, but did not depolarize. Note that in cells treated with H89 the initial phase of depolarization occurred, but mitochondria did not fragment. Scale bars, 20 and 5 μm for the zoom images.

E, F Quantification of the fraction of cells that undergo $\Delta\psi_m$ depolarization in response to NE as indicated. The fraction of mitochondrial fragmentation was quantified in cells in which depolarization occurred. Note that H89 reduced the fraction of cells that fragmented their mitochondria in response to NE. H89 inhibited fragmentation independent of mitochondrial depolarization. (n = 263 cells). (Numbers = Mean% ± s.e.), ($P < 0.0001$).

Source data are available online for this figure.

Indeed low concentrations of palmitate and oleate (0.1 and 0.2 mM each) elicit a larger respiratory response in Mfn2 KD BA as compared to control cells (Fig 9C–E). Like Drp1 DN, Mfn2 knockdown did not affect BA differentiation, as shown by the absence of changes in total Ucp1 protein levels and maximal capacity of mitochondrial oxygen consumption stimulated by FCCP (Supplemen-

 © 2014 The Authors

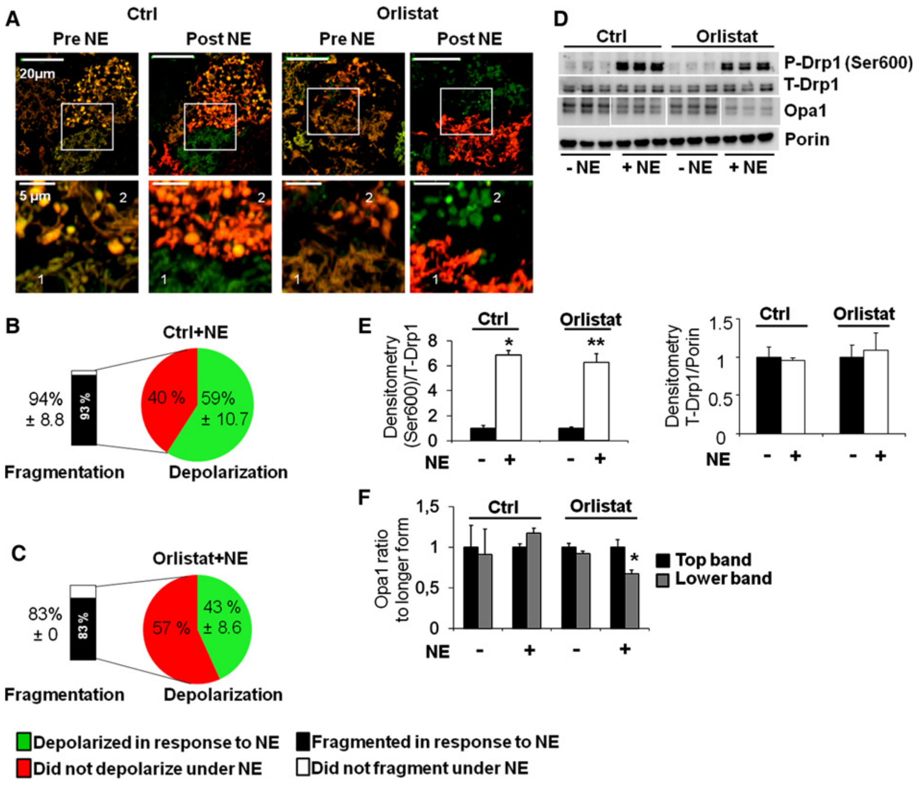

Figure 7. Mitochondrial fragmentation is independent of lipolysis.

A Cells stained with TMRE and MTG and imaged before and after (50 min) NE stimulation −/+ the lipolysis inhibitor Orlistat (100 μm). Cells pre-incubated with Orlistat are compared to control cells (vehicle). Note that Orlistat-treated cells still show fragmented mitochondria after NE stimulation. Scale bars, 20 and 5 μm for the zoom images.

B, C Quantification of the fraction of cells that undergo $\Delta\psi_m$ depolarization in response to NE as indicated. The fraction of mitochondrial fragmentation was quantified in cells in which depolarization did not occur. Note that Orlistat did not reduce significantly the fraction of cells that fragmented their mitochondria in response to NE. Orlistat reduced the fraction of the cells that undergo depolarization in response to NE, but did not affect mitochondrial fragmentation. Numbers are Mean % of cells ± s.d.

D Western blot for total Drp1, phosphorylated Drp1 (Ser600) and Opa1 under control and Orlistat. Porin is used as a loading control. Note that Orlistat does not reduce the increase in Ser600 phosphorylation with NE stimulation (50 min). $n = 3$ per condition.

E Quantification of Western blot analyses for phosphorylated Drp1 (Ser600) and total (T-) Drp1 shown in (D), normalized to T-Drp1 and Porin respectively. $*P < 0.0002$ and $**P < 0.002$.

F Quantification of Western blot analyses for Opa1 (Top and Lower bands) shown in (D), normalized to Porin and Top band. $*P < 0.04$. Note that Orlistat prevents Opa1 cleavage after NE stimulation.

Source data are available online for this figure.

tary Fig 6D–F). In contrast to Drp1 inhibition, Mfn2 KD thus increased the capacity of exogenous FFA to promote energy expenditure in BA.

Discussion

Previous studies have documented an association of mitochondrial fragmentation with excess nutrient environment, suggesting a potential role for mitochondrial dynamics in nutrient utilization and energy expenditure (Liesa & Shirihai, 2013; Molina *et al*, 2009; Zhang *et al*, 2010). Here we demonstrate that mitochondrial fragmentation is a physiological response that enhances mitochondrial uncoupling and energy expenditure in brown adipocytes. As such, stimulation of brown adipose tissue represents the first physiological and hormonal responses associated with complete mitochondrial fragmentation. This study not only provides further evidence that mitochondrial fission is not deleterious per se (Twig & Shirihai, 2011) but it also presents a novel pathway regulating energy expenditure and mitochondrial uncoupling beyond lipolysis, FFA release and Ucp1 activation in BA.

Exogenous FFAs are able to amplify energy expenditure in NE-stimulated BA

The initial finding of our study was that hormonal stimulation of brown adipocytes with suboptimal concentrations of NE allowed exogenous FFA to further stimulate energy expenditure and mitochondrial uncoupling. This result is consistent with other findings showing that increased FFA availability to UCP1 through increased lipolysis is not the exclusive mechanism by which NE activates mitochondrial uncoupling in BA. In agreement with this, addition of exogenous FFAs alone was not as efficient in inducing mitochondrial

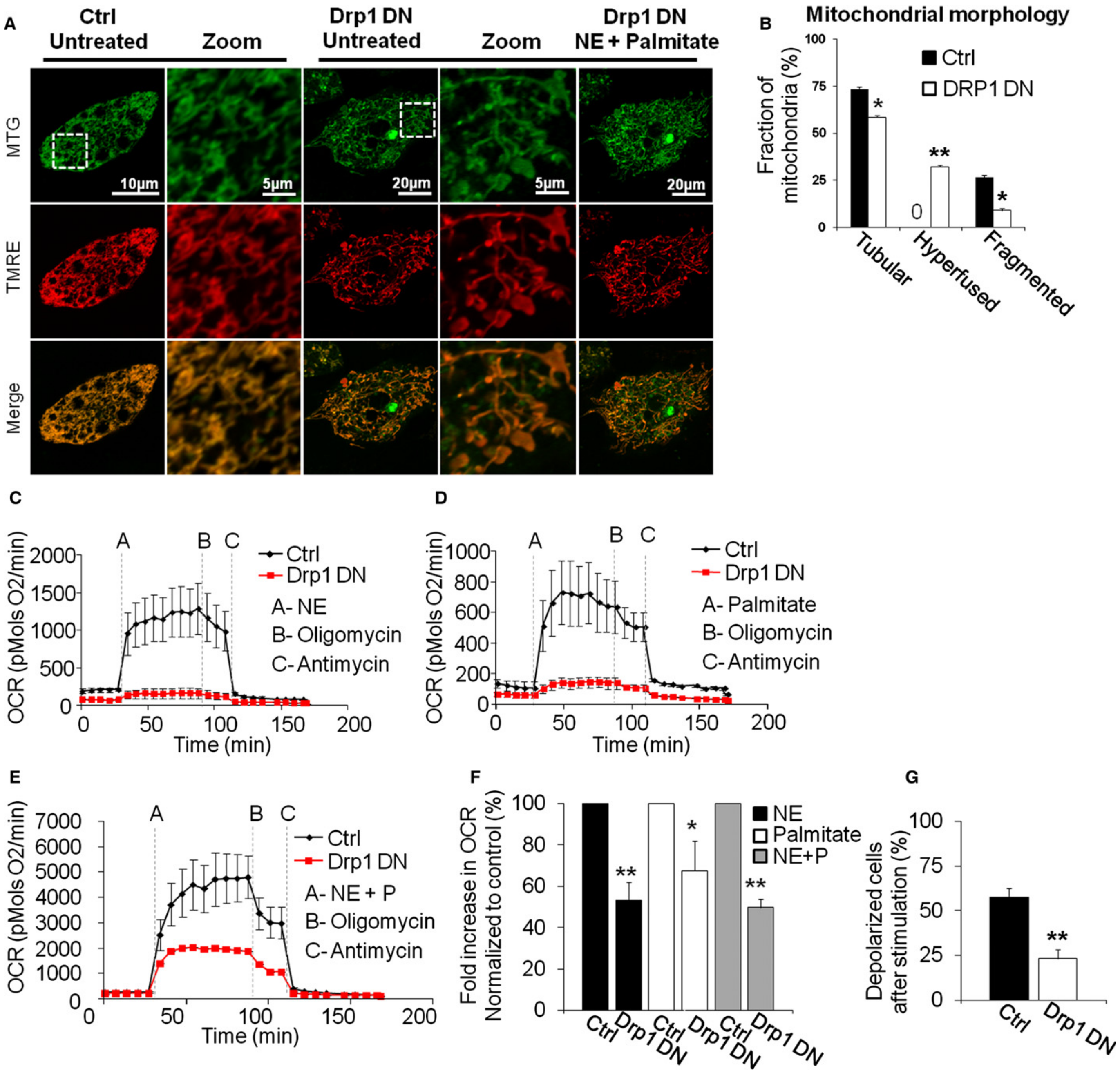

Figure 8. Inhibition of fission dampens stimulated brown adipocyte energy expenditure.

A Inhibition of mitochondrial fragmentation by the expression of Drp1 DN (DRP1 K38A) blocks $\Delta\psi_m$ depolarization. Typical appearance of cell stained with TMRE/MTG expressing Drp1 DN, imaged before and after NE plus palmitate stimulation. Note the hyperfused mitochondria in Drp1 DN cells and the lack of change in mitochondrial morphology or $\Delta\psi_m$ upon stimulation (only Drp1 DN cells are shown). White square indicates area of zoom. Control cells were infected with equivalent amounts of $_{mt}$PAGFP adenovirus. Scale bar 10 and 5 μm for the zoom images of the control cells and 20 and 5 μm for the zoom images of the Drp1 DN cells.

B Quantification of mitochondrial morphology in unstimulated cells expressing Drp1 DN versus control. $^{**}P < 0.0001$. $^{*}P < 0.05$. $n = 20$ (control), $n = 23$ (Drp1 DN).

C Drp1 DN cells show diminished response in oxygen consumption rates (OCR, pmols O_2/min) to NE alone.

D Drp1 DN cells show diminished response in OCR to palmitate alone.

E Drp1 DN cells show diminished response in OCR to NE plus palmitate. Note that prior to stimulation there is no difference in oxygen consumption.

F Quantification of OCR in brown adipocytes expressing Drp1 DN and stimulated with NE and/or palmitate. Fold is calculated as the fold increase in OCR over the pre-stimulated OCR, with OCR values obtained after the addition of antimycin being used as the zero OCR value ($n = 10$ each condition). $^{*}P < 0.05$, $^{**}P < 0.001$.

G Quantification of the fraction of Drp1 DN cells that depolarize in response to NE plus palmitate. $n = 19$ (control), $n = 22$ (Drp1 DN). $^{**}P < 0.0001$.

© 2014 The Authors

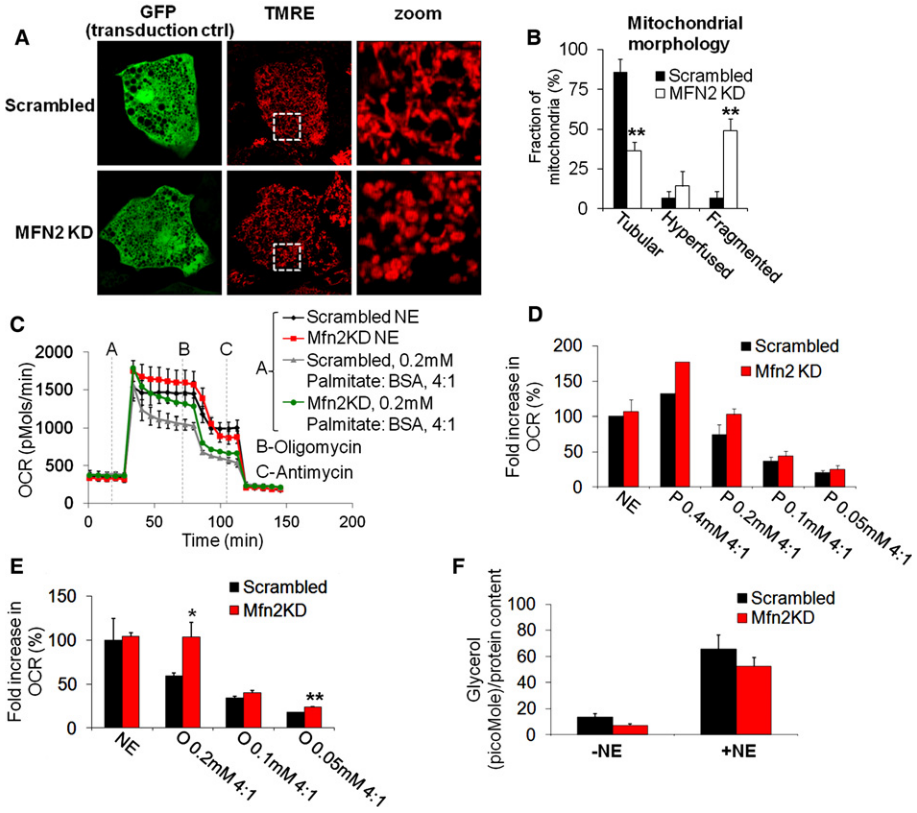

Figure 9. Knock down of Mfn2 enhances brown adipocyte sensitivity to FFA.

A Mfn2 knock down (KD) elicits mitochondrial fragmentation in BA. Typical appearance of cell stained with TMRE and expressing microRNA targeting MFN2 or scrambled as control (both co-expressing GFP). Note the fragmented and swollen mitochondria in Mfn2 KD. White square indicates area of zoom.

B Quantification of mitochondrial morphology in unstimulated MFN2 KD cells versus control. n = 31 (control), 32 (MFN2 KD). $^{**}P < 0.01$.

C Representative oxygen consumption rates (OCR) traces of cells stimulated with NE or palmitate. Mfn2 KD cells show similar response in OCR to NE but increased response to palmitate. Each trace represents 2–3 replicates per group.

D Quantification of the effect of Mfn2 KD on OCR in response to stimulation with NE alone or with different palmitate concentrations as indicated. Mfn2 KD cells show increased sensitivity to palmitate in oxygen consumption. Data are normalized to scrambled (control) NE. n = 3 independent experiments. Fold is calculated as the fold increase in OCR over the pre-stimulated OCR, with OCR values obtained after the addition of antimycin being used as the zero OCR value.

E Quantification of the effect of Mfn2 KD on OCR in response to stimulation with NE alone or with different oleate concentrations as indicated. Mfn2 KD cells show increased sensitivity to oleate in oxygen consumption. Data are normalized to scrambled (control) NE. Fold was calculated as in (D). n = 3 independent experiments. $^{*}P = 0.05$, $^{**}P = 0.0012$.

F Quantification of the glycerol release in the presence and absence of the NE. Note that there is slightly lower glycerol release in the Mfn2 KD both in the basal and stimulated conditions. n = 3 independent experiments.

Source data are available online for this figure.

uncoupling as NE alone. These initial findings per se are relevant in the context of research exploring brown adipose tissue as a therapeutic approach for metabolic diseases. Our data further confirm other studies supporting that unstimulated brown fat will not significantly oxidize exogenous nutrients to generate heat via uncoupled respiration (Bartelt *et al*, 2011), but will accumulate part of these nutrients into lipid droplets (Betz *et al*, 2012). Consistent with this, therapeutic approaches activating mitochondrial processes downstream of NE activation are likely to be more efficient in terms of eliminating nutrients in excess, when compared to approaches that just increase BAT mass. In this study, we have identified that mitochondrial fragmentation is a process downstream of NE that enhances mitochondrial uncoupling in BA. As a consequence of this finding, we characterized the molecular mechanisms leading to this fragmentation in BA.

Mechanisms for NE-mediated mitochondrial fragmentation in brown adipocytes

NE acutely stimulates mitochondrial fission through Drp1 activation

We have demonstrated that NE induces fragmentation through Drp1-mediated fission. This is associated with increased Drp1 punctae formation and Drp1 phosphorylation in the equivalent serine to human Ser600 (ubiquitous isoform). This phosphorylation was previously shown to stimulate Drp1-mediated fission in other cell types (Han *et al*, 2008). In this regard, we have identified that mouse Drp1 phosphorylation in the equivalent serine to human Ser600 was dependent on Protein Kinase A (PKA) activity, as it was prevented by the PKA inhibitor H89. PKA activity is one of the main mediators transducing NE signal inside BA, including lipolysis (Can-

© 2014 The Authors

non & Nedergaard, 2004). We show for the first time that NE stimulates Drp1-mediated fission and its phosphorylation by PKA. Of note, we have confirmed the functional importance of Drp1-mediated fission during BA activation by specifically inhibiting Drp1 function using transient overexpression of Drp1 dominant negative (DN, K38A, inactivating its GTPase domain and fission activity). Drp1 DN overexpression decreased mitochondrial depolarization and respiration rates after NE stimulation, showing that Drp1 activity is required for acute mitochondrial uncoupling and energy expenditure in BA. Importantly, when lipolysis was prevented using Orlistat, mitochondrial depolarization was inhibited, but neither fragmentation nor Drp1 phosphorylation was affected. This result suggests that NE-mediated activation of Drp1 is regulated by hormone-mediated phosphorylation of Drp1 and does not require induction of depolarization.

Inhibition of mitochondrial fusion after NE-stimulated fission in BA is associated with depolarization and Opa1 processing

We observed that mitochondrial fusion event in non-stimulated brown adipocytes are abundant. As mitochondria go through cycles of a fission and fusion event (Twig *et al*, 2008a), one would expect that the arrest of fusion would only result in complete fragmentation after multiple cycles in which fission events were not followed by fusion events. This means that fusion should be inhibited for approximately an hour to result in visible fragmentation. Thus, the kinetics of mitochondrial fusion rates in non-stimulated BA corroborates that increased fission is the first event causing fragmentation after NE stimulation. However, we tested whether these fragmented mitochondria in activated BA maintained normal fusion rates. Dilution rates of the fluorescence intensity signal from mitochondrially-targeted photoactivated GFP (mtPAGFP) are used as an estimation of mitochondrial fusion rates. Fragmented mitochondria both in BA treated with NE or with both NE + palmitate failed to dilute their $_{mt}$PAGFP compared to unstimulated cells. These results demonstrate that BA activation not only increased Drp1-mediated fission, but also decreased mitochondrial fusion rates. We next examined the mechanism by which fusion rates might be decreased in mitochondria from activated brown adipocytes, focusing on the mitochondrial fusion protein Opa1.

Opa1 is a mitochondrial GTPase with multiple that forms, generated both by splicing and proteolytic processing. Some of these forms are integral inner membrane proteins (longer forms) and others are soluble and located in the intermembrane space (Liesa *et al*, 2009). Decrease in the longer forms is associated with mitochondrial fragmentation (Guillery *et al*, 2008), since the short forms cannot mediate mitochondrial fusion per se (Duvezin-Caubet *et al*, 2006; Ishihara *et al*, 2006). In this regard, inhibition of mitochondrial fusion induced by mitochondrial membrane potential depolarization (FCCP treatment) was partly attributed to this Opa1 processing (Duvezin-Caubet *et al*, 2006; Ishihara *et al*, 2006). Loss of mitochondrial membrane potential and ATP deficiency both trigger this Opa1 processing by the Oma1 protease (Ehses *et al*, 2009). Consistent with these studies performed in other cell types, we detected a shift to the shorter Opa1 forms in BA treated with NE, which may also explain the decrease in mitochondrial fusion rates. Interestingly, mice harboring a long-term deficiency in Oma1 (whole body knock-out) show decreased BAT thermogenic function, together with an impairment of Opa1 proteolytic processing in BAT (Quiros *et al*, 2012). Since OMA1 excision also impaired BAT differentiation, the authors could not use this model to study the isolated effect of Opa1 processing on acute activation. One would expect that a post differentiation KD of Oma1 would lead to the generation of BAT with the inability to process Opa1 after NE stimulation. We expect that this inability to process Opa1 and inhibit fusion would decrease the capacity of mitochondrial uncoupling and energy expenditure.

In addition to increased fission and decreased fusion rates, we found that BA mitochondria swelled after NE+FFA, showing a balloon shape with an enlargement of the matrix volume. This swelling was reversible and did not affect later stimulation of mitochondrial function. This data suggested that this swelling represents a physiological or at least controlled response. Consistent with this, we found that this spherical shape was seen in brown adipose tissue of mice exposed to cold, both by electron microscopy and immuno-histochemistry, but not in BAT from mice under thermoneutrality. These data are also consistent with previous electron microscopy studies of BAT. Mitochondria isolated from cold-acclimated rats were reported to be enlarged (Desautels & Himms-Hagen, 1980). In a study in which BAT was sampled after NE injection, mitochondria showed transient swelling (Vallin, 1970). Thus, although typically being associated with pathology, mitochondrial swelling may under certain conditions play a physiological role. Moreover, it was shown that Opa1 knock down leads to swollen mitochondria and cristae before mitochondrial fragmentation (Griparic *et al*, 2004). Thus, the decrease in long Opa1 isoforms may be involved in the transition of mitochondria to the large spherical architecture. As a consequence, decreased mitochondrial fusion rates might be facilitating this mitochondrial swelling. Bioenergetically, swelling may favor uncoupled respiration. Changes to the curvature of the cristae are expected to reduce ATP synthase dimerization and reduce coupled respiration as cristae structure and ATP synthase dimerization are interrelated (Paumard *et al*, 2002).

Mitochondrial fragmentation increases mitochondrial uncoupling capacity of exogenous FFA

While our results show that mitochondrial fragmentation is required for proper BA activation, we wanted to test whether forced mitochondrial fragmentation could enhance mitochondrial uncoupling and thus energy expenditure. A positive result in this test would support that increased mitochondrial fragmentation in BAT could be a potential therapeutic target for metabolic diseases. In other words, we would have found a pathway to potentially increase energy expenditure in BAT and deal with nutrient excess bypassing adrenergic stimulation. In order to force fragmentation, we knocked down Mfn2 in primary BA. We did not find any alteration in the ability of NE to stimulate energy expenditure in Mfn2 KD BA. This result suggests that forced mitochondrial fragmentation by Mfn2 KD does not alter mitochondrial function nor lipolysis stimulation or FFA-mediated activation of UCP1. On the other hand, we found that low and physiological concentrations of FFA (both oleate and palmitate) increased BA energy expenditure to a larger extent in Mfn2 KD BA, when compared to control BA. In this case, control BA treated with FFA did not show fragmentation, whereas Mfn2 KD BA did. Alto-

 © 2014 The Authors

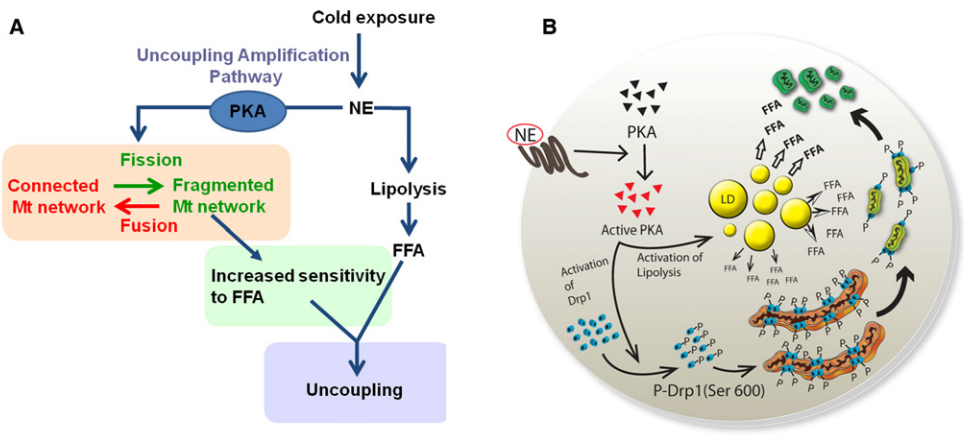

Figure 10. Model of the role of mitochondrial dynamics in the brown adipocyte.
Mitochondrial uncoupling in the brown adipocyte is a result of the coordinated action of two pathways that synergize. NE induces the production of FFA by lipolysis, while the amplification pathway proposed here increases the sensitivity of mitochondria to FFA by changing mitochondrial architecture, which is dependent on PKA. The combination of adrenergic stimuli and FFAs activates both pathways and induces a synergistic increase in thermogenesis/uncoupling.

gether, these results suggest that the enhanced capacity of FFA to stimulate respiration in Mfn2 KD is caused by the permissive action of fragmentation increasing and/or facilitating BA energy expenditure. While the differences in respiration detected in this model of fragmentation are significant and considerable, it is hard to predict whether forced fragmentation by Mfn2 KD in BAT would be sufficient to increase energy expenditure in a whole organism in the context of nutrient excess. However, these differences strongly support the hypothesis that forced fragmentation might be a feasible pathway to increase energy expenditure in BAT. Therefore, a therapeutic approach selectively increasing fragmentation in BAT could potentially increase energy expenditure bypassing the requirement for adrenergic stimulation. This approach would prevent the expected and known complications associated with a potential treatment based on adrenergic stimulation.

Conclusions and Model

We propose a model where NE-induced mitochondrial fragmentation serves as an amplification pathway for uncoupling in brown adipocytes acting downstream of lipolysis and facilitating Ucp1 activation by FFA (Fig 10). Consequently, this may represent a novel role for mitochondrial dynamics in the regulation of energy efficiency. This change in mitochondrial dynamics may be increasing the capacity of FFA to reach Ucp1 or increasing the sensitivity of Ucp1 to FFA. Indeed, the higher ability of oleate to induce mitochondrial uncoupling compared to palmitate might be consistent with a current report showing the mechanism of activation of Ucp1 by FFAs. This study showed that the longer the hydrophobic tail was, the more efficiently the FFA activated Ucp1 (Fedorenko *et al*, 2012). The fact that oleate's hydrophobic tail (C18) is longer than palmitate (C16), together with a hypothetical better ability of oleate to be protonated in the inter-membrane space and thus translocate protons, could hypothetically explain its higher efficiency in depolarizing mitochondria.

Consistent with these findings, our results suggest that induction of mitochondrial fragmentation could be used as a mechanism to enhance the capacity of plasma FFA to induce thermogenesis, even in the absence of adrenergic stimulation. In all, our study shows that mitochondrial dynamics in the brown adipose tissue might be a novel potential therapeutic target to treat metabolic diseases associated with nutrient excess.

Materials and Methods

Experimental animals

BAT was harvested from 3 to 4-week-old wild-type male C57BL6/J mice (Jackson lab, Bar Harbor, ME). Animals were fed standard chow (mouse diet 9F, PMI Nutrition International, Brentwood, MO) and maintained under controlled conditions (19–22°C and a 14:10 h light-dark cycle) until death by CO_2 asphyxiation. All procedures were performed in accordance with the Boston University Institutional Guidelines for Animal Care (IACUC) in compliance with U.S. Public Health Service Regulation.

Cell culture and oxygen consumption measurements

Mouse BA were isolated and cultured as previously described in detail (Cannon & Nedergaard, 2001). In brief, preadipocytes isolated from BAT from three 3-week-old C57BL6/J male mice were resuspended in 6 ml of culture medium after BAT collagenase digestion and washing steps. For oxygen consumption experiments, 100 µl were seeded into each well of a V7 cell culture plate (Seahorse Bioscience, Billerica, MA), followed by cell attachment for 1 h and thereafter addition of 150 µl medium consisting of DMEM supplemented with 10% newborn calf serum, 4 nM insulin, 10 mM HEPES, 4 mM glutamine, 50 IU of penicillin, 50 µg streptomycin,

© 2014 The Authors

25 μg/ml sodium ascorbate and 1 μM rosiglitazone. For microscopy, 200 μl were seeded in the center of 35-mm glass bottom plates (MatTek, Ashland, MD), allowing cell attachment for 45 min and thereafter addition of 1.8 ml of medium. Medium was replaced the day after seeding and every second day until preadipocytes had differentiated into brown adipocytes (7 days of culture). Rosiglitazone (Toronto research chemicals, Ontario, Canada) was used as differentiation agent (Petrovic *et al*, 2010) (Supplementary Fig 7).

Before the oxygen consumption measurements, BA media was replaced to assay media (3 mM glucose, 0.8 mM Mg^{2+}, 1.8 mM Ca^{2+}, 143 mM NaCl, 5.4 mM KCl, 0.91 mM NaH_2PO_4, and Phenol red 15 mg/ml (Seahorse Bioscience), followed by incubation for 60 min at 37°C (no CO_2) before loading into the XF24 extracellular analyzer (Seahorse Bioscience) (Wu *et al*, 2007). During these 60 min, the ports of the cartridge containing the oxygen probes were loaded with the compounds to be injected during the assay (50 μl/port) and the cartridge was calibrated. Cells were incubated with H89 (Sigma), and Orlistat (Sigma) in regular media for 1 h before the experiment at the concentrations of 5 and 100 μM respectively. H89 was diluted in H_2O and Orlistat was diluted in DMSO. Both were present in the assay media during stimulation with NE.

Injection compounds used for imaging and oxygen consumption measurements

NE, cirazoline, and CL-316243 (Sigma, St Louis, MO) were prepared freshly in assay media at stock concentrations of 10 mM and used at final concentrations of 1 μM (which was used in all experiments). Palmitate and oleate were dissolved in DMSO to a final concentration of 0.1, 0.2 and 0.4 M and then dissolved at 56°C in RPMI medium containing 5% fatty-acid-free BSA (Roche, Nutley, NJ) to make a 10× stock (4 mM). To determine the optimal ratio of FFA:BSA, we performed a titration curve for both FFA and for NE (Supplementary Fig 1A and B). We determined that a FFA:BSA ratio of 4:1 gives the strongest respiratory response. FCCP (Sigma) was dissolved in DMSO to 20 mM and used at final concentrations of 1 μM. FCCP was injected with sodium pyruvate (Sigma) at final concentration of 5 mM. Oligomycin and antimycin were also used at the final concentration of 5 μM.

Viruses

Lentiviral particles (for $_{mt}$DsRed and $_{mt}$PAGFP transduction) were generated by transient transfection of 293T cells using FuGENE 6 (Roche, Nutley, NJ) according to manufacturer's instructions and the three-plasmid system according to Tronolab's protocols. This system is comprised of the packaging plasmid pCMV-AdR8.91, the envelope coding plasmid pMD2.G and the transfer vector plasmid pLVCTH. Adenoviral particles for Drp1 DN expression was generated using the AdenoEasy system as described before (Molina *et al*, 2009). $_{mt}$PAGFP adenovirus were used as transduction control (Molina *et al*, 2009). For Mfn2 knock down, as a transduction control, we used Ad-miRCt (encoding for control miRNA) and Ad-miR2 encoding for 5 miRNAs against MFN2. miRNAs were generated with the Block-it Pol II system from Invitrogen using the expression vector pcDNA 6.2 Gw/EmGFP/miR, which allows the cloning of several miRNAs in tandem and contains a GFP expression cassette. miRNAs were cloned by recombination into the pAdeno-CMV-V5 adenoviral vector (Invitrogen) by using the Gateway system (Sebastian *et al*, 2012). Adenoviruses were generated by transfection of the linearized adenoviral expression vectors in a human embryonic kidney cell line (HEK293). The adenoviruses generated were then amplified in HEK293 cells, titrated with the Adeno-X Rapid Titer kit (Clontech), and used directly for cell transduction.

Fluorescent dyes

Nile Red was used at 1 μg/ml for 15 min followed by wash-out before imaging. Mitotracker Green (MTG) was used at 200 nM for 90 min followed by wash-out before imaging. Tetramethylrhodamine-ethyl-ester-perchlorate (TMRE) was loaded at 30 nM and present during imaging. All dyes were from Invitrogen.

Immunohistochemistry

After fixation with 4% paraformaldehyde in PBS for 15 min, cells were permeabilized with 0.2% Triton X-100 (Sigma) for 10 min and thereafter unspecific binding sites were blocked using PBS with 1% BSA (Calbiochem, La Jolla, CA). Ucp1 primary antibody (antimouse, rabbit polyclonal, raised against COOH-terminal decapeptide) was used at a dilution of 1:3,000 and incubated for 30 min. Alexa 594 (Invitrogen) was used as a secondary antibody at 1:500 and incubated for 30 min. Drp1 primary antibody (antimouse, mouse monoclonal, BD Biosciences) was diluted in PBS with 1% BSA at the concentration of 1:100 and incubated for 1 h. Alexa 488 (Invitrogen) was used as a secondary antibody at 1:500 in PBS and incubated for 1 h.

Microscopy

Zeiss LSM 710 confocal microscope was used for the imaging experiments. 488 nm laser was used to excite MTG, activated $_{mt}$PAGFP and Alexa 488, while 543 nm laser was used for detection of $_{mt}$DsRed, Ucp1 immunostaining, Nile Red and TMRE. A 63× objective was used for all imaging.

Photoconversion of $_{mt}$PAGFP to its active (fluorescent) form was achieved by using 2-photon laser (750 nm) to give a 375 nm photon-equivalence at the focal plane. This allowed for selective activation of mitochondrial regions that have submicron thickness and are < 0.5 μm^2 (Twig *et al*, 2006).

Electron microscopy

IBAT from mouse acclimated to 28°C for 2–4 weeks and iBAT from mouse acclimated to 6°C for 1–5 days were harvested. Small tissue fragments fixed in buffered 4% formaldehyde overnight at 4°C were embedded in epoxy resin and selected areas were sectioned for electron microscopy observation. Small fragments of tissue were treated for maceration procedure (Riva *et al*, 1999) and studied in a ZEISS Supra40 HR SEM.

Image analysis

Ten images for each experimental group were acquired by the TEM digital camera at 6200 and 7100 magnifications. For each image, all whole mitochondria were profiled by LUCIA (laboratory Universal

 © 2014 The Authors

Computer Image Analysis) to obtain the mean mitochondria area (in μm^2). The image analysis software was calibrated using cross ruled gratings of known distance (0.463 μm).

Mitochondrial membrane potential measurements

$\Delta\psi_m$ was measured by quantifying the average fluorescence intensity of BA stained with the combination of MTG and TMRE before and after stimulation, as previously described in detail (Wikstrom *et al*, 2007). In addition, BA showing $\Delta\psi_m$ depolarization was determined by visual inspection and defined as by the loss (≥ 30% reduction) of TMRE staining after stimulation.

Image analysis

Metamorph image analysis software (Molecular Devices, Sunnyvale, CA) was used to analyze all images for fluorescence intensities that were in turn further processed in Excel software. Image contrast and intensities were not adjusted in images used for quantifications (MTG/TMRE for $\Delta\psi_m$ and fraction of depolarized cells, Ucp1 content, $_{mt}$PAGFP intensity, and mitochondrial morphology). Quantification of mitochondrial morphology was performed by visual inspection and cells were divided into three groups exhibiting tubular, fragmented or hyperfused mitochondria.

Western blotting

Protein extracts were subjected to SDS–polyacrylamide gel electrophoresis and immunoblotting using the following primary antibodies: Ucp1 (rabbit polyclonal, raised against COOH-terminal decapeptide), Ucp1 (rabbit polyclonal; Abcam, Cambridge, MA), porin and Mfn2 (mouse monoclonal; Abcam), Opa1 and Drp1 (mouse monoclonal; BD Biosciences), Phospho-Drp1(Ser600/637) (rabbit polyclonal; Cell Signaling, Beverly, MA).

Data are given as means ± s.e. Two-tailed, unpaired, or paired Student's *t*-tests were used to compare data sets. $P < 0.05$ was considered significant.

Supplementary information for this article is available online: http://emboj.embopress.org

Acknowledgements

We thank Drs Charlotte Mattsson, Irina Shabalina, Mike Kirber, Jude Deeney, Susan Fried, Daniel Dagan, Alenka Lowy Wheeler, Andrew Ferree, Kyle Trudeau, Fernanda Cerqueira, Linsey Stiles, Tova Meshulam and Charles Berdan for helpful discussions and advice. M.L. is an Evans Center Fellow and was the recipient of a post-doctoral fellowship from Fundacion Ramon Areces. This work was supported by National Institutes of Health grants R01 DK074778, R01 DK56690-11 and by the Swedish Research Council as well as DIABAT FP7, collaborative EU Grant HEALTH-F2-2011-278373 to SC, BC and JN and the Wallenberg Foundation (to BC and JN).

Author contributions

JDW, KM, ML designed and performed experiments, analyzed data and wrote the paper together with OSS. SBS, YS, GL, GT, NP, CZ, AG performed experiments and analyzed data. OSS, BC, JN, BEC and SC mentored the study.

Conflict of interest

The authors declare that they have no conflict of interest.

References

Bachman ES, Dhillon H, Zhang CY, Cinti S, Bianco AC, Kobilka BK, Lowell BB (2002) BetaAR signaling required for diet-induced thermogenesis and obesity resistance. *Science* 297: 843 – 845

Bartelt A, Bruns OT, Reimer R, Hohenberg H, Ittrich H, Peldschus K, Kaul MG, Tromsdorf UI, Weller H, Waurisch C, Eychmuller A, Gordts PL, Rinninger F, Bruegelmann K, Freund B, Nielsen P, Merkel M, Heeren J (2011) Brown adipose tissue activity controls triglyceride clearance. *Nat Med* 17: 200 – 205

Betz MJ, Bielohuby M, Mauracher B, Abplanalp W, Muller HH, Pieper K, Ramisch J, Tschop MH, Beuschlein F, Bidlingmaier M, Slawik M (2012) Isoenergetic feeding of low carbohydrate-high fat diets does not increase brown adipose tissue thermogenic capacity in rats. *PLoS ONE* 7: e38997

Cannon B, Nedergaard J (2001) Cultures of adipose precursor cells from brown adipose tissue and of clonal brown-adipocyte-like cell lines. *Methods Mol Biol* 155: 213 – 224

Cannon B, Nedergaard J (2004) Brown adipose tissue: function and physiological significance. *Physiol Rev* 84: 277 – 359

Cannon B, Sundin U, Romert L (1977) Palmitoyl coenzyme A: a possible physiological regulator of nucleotide binding to brown adipose tissue mitochondria. *FEBS Lett* 74: 43 – 46

Cereghetti GM, Stangherlin A, Martins de Brito O, Chang CR, Blackstone C, Bernardi P, Scorrano L (2008) Dephosphorylation by calcineurin regulates translocation of Drp1 to mitochondria. *Proc Natl Acad Sci USA* 105: 15803 – 15808

Chang CR, Blackstone C (2007) Drp1 phosphorylation and mitochondrial regulation. *EMBO Rep* 8: 1088 – 1089; author reply 1089–1090

Cypess AM, Lehman S, Williams G, Tal I, Rodman D, Goldfine AB, Kuo FC, Palmer EL, Tseng YH, Doria A, Kolodny GM, Kahn CR (2009) Identification and importance of brown adipose tissue in adult humans. *N Engl J Med* 360: 1509 – 1517

Desautels M, Himms-Hagen J (1980) Parallel regression of cold-induced changes in ultrastructure, composition, and properties of brown adipose tissue mitochondria during recovery of rats from acclimation to cold. *Can J Biochem* 58: 1057 – 1068

Duvezin-Caubet S, Jagasia R, Wagener J, Hofmann S, Trifunovic A, Hansson A, Chomyn A, Bauer MF, Attardi G, Larsson NG, Neupert W, Reichert AS (2006) Proteolytic processing of OPA1 links mitochondrial dysfunction to alterations in mitochondrial morphology. *J Biol Chem* 281: 37972 – 37979.

Ehses S, Raschke I, Mancuso G, Bernacchia A, Geimer S, Tondera D, Martinou JC, Westermann B, Rugarli EI, Langer T (2009) Regulation of OPA1 processing and mitochondrial fusion by m-AAA protease isoenzymes and OMA1. *J Cell Biol* 187: 1023 – 1036

Fedorenko A, Lishko PV, Kirichok Y (2012) Mechanism of fatty-acid-dependent UCP1 uncoupling in brown fat mitochondria. *Cell* 151: 400 – 413

Freedman NJ, Lefkowitz RJ (1996) Desensitization of G protein-coupled receptors. *Recent Prog Horm Res* 51: 319 – 351

Gomes LC, Di Benedetto G, Scorrano L (2011) During autophagy mitochondria elongate, are spared from degradation and sustain cell viability. *Nat Cell Biol* 13: 589 – 598

Griparic L, van der Wel NN, Orozco IJ, Peters PJ, van der Bliek AM (2004) Loss of the intermembrane space protein Mgm1/OPA1 induces swelling and

© 2014 The Authors

localized constrictions along the lengths of mitochondria. *J Biol Chem* 279: 18792–18798
Guillery O, Malka F, Landes T, Guillou E, Blackstone C, Lombes A, Belenguer P, Arnoult D, Rojo M (2008) Metalloprotease-mediated OPA1 processing is modulated by the mitochondrial membrane potential. *Biol Cell* 100: 315–325
Hall RA (2004) Beta-adrenergic receptors and their interacting proteins. *Semin Cell Dev Biol* 15: 281–288
Han XJ, Lu YF, Li SA, Kaitsuka T, Sato Y, Tomizawa K, Nairn AC, Takei K, Matsui H, Matsushita M (2008) CaM kinase I alpha-induced phosphorylation of Drp1 regulates mitochondrial morphology. *J Cell Biol* 182: 573–585
Ishihara N, Fujita Y, Oka T, Mihara K (2006) Regulation of mitochondrial morphology through proteolytic cleavage of OPA1. *EMBO J* 25: 2966–2977
Karbowski M, Arnoult D, Chen H, Chan DC, Smith CL, Youle RJ (2004) Quantitation of mitochondrial dynamics by photolabeling of individual organelles shows that mitochondrial fusion is blocked during the Bax activation phase of apoptosis. *J Cell Biol* 164: 493–499
Kim MJ, Kang KH, Kim CH, Choi SY (2008) Real-time imaging of mitochondria in transgenic zebrafish expressing mitochondrially targeted GFP. *Biotechniques* 45: 331–334
Liesa M, Palacin M, Zorzano A (2009) Mitochondrial dynamics in mammalian health and disease. *Physiol Rev* 89: 799–845
Liesa M, Shirihai OS (2013) Mitochondrial dynamics in the regulation of nutrient utilization and energy expenditure. *Cell Metab* 17: 491–506
Liu X, Hajnoczky G (2011) Altered fusion dynamics underlie unique morphological changes in mitochondria during hypoxia-reoxygenation stress. *Cell Death Differ* 18: 1561–1572
van Marken Lichtenbelt WD, Vanhommerig JW, Smulders NM, Drossaerts JM, Kemerink GJ, Bouvy ND, Schrauwen P, Teule GJ (2009) Cold-activated brown adipose tissue in healthy men. *N Engl J Med* 360: 1500–1508
Molina AJ, Wikstrom JD, Stiles L, Las G, Mohamed H, Elorza A, Walzer G, Twig G, Katz S, Corkey BE, Shirihai OS (2009) Mitochondrial networking protects beta-cells from nutrient-induced apoptosis. *Diabetes* 58: 2303–2315.
Mouli PK, Twig G, Shirihai OS (2009) Frequency and selectivity of mitochondrial fusion are key to its quality maintenance function. *Biophys J* 96: 3509–3518
Nedergaard J, Bengtsson T, Cannon B (2007) Unexpected evidence for active brown adipose tissue in adult humans. *Am J Physiol Endocrinol Metab* 293: E444–E452
Parone PA, Da Cruz S, Tondera D, Mattenberger Y, James DI, Maechler P, Barja F, Martinou JC (2008) Preventing mitochondrial fission impairs mitochondrial function and leads to loss of mitochondrial DNA. *PLoS ONE* 3: e3257
Paumard P, Vaillier J, Coulary B, Schaeffer J, Soubannier V, Mueller DM, Brethes D, di Rago JP, Velours J (2002) The ATP synthase is involved in generating mitochondrial cristae morphology. *EMBO J* 21: 221–230
Petrovic N, Walden TB, Shabalina IG, Timmons JA, Cannon B, Nedergaard J (2010) Chronic peroxisome proliferator-activated receptor gamma (PPARgamma) activation of epididymally derived white adipocyte cultures reveals a population of thermogenically competent, UCP1-containing adipocytes molecularly distinct from classic brown adipocytes. *J Biol Chem* 285: 7153–7164
Presley AD, Fuller KM, Arriaga EA (2003) MitoTracker Green labeling of mitochondrial proteins and their subsequent analysis by capillary electrophoresis with laser-induced fluorescence detection. *J Chromatogr B Analyt Technol Biomed Life Sci* 793: 141–150
Quiros PM, Ramsay AJ, Sala D, Fernandez-Vizarra E, Rodriguez F, Peinado JR, Fernandez-Garcia MS, Vega JA, Enriquez JA, Zorzano A, Lopez-Otin C (2012) Loss of mitochondrial protease OMA1 alters processing of the GTPase OPA1 and causes obesity and defective thermogenesis in mice. *EMBO J* 31: 2117–2133
Riva A, Loffredo F, Puxeddu R, Testa Riva F (1999) A scanning and transmission electron microscope study of the human minor salivary glands. *Arch Oral Biol* 44(Suppl. 1): S27–S31
Sebastian D, Hernandez-Alvarez MI, Segales J, Sorianello E, Munoz JP, Sala D, Waget A, Liesa M, Paz JC, Gopalacharyulu P, Oresic M, Pich S, Burcelin R, Palacin M, Zorzano A (2012) Mitofusin 2 (Mfn2) links mitochondrial and endoplasmic reticulum function with insulin signaling and is essential for normal glucose homeostasis. *Proc Natl Acad Sci USA* 109: 5523–5528
Twig G, Elorza A, Molina AJ, Mohamed H, Wikstrom JD, Walzer G, Stiles L, Haigh SE, Katz S, Las G, Alroy J, Wu M, Py BF, Yuan J, Deeney JT, Corkey BE, Shirihai OS (2008a) Fission and selective fusion govern mitochondrial segregation and elimination by autophagy. *EMBO J* 27: 433–446
Twig G, Hyde B, Shirihai OS (2008b) Mitochondrial fusion, fission and autophagy as a quality control axis: the bioenergetic view. *Biochim Biophys Acta* 1777: 1092–1097
Twig G, Graf SA, Wikstrom JD, Mohamed H, Haigh SE, Elorza A, Deutsch M, Zurgil N, Reynolds N, Shirihai OS (2006) Tagging and tracking individual networks within a complex mitochondrial web with photoactivatable GFP. *Am J Physiol Cell Physiol* 291: C176–C184
Twig G, Liu X, Liesa M, Wikstrom JD, Molina AJ, Las G, Yaniv G, Hajnoczky G, Shirihai OS (2010) Biophysical properties of mitochondrial fusion events in pancreatic beta-cells and cardiac cells unravel potential control mechanisms of its selectivity. *Am J Physiol Cell Physiol* 299: C477–C487
Twig G, Shirihai OS (2011) The interplay between mitochondrial dynamics and mitophagy. *Antioxid Redox Signal* 14: 1939–1951
Vallin I (1970) Norepinephrine response in brown adipose tissue from newborn rats. *Acta Zoologica* 51: 129–139
Virtanen KA, Lidell ME, Orava J, Heglind M, Westergren R, Niemi T, Taittonen M, Laine J, Savisto NJ, Enerback S, Nuutila P (2009) Functional brown adipose tissue in healthy adults. *N Engl J Med* 360: 1518–1525
Wikstrom JD, Katzman SM, Mohamed H, Twig G, Graf SA, Heart E, Molina AJ, Corkey BE, de Vargas LM, Danial NN, Collins S, Shirihai OS (2007) Beta-cell mitochondria exhibit membrane potential heterogeneity that can be altered by stimulatory or toxic fuel levels. *Diabetes* 56: 2569–2578
Wu J, Boström P, Sparks LM, Ye L, Choi JH, Giang AH, Khandekar M, Virtanen KA, Nuutila P, Schaart G, Huang K, Tu H, van Marken Lichtenbelt WD, Hoeks J, Enerbäck S, Schrauwen P, Spiegelman BM (2012) Beige adipocytes are a distinct type of thermogenic fat cell in mouse and human. *Cell* 150: 366–376
Wu M, Neilson A, Swift AL, Moran R, Tamagnine J, Parslow D, Armistead S, Lemire K, Orrell J, Teich J, Chomicz S, Ferrick DA (2007) Multiparameter metabolic analysis reveals a close link between attenuated mitochondrial bioenergetic function and enhanced glycolysis dependency in human tumor cells. *Am J Physiol Cell Physiol* 292: C125–C136
Zhang L, Seitz LC, Abramczyk AM, Chan C (2010) Synergistic effect of cAMP and palmitate in promoting altered mitochondrial function and cell death in HepG2 cells. *Exp Cell Res* 316: 716–727
Zingaretti MC, Crosta F, Vitali A, Guerrieri M, Frontini A, Cannon B, Nedergaard J, Cinti S (2009) The presence of UCP1 demonstrates that metabolically active adipose tissue in the neck of adult humans truly represents brown adipose tissue. *Faseb J* 23: 3113–3120

 © 2014 The Authors
