# Supplementary material for: Mitochondrial calcium regulates lipid metabolism by modulating tethering of mitochondria to lipid droplets
Source: EMBO J. 2026 Jul 3;45(14):4820–48. doi: 10.1038/s44318-026-00827-8 (PMC13373242; doi:10.1038/s44318-026-00827-8)
Supplement: Supplementary file 11 — Figure EV3 Source Data [file 44318_2026_827_MOESM11_ESM.zip › Figure EV3/Figure EV3C/from Jakob paper 2014.pptx]

## Slide 1
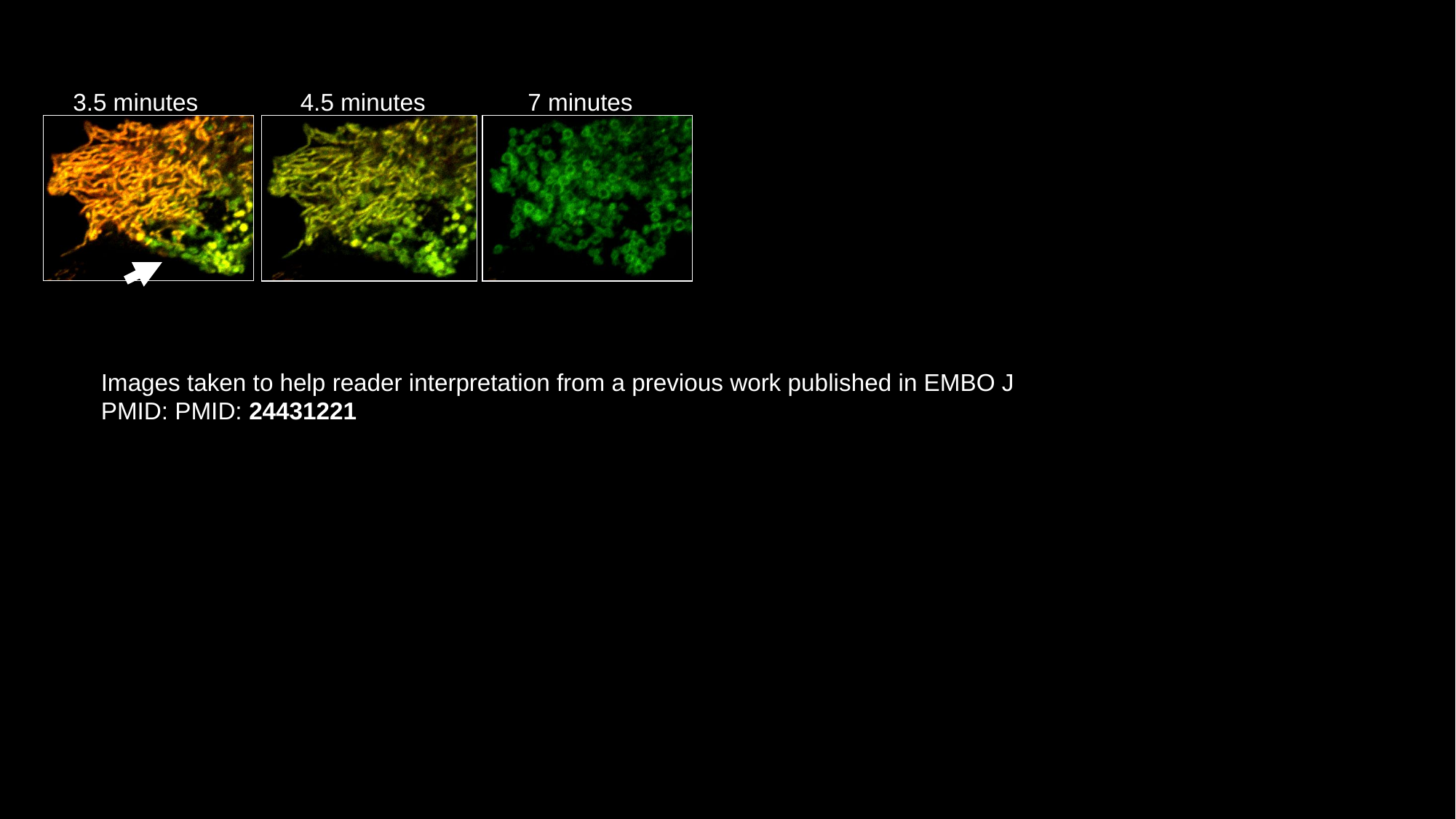

3.5 minutes
4.5 minutes
7 minutes
Images taken to help reader interpretation from a previous work published in EMBO J
PMID: PMID: 24431221
