# Supplementary material for: Mitochondrial calcium regulates lipid metabolism by modulating tethering of mitochondria to lipid droplets
Source: EMBO J. 2026 Jul 3;45(14):4820–48. doi: 10.1038/s44318-026-00827-8 (PMC13373242; doi:10.1038/s44318-026-00827-8)
Supplement: Supplementary file 13 — Expanded View Figures [file 44318_2026_827_MOESM13_ESM.pdf]

Expanded View Figures

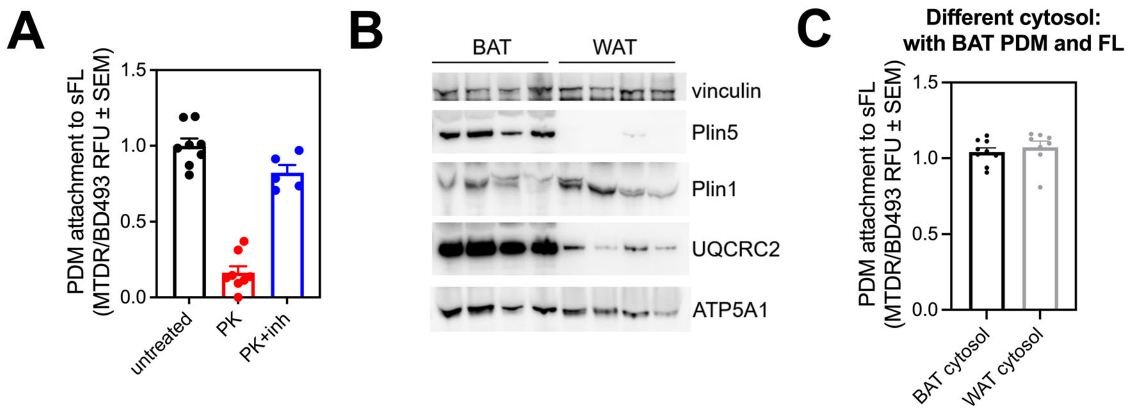

Figure EV1. Proteins coating the LD are important in tethering mitochondrial interaction with LD.

(**A**) Quantification of BAT PDM attached to LD in the presence of proteinase K and proteinase K inhibitors in MAS buffer ($n \geq 5$). (**B**) Western blot in WAT and BAT lysates using the indicated antibodies. (**C**) Comparative analysis of attachment efficacy of mitochondria, LD and cytosol from BAT vs WAT. Reconstituted binding assay using FL and PDM from BAT and either WAT or BAT cytosol ($n \geq 9$). (**A**, **C**) Each point represents a biological replica sample. For each biological replicate, technical replicates were averaged. Data represent average ± SEM. Source data are available online for this figure.

 © The Author(s)

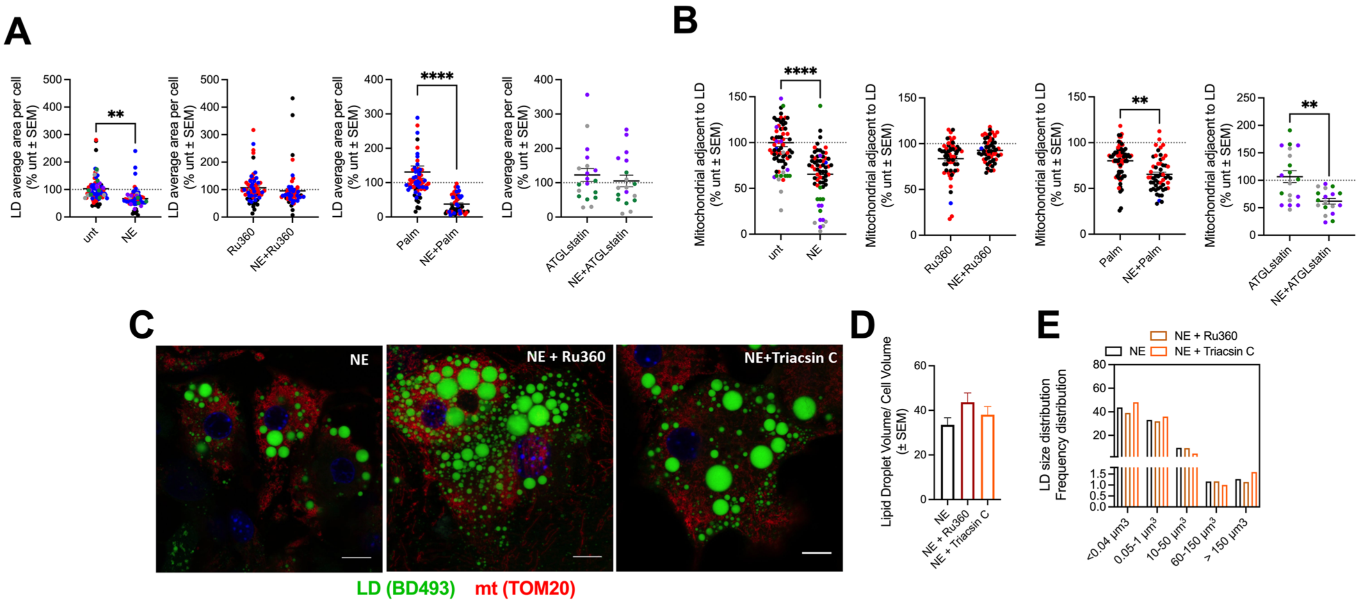

Figure EV2. Effect of modulating re-esterification in lipid droplet size and distribution in pBA.

(**A**, **B**) Integrated LD area per cell (**A**) and PDM (**B**) were quantified as mitochondrial pixels located within 0.3 µm of a lipid droplet in pBA under the indicated conditions ($n \geq 18$). (**C**) Mitochondria (red, TOM20) and LD (green, BD493) 3D imaging in fixed BA under the indicated treatments. Scale bar = 10 µm. (**D**) Quantification of the volume of the cell occupied by LD versus the total volume of the cell in pBA subjected to the indicated treatments ($n \geq 25$). (**E**) Analysis of LD size distribution in pBA subjected to the indicated treatments. Data represent average ± SEM. **$P < 0.01$; ****$P < 0.0001$. (**A**, **B**) Mixed-effects analysis and Welch's test. Source data are available online for this figure.

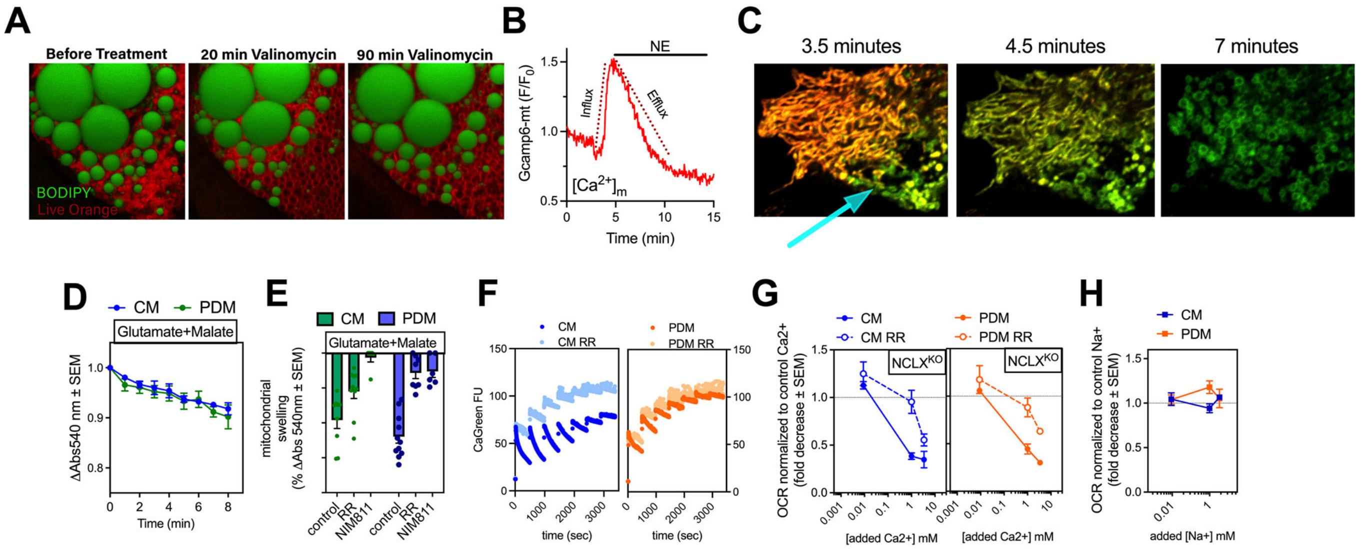

Figure EV3. NE induces a transient surge of calcium in mitochondria that is associated with a change in mitochondrial architecture.

(**A**) Super-resolution confocal images pBA in the presence of Valinomycin for the indicated times. Valinomycin induces swelling and PDM detachment. Mitochondria were stained with Live Orange and LDs with BODIPY 493/503 (BD493). (**B**) Representative trace of mt-Ca^{2+} influx and efflux in primary adipocytes using mt-GCaMP6. (**C**) Effect of NE on mitochondrial architecture and $\Delta\psi_m$ in primary brown adipocytes at different time points. Adipocytes were stained with TMRE (red) and Mitotracker Green (green). Depolarized mitochondria lose red TMRE staining and become green while mitochondria that maintain $\Delta\psi_m$ show yellow-orange mitochondrial staining. Note that NE-stimulation results in a mitochondrial shape change from elongated to fragmented and swollen mitochondria. From Wikstrom et al, 2014. (**D, E**) mt-Ca^{2+} induced swelling assay in CM and PDM isolated from WT mice using Glutamate+malate as substrates ($n \geq 6$), and in the presence or absence of NIM811, a pharmacological inhibitor of the mitochondrial permeability pore (mt-PTP) modulator, Cyclophylin D (CypD). (**F**) Representative traces of buffer Ca^{2+} following the sequential addition of 50 μM bolus of $CaCl_2$ in respiring isolated CM and PDM. RR is used as a negative control of Ca^{2+} entry in mitochondria. (**G**) PC-dependent respiration in BAT CM (blue) and PDM (orange) from $NCLX^{KO}$ mice under the indicated Ca^{2+} concentration and in the absence or presence of RR ($n = 3$). Note that deletion of NCLX eliminates the differences between PDM and CM shown in Fig. 3I,J, resulting in CM taking the same Ca^{2+} sensitivity phenotype as PDM. (**H**) Testing whether altered sodium concentrations contribute to the observed Ca^{2+} sensitivity due to NCLX activity. State 3 PC-dependent respiration was measured in CM (blue) and PDM (orange) from WT mice under the varying sodium concentrations ($n = 6$). Note that changes in sodium concentrations did not impair respiration. Each point represents a biological replica sample. For each biological replicate, technical replicates were averaged. Data represent average ± SEM. Source data are available online for this figure.

 © The Author(s)

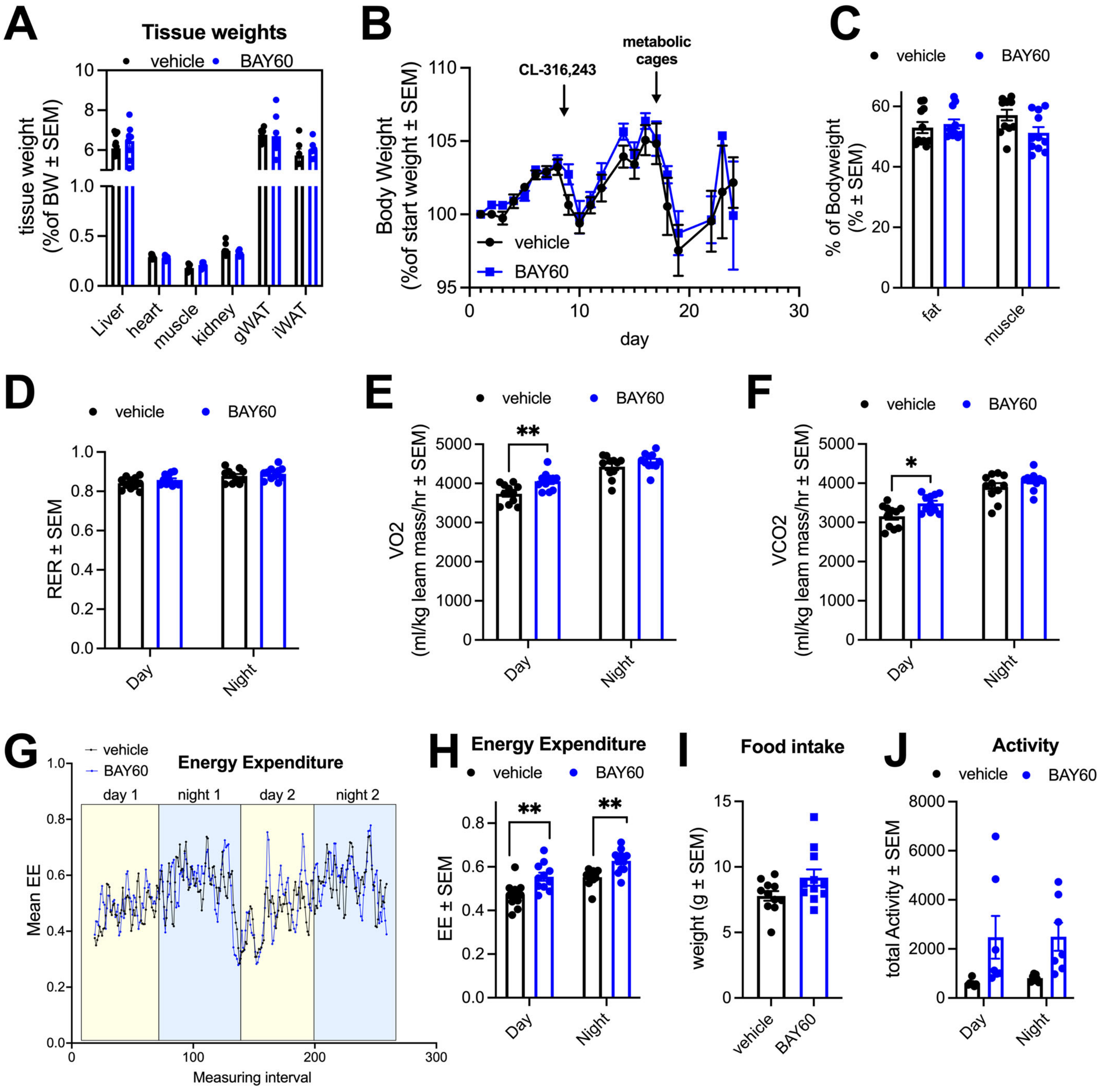

Figure EV4. Mitochondrial function upon PDE2 inhibition in *ob/ob* mice.

(**A**) Weight measurements of the indicated tissues from *ob/ob* mice treatment with vehicle or BAY60. (**B**) Body weight measurements of *ob/ob* mice treated with vehicle or BAY60. Body weights were normalized to weight at day 0 for each mouse. (**C**) Quantification of lean (muscle) and fat mass measured by NMR after 2 weeks of treatment with vehicle or BAY60. (**D**) Quantification of the RER measurements at both light and dark cycles. Values are calculated as the ratio of VCO_2 to VO_2 produced and consumed by the mice, respectively. (**E**) Quantification of VO_2 normalized to lean mass in *ob/ob* mice treated with vehicle or BAY60. (**F**) Quantification of VCO_2 normalized to lean mass in *ob/ob* mice treated with vehicle or BAY60. (**G**, **H**) Representative profile of Energy expenditure measurements during the day and night period in *ob/ob* mice treated with vehicle or BAY60 (**G**) and quantification (**H**). (**I**) Food intake measurements in *ob/ob* mice treated with vehicle or BAY60. (**J**) Total activity in *ob/ob* mice treated with vehicle or BAY60 measured in the metabolic cages. (**A–J**) ($n \geq 6$). Measurements were after 2 weeks of treatment with vehicle or BAY60. Each point represents a biological replica sample. For each biological replicate, technical replicates were averaged. Data represent average ± SEM. *$P < 0.05$; **$P < 0.01$. (**E**, **F**) Two-way ANOVA and (**H**) multiple unpaired *t* test. Source data are available online for this figure.

© The Author(s)
