# Supplementary material for: Mitochondrial calcium regulates lipid metabolism by modulating tethering of mitochondria to lipid droplets
Source: EMBO J. 2026 Jul 3;45(14):4820–48. doi: 10.1038/s44318-026-00827-8 (PMC13373242; doi:10.1038/s44318-026-00827-8)
Supplement: Supplementary file 2 — Peer Review File [file 44318_2026_827_MOESM2_ESM.pdf]

THE EMBO JOURNAL

Mitochondrial Calcium Regulates Lipid Metabolism By Modulating Tethering of Mitochondria to Lipid Droplets

Rebeca Acin-Perez, Essam Assali, Michaela Veliova, Jennifer Ngo, Alexandra Brownstein, Francisco Villalobos, Anton Petcherski, Pablo Hernansanz, Doyeon Kim-Vasquez, Shili Xu, Mikayla Tamboline, Raquel Silva, Alexander Upcher, Cynthia Shu, Daniel Ferriss, Marc Liesa, Jose Antonio Enriquez, Israel Sekler, and Orian Shirihai

Corresponding authors: Orian Shirihai (oshirihai@mednet.ucla.edu) , Israel Sekler (sekler@bgu.ac.il)

Review Timeline:

Submission Date:	17th Aug 25
Editorial Decision:	29th Sep 25
Revision Received:	24th Feb 26
Editorial Decision:	7th Apr 26
Revision Received:	19th May 26
Accepted:	21st May 26

Editor: Daniel Klimmeck

Transaction Report:

(Note: With the exception of the correction of typographical or spelling errors that could be a source of ambiguity, letters and reports are not edited. Depending on transfer agreements, referee reports obtained elsewhere may or may not be included in this compilation. Referee reports are anonymous unless the Referee chooses to sign their reports.)

Dear Orian,

Thank you again for the submission of your manuscript (EMBOJ-2025-122194) to The EMBO Journal, as well as for your patience with our feedback at this time. As mentioned earlier, your study was assessed by three reviewers with expertise in mitochondrial Ca2+ signaling, lipid biology and systemic energy metabolism, whose comments are enclosed below.

As you will see from the experts' reports, the referees acknowledge the analysis and potential interest and value of your findings. However, they also express important issues that will need to be conclusively addressed before they can support publication at the EMBO Journal. In more detail, referee #2 points to major concerns regarding the robustness of the reconstitution assay developed and validity of the results derived thereof (ref#2, pt.1). This expert also raises issues with the completeness of the mechanistic connections drawn (ref#2, pt.6) and support for in vivo relevance of your findings (ref#2, pt.3). Reviewer #1 requests additional complementary experimentation to consolidate your results (ref#1, pt.2). Further, the reviewers raise a number of issues related to the presentation of the findings, clarity of the data presentation and plausibility of the claims from the data shown, that would need to be conclusively addressed to achieve the level of robustness and clarity needed for The EMBO Journal.

Given the overall interest stated and broader angle of your findings, we are able to invite you to revise your manuscript experimentally to address the referees' comments. However, I need to stress that we do require strong support from the referees on a revised version of the study in order to move on to publication of the work.

I would appreciate if you could contact me during the next weeks for exchange e.g. a video call to discuss your perspective on the comments and potential plan for revisions.

Please feel free to contact me if you have any questions or need further input on the referee comments.

We generally allow three months as standard revision time. As a matter of policy, competing manuscripts published during this period will not negatively impact on our assessment of the conceptual advance presented by your study. However, we request that you contact the editor as soon as possible upon publication of any related work, to discuss how to proceed. Should you foresee a problem in meeting this three-month deadline, please let us know in advance and we may be able to grant an extension.

When submitting your revised manuscript, please carefully review the instructions below.

Please feel free to approach me any time should you have additional questions related to this.

Thank you for the opportunity to consider your work for publication.

I look forward to your revision.

Best regards,

Daniel

Daniel Klimmeck, PhD
Senior Editor
The EMBO Journal

Instruction for the preparation of your revised manuscript:

1) a .docx formatted version of the manuscript text (including legends for main figures, EV figures and tables). Please make sure that the changes are highlighted to be clearly visible.

2) individual production quality figure files as .eps, .tif, .jpg (one file per figure).

3) a .docx formatted letter INCLUDING the reviewers' reports and your detailed point-by-point response to their comments. As part of the EMBO Press transparent editorial process, the point-by-point response is part of the Review Process File (RPF), which will be published alongside your paper.

4) a complete author checklist, which you can download from our author guidelines (https://wol-prod-cdn.literatumonline.com/pb-assets/embo-site/Author Checklist%20-%20EMBO%20J-1561436015657.xlsx). Please insert information in the checklist that is also reflected in the manuscript. The completed author checklist will also be part of the RPF.

5) Please note that all corresponding authors are required to supply an ORCID ID for their name upon submission of a revised manuscript.

6) It is mandatory to include a 'Data Availability' section after the Materials and Methods. Before submitting your revision, primary datasets produced in this study need to be deposited in an appropriate public database, and the accession numbers and database listed under 'Data Availability'. Please remember to provide a reviewer password if the datasets are not yet public (see https://www.embopress.org/page/journal/14602075/authorguide#datadeposition).
In case you have no data that requires deposition in a public database, please state so in this section. Note that the Data Availability Section is restricted to new primary data that are part of this study.
*** Note - All links should resolve to a page where the data can be accessed. ***

7) Our journal encourages inclusion of *data citations in the reference list* to directly cite datasets that were re-used and obtained from public databases. Data citations in the article text are distinct from normal bibliographical citations and should directly link to the database records from which the data can be accessed. In the main text, data citations are formatted as follows: "Data ref: Smith et al, 2001" or "Data ref: NCBI Sequence Read Archive PRJNA342805, 2017". In the Reference list, data citations must be labeled with "[DATASET]". A data reference must provide the database name, accession number/identifiers and a resolvable link to the landing page from which the data can be accessed at the end of the reference. Further instructions are available at .

8) At EMBO Press we ask authors to provide source data for the main manuscript figures. You will receive a separate email with instructions for providing source data with your revised manuscript, including how to upload and organize the files.

Numerical data can be provided as individual .xls or .csv files (including a tab describing the data). For 'blots' or microscopy, uncropped images should be submitted (using a zip archive or a single pdf per main figure if multiple images need to be supplied for one panel).

Additional information on source data and instruction on how to label the files are available

9) We replaced Supplementary Information with Expanded View (EV) Figures and Tables that are collapsible/expandable online (see examples in https://www.embopress.org/doi/10.15252/embj.201695874). A maximum of 5 EV Figures can be typeset. EV Figures should be cited as 'Figure EV1, Figure EV2" etc. in the text and their respective legends should be included in the main text after the legends of regular figures.

- For the figures that you do NOT wish to display as Expanded View figures, they should be bundled together with their legends in a single PDF file called *Appendix*, which should start with a short Table of Content. Appendix figures should be referred to in the main text as: "Appendix Figure S1, Appendix Figure S2" etc. See detailed instructions regarding expanded view here: .

- Additional Tables/Datasets should be labelled and referred to as Table EV1, Dataset EV1, etc. Legends have to be provided in a separate tab in case of .xls files. Alternatively, the legend can be supplied as a separate text file (README) and zipped together with the Table/Dataset file.

10) When assembling figures, please refer to our figure preparation guideline in order to ensure proper formatting and readability in print as well as on screen:
http://bit.ly/EMBOPressFigurePreparationGuideline

Please remember: Digital image enhancement is acceptable practice, as long as it accurately represents the original data and conforms to community standards. If a figure has been subjected to significant electronic manipulation, this must be noted in the figure legend or in the 'Materials and Methods' section. The editors reserve the right to request original versions of figures and the original images that were used to assemble the figure.

11) For data quantification: please specify the name of the statistical test used to generate error bars and P values, the number (n) of independent experiments (specify technical or biological replicates) underlying each data point and the test used to calculate p-values in each figure legend. The figure legends should contain a basic description of n, P and the test applied. Graphs must include a description of the bars and the error bars (s.d., s.e.m.).

Please remember: Digital image enhancement is acceptable practice, as long as it accurately represents the original data and conforms to community standards. If a figure has been subjected to significant electronic manipulation, this must be noted in the figure legend or in the 'Materials and Methods' section. The editors reserve the right to request original versions of figures and the original images that were used to assemble the figure.

Further information is available in our Guide For Authors: https://www.embopress.org/page/journal/14602075/authorguide

We realize that it is difficult to revise to a specific deadline. In the interest of protecting the conceptual advance provided by the work, we recommend a revision within 3 months (28th Dec 2025). Please discuss the revision progress ahead of this time with the editor if you require more time to complete the revisions.

Referee #1:

The manuscript entitled, "Mitochondrial Calcium Regulates Lipid Metabolism by Modulating Tethering of Mitochondria to Lipid Droplets" is an exciting study linking control of mitochondrial calcium efflux to their association with the lipid droplet and associated utilization of pyruvate rather than lipids for metabolic activity. Hence, the authors show that transient elevation of mitochondrial calcium activates the mitochondrial permeability transition in PDM (CM were resistant), however, the elevated NCLX activity observed in PDM facilitates recovery from the MPT and resumption of OxPhos. They further identify PDE activity within the mitochondria as a previously unappreciated regulator of NCLX activity. Finally, they show that treating ob/ob mice with the PDE inhibitor BAY60 leads to fat browning, dramatic shifts in substrate utilization towards sugars and increased metabolic utilization. While I found the study highly rational and informative; addressing the issues raised below will significantly increase readability and I urge you to consider these points carefully.

Major comments:

1. Figure 3B is an important panel showing disparate calcium efflux rates between CM and PDM. As shown, there appears to be one black line showing average cytosolic calcium content in CM and a lot of "red" representing PDM calcium? What this is really isn't very clear. In addition, if CM exhibits 2 different rates of efflux at different times, both should be plotted. And it should be possible to see where the rates were taken from the graph of cytosolic calcium concentration. As shown, this graph is very confusing and I struggled to appreciate what it really shows.

2. There are a number of very interesting observations made about the MPT. I am somewhat surprised that there are no measurements of MMP. I would be particularly interested in seeing the MMP recovery that would be expected to accompany a transient MPT event.

3. The sheer number of abbreviations was problematic. I do understand that they were defined, but many sentences were very challenging to follow. If there are space limitations, I must ask the journal to consider readability and permit you more words.

Minor Comments:
1. In reference to figure 1J, there is a statement that Calcium caused a 20% reduction in PDM attachment. Looking at the graph, this statement does not seem to be accurate.

2. Lines 420 to 423, CM is used to describe data from both CM and PDM.

Referee #2:

The manuscript by Acin-Perez, Assali and coworkers study the mechanisms of mitochondria-lipid droplet (LD) interactions in murine brown adipocyte derived material, building on earlier investigations. Previous reports have identified two distinct populations of mitochondria in brown adipose tissue, peridroplet mitochondria (PDM) that are attached to LDs and primarily oxidize pyruvate and cytosolic mitochondria (CM) that are located away from LDs and primarily oxidize fatty acids. It has been shown that adrenergic stimulation results in the detachment of PDM from LDs and leads to mitochondrial fragmentation, balling,

uncoupling and lipid utilization. Adrenergic stimulation of brown adipose tissue also results in an increase in cAMP, PKA activation and a surge of Ca2+ into the mitochondrial matrix. Phosphorylation targets of PKA include lipases like HSL and ATGL and Plin5, which regulates PDM-LD-interaction and ATGL activity. Ca2+ flows into mitochondria through the mitochondrial calcium uniporter (MCU) and this the rise in mitochondrial matrix Ca2+ is followed by a sharp Ca2+decline due to the activation of Ca2+ efflux through the Na/Ca2+ exchanger NCLX. NCLX is responsible for preventing mitochondrial Ca2+ overload and is activated by mitochondrial PKA and inhibited by a phosphodiesterase PDE2A. How these responses are linked to the mitochondrial detachment and attachment to LDs are unclear and the molecular mechanisms regulating mitochondria-LD contacts upon adrenergic stimulation are not well known.

In the present work, the authors developed extensive in vitro reconstitution assays for studying the binding and detachment of isolated mitochondria and LDs under various in vitro conditions. They measured mitochondrial Ca2+ dynamics and function and mitochondria-LD interaction ex vivo and in brown adipocytes in conditions where they manipulated mitochondrial matrix Ca2+ levels by manipulating the function of mitochondrial Ca2+ transporters. Based on the results the authors suggest that elevation in mitochondrial matrix Ca2+, triggered by adrenergic stimulation, induces morphological changes in PDM and causes their detachment from LDs, thus facilitating lipolysis. The research topic is interesting and the effort to elucidate the mechanisms underlying mitochondrial detachment from LDs is well justified. However, the manuscript suffers from major conceptual issues and technical shortcomings.

Major problems:

1. While I do understand the idea of simplifying the experimental setup by reconstituting BAT mitochondria-LD interaction ex vivo, the significance of the data produced by these experiments is unclear. The reconstituted binding and detachment assays are questionable as the relevance of measuring binding and re-detachment of mitochondria to LDs after they have been mechanically detached is not obvious. Based on previous reports, it is not a surprising finding that Ca2+ would play a role in mitochondria-LD interaction. It seems that the effects of manipulating mitochondrial Ca2+ levels could have been tested directly in cells. Why are the drugs and conditions (RR, different fuels, NIM811, CGP, BAY60, and NCLX-KO) that are used to manipulate Ca2+ levels in the ex vivo experiments not systematically tested in brown adipocytes in culture together with NE stimulus? This would be a more straight-forward strategy to find out the relevance of Ca2+ signaling on PDM detachment in the functional cell setting.

2. There are a lot of confusing and contradictory results in the data panels that are not at all discussed in the text or disagree with the Method descriptions. Here just a few examples: In Fig 1D, addition of cytosol increases the binding of CM to LDs to the same level with PDM in the buffer and the attachment value is over 1.5, which is very high compared to values for PDM in other panels. Another example: In Fig 1L, there is clearly more MTDR signal left after Ca2+ detachment compared to the mechanically stripped sample. However, the text says "Confocal imaging analysis before and after RDA confirmed that Ca2+ induced the detachment of mitochondria from FL to the same extent as mechanical stripping of PDM (Figure 1L)". In Fig 2C, PDM detachment from FL is measured and detachment upon 10 uM Ca2+ is less than half of what is achieved mechanically. In Fig 3F, cold exposure has a stronger effect on PDM amount than what NCLX-KO has, in the following panels 3G,H this is the other way round. According to Methods, both intracellular and medium lipids were analyzed for TLC. In the only TLC data shown (Fig. 4L), only cellular lipids are shown. What about the medium lipids?

3. The in vivo data presented in the Fig. 5 feels disconnected from the rest of the story. Why are the in vivo mouse studies only done in an ob/ob mouse model? WT mice should also be analyzed for BAY60 effects. Why is the effect of BAY60 on mitochondria not studied under adrenergic stimulation / cold exposure in mice? According to the Methods, "For in vivo experiments, male and female mice of 10-12 weeks old were used". Does this imply that the results presented are pooled from both genders? If so, it would be good to present and comment on the similar effects between male and female mice, as there are often gender differences between these types of metabolic parameters.

4. It is often not clear how exactly each experiment or measurement was performed. Unfortunately, the extensive and somewhat unorthodox Supplementary Tables that attempt to summarize the experiments presented in the Figures, provide little help in this regard.

5. The overall structure of the manuscript and data presentation are not well designed, obscuring key findings. The figures have a huge amount of data, the presentation of which is not carefully designed and rigorously presented. It should be carefully considered which panels are presented in the main figures, what is presented in the supplement and what is left out from the manuscript.

Minor concerns:

6. The proposed mechanism of PDM detachment including a morphological change in the mitochondria is insufficiently tested. The sensitivity to Ca2+ and morphological changes are mentioned as a possible cause of PDM detachment, but this is not studied. In addition to panels Fig 2G,H with measurements of mitochondrial swelling there is no data on this - although this is part of the suggested mechanism of detachment.

7. Analysis of mitochondria-LD contacts in fluorescent images requires better quality images and images with higher magnification. In the current manuscript, it is unclear how the measurements of LD and mitochondria are done in the images with relatively low magnification (Fig. 3A,B,C and 4G,H).

Referee #3:

The present study reports the novel regulatory mechanism of peri-droplet mitochondria (PDM) dynamics in brown adipocytes. The authors established a new cell-free system that can quantify mitochondria-LD attachment and detachment. With this system, the authors showed that adrenergic stimulation triggers a surge in mitochondrial Ca^{2+} that drives the detachment of PDM from lipid droplets. The authors further showed that NCLX is required for PDM detachment from lipid droplets, and the PDE2A pathway negatively regulates NCLX and the PDM-LD attachment.

Together, the study elegantly demonstrated that mitochondrial Ca^{2+} efflux via NCLX regulates PDM dynamics and lipid catabolism. This is a tour de force that addresses an important biological question pertinent to mitochondrial dynamics in response to cold stimulus. The analyses are comprehensive, and the data are overall rigorous, supporting the conclusion of this paper. The reviewer suggests the following minor points that the authors wish to address prior to publication.

1. In Fig. 3, there is a large variation in the mt-LD contact between WT vs. NCLX KO mice (e.g., Fig.3F). The authors wish to discuss possible reasons that account for such variation. This also raises the possibility of compensatory pathways that are active in the absence of NCLX.

2. Related to the above point, the authors described that "Taken together, these results suggest that PDM detachment is dependent on mt-$Ca^{2+}$and that this process is regulated by NCLX." The authors should soften the sentences as the data may suggest regulatory mechanisms other than NCLX.

3. In Fig.4, the authors studied the effect of NE and Bay60 on PDM dynamics. It would be insightful to test if PDM detachment is still induced by NE signaling even when NE-stimulated lipolysis is blocked (e.g., ATGL inhibitor). NE and an ATGL inhibitor would stimulate cAMP signaling while lipid droplet size remains unchanged.

4. Energy expenditure data in Fig.5 needs some clarification. The authors should show the tracing of EE during the day and night. Also, the authors should examine how BAY60 alters RER. Additionally, the authors should show changes in food intake and locomotor activity.

Point by point response to referees

We thank the referees for their time, effort, and thoughtful comments. Their suggestions have substantially strengthened the manuscript, improving the robustness of the conclusions, the scientific accuracy, and the overall clarity.

Referee #1:

The manuscript entitled, "Mitochondrial Calcium Regulates Lipid Metabolism by Modulating Tethering of Mitochondria to Lipid Droplets" is an exciting study linking control of mitochondrial calcium efflux to their association with the lipid droplet and associated utilization of pyruvate rather than lipids for metabolic activity. Hence, the authors show that transient elevation of mitochondrial calcium activates the mitochondrial permeability transition in PDM (CM were resistant), however, the elevated NCLX activity observed in PDM facilitates recovery from the MPT and resumption of OxPhos. They further identify PDE activity within the mitochondria as a previously unappreciated regulator of NCLX activity. Finally, they show that treating ob/ob mice with the PDE inhibitor BAY60 leads to fat browning, dramatic shifts in substrate utilization towards sugars and increased metabolic utilization. While I found the study highly rational and informative; addressing the issues raised below will significantly increase readability and I urge you to consider these points carefully.

Major comments:

1. Figure 3B is an important panel showing disparate calcium efflux rates between CM and PDM. As shown, there appears to be one black line showing average cytosolic calcium content in CM and a lot of "red" representing PDM calcium? What this is really isn't very clear. In addition, if CM exhibits 2 different rates of efflux at different times, both should be plotted. And it should be possible to see where the rates were taken from the graph of cytosolic calcium concentration. As shown, this graph is very confusing and I struggled to appreciate what it really shows.

We thank the referee for highlighting this lack of clarity and the need to plot the rates of the 2 slopes. In this experimental set, we assess mitochondrial Ca2+ levels using a mitochondrial Ca^{2+} reporter, mt-GCaMP6. Changes in fluorescence are attributed to the levels of Ca^{2+} inside the mitochondrial matrix. In the revised manuscript, we have redrawn this panel to explicitly visualize and quantify the distinct Ca^{2+} efflux kinetics in CM versus PDM, with the mean efflux phases being overlaid. Now, we show linear fits used to calculate first- and second-phase slopes, with vertical markers drawn on the plot indicating the exact time windows used for each fit allowing the reader to see precisely where each rate was derived (NEW Figure 4B). Quantification of first- and second-phase slopes (NEW Figure 4D), percent efflux in the first phase (Figure 4C), and total AUC (NEW Figure 4E) is provided.

In the revised version, all Ca^{2+} traces were reanalyzed using consistent baseline normalization to the pre-NE period (F/F_0), and sample sizes and statistical analyses are detailed in the figure legend and Methods (Mitochondrial Ca2+ imaging and analysis). Please note, following re-analysis, the two-phase efflux pattern is preserved and quantified and remains evident, with the majority of Ca2+ clearance (~70–80%) occurring during the initial phase in CM while the remaining removal is slower in the second phase. Direct quantitative comparisons between CM and PDM are provided in the updated figure 4. Lastly, the two-phase kinetics also appear

across physiology and pharmacology and the second phase is likely linked to slower mitochondrial Ca2+ buffering NCLX-dependent fluxes which are regulated by MMP and PTMs can also affect the Ca2+ clearance kinetics. This phenomenon is quite common and it has been shown previously in other tissues such as neurons (Stavsky *et al*, 2021). The investigation of this kinetics, however, requires dedicated attention, which we will pursue in later studies.

2. There are a number of very interesting observations made about the MPT. I am somewhat surprised that there are no measurements of MMP. I would be particularly interested in seeing the MMP recovery that would be expected to accompany a transient MPT event.

Indeed, MMP measurements during the detachment in of PDM in intact cells could be revealing. We have published such images in Wikstrom et al EMBO 2014, and what one can see is the complexity of interpreting the MMP dynamics post NE. Given that brown adipocytes express UCP1 and that both CM and PDM UCP1 is activated, a transient depolarization induced by MTP will be replaced by that of UCP1. In the published data one can see that all mitochondria in the cell become depolarized, and once NE is removed, the entire mitochondrial population restores MMP to its baseline within 45 minutes.

However, the respirometry data is covering this point by looking at state 3 respiration. A low conductance open MPT would result in increased OCR, where a lethal MPT would abolish oxygen consumption. Figure 3F shows that concentrations that are detaching mitochondria from LDs do not increase or abolish respiration.

In principle, MMP could be measured in a plate reader using a *quenching* mode. However, during the detachment process, the mitochondrial population would comprise a heterogeneous mixture of PDM that remain attached to lipid droplets and those undergoing detachment. Because both populations would contribute to the MMP signal, the resulting measurement would be difficult to interpret unambiguously.

3. The sheer number of abbreviations was problematic. I do understand that they were defined, but many sentences were very challenging to follow. If there are space limitations, I must ask the journal to consider readability and permit you more words.

We thank the referee for this valuable suggestion which have now addressed in the revised version. <u>In the revised manuscript</u> we spell out some of the abbreviations (like ATGL: Adipose Triglyceride Lipase, HSL: Hormone Sensitive Lipase or MPC: Mitochondrial Pyruvate carrier) to make the text easier to read and follow since they appear less times in the manuscript. Some other abbreviations regarding names of buffer or reagents are now spelled out in the methods sections. We have modified the text and stick to only a reduced number of abbreviations that would be the more important ones. We have also gone through the text and identified sentences with more than 2 abbreviations and took care to spell them out.

We summarize the changes below, demonstrating that the number of abbreviations has been reduced by 45%.

Original list of abbreviations: [^{18}F]-FDG, ATGL, AU, BAT, BD493, Ca^{2+}, CM, DAPI, EE, EGTA, FFA, HSL, FL, LD, MAS, MCU, MPC, MRR, mt-PTP, NCLX, NE, OCR, OPA1, PC, PDE, PDM, PKA, Plin5, PM, RBA, RDA, RER, RR, RT, SDHA, SEM, Succ, TEM, TN, WAT, WT

Corrected list of abbreviations: BAT, BD493, Ca^{2+}, CM, EE, FFA, FL, LD, MCU, MTDR, NCLX, NE, PC, PDE, PDM, PM, RBA, RDA, RR, RT, TEM.

Minor Comments:

1. In reference to figure 1J, there is a statement that Calcium caused a 20% reduction in PDM attachment. Looking at the graph, this statement does not seem to be accurate.

We have now corrected the statement in the revised manuscript. We thank the reviewer for pointing out this mistake. Indeed, addition of calcium did not increase attachment when added on top of PC.

2. Lines 420 to 423, CM is used to describe data from both CM and PDM.

We acknowledge that the description of the results could be confusing. When describing the influx and efflux, we want to point out that although measured calcium influx was similar in CM and PDM, the efflux rates between the two mitochondrial populations are very different. The text now reads as follows:

"To distinguish peri-droplet mitochondria (PDM) from cytosolic mitochondria (CM), cells were co-stained with the neutral lipid dye BODIPY 558/568-C12 (BODIPY C12) one hour prior to imaging (Figure 4A). NE stimulation triggered an immediate increase in mitochondrial matrix Ca^{2+} in both PDM and CM, as indicated by a rapid rise in mt-GCaMP6 fluorescence (Figure 4A). Analysis of the initial slope of fluorescence increase revealed comparable rates of Ca^{2+} uptake in CM and PDM (Figure 4B–C), indicating similar Ca^{2+} influx kinetics.

In contrast, Ca^{2+} efflux kinetics differed markedly between the two mitochondrial populations. In CM, Ca^{2+} efflux rapidly returned matrix Ca^{2+} to near-basal levels within approximately 2 minutes of the NE-induced peak, followed by a slower secondary phase that reduced matrix Ca^{2+} below pre-stimulation baseline (Figure 4B). This biphasic behavior closely resembles previously reported mitochondrial Ca^{2+} dynamics in brown adipocytes exposed to NE (Assali et al., 2020). By comparison, PDM exhibited significantly slower Ca^{2+} efflux during both the rapid and slow phases of Ca^{2+} clearance (Figure 4C–D).

The cumulative consequence of these kinetic differences is captured by the area under the curve (AUC), which integrates matrix Ca^{2+} levels over time and was significantly greater in PDM than in CM (Figure 4E)."

Referee #2:

The manuscript by Acin-Perez, Assali and coworkers study the mechanisms of mitochondria-lipid droplet (LD) interactions in murine brown adipocyte derived material, building on earlier investigations. Previous reports have identified two distinct populations of mitochondria in brown adipose tissue, peridroplet mitochondria (PDM) that are attached to LDs and primarily oxidize pyruvate and cytosolic mitochondria (CM) that are located away from LDs and primarily oxidize fatty acids. It has been shown that adrenergic stimulation results in the detachment of PDM from LDs and leads to mitochondrial fragmentation, balling, uncoupling and lipid utilization. Adrenergic stimulation of brown adipose tissue also results in an increase in cAMP, PKA activation and a surge of Ca2+ into the mitochondrial matrix. Phosphorylation targets of PKA include lipases like HSL and ATGL and Plin5, which regulates PDM-LD-interaction and ATGL activity. Ca2+ flows into mitochondria through the mitochondrial calcium uniporter (MCU) and this the rise in mitochondrial matrix Ca2+ is followed by a sharp Ca2+decline due to the activation of Ca2+ efflux through the Na/Ca2+ exchanger NCLX. NCLX is responsible for preventing mitochondrial Ca2+ overload and is activated by mitochondrial PKA and inhibited

by a phosphodiesterase PDE2A. How these responses are linked to the mitochondrial detachment and attachment to LDs are unclear and the molecular mechanisms regulating mitochondria-LD contacts upon adrenergic stimulation are not well known.

In the present work, the authors developed extensive in vitro reconstitution assays for studying the binding and detachment of isolated mitochondria and LDs under various in vitro conditions. They measured mitochondrial Ca2+ dynamics and function and mitochondria-LD interaction ex vivo and in brown adipocytes in conditions where they manipulated mitochondrial matrix Ca2+ levels by manipulating the function of mitochondrial Ca2+ transporters. Based on the results the authors suggest that elevation in mitochondrial matrix Ca2+, triggered by adrenergic stimulation, induces morphological changes in PDM and causes their detachment from LDs, thus facilitating lipolysis. The research topic is interesting and the effort to elucidate the mechanisms underlying mitochondrial detachment from LDs is well justified. However, the manuscript suffers from major conceptual issues and technical shortcomings.

Major problems:

1. While I do understand the idea of simplifying the experimental setup by reconstituting BAT mitochondria-LD interaction ex vivo, the significance of the data produced by these experiments is unclear. The reconstituted binding and detachment assays are questionable as the relevance of measuring binding and re-detachment of mitochondria to LDs after they have been mechanically detached is not obvious. Based on previous reports, it is not a surprising finding that Ca2+ would play a role in mitochondria-LD interaction. It seems that the effects of manipulating mitochondrial Ca2+ levels could have been tested directly in cells. Why are the drugs and conditions (RR, different fuels, NIM811, CGP, BAY60, and NCLX-KO) that are used to manipulate Ca2+ levels in the ex vivo experiments not systematically tested in brown adipocytes in culture together with NE stimulus? This would be a more straight-forward strategy to find out the relevance of Ca2+ signaling on PDM detachment in the functional cell setting.

We thank the reviewer for bringing this point up and are happy we have the chance to explain the rationale, and to address the comment with additional experiment.

First, the *rationale* for presenting two complementary *ex vivo* approaches in intact cells and cell-free systems, is to determine whether the tested signals act *directly* on mitochondria and lipid LDs. This strategy parallels classic reductionist experiments (for example showing that microtubules can drive movement or that ribosomes can translate proteins in cell-free systems). In our case, cell-free assays were indispensable because they allow direct interrogation of mitochondrial-LD interactions without confounding extra-mitochondrial factors such as organelle motility and cellular architecture, which strongly affect attachment and detachment dynamics in intact cells and therefore cannot be performed reliably in intact cells alone. In this context, the reductionist approach provides mechanistic insights that cannot be obtained reliably from intact-cell experiments alone and is therefore essential for disentangling direct mitochondrial-LD interactions from indirect cellular contributions. In the revised manuscript we explain the rationale both int eh results and in the discussion.

Importantly, we did not rely solely on the cell free system. As the referee rightfully suggested, we also tested the same interventions in intact cellular contexts, including: NE and Ru360 (now in Figure 2 and Figure EV2), Triacsin C (now Figure EV2), BAY60 (now Figure 5) and studies using $NCLX^{KO}$ – derived BAT *in vitro* in primary BA and tissue.

In addition, in the revised manuscript we add new imaging data in primary BA treated with NE and Ru360, CGP, ATGLstatin and palmitate in the revised Figure 2 and an additional panel in Figure 4 (Figure 4L-N). We have summarized all the data in pBA in a new table (Table 2).

Together, these complementary approaches ensure that our conclusions are supported by both reductionist mechanistic assays and physiologically relevant intact-cell experiments.

2. There are a lot of confusing and contradictory results in the data panels that are not at all discussed in the text or disagree with the Method descriptions.

We acknowledge the reviewer for his/her comment. We describe below how we have addressed each of the reviewer's concerns.

Fig 1D, addition of cytosol increases the binding of CM to LDs to the same level with PDM in the buffer and the attachment value is over 1.5, which is very high compared to values for PDM in other panels

The reviewer is correct. Comparing original Figure 1D and 1I, it may seem that the effect is different; however, in the experiments in Figure 1I, the assays were performed in MAS buffer in the absence of isolated cytosol to specifically address the contribution of the substrates to the attachment of PDM to LD. We have now clarified this in the text as well as the figure legend. Thank you for pointing this out!

In other panels, Figure E-H, we are presenting the data as relative to the BAT RT conditions and not the absolute values. In the revised manuscript we clarify that point in the figure legend.

In Fig 1L, there is clearly more MTDR signal left after Ca2+ detachment compared to the mechanically stripped sample. However, the text says "Confocal imaging analysis before and after RDA confirmed that Ca2+ induced the detachment of mitochondria from FL to the same extent as mechanical stripping of PDM (Figure 1L)"

We agree with the reviewer that the image shown in the original Figure 1L, now Figure 2B, displays a stronger MTDR signal in the Ca^{2+}-induced detachment condition; however, this signal originates from mitochondria that are *not* attached to LDs. The purpose of this experiment was to confirm, using an imaging-based approach, the plate reader results demonstrating that Ca^{2+} promotes PDM detachment. A key advantage of the imaging approach is that it allows us to avoid centrifugation, which would otherwise remove detached mitochondria and potentially bias the analysis. In panel 2B, we image mitochondria and LDs and use quantitative image analysis to assess both the fraction of PDM that remain attached to LDs and LD size. To further support our conclusion, in the revised manuscript we add new statistical analyses of the remaining PDM and LD size following Ca^{2+} treatment, shown in panels 1M and 1N. We have also added text clarifying that in 1L we did not remove the detached mitochondria from the sample and they can be seen floating in between LDs. Now it reads as follows:

"To exclude the possibility that centrifugation artifacts contributed to the observed detachment, we independently validated the effect of Ca^{2+} using confocal imaging of FL before and after Ca^{2+} exposure. Imaging revealed that addition of Ca^{2+} resulted in LDs retaining a reduced number of attached PDM, while detached mitochondria were visible in the surrounding medium (Figure 2B). Quantitative analysis showed that both mechanical stripping and Ca^{2+}-induced detachment reduced PDM abundance without altering LD size (Figure 2C–D), confirming that detachment occurs independently of LD shrinkage, since LD diameter was unchanged. These findings corroborate the biochemical assays and are consistent with Figure 1A, supporting the conclusion that lipolysis occurs downstream of mitochondrial detachment."

In Fig 2C, PDM detachment from FL is measured and detachment upon 10 uM Ca2+ is less than half of what is achieved mechanically.

The reviewer is correct. Mechanically stripping is always more effective than Ca^{2+} mediated detachment. Previous studies indeed demonstrated that following activation of thermogenesis, LDs can retain a small fraction of mitochondria still attached. For the following explanation, please note that the original Figure 2 is now Figure 3. The combination of Ca^{2+} and PC is the one that mimics best the mechanically stripped condition. The purpose of the experiments shown now in Figures 3E-H is to compare physiological Ca^{2+} vs Ca^{2+} overload (overload means a concentration that has been shown to trigger mitochondrial apoptosis). In the revised manuscript, in the new Figure 4K we compare the detachment efficiency of mechanical striping, PC alone, Ca^{2+} plus PC and Ca plus PC plus NCLX inhibition.

In Fig 3F, cold exposure has a stronger effect on PDM amount than what NCLX-KO has, in the following panels 3G,H this is the other way round.

We thank the referee for raising this point. The results are not contradictory. In panel G (now Figure 4G), the $NCLX^{KO}$ starts from a lower baseline at room temperature (RT) compared with WT; consequently, the temperature-dependent change is smaller in the KO than in the WT. As stated in the manuscript, NCLX deletion modulates but does not completely abolish the onset of PDM dissociation.

The key message of the original panels 3F-H (now new Figure 4H-I) is that WT and NCLX KO cells display distinct PDM levels, as measured by two independent methods (TEM and fluorescent-dye–based PDM quantification), and that cold exposure reduces PDM in both genotypes, with a more pronounced effect in the $NCLX^{KO}$. Panel H compares PDM levels at RT between WT and $NCLX^{KO}$, whereas panel I shows PDM levels after exposure to 4°C.

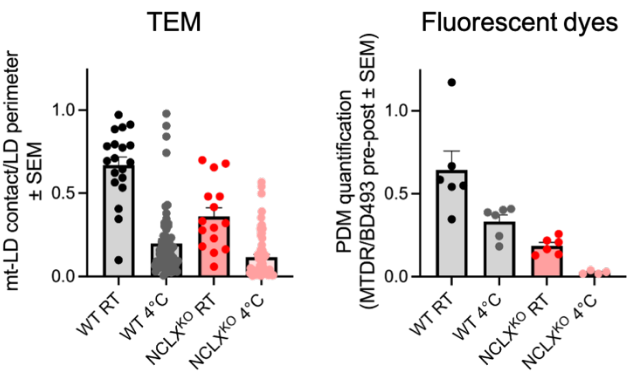

For clarity, in the revised manuscript we are separating the data into two graphs (RT and cold exposure). If all conditions were plotted together, as in panel G, the resulting graph would show the same trends. We hope this clarification resolves the reviewer's concern and demonstrates that the findings are internally consistent.

Furthermore, in the revised manuscript we provide a new heatmap, now Figure 4N, where we compared this analysis together with the imaging in pBA to show that cold stimulation in control conditions is very similar to NCLX activity inhibition and that inhibition of NCLX and adrenergic stimulation is always the condition with less PDM.

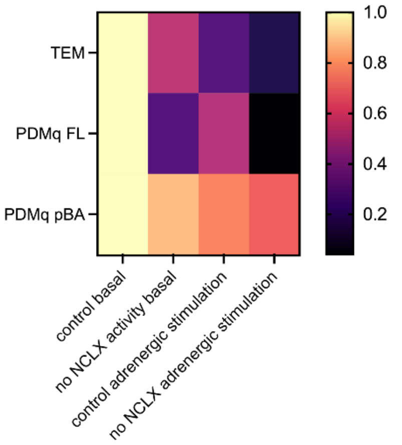

According to Methods, both intracellular and medium lipids were analyzed for TLC. In the only TLC data shown (Fig. 4L), only cellular lipids are shown. What about the medium lipids?

We used the media to assess glycerol release. We acknowledge the reviewer for pointing it out. In the revised manuscript we have revised the methods accordingly. Note that the original Figure 4 is now Figure 5 in the revised version.

3. The in vivo data presented in the Fig. 5 feels disconnected from the rest of the story. Why are the in vivo mouse studies only done in an ob/ob mouse model? WT mice should also be analyzed for BAY60 effects. Why is the effect of BAY60 on mitochondria not studied under adrenergic stimulation / cold exposure in mice? According to the Methods, "For in vivo experiments, male and female mice of 10-12 weeks old were used". Does this imply that the results presented are pooled from both genders? If so, it would be good to present and comment on the similar effects between male and female mice, as there are often gender differences between these types of metabolic parameters.

We thank the reviewer for this comment. Although inclusion of both sexes would have been ideal, these experiments were performed in male mice only; we apologize for the error in the original methodological description, which has now been corrected in the revised manuscript.

We focused on *ob/ob* mice because their brown adipose tissue (BAT) has reduced peri-droplet mitochondria (PDM) content, as we previously reported (Acin-Perez et al., 2021). This model therefore provides a stringent context in which to test whether modulation of NCLX activity and the PDE2 pathway (using Bay-607550) can increase PDM content. Therefore, ob/ob model is expected to provide a wider dynamic range for the restoration of attachment, not for the increase in detachment.

Acute cold exposure is not suitable for *ob/ob* mice, as they have low UCP1 levels and require prior cold acclimation (Milner & Trayhurn, 1989; Smith & Romsos, 1984). Moreover, cold exposure strongly enforces PDM detachment, leaving little dynamic range in mice that already have low baseline PDM levels. For these reasons, we conducted the study at room temperature, which is already considered a mild cold stress in this model and was well tolerated, whereas direct cold exposure was not.

In the revised manuscript we also toned down the conclusions drawn from this experiment pertaining to browning and energy expenditure. We explain both in the results and in the discussion that this experiment alone cannot be used to draw a conclusion that increase in

PDM is sufficient to induce browning or an increase in energy expenditure. When combined with the cell free reconstituted system, we can conclude that inhibition of mitochondrial PDE2A is sufficient to induce mitochondrial association with LDs, confirming that induction of PDM formation in ob/ob mice is not dependent on browning.

The original Figure 5 is now presented as Figure 6 in the revised manuscript.

4. It is often not clear how exactly each experiment or measurement was performed. Unfortunately, the extensive and somewhat unorthodox Supplementary Tables that attempt to summarize the experiments presented in the Figures, provide little help in this regard.

We have included the tables as supportive, summarized material that could help the reader to understand better the question asked in each experimental set up. We apologize that the tables were not clear enough and we have tried to make them easier to follow in this new version of the manuscript. We have discussed this point with the editor and we have followed his indications to summarize the experiments in a clearer way. Following his recommendations, we have included the tables as part of the main text and not as supplementary data.

5. The overall structure of the manuscript and data presentation are not well designed, obscuring key findings. The figures have a huge amount of data, the presentation of which is not carefully designed and rigorously presented. It should be carefully considered which panels are presented in the main figures, what is presented in the supplement and what is left out from the manuscript.

We apologize to the reviewer on how the data have been presented. In the revised manuscript we have included new panels (Figure 2G-H, Figure EV2 and Figure 4L-M) focused in analyzing detachment in pBA by imaging techniques. We have also added additional panels to clarify the experimental outcome and to address some of the reviewers' concerns. In Figure 2, we have included panel C and D to quantify LD size and PDM mount in the FL imaging experiments. In the new Figure 3, we have added the valinomycin induced swelling experiment (Figure 3A) and the effect of valinomycin in detachment (Figure 3B). In the new Figure 4, we have added imaging pf pBA upon NCLX inhibition with CGP and the corresponding image analysis (Figure 4L-M). Finally in Figure EV4, we have added the panels corresponding to EE profiles at day and night (Figure EV4G) and to food intake (Figure EV4H) and activity (Figure EV4I).

Minor concerns:

6. The proposed mechanism of PDM detachment including a morphological change in the mitochondria is insufficiently tested. The sensitivity to Ca2+ and morphological changes are mentioned as a possible cause of PDM detachment, but this is not studied. In addition to panels Fig 2G,H with measurements of mitochondrial swelling there is no data on this - although this is part of the suggested mechanism of detachment.

We thank the reviewer for his suggestion. We indeed have data on RDA with PC+Ca^{2+} and in the presence/absence of NIM811 plotted in Figure 2C, while the swelling data are presented in Figure 2I-J.

In the revised manuscript we included new data where we performed new detachment assays where we compare the effect of swelling in the absence of Ca^{2+} using valinomycin. The new data reveal that swelling in the presence of valinomycin can induce detachment per se. We have included this new data in Figure 3A-B.

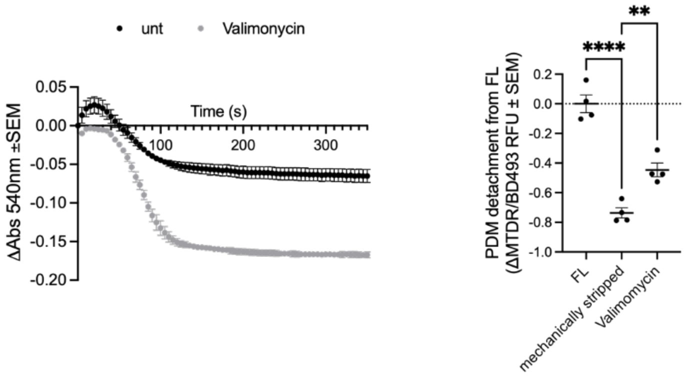

We have also performed image analysis in the samples obtained from the cell free assay and we can show that Ca2+ induce detachment does not change the LD size. These data are now presented in the new Figure 2C-D. This is probably due to the fact that to achieve lipolysis both signaling branches activated upon adrenergic stimulation (Ca^{2+} and PKA activation) need to be triggered.

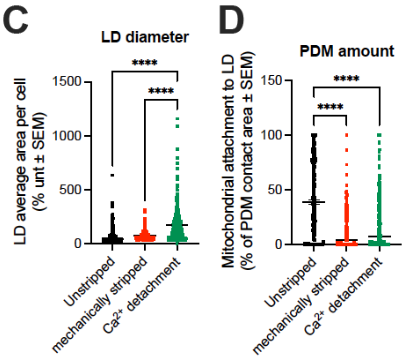

7. Analysis of mitochondria-LD contacts in fluorescent images requires better quality images and images with higher magnification. In the current manuscript, it is unclear how the measurements of LD and mitochondria are done in the images with relatively low magnification (Fig. 3A,B,C and 4G,H).

In the revised manuscript we are including a more detailed description on how PDM and LD quantification were performed using high resolution imaging. Now the method section on this matter reads as follows:

Peridroplet Mitochondria and Fat Layer imaging

Imaging of fat layer was done using the on a Zeiss LSM880 microscope. Samples were stained with MTDR and BD493, subjected to the different treatments and embedded in 1% agarose.

Fluorescence microscopy and image analysis

Whole Mount tissues were imaged using a Zeiss LSM 880 confocal microscope with Airyscan super-resolution detector using a 40x Apochromat oil objective.

Super resolution live cell imaging and fat layer imaging were performed on a Zeiss LSM880 using a 63x Plan-Apochromat oil-immersion lens and AiryScan super-resolution detector with humidified 5 % CO2 chamber on a temperature-controlled stage (37°C).

Referee #3:

The present study reports the novel regulatory mechanism of peri-droplet mitochondria (PDM) dynamics in brown adipocytes. The authors established a new cell-free system that can quantify mitochondria-LD attachment and detachment. With this system, the authors showed that adrenergic stimulation triggers a surge in mitochondrial Ca2+ that drives the detachment of PDM from lipid droplets. The authors further showed that NCLX is required for PDM detachment from lipid droplets, and the PDE2A pathway negatively regulates NCLX and the PDM-LD attachment.

Together, the study elegantly demonstrated that mitochondrial Ca2+ efflux via NCLX regulates PDM dynamics and lipid catabolism. This is a tour de force that addresses an important biological question pertinent to mitochondrial dynamics in response to cold stimulus. The analyses are comprehensive, and the data are overall rigorous, supporting the conclusion of this paper. The reviewer suggests the following minor points that the authors wish to address prior to publication.

1. In Fig. 3, there is a large variation in the mt-LD contact between WT vs. NCLX KO mice (e.g., Fig.3F). The authors wish to discuss possible reasons that account for such variation. This also raises the possibility of compensatory pathways that are active in the absence of NCLX.

This is a very interesting point. In the revised manuscript we are including additional detailed explanation of the possible compensatory pathways that could be taking part in the absence of NCLX.

Now it reads as follows:

"Several limitations of this study warrant consideration. ….. loss of NCLX may trigger compensatory responses that limit mitochondrial Ca^{2+} accumulation. Possible mechanisms include increased activity of secondary alternative mitochondrial Ca^{2+} extrusion routes, modulation of ER-mitochondria Ca transfer through altered contact site dynamics, and metabolic reprogramming that reduces Ca^{2+}-sensitive fluxes. Such adaptations could partially preserve mitochondrial function in NCLX-deficient BAT and warrant targeted investigation. Nevertheless, our data indicate that Ca^{2+} efflux is significantly impaired in the absence of NCLX, producing mitochondrial Ca^{2+} overload as a downstream consequence."

.

2. Related to the above point, the authors described that "Taken together, these results suggest that PDM detachment is dependent on mt-Ca2+and that this process is regulated by NCLX." The authors should soften the sentences as the data may suggest regulatory mechanisms other than NCLX.

We acknowledge the reviewer for his /her comment. We have now fixed the text and softened the conclusion, Now the Results section text reads as follows "*Together, these results*

demonstrate that PDM detachment is governed by mitochondrial matrix Ca^{2+} handling and is regulated, at least in part, by NCLX-mediated Ca^{2+} extrusion."

And in the discussion "although our data identify matrix Ca^{2+} and the PDE2A–NCLX axis as central regulators of PDM positioning, we cannot exclude contributions from additional post-translational modifications or structural tethering proteins that may cooperate with Ca^{2+} signaling"

3. In Fig.4, the authors studied the effect of NE and Bay60 on PDM dynamics. It would be insightful to test if PDM detachment is still induced by NE signaling even when NE-stimulated lipolysis is blocked (e.g., ATGL inhibitor). NE and an ATGL inhibitor would stimulate cAMP signaling while lipid droplet size remains unchanged.

We thank the reviewer for this comment and the suggested experiment. In the revised version, we are including new image analysis on LD size and PDM amount in primary BA in the presence of NE and ATGLstatin to block lipolysis. The results are now presented in the new Figure 2F-H and Figure EV2A-B.

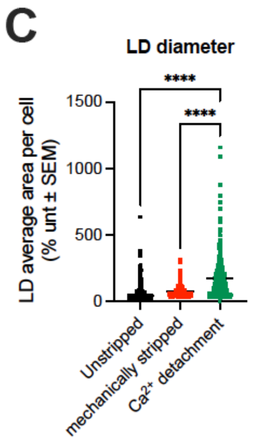

In addition, we performed NEW cell-free detachment assays in the presence of Calcium and assessed LD size after detachment by imaging. We find that detachment alone does not promote LD shrinking by itself, but it rather facilitates lipases access once PDM are removed (detached), then to allow lipolysis (Figure 1A), thereby detachment is necessary to kick-in lipolysis but not sufficient. We have included the new set of data on detachment without changes in LD size in the new Figure 2C.

Please see the schematic drawing highlighting the process:

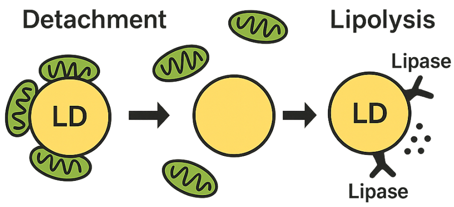

4. Energy expenditure data in Fig.5 needs some clarification. The authors should show the tracing of EE during the day and night. Also, the authors should examine how BAY60 alters RER. Additionally, the authors should show changes in food intake and locomotor activity.

We thank the reviewer for this comment. We did not include the EE traces during the day and night period in the initial submission; we just showed the quantification of the EE in different periods to simplify the data description. In the revised version we are including the new data as suggested by the reviewer (see below) as part of the new Figure EV4, together with the RER data that were placed there in the first version of the manuscript.

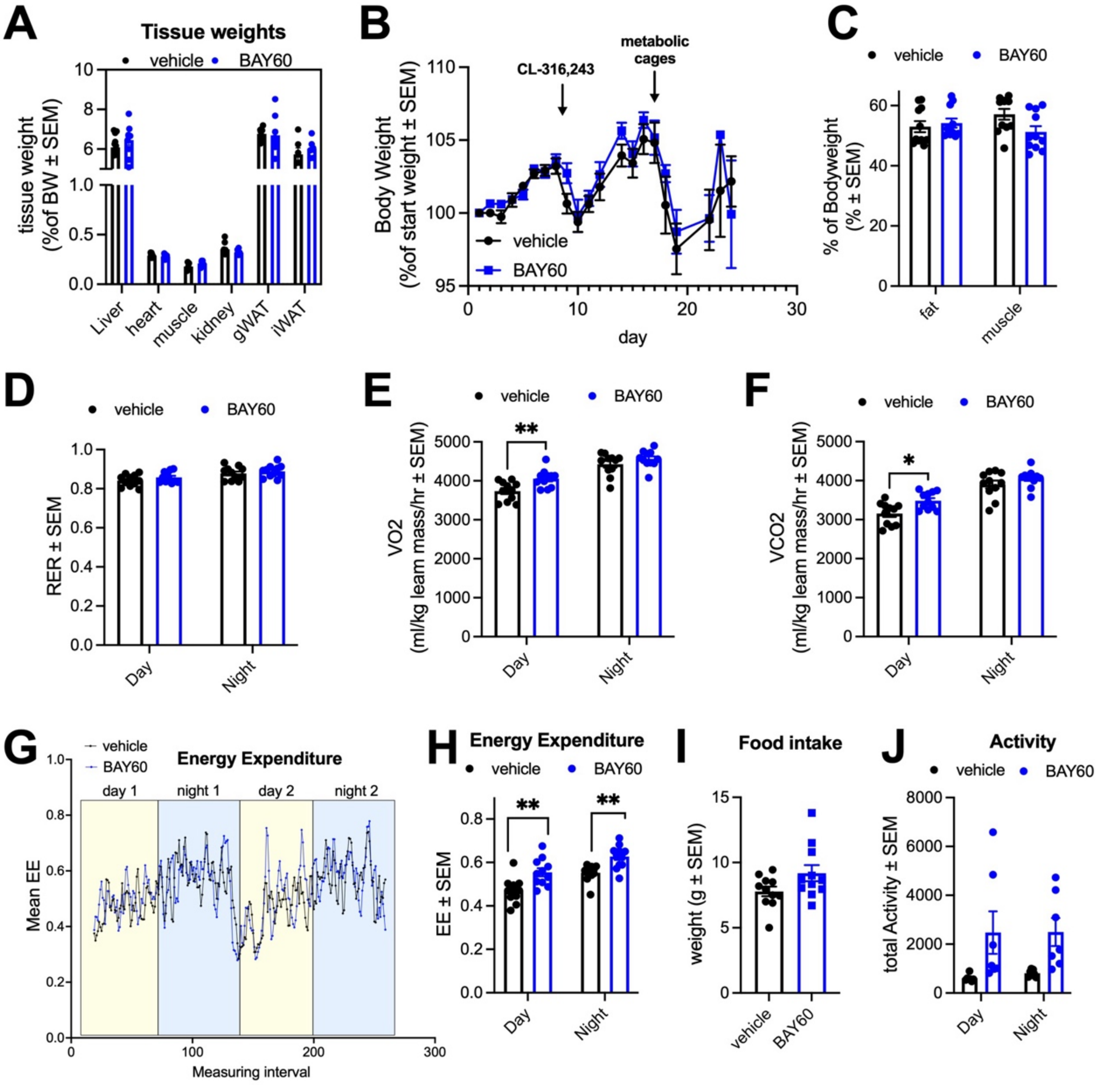

References

Acin-Perez R, Petcherski A, Veliova M, Benador IY, Assali EA, Colleluori G, Cinti S, Brownstein AJ, Baghdasarian S, Livhits MJ *et al* (2021) Recruitment and remodeling of peridroplet mitochondria in human adipose tissue. *Redox Biol* 46: 102087

Milner RE, Trayhurn P (1989) Cold-induced changes in uncoupling protein and GDP binding sites in brown fat of ob/ob mice. *Am J Physiol* 257: R292-299

Smith CK, Romsos DR (1984) Cold acclimation of obese (ob/ob) mice: effects of energy balance. *Metabolism* 33: 853-857

Stavsky A, Stoler O, Kostic M, Katoshevsky T, Assali EA, Savic I, Amitai Y, Prokisch H, Leiz S, Daumer-Haas C *et al* (2021) Aberrant activity of mitochondrial NCLX is linked to impaired synaptic transmission and is associated with mental retardation. *Commun Biol* 4: 666

Dear Orian,

Thank you again for submitting your amended manuscript (EMBOJ-2025-122194R) to The EMBO Journal.

Your revised study was sent back to the referees for their scientific reassessment, and we have received re-reports from all of them, which I enclose below. As you will see, the referees state that the work has been substantially enhanced by the revisions and they are now in favour of publication, pending minor revision.

Thus, we are pleased to inform you that your manuscript has been accepted in principle for publication in The EMBO Journal.

Please carefully consider the remaining minor issues still raised by referee #2 by adjusting data presentation and discussion of the findings in the manuscript text.

Also, we now need you to take care of a number of minor issues related to formatting and data annotation, as detailed below, together with additional changes and requests by our production team for Source Data provision.

As you might remember from previous experience, every paper at the EMBO Journal now includes a 'Synopsis', displayed on the html and freely accessible to all readers. The synopsis includes a 'model' figure as well as 2-5 one-short-sentence bullet points that summarize the article. I would appreciate if you could provide this figure and the bullet points.

Please submit a revised version of the manuscript using the link enclosed below, addressing the advisor's comments.

Thank you again for giving us the chance to consider your manuscript for The EMBO Journal, I look forward to hearing from you and receiving your final revised version of the manuscript for swift acceptance and publication.

Kind regards,

Daniel

Daniel Klimmeck PhD
Senior Editor
The EMBO Journal

****

Formatting changes required for the revised version of the manuscript:

>> Please limit the keywords for your study to maximally five.

>> Author information: please resolve the name discrepancy between Frankie Villalobos (manuscript) vs Francisco Villalobos (online system).

>> Author Contributions: Remove the author contributions information from the manuscript text. Note that CRediT has replaced the traditional author contributions section as of now because it offers a systematic machine-readable author contributions format that allows for more effective research assessment. and use the free text boxes beneath each contributing author's name to add specific details on the author's contribution.

More information is available in our guide to authors.
https://link.springer.com/journal/44318/submission-guidelines

>> Section order should be as follows: title page with complete author information, abstract, keywords, introduction, results, discussion, methods, data availability section, acknowledgements, disclosure and competing interests statement, references, main figure legends, tables, expanded figure legends.´.

>> Adjust the title of the 'Declaration of Interests' section to 'Disclosure and Competing Interests Statement'.

>> Highlights need to be removed from the manuscript.

>> The 'Acronyms' abbreviations section needs to be removed from the manuscript. Abbreviations should be defined in brackets after their first mention in the text, not in a list of abbreviations.

>> 'Experimental Procedures' need to be labeled as 'Methods'.

>> Figure callouts: please enter a callout for a Fig. 2D in the running main text.

>> Add a Reagents and Tools table to the Methods section, as a separate file using the existing template in the Guide For Authors, listing key reagents, experimental models, software and relevant equipment.

>> Figures in separate files: EV figures should be uploaded as individual files if they are staying as EV figures.

>> Appendix file with ToC / EV figures included in Appendix file:

option 1: if they are staying in Appendix nomenclature and callouts to be adjusted (Appendix Figure S1 etc.); legends need to be removed from the manuscript; Appendix file needs title page with: Appendix for 'manuscript title' and Table of contents with each item listed with page number

option 2: they are uploaded as individual figure files, nomenclature stays as Figure EV1 etc.; legends at the end of the manuscript (Expanded View Figure Legends)

>>Source Data:

- Figure files and schematics should be removed from the source data folders

- There are some discrepancies in the completed checklist and source data required/provided, Fig 1 I,I are missing in the checklist but numerical data has been provided, Fig 2 lists images where only numerical data should be, etc. As this is an internal file, is this OK or should it be corrected?

- Fig 6H contains two large .xif files that I cannot open. Could an alternative image file format be used instead? Ideally Figure 6 source data should be uploaded as one ZIP file. If image files are too large, consider depositing them to BioStudies and adding a link in the Data Availability section.

- Source data folder for Fig 7 can be removed as it has no source data

>> Dataset EV legends: Tables 1-4 to be either uploaded as EV tables - nomenclature and callouts to be adjusted to Table EV1 etc.; legends included in the file; docx or xlsx file format; Might be better to move them to Appendix (nomenclature Appendix Table S1 etc.).

>> Consider additional changes and comments from our production team as indicated below:

- Figure legends:
1. Please note that the exact p values are not provided in the legends of figures 1A, D, E-J; 2A, C, D, E, G, H, I; 3B, C, D, E, F, G, H, I, J, N, P; 4C, D, E, G, H, I, J, K, M; 4C, D, E, G, H, I, J, K, M; 5A, B, E, F, H, J, K, L, M; 6A-E, F, G, I, J, L; EV4 E, F, H
2. Please indicate the statistical test used for data analysis in the legends of figures 1A, D, E-J; 2A, C, D, E, G, H, I; 3B, C, D, E, F, G, H, I, J, N, P; 4C, D, E, G, H, I, J, K, M; 5A, B, E, F, H, J, K, L, M; 6A-E, F, G, I, J, L; EV2 A, B; EV4 E, F, H
3. Please note that information related to n is missing in the legends of figures 1A , 2E, G, H; 4M, 5A, B; EV1 C, EV2 A, B, D.
4. Please note that the error bars are not defined in the legends of figures EV3 C, D, F, G.
5. Please note that the scale bar needs to be defined for figure EV2 C

Further information is available in our Guide For Authors: https://link.springer.com/journal/44318/submission-guidelines

----

Referee #1:

All of my comments have been addressed and I have no further concerns.

Referee #2:

The revised manuscript by Acin-Perez, Assali et al has successfully addressed most of the points raised and overall, the manuscript is improved. Yet, there is still some curious lack of attention to detail even in the revised version. The authors should carefully scrutinize the text to eliminate mistakes/inaccuracies, as this cannot be left to the reviewer.

Specific examples:

1. Mitotracker Deep Red ia not a membrane potential-independent mitochondrial dye (line 150).
2. The results state that the LD diameter is unchanged (line 290) but Figure 2C shows that it changes.
3. In several experiments, the treatment times are not provided (e.g. Figure 2F, Figure 5M). In Fig. 5M, the glucose content of the medium is apparently reduced from 5 mM to about 2 mM by NE+BAY60. How long does this take?
4. According to Figure legend (line 914) and Results (line588), mitochondria adjacent to LDs were quantified as mitochondrial pixels located within 0. 3 microns of a lipid droplet and according to Methods (line 1285), 0.5 microns of lipid droplet edge.
5. The Discussion is now twice as long as originally. There are indeed several interesting points worth discussing, but this section could be condensed by avoiding the repetition of Results.
(In the responses to Reviewer, the authors refer to panels 1L, M and N, but Figure 1 does not have such panels.)

Referee #3:

The authors addressed the reviewer's comments satisfactorily. No more comments from this reviewer.

Referee #1:

All of my comments have been addressed and I have no further concerns.

Referee #2:

The revised manuscript by Acin-Perez, Assali et al has successfully addressed most of the points raised and overall, the manuscript is improved. Yet, there is still some curious lack of attention to detail even in the revised version. The authors should carefully scrutinize the text to eliminate mistakes/inaccuracies, as this cannot be left to the reviewer.

Specific examples:

1. Mitotracker Deep Red ia not a membrane potential-independent mitochondrial dye (line 150).

We have previously published a protocol for the use of MTDR as a mitochondrial mass indicator where we verified that MTDR can stain depolarized mitochondria. In the revised version we are now citing that protocol paper as well as a recent review by Marc Liesa that goes over the use of membrane potential dyes (PMID: 41249505) We also revised the text to say "MTDR that binds both depolarized and polarized mitochondria ".

2. The results state that the LD diameter is unchanged (line 290) but Figure 2C shows that it changes.

The referee is correct and we fixed this. The purpose of the sentence was to assure that mitochondria did not leave the LD in response to a potential decrease in the surface area of the LDs. This is to say that lipolysis did not occur in the cell-free system. Therefore, the proper language should be that "LD surface area did not decrease".

Indeed, as the referee says, LD diameter showed a non-significant increase, which may be due to LD fusion, a very interesting phenomenon to consider for future research.

Now it reads as follows: Quantitative analysis showed that both mechanical stripping and Ca^{2+}-induced detachment reduced PDM abundance without reducing LD size (Figure 2C–D), confirming that detachment did not require LD shrinkage, as LD diameter was not decreased.

3. In several experiments, the treatment times are not provided (e.g. Figure 2F, Figure 5M).

Duration of treatments have been included in the text and figure legends.

In Fig. 5M, the glucose content of the medium is apparently reduced from 5 mM to about 2 mM by NE+BAY60. How long does this take?

We have added the duration time in the results description as well and in the Figure legend. Treatment time was 6 hours.

4. According to Figure legend (line 914) and Results (line588), mitochondria adjacent to LDs were quantified as mitochondrial pixels located within 0. 3 microns of a lipid droplet and according to Methods (line 1285), 0.5 microns of lipid droplet edge.
We thank the reviewer for this observation. We have changed it now and it is consistent in the text and methods.

5. The Discussion is now twice as long as originally. There are indeed several interesting points worth discussing, but this section could be condensed by avoiding the repetition of Results.
We have shortened it following the reviewers suggestion.
We believe that the discussion summarizes the results in line with the state of the art in the field without being too redundant in the results description.

(In the responses to Reviewer, the authors refer to panels 1L, M and N, but Figure 1 does not have such panels.)
Thank you for noticing this. Those were the former panel numbers. We have carefully revised the text to make sure they are properly called out.

Referee #3:

The authors addressed the reviewer's comments satisfactorily. No more comments from this reviewer.

2nd Revision - Editorial Decision 21st May 2026

Dear Orian,

Thank you for submitting the revised version of your manuscript. I have now evaluated your amended manuscript and concluded that the remaining minor concerns have been sufficiently addressed.

I am thus pleased to inform you that your manuscript has been accepted for publication in the EMBO Journal.

Your manuscript will be processed for publication by EMBO Press. It will be copy edited and you will receive page proofs prior to publication. Please note that you will be contacted by Springer Nature Author Services to complete licensing and payment information.

Please note that it is The EMBO Journal policy for the transcript of the editorial process (containing referee reports and your response letter) to be published as an online supplement to each paper. If you do NOT want this, you will need to inform the Editorial Office via email immediately. More information is available here: https://www.embopress.org/transparent-process#Review_Process

You may qualify for financial assistance for your publication charges - either via a Springer Nature fully open access agreement or an EMBO initiative. Check your eligibility: https://www.embopress.org/page/journal/14602075/authorguide#chargesguide

Should you be planning a Press Release on your article, please notify the production team via this form in order to coordinate publication and release dates: https://forms.cloud.microsoft/e/eH4A8JVP6g

If you have any questions, please do not hesitate to contact the Editorial Office. Thank you for your contribution to The EMBO Journal.

Thank you again for this contribution to The EMBO Journal and congratulations on a successful publication! Please consider us again in the future for your most exciting work.

Best regards,

Daniel

Daniel Klimmeck, PhD
Senior Editor
The EMBO Journal
EMBO
Postfach 1022-40
Meyerhofstrasse 1
D-69117 Heidelberg
contact@embojournal.org

*****
Please note that it is The EMBO Journal policy for the transcript of the editorial process (containing referee reports and your response letters) to be published as an online supplement to each paper. If you should prefer removal of any referee-only figures included in the point-by-point response(s), e.g. because they may still be used for future publication or because they have been reproduced from published work by others, please do let us know immediately via response email.
More information is available here: https://link.springer.com/partners/embo-press/editorial-policies#Peer%20review

EMBO Press Author Checklist

Corresponding Author Name: Orina S Shirihai	
Journal Submitted to: EMBO J	
Manuscript Number: Submission Summary	

USEFUL LINKS FOR COMPLETING THIS FORM
The EMBO Journal - Author Guidelines
EMBO Reports - Author Guidelines
Molecular Systems Biology - Author Guidelines
EMBO Molecular Medicine - Author Guidelines

Reporting Checklist for Life Science Articles (updated January
This checklist is adapted from Materials Design Analysis Reporting (MDAR) Checklist for Authors. MDAR establishes a minimum set of requirements in transparent reporting in the life sciences (see Statement of Task: 10.31222/osf.io/9sm4x). Please follow the journal's guidelines in preparing your
Please note that a copy of this checklist will be published alongside your article.

Abridged guidelines for figures
1. Data
The data shown in figures should satisfy the following conditions:
- the data were obtained and processed according to the field's best practice and are presented to reflect the results of the experiments in an accurate and unbiased manner.
- ideally, figure panels should include only measurements that are directly comparable to each other and obtained with the same assay.
- plots include clearly labeled error bars for independent experiments and sample sizes. Unless justified, error bars should not be shown for technical
- if n<5, the individual data points from each experiment should be plotted. Any statistical test employed should be justified.
- Source Data should be included to report the data underlying figures according to the guidelines set out in the authorship guidelines on Data

2. Captions
Each figure caption should contain the following information, for each panel where they are relevant:
- a specification of the experimental system investigated (eg cell line, species name).
- the assay(s) and method(s) used to carry out the reported observations and measurements.
- an explicit mention of the biological and chemical entity(ies) that are being measured.
- an explicit mention of the biological and chemical entity(ies) that are altered/varied/perturbed in a controlled manner.
- the exact sample size (n) for each experimental group/condition, given as a number, not a range;
- a description of the sample collection allowing the reader to understand whether the samples represent technical or biological replicates (including how many animals, litters, cultures, etc.).
- a statement of how many times the experiment shown was independently replicated in the laboratory.
- definitions of statistical methods and measures:
  - common tests, such as t-test (please specify whether paired vs. unpaired), simple χ2 tests, Wilcoxon and Mann-Whitney tests, can be unambiguously identified by name only, but more complex techniques should be described in the methods section;
  - are tests one-sided or two-sided?
  - are there adjustments for multiple comparisons?
  - exact statistical test results, e.g., P values = x but not P values < x;
  - definition of 'center values' as median or average;
  - definition of error bars as s.d. or s.e.m.

Please complete ALL of the questions below.
Select "Not Applicable" only when the requested information is not relevant for your study.

Materials

Newly Created Materials	Information included in the manuscript?	In which section is the information available? (Reagents and Tools Table, Materials and Methods, Figures, Data Availability Section)
New materials and reagents need to be available; do any restrictions apply?	Not Applicable	

Antibodies	Information included in the manuscript?	In which section is the information available? (Reagents and Tools Table, Materials and Methods, Figures, Data Availability Section)
For **antibodies** provide the following information:<br>- Commercial antibodies: RRID (if possible) or supplier name, catalogue number and or/clone number<br>- Non-commercial: RRID or citation	Yes	Described in the methods

DNA and RNA sequences	Information included in the manuscript?	In which section is the information available? (Reagents and Tools Table, Materials and Methods, Figures, Data Availability Section)
**Short novel DNA or RNA including primers, probes**: provide the sequences.	Not Applicable	

Cell materials	Information included in the manuscript?	In which section is the information available? (Reagents and Tools Table, Materials and Methods, Figures, Data Availability Section)
**Cell lines:** Provide species information, strain. Provide accession number in repository **OR** supplier name, catalog number, clone number, and/**OR** RRID.	Not Applicable	
**Primary cultures:** Provide species, strain, sex of origin, genetic modification status.	Yes	Information provided in the methods
Report if the cell lines were recently **authenticated** (e.g., by STR profiling) and tested for mycoplasma contamination.	Not Applicable	

Experimental animals	Information included in the manuscript?	In which section is the information available? (Reagents and Tools Table, Materials and Methods, Figures, Data Availability Section)
**Laboratory animals or Model organisms:** Provide species, strain, sex, age, genetic modification status. Provide accession number in repository **OR** supplier name, catalog number, clone number, **OR** RRID.	Yes	Information provided in the methods
**Animal observed in or captured from the field:** Provide species, sex, and age where possible.	Not Applicable	
Please detail **housing and husbandry conditions**.	Yes	Information provided in the methods

Plants and microbes	Information included in the manuscript?	In which section is the information available? (Reagents and Tools Table, Materials and Methods, Figures, Data Availability Section)
**Plants:** provide species and strain, ecotype and cultivar where relevant, unique accession number if available, and source (including location for collected wild specimens).	Not Applicable	
**Microbes:** provide species and strain, unique accession number if available, and source.	Not Applicable	

Human research participants	Information included in the manuscript?	In which section is the information available? (Reagents and Tools Table, Materials and Methods, Figures, Data Availability Section)
If collected and within the bounds of privacy constraints report on age, sex and gender or ethnicity for all study participants.	Not Applicable	

Core facilities	Information included in the manuscript?	In which section is the information available? (Reagents and Tools Table, Materials and Methods, Figures, Data Availability Section)
If your work benefited from core facilities, was their service mentioned in the acknowledgments section?	Not Applicable	

Design

Study protocol	Information included in the manuscript?	In which section is the information available? (Reagents and Tools Table, Materials and Methods, Figures, Data Availability Section)
If study protocol has been **pre-registered, provide DOI in the manuscript**. For clinical trials, provide the trial registration number **OR** cite DOI.	Not Applicable	
Report the **clinical trial registration number** (at ClinicalTrials.gov or equivalent), where applicable.	Not Applicable	

Laboratory protocol	Information included in the manuscript?	In which section is the information available? (Reagents and Tools Table, Materials and Methods, Figures, Data Availability Section)
Provide DOI OR other citation details if **external detailed step-by-step protocols** are available.	Not Applicable	

Experimental study design and statistics	Information included in the manuscript?	In which section is the information available? (Reagents and Tools Table, Materials and Methods, Figures, Data Availability Section)
Include a statement about **sample size** estimate even if no statistical methods were used.	Yes	Information provided in the methods
Were any steps taken to minimize the effects of subjective bias when allocating animals/samples to treatment (e.g. **randomization procedure**)? If yes, have they been described?	Not Applicable	
Include a statement about **blinding** even if no blinding was done.	Not Applicable	
Describe **inclusion/exclusion criteria** if samples or animals were excluded from the analysis. Were the criteria pre-established?<br><br>If sample or data points were omitted from analysis, report if this was due to attrition or intentional exclusion and provide justification.	Not Applicable	
For every figure, are **statistical tests** justified as appropriate? Do the data meet the assumptions of the tests (e.g., normal distribution)? Describe any methods used to assess it. Is there an estimate of variation within each group of data? Is the variance similar between the groups that are being statistically compared?	Yes	Information provided in the methods and Figure legends

Sample definition and in-laboratory replication	Information included in the manuscript?	In which section is the information available? (Reagents and Tools Table, Materials and Methods, Figures, Data Availability Section)
In the figure legends: state number of times the experiment was **replicated** in laboratory.	Yes	Information provided in the Figure legends
In the figure legends: define whether data describe **technical or biological replicates**.	Yes	Information provided in the Figure legends

Ethics

Ethics	Information included in the manuscript?	In which section is the information available? (Reagents and Tools Table, Materials and Methods, Figures, Data Availability Section)
Studies involving **human participants**: State details of **authority granting ethics approval** (IRB or equivalent committee(s), provide reference number for approval.	Not Applicable	
Studies involving **human participants**: Include a statement confirming that **informed consent** was obtained from all subjects and that the experiments conformed to the principles set out in the WMA Declaration of Helsinki and the Department of Health and Human Services Belmont Report.	Not Applicable	
Studies involving **human participants: For** publication of **patient photos**, include a statement confirming that consent to publish was obtained.	Not Applicable	
Studies involving experimental **animals**: State details of **authority granting ethics approval** (IRB or equivalent committee(s), provide reference number for approval. Include a statement of compliance with ethical regulations.	Yes	Information provided in the Methods
Studies involving **specimen and field samples:** State if relevant **permits** obtained, provide details of authority approving study; if none were required, explain why.	Not Applicable	

Dual Use Research of Concern (DURC)	Information included in the manuscript?	In which section is the information available? (Reagents and Tools Table, Materials and Methods, Figures, Data Availability Section)
Could your study fall under dual use research restrictions? Please check biosecurity documents and list of **select agents and toxins** (CDC): https://www.selectagents.gov/sat/list.htm	Not Applicable	
If you used a select agent, is the security level of the lab appropriate and reported in the manuscript?	Not Applicable	
If a study is subject to dual use research of concern regulations, is the name of the **authority granting approval and reference number** for the regulatory approval provided in the manuscript?	Not Applicable	

Reporting

The MDAR framework recommends adoption of discipline-specific guidelines, established and endorsed through community initiatives. Journals have their own policy about requiring specific guidelines and recommendations to complement MDAR.

Adherence to community standards	Information included in the manuscript?	In which section is the information available? (Reagents and Tools Table, Materials and Methods, Figures, Data Availability Section)
State if relevant guidelines or checklists (e.g., **ICMJE, MIBBI, ARRIVE, PRISMA**) have been followed or provided.	Not Applicable	
For **tumor marker prognostic studies,** we recommend that you follow the **REMARK** reporting guidelines (see link list at top right). See author guidelines, under 'Reporting Guidelines'. Please confirm you have followed these guidelines.	Not Applicable	
For **phase II and III randomized controlled trials**, please refer to the **CONSORT** flow diagram (see link list at top right) and submit the CONSORT checklist (see link list at top right) with your submission. See author guidelines, under 'Reporting Guidelines'. Please confirm you have submitted this list.	Not Applicable	

Data Availability

Data availability	Information included in the manuscript?	In which section is the information available? (Reagents and Tools Table, Materials and Methods, Figures, Data Availability Section)
Have **primary datasets** been deposited according to the journal's guidelines (see 'Data Deposition' section) and the respective accession numbers provided in the Data Availability Section?	Not Applicable	
Were **human clinical and genomic datasets** deposited in a public access-controlled repository in accordance to ethical obligations to the patients and to the applicable consent agreement?	Not Applicable	
Are **computational models** that are central and integral to a study available without restrictions in a machine-readable form? Were the relevant accession numbers or links provided?	Not Applicable	
If publicly available data were reused, provide the respective **data citations in the reference list**.	Not Applicable	
